# Catalyst-controlled regioselectivity in phosphine catalysis: the synthesis of spirocyclic benzofuranones *via* regiodivergent [3 + 2] annulations of aurones and an allenoate†
†Electronic supplementary information (ESI) available. CCDC 1517706 and 1517707. For ESI and crystallographic data in CIF or other electronic format see DOI: 10.1039/c7sc02176c



**DOI:** 10.1039/c7sc02176c

**Published:** 2017-06-12

**Authors:** Huanzhen Ni, Zhaoyuan Yu, Weijun Yao, Yu Lan, Nisar Ullah, Yixin Lu

**Affiliations:** a Graduate School for Integrative Sciences & Engineering (NGS) , National University of Singapore , #05-01, 28 Medical Drive , 117456 , Singapore . Email: chmlyx@nus.edu.sg; b Department of Chemistry , National University of Singapore , 3 Science Drive 3 , 117543 , Singapore; c School of Chemistry and Chemical Engineering , Chongqing University , Chongqing 400030 , P. R. China . Email: lanyu@cqu.edu.cn; d Department of Chemistry , Zhejiang Sci-Tech University , 310018 , P. R. China; e Chemistry Department , King Fahd University of Petroleum and Materials , Dhahran 31261 , Saudi Arabia . Email: nullah@kfupm.edu.sa; f National University of Singapore (Suzhou) Research Institute , 377 Lin Quan Street, Suzhou Industrial Park , Suzhou , Jiangsu 215123 , P. R. China

## Abstract

Catalyst-controlled regiodivergent [3 + 2] annulations of aurones and allenoates have been developed. When a dipeptide phosphine catalyst with an l-d- configuration was employed, α-selective [3 + 2] annulation products could be obtained with good regioselectivities and enantioselectivities. With the employment of l-l- dipeptide phosphines, γ-selective annulation products could be selectively obtained with excellent enantioselectivities. By simply tuning the catalyst configurations, a wide range of α-selective or γ-selective spirocyclic benzofuranones with either aryl or alkyl substitutions could be readily prepared. DFT calculations suggest that the conformation of the dipeptide phosphines influences the hydrogen bonding interactions or the distortion energy, resulting in delicate energy differentiation in the transition states, and accounting for the observed regioselectivity.

## Abstract 2

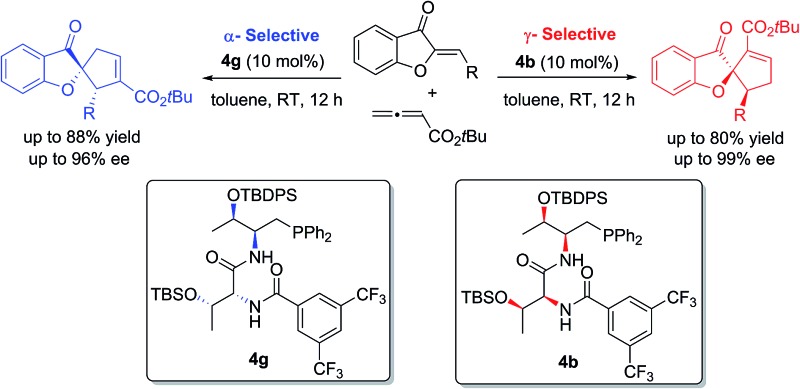
Catalyst-controlled regiodivergent [3 + 2] annulations of aurones and allenoates have been developed.

## 1. Introduction

**Scheme 1:**
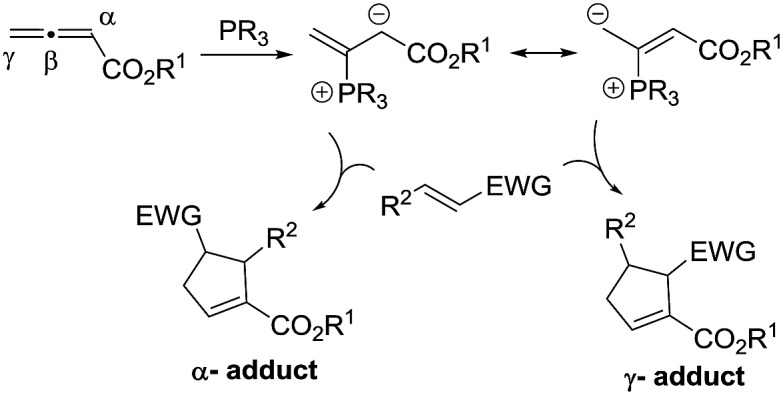
Regioselectivity in Lu’s [3 + 2] annulation between allenoates and activated alkenes.

The past decade has witnessed the blossoming of enantioselective nucleophilic phosphine catalysis.^1^ Among the wide range of phosphine-mediated asymmetric processes, phosphine-catalyzed annulations^2–4^ are arguably the most important reactions in synthetic organic chemistry. Ever since Lu’s seminal discovery of the phosphine-catalyzed [3 + 2] annulation of electron-deficient allenes with activated olefins in 1995,^2*a*^ this powerful mode of cyclization has attracted enormous attention from synthetic organic chemists and has now become a common method for the construction of 5-membered ring systems. In a typical phosphine-catalyzed [3 + 2] annulation reaction^5^ between an allenoate and an activated alkene, the phosphine adds on to the allene and forms a zwitterionic intermediate, which has two resonance forms, and their reactions with activated olefins lead to the formation of α- or γ- regioisomers (Scheme 1). In the reported phosphine-catalyzed [3 + 2] annulation reactions, α-adducts and γ-adducts are often mixed. In most cases, the α-adducts can be obtained as the major or sole regioisomer. There are only a handful of examples describing the asymmetric formation of γ-selective regioisomers in [3 + 2] annulation processes.^2g,2j,2k^ While the issue of regioselectivity is not particularly attended, it appears that the employment of different activated olefin substrates is the key to the observed γ-selectivity in those studies.

**Scheme 2:**
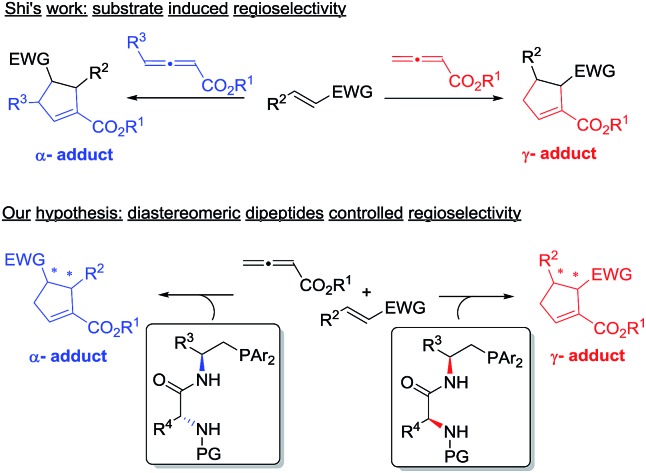
Controlling regioselectivity in [3 + 2] annulations.

Given the widespread use of phosphine-mediated annulation reactions for ring construction, and the fact that obtaining different regioisomers in annulation reactions in an uncontrolled manner impedes the efficiency of these processes, it was quite surprising to note that the regioselectivity issue in phosphine-catalyzed [3 + 2] cycloadditions has not drawn much investigation. The only report^2*u*^ devoting efforts to obtain both α- and γ- regioisomers is a study by Shi and co-workers, in which they employed either simple or γ-substituted allenoates as annulation partners in order to obtain different regioisomers (Scheme 2). It is certainly not very desirable that different substrates have to be prepared in order to access different regioisomers. Moreover, the requirement of employing different allene/olefin reaction partners limits the general applicability of the annulation methods. We aimed to address this challenging issue by developing a general strategy to access different regioisomers from the same starting materials, *i.e.* without varying the allenes and olefins in phosphine-triggered [3 + 2] annulation reactions. Building upon our previous success of dipeptide-based phosphines,^6^ we hypothesize that different regioisomers may be obtained by employing different diastereomeric dipeptide phosphine catalysts (Scheme 2). We envision that the ready tunability of dipeptide structures in phosphine catalysts may be utilized to provide not only efficient stereochemical control, but also serve as an effective means to differentiate pathways leading to the divergent formation of regioisomers.

Aurones are an important class of unique natural products exhibiting remarkable biological activities,^7^ and they are widely used as synthetic building blocks.^8^ However, the applications of aurones in phosphine catalysis are unknown. As part of our continuous efforts in asymmetric phosphine catalysis,^9^ we envisioned that aurones could be used as C_2_ synthons in [3 + 2] annulation with allenes, for the creation of structurally unique spiroaurone motifs. In this article, we document the first catalyst-controlled regiodivergent [3 + 2] annulations of aurones; by employing dipeptide phosphines with either an l-d- or an l-l- configuration, the annulation of aurones with allenoates yielded either α- or γ-selective spirobenzofuranones in a highly enantioselective and diastereoselective manner.

## 2. Results and discussion

### 2.1 Tuning the configurations of dipeptide phosphines for different regioselectivity

We initiated our investigation by examining the catalytic effects of a number of amino acid-derived phosphines on the annulation reaction between aurone **1a** and allenoate **2a**, and the results are summarized in Table 1. Mono-amino acid-derived phosphines led to the formation of products with a certain degree of regioselectivity and moderate enantioselectivity (entries 1–6). We were delighted to discover that dipeptide phosphine catalysts were efficient in promoting regiodivergent cyclizations. l-Val-l-thr-derived **4a**, l-thr-l-thr-based **4b**, and l-thr-l-val-derived **4c** all favored the formation of γ-adducts, which were obtained with excellent enantioselectivity (entries 7–9). Interestingly, when dipeptide catalysts **4d**, **4e**, **4f**, and **4g** with an l-d- configuration were used, α-selective cycloaddition products were constantly obtained as the major regioisomer with excellent enantioselectivity (entries 10–13). The regioselectivity of the annulations was further enhanced by performing a quick solvent screening (entries 14–18). Under optimal conditions, the [3 + 2] annulation reaction catalyzed by l-thr-d-thr-based **4g** in ether afforded regioisomers in a ratio of 13 : 1, favoring the α-isomer (94% ee) (entry 14), whereas the reaction promoted by l-thr-l-thr-derived **4b** in CH_2_Cl_2_ led to the selective formation of the α-annulation product (*α*/*γ* = 1 : 6, 98% ee for γ-isomer) (entry 17).

**Table 1:** [3 + 2] Annulation of aurone **1a** with allenoate **2a**: catalyst screening^*a*^

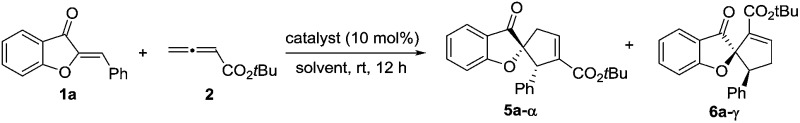					
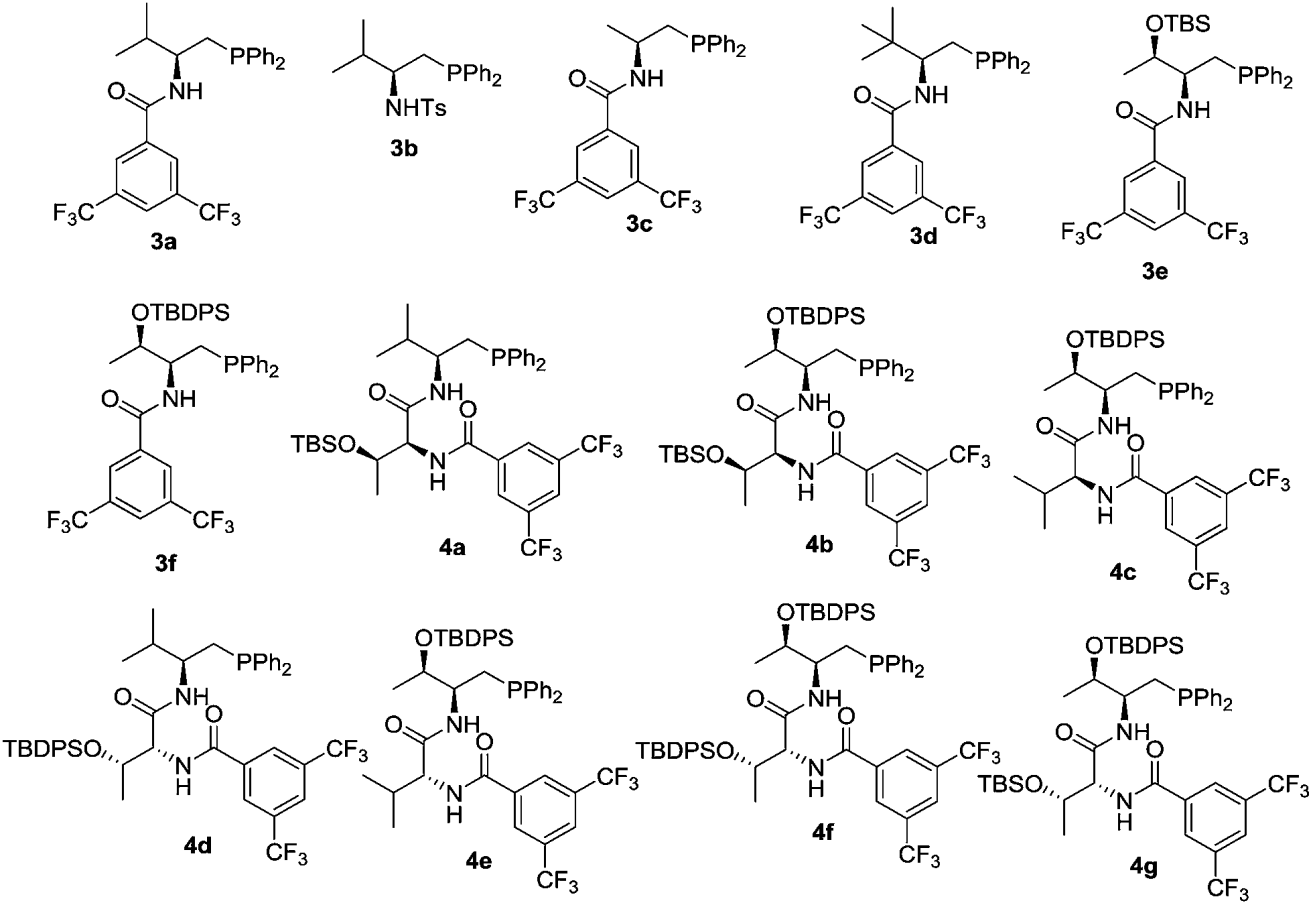					
Entry	Cat.	Solvent	**5a** : **6a** ^*b*^	Yield^*c*^ (%)	ee^*d*^ (%)
1	**3a**	Toluene	3 : 1	68	52
2	**3b**	Toluene	2 : 1	42	1
3	**3c**	Toluene	1 : 1	23	34
4	**3d**	Toluene	2 : 1	58	79
5	**3e**	Toluene	3 : 1	70	64
6	**3f**	Toluene	3 : 1	69	74
7	**4a**	Toluene	1 : 4	72	97
8	**4b**	Toluene	1 : 4	74	98
9	**4c**	Toluene	1 : 3.5	70	92
10	**4d**	Toluene	2 : 1	52	79
11	**4e**	Toluene	6 : 1	78	93
12	**4f**	Toluene	5 : 1	76	94
13	**4g**	Toluene	6 : 1	80	96
14	**4g**	**Ether**	**13 : 1**	**88**	**94**
15	**4g**	CH_2_Cl_2_	4 : 1	73	91
16	**4g**	EtOAc	19 : 1	92	90
17	**4b**	**CH**_**2**_ **Cl**_**2**_	**1 : 6**	**80**	**98**
18	**4b**	CHCl_3_	1 : 5	76	97

^*a*^Reactions were performed with **1a** (0.10 mmol), **2a** (0.12 mmol) and the catalyst (0.01 mmol) in the solvent specified (1 mL) at room temperature.

^*b*^Determined by crude ^1^H NMR analysis.

^*c*^Isolated yield for the major regioisomer.

^*d*^The ee value for the major regioisomer, determined by HPLC analysis on a chiral stationary phase.

### 2.2 The α-selective and γ-selective [3 + 2] annulations: the substrate scope

The scope of this regiodivergent [3 + 2] annulation was subsequently probed. When different substituted aurones **1** were reacted with allenoate **2a** in the presence of l-thr-d-thr-based **4g** in ether, the α-adducts were formed selectively (Table 2). The reaction was applicable to various aryl-substituted aurones, regardless of the substitution patterns and electronic properties of the aryl moiety, and excellent enantioselectivities and very good regioselectivities were attainable (entries 1–12). Aurones with an aliphatic substituent could also be used, albeit lower α-selectivities were observed (entries 13–16). In all the examples examined, the pure α-adducts could be isolated mostly in good yields and with excellent enantioselectivities.

**Table 2:** The α-selective [3 + 2] annulation of aurones **1** with allenoate **2a** ^*a*^

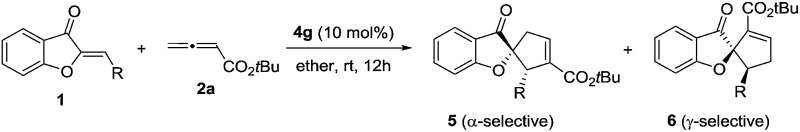					
Entry	R (**1**)	**5** : **6** ^*b*^	**5**	Yield^*c*^ (%)	ee^*d*^ (%)
1	Ph (**1a**)	13 : 1	**5a**	88	94
2	4-Cl-C_6_H_4_ (**1b**)	9 : 1	**5b**	81	93
3	3-Cl-C_6_H_4_ (**1c**)	6 : 1	**5c**	75	91
4	2-Cl-C_6_H_4_ (**1d**)	7 : 1	**5d**	76	91
5	4-F-C_6_H_4_ (**1e**)	9 : 1	**5e**	83	94
6	4-OMe-C_6_H_4_ (**1f**)	10: 1	**5f**	87	96
7	4-Me-C_6_H_4_ (**1g**)	8 : 1	**5g**	76	95
8	2-Me-C_6_H_4_ (**1h**)	15 : 1	**5h**	85	97
9	4-CN-C_6_H_4_ (**1i**)	13 : 1	**5i**	69	93
10	2-Naphthyl (**1j**)	5 : 1	**5j**	74	94
11	3,4-(OMe)_2_-C_6_H_4_ (**1k**)	5 : 1	**5k**	80	96
12	2-Thienyl (**1l**)	12 : 1	**5l**	73	95
13^*e*^	Cyclohexyl (**1m**)	3 : 1	**5m**	62	95
14^*e*^	Isopropyl (**1n**)	3 : 1	**5n**	53	94
15^*e*^	*n*Bu (**1o**)	5 : 1	**5o**	40	94
16^*e*^	Ethyl (**1p**)	6 : 1	**5p**	60	96

^*a*^Reactions were performed with **1** (0.10 mmol), **2a** (0.12 mmol) and **4g** (0.01 mmol) in ether (1 mL) at room temperature.

^*b*^Determined by crude ^1^H NMR analysis.

^*c*^Isolated yield for the pure α-regioisomer.

^*d*^The ee value for the α-regioisomer, determined by HPLC analysis on a chiral stationary phase.

^*e*^The catalyst loading was 20 mol%.

The scope of γ-selective [3 + 2] annulation between substituted aurones **1** and allenoate **2a** in the presence of l-thr-l-thr-based **4b** was next investigated (Table 3). Similarly, aurones with simple/fused aryl and heterocyclic substituents (entries 1–12) and alkyl substituents (entries 13–16) were shown to be suitable, and pure γ-adducts were generally isolated in good yields. Notably, the enantioselectivities for the above γ-selective [3 + 2] cyclization were extremely high, between 96% to 99% ee. The absolute configurations of the α-selective and γ-selective products were assigned based on the X-ray crystallographic analysis of the products **5b** ^10^ and **6b**,^11^ respectively.

**Table 3:** The γ-selective [3 + 2] annulation of aurones **1** with allenoate **2a** ^*a*^

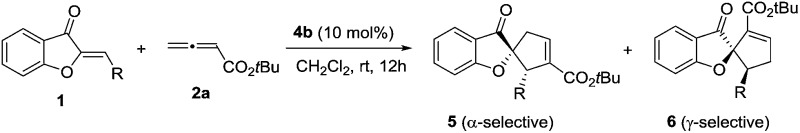					
Entry	R (1)	**5** : **6** ^*b*^	**6**	Yield^*c*^ (%)	ee^*d*^ (%)
1	Ph (**1a**)	1 : 6	**6a**	80	98
2	4-Cl-C_6_H_4_ (**1b**)	1 : 5	**6b**	72	98
3	3-Cl-C_6_H_4_ (**1c**)	1 : 3	**6c**	63	98
4	2-Me-C_6_H_4_ (**1k**)	1 : 4	**6d**	67	99
5	4-Br-C_6_H_4_ (**1s**)	1 : 4	**6e**	70	98
6	4-OMe-C_6_H_4_ (**1f**)	1 : 5	**6f**	80	99
7	4-Me-C_6_H_4_ (**1g**)	1 : 5	**6g**	74	99
8	3-Me-C_6_H_4_ (**1r**)	1 : 6	**6h**	78	98
9	4 F-C_6_H_4_ (**1e**)	1 : 3	**6i**	64	96
10	2-Naphthyl (**1j**)	1 : 5	**6j**	75	98
11	3,4-(OMe)_2_-C_6_H_4_ (**1k**)	1 : 6	**6k**	70	99
12	2-Thienyl (**1l**)	1 : 6	**6l**	70	99
13^*e*^	Cyclohexyl (**1m**)	1 : 7	**6m**	68	98
14^*e*^	Isopropyl (**1n**)	1 : 6	**6n**	74	98
15^*e*^	*n*Bu (**1o**)	1 : 3	**6o**	35	97
16^*e*^	Ethyl (**1p**)	1 : 4	**6p**	40	98

^*a*^Reactions were performed with **1** (0.10 mmol), **2a** (0.12 mmol) and **4g** (0.01 mmol) in CH_2_Cl_2_ (1 mL) at room temperature.

^*b*^Determined by crude ^1^H NMR analysis.

^*c*^Isolated yield for the pure γ-regioisomer.

^*d*^The ee value for the γ-regioisomer, determined by HPLC analysis on a chiral stationary phase.

^*e*^Catalyst loading was 20 mol%.

**Scheme 3:**
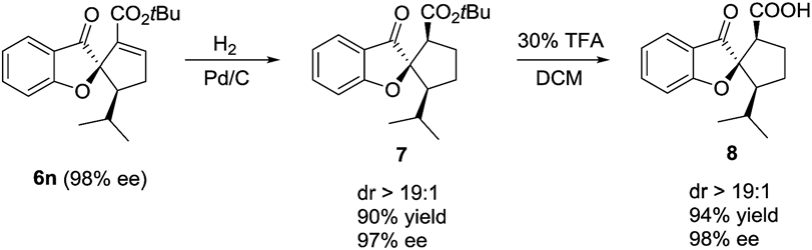
Deriving an anti-fungi analog from the annulation product.

It is noteworthy that the spiro[benzofuran-2,1′-cyclopentane] motif prepared in the above [3 + 2] annulation reaction is widely present in many natural products and bioactive molecules, and thus is of great significance in medicinal chemistry.^12^ As an illustration (Scheme 3), the γ-adduct **6n** was readily converted in a highly diastereoselective and enantio-retentive manner to **8**, a close analogue of a bioactive natural product extracted from fungi.^12*a*^

### 2.3 Theoretical studies to understand the origin of the observed regioselectivities

**Scheme 4:**
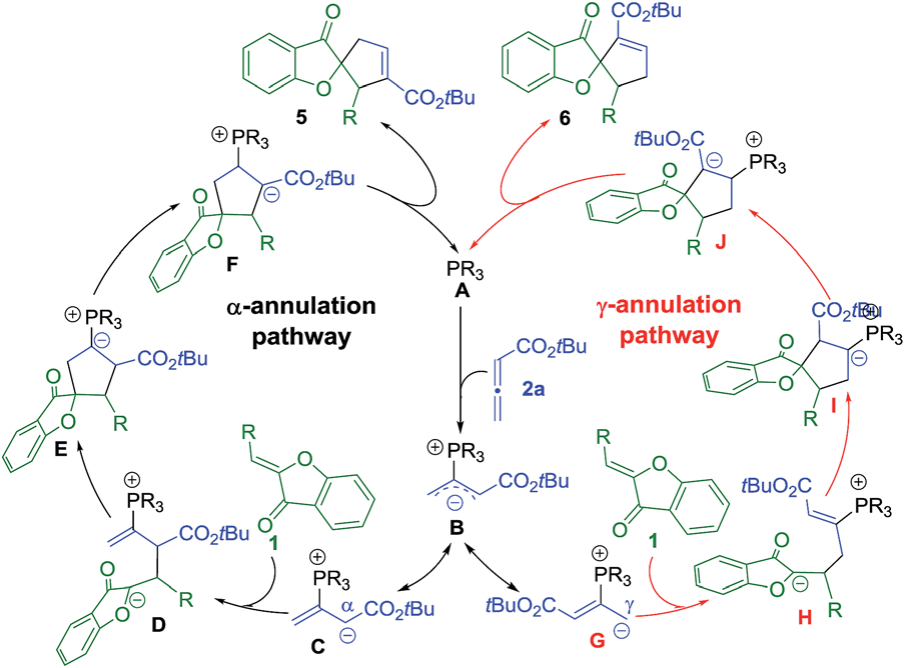
Proposed mechanism for the phosphine-catalyzed [3 + 2] annulation of aurones with allenoate **2a**.

The mechanism of the phosphine-catalyzed [3 + 2] annulation reaction between aurone and allene is shown in Scheme 4, which follows the general pathways commonly accepted in the literature.^5^ The nucleophilic attack of the phosphine catalyst **A** on allene **2a** yields zwitterionic intermediate **B**, which has two resonance forms with the negative charge either delocalized on the α-carbon (**C**) or the γ-carbon (**G**). The subsequent [3 + 2] annulation of **C** or **G** with aurone **1** then affords advanced intermediate **E** or **I**. The following proton transfer process, regeneration of the phosphine catalyst, and formation of the desired α-selective (**5**) or γ-selective (**6**) products complete the catalytic cycle.

Density functional theory (DFT) calculations were performed to gain insight into the catalyst-controlled regioselectivity in bifunctional phophine-catalyzed [3 + 2] annulation.^13^ Aurone **1a** and allene **2a** were chosen for our theoretical studies, and the phosphines **4c** and **4e** were selected since they offered similar regioselectivities to those of **4b** and **4g** in the annulation reactions, but possess slightly simpler structures. The Gibbs free energy profiles of the **4c** or **4e**-catalyzed [3 + 2] cycloaddition of aurone **1a** to allenoate **2a** were calculated, and we focused on the addition step of the phosphonium zwitterionic intermediate **C** or **G** to aurone **1a** to understand the observed regioselectivity.

**Fig. 1:**
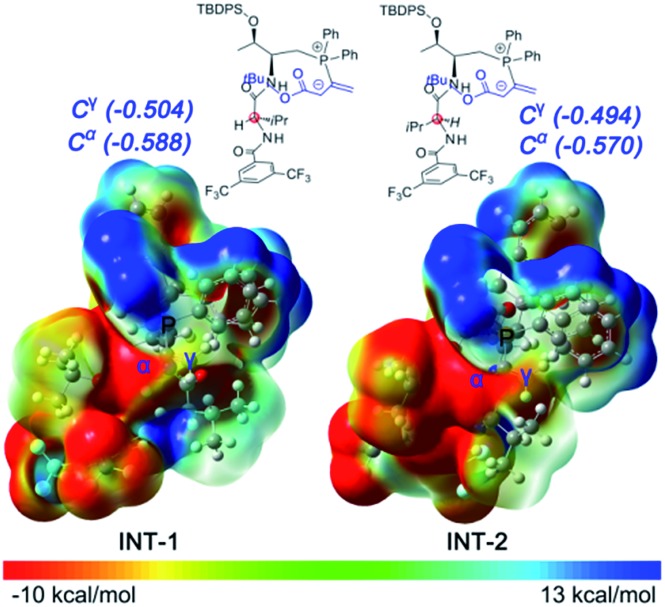
The B3LYP calculated NPA charge distributions for intermediate **INT-1** and **INT-2**.

Initially, we suspected that the electron density of the phosphonium enolate may influence the regioselectivity, therefore we calculated the electrostatic potential (ESP) surface and nature population analysis (NPA) charge distribution for the **4e**-derived **INT-1** and **4c**-derived **INT-2** zwitterionic intermediates. Both the ESP and NPA calculations showed that the negative charges of C-α and C-γ in **INT-1**/**INT-2** are close, therefore the difference of reactivity for C-α and C-γ is not the reason behind the observed regioselectivitiy (Fig. 1).

**Fig. 2:**
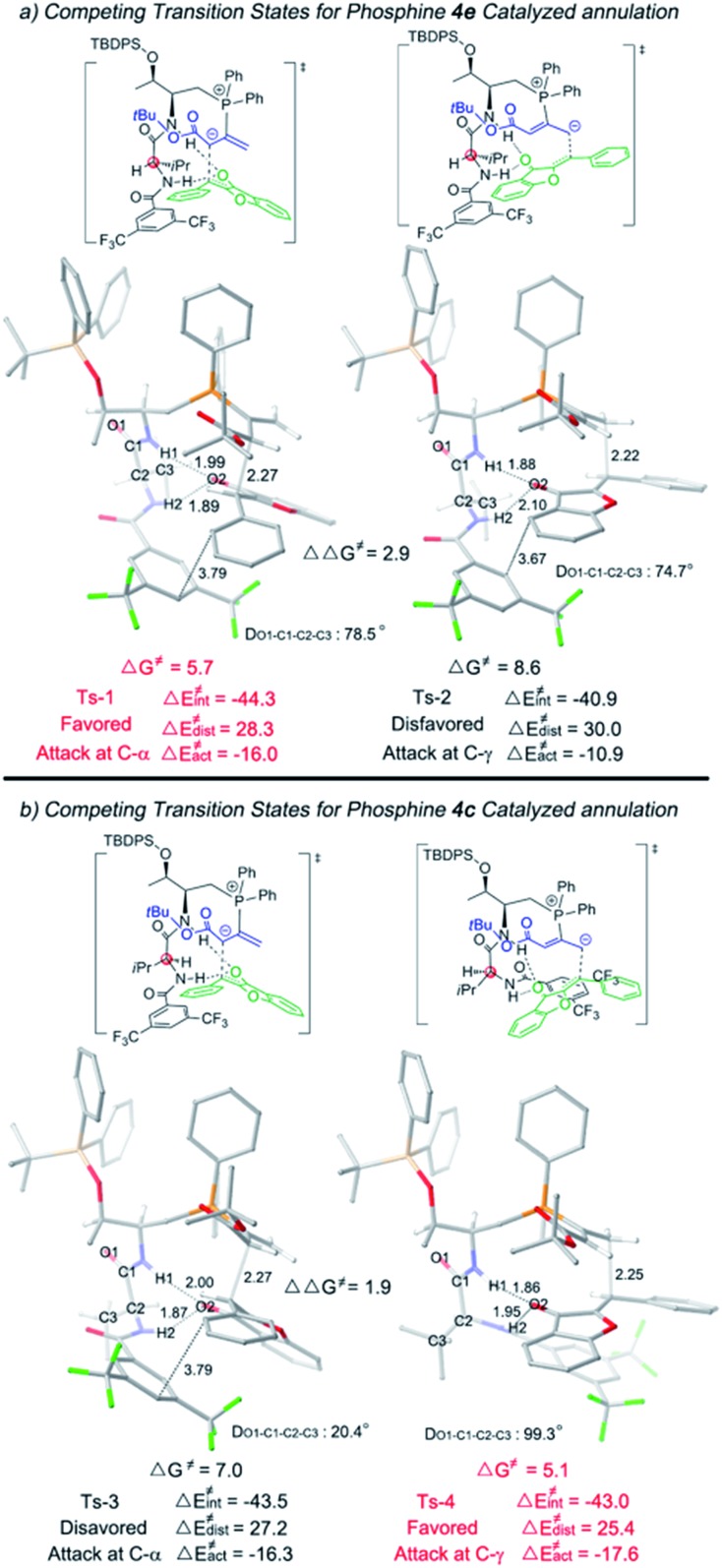
Optimized transition states **Ts-1**, **Ts-2**, **Ts-3** and **Ts-4**. The relative free energies are given in kilocalories per mole.

We then applied a distortion/interaction model^14^ (Δ*E*≠act = Δ*E*≠dist + Δ*E*≠int) utilizing phosphonium allenoate and aurone as two fragments to gain more mechanistic insights. For the annulation reaction catalyzed by l-d-dipeptide phosphine **4e** (Fig. 2a), the difference of the distortion energy terms (Δ*E*≠dist) between **Ts-1** and **Ts-2** is only 1.7 kcal mol^–1^. However, the difference of the interaction energy terms (Δ*E*≠int) between those two transition states is 3.4 kcal mol^–1^, which suggests that the interaction energy played a key role in determining the regioselectivity of the reaction. In the α-attack pathway (**Ts-1**), the aurone is activated by two hydrogen bonds with bond lengths of 1.89 Å and 1.99 Å, respectively. However, in the γ-attack pathway (**Ts-2**), the two bond distances become 1.88 Å and 2.10 Å, suggesting that one hydrogen bond is weaker. The strength of the hydrogen bond is determined by the conformation of the l-d- dipeptide. In **Ts-1**, the dihedral angle of O1-C1-C2-C3 is 78.5°, indicating that the isopropyl group is almost perpendicular to the amide moiety when the H2···O2 hydrogen bond is formed. On the other hand, a smaller dihedral angle of 74.7° is observed in **Ts-2**, and the strain of the isopropyl group in the valine residue results in the H2 atom in the valine residue rotating far away from the O2 atom of the aurone moiety, thus leading to a weaker H2···O2 hydrogen bond. The more favorable hydrogen bonding interactions, resulting from the conformation of the l-d- dipeptide moiety in the advanced phosphonium enolate intermediate, account for the observed α-selectivity in the annulation reaction.

In the [3 + 2] annulation promoted by the l-l- dipeptide phosphine **4c** (Fig. 2b), the activation energy of the γ-addition pathway (**Ts-4**) is more favored than the α-addition (**Ts-3**) by 1.9 kcal mol^–1^. The conformation of the l-l- dipeptide phosphine again accounts for the energy difference in the two transition states. In **Ts-3**, the dihedral angle of O1-C1-C2-C3 is only 20.4°, which exhibits a strong steric repulsion between the O1 atom and isopropyl group. Whereas in **Ts-4**, the amino moiety is rotated clockwise about 80° to form the H2···O2 hydrogen bond, thus the isopropyl group is perpendicular to the amide moiety, leading to an O1-C1-C2-C3 dihedral angle of 99.3° and a smaller distortion energy, meaning that the γ-isomer is selectively formed in the cyclization reaction.

## 3. Conclusions

In conclusion, we have utilized aurones as C_2_ synthons in phosphine catalysis for the first time. We have also successfully developed the first catalyst-controlled regiodivergent [3 + 2] annulation reaction. By simply utilizing dipeptide phosphines with either an l-l- or l-d- configuration, the γ-selective or α-selective annulation products could be readily obtained with excellent enantioselectivity. DFT calculations suggest that the observed catalyst-controlled α/γ- regioselectivity is determined by the conformation of the dipeptide phosphine catalysts, which differentiates the distortion energy or hydrogen bonding interactions in the competing transition state pathways, thus favoring the formation of specific regioisomers. Currently, we are extending our findings in this report to the discovery of other regiodivergent processes in asymmetric phosphine catalysis.
