# Supplementary material for: Catalyst-controlled regioselectivity in phosphine catalysis: the synthesis of spirocyclic benzofuranones via regiodivergent [3 + 2] annulations of aurones and an allenoate
Source: Chem Sci. 2017 Jun 12;8(8):5699–704. doi: 10.1039/c7sc02176c (PMC5621158; doi:10.1039/c7sc02176c)

Electronic Supplementary Material (ESI) for Chemical Science.
This journal is © The Royal Society of Chemistry 2017

Catalyst-controlled Regioselectivity in Phosphine Catalysis: Synthesis of Spirocyclic Benzofuranones via Regiodivergent [3+2] Annulations of Aurones and an Allenoate

Huanzhen Ni,[a][b] Zhaoyuan Yu,[b][c] Weijun Yao,[d] Yu Lan,*[c] Nisar Ullah,*[e] and Yixin Lu*[a][b][f]

[a]Graduate School for Integrative Sciences & Engineering (NGS), Centre for Life Sciences (CeLS), National University of Singapore, #05-01, 28 Medical Drive, 117456 Singapore

[b]Department of Chemistry, National University of Singapore, 3 Science Drive 3, 117543 Singapore.

[c] School of Chemistry and Chemical Engineering, Chongqing University, Chongqing 400030, P. R. China.

[d] Department of chemistry, Zhejiang Sci-Tech University, 310018, P. R. China.

[e]Chemistry Department, King Fahd University of Petroleum and Materials, Dhahran 31261, Saudi Arabia.

[f]National University of Singapore (Suzhou) Research Institute, 377 Lin Quan Street, Suzhou Industrial Park, Suzhou, Jiangsu, PR China, 215123

Email: chmlyx@nus.edu.sg; lanyu@cqu.edu.cn; nullah@kfupm.edu.sa

Supporting Information

A.	General information	S2
B.	Representative Procedure	S2
C.	Synthesis of dipeptide phosphine catalysts	S3
D.	Analytical data and HPLC Chromatogram of the Products	S4
E.	Synthesis of product **8**	S45
F.	Determination of stereochemistry of product **8** by 2D NMR	S48
G.	Computational methods	S49
H.	Proposed Reaction Cycle and DFT studies	S50
I.	Calculated Gibbs free energy profiles for the phosphine-catalyzed [3+2] annulation of aurone **1a** and allene **2a**	S51
J.	B3LYP and M11 absolute calculation energies, enthalpies, and free energies.	S55
K.	B3LYP geometries for all the optimized compounds and transition states.	S57
L.	References	S114
M.	NMR Spectra of the Products	S116

A. General Information

Unless otherwise specified, all reactions were carried out under a nitrogen atmosphere in anhydrous conditions. All the solvents were purified according to the standard procedures. All chemicals which are commercially available were used without further purification unless otherwise noted. Thin-layer chromatography (TLC) was performed on silica gel plates (60F-254) using UV-light (254 and 365 nm). Flash chromatography was conducted on silica gel (200–300 mesh). ^{1}H and ^{13}C NMR spectra were recorded at ambient temperature in $CDCl_3$ on a Bruker AMX500 (500 MHz) spectrometer. Chemical shifts were reported in parts per million (ppm). All high resolution mass spectra were obtained on a Finnigan/MAT 95XL-T spectrometer. Optical rotations were measured using a Jasco DIP-1000 polarimeter. Enantiomeric excesses were determined by HPLC analysis on a chiral stationary phase.

Catalyst **3** and **4** were synthesized by following our previously reported procedures.[1] Aurones **1** were synthesized according to literature reported procedures.[2]

B. Representative Procedure

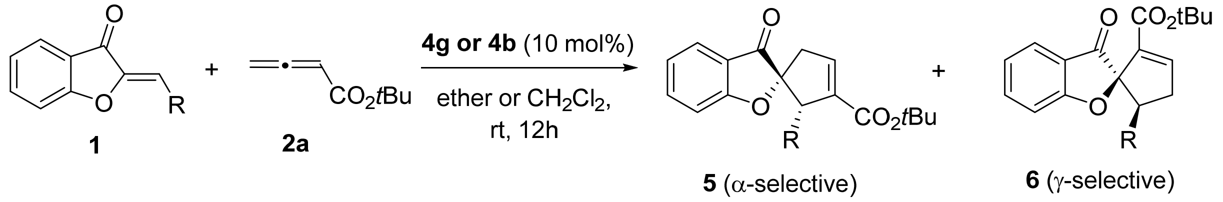

To a dried round bottle flask with a magnetic stirring bar under N_2 at room temperature were added aurones **1** (0.1 mmol) and allenoate **2a** (0.12 mmol), followed by the addition of anhydrous ether (0.5 mL, for α-selective reaction) or CH_2Cl_2 (0.5 mL, for γ-selective reaction). Catalyst **4g** (0.01 mmol, 10 mg, for α-selective reaction) or **4b** (0.01 mmol, 10 mg, for γ-selective reaction) was then introduced, and the reaction mixture was stirred for 12 hours. The solvent was then removed under reduced pressure and crude product was dissolved in $CDCl_3$ to for ^{1}H-NMR to analyze the ratio for product **5** and **6**. The crude product was then purified by column chromatography (Hexane: ether = 10:1) on silica gel to afford the major annulation adducts **5** or **6**.

C. Synthesis of dipeptide phosphine catalysts

Dipeptide catalysts were synthesized according to the previous reported procedure.[1]

Catalyst **4c**: a white solid; ^{1}H NMR (500 MHz, acetone-d_6) δ 8.56 (s, 2H), 8.21 (s, 1H), 7.69 (ddd, *J* = 7.1 Hz, 5.8 Hz, 1.3 Hz, 4H), 7.48–7.40 (m, 7H), 7.39–7.28 (m, 10H), 4.61–4.50 (m, 1H), 4.14 (qd, *J* = 6.2 Hz, 2.8 Hz, 1H), 4.07 (ddd, *J* = 11.6 Hz, 8.9 Hz, 4.5 Hz, 1H), 2.57 (ddd, *J* = 13.7 Hz, 4.1 Hz, 2.9 Hz, 1H), 2.40 (ddd, *J* = 13.6 Hz, 10.5Hz, 3.0 Hz, 1H), 2.31 (dq, *J* = 13.7 Hz, 6.8 Hz, 1H), 1.08 (d, *J* = 6.7 Hz, 3H), 1.05 (d, *J* = 6.8 Hz, 3H), 1.02 (s, 9H), 0.94 (d, *J* = 6.2 Hz, 3H); ^{13}C NMR (125 MHz, acetone-d_6) δ 170.42, 163.94, 139.26, 139.15, 138.65, 138.53, 136.94, 135.89, 135.86, 134.09, 133.42, 133.26, 133.10, 132.32, 132.17, 131.48, 131.21, 129.72, 129.65, 128.82, 128.52, 128.46, 128.41, 128.31, 128.19, 128.17, 127.65, 127.50, 124.74, 124.43, 122.26, 71.00, 70.93, 59.69, 51.97, 51.84, 30.54, 26.61, 19.44, 19.42, 18.98, 18.46, 18.02; ^{31}P NMR (202 MHz, acetone-d_6) δ -22.24; HRMS (ESI) m/z calcd for $C_{46}H_{50}F_6N_2O_3PSi$ $[M + H]^+$ = 851.3227, found = 851.3229.

Catalyst **4e**: a white solid; ^{1}H NMR (500 MHz, acetone-d_6) δ 8.60 (s, 2H), 8.28–8.17 (m, 2H), 7.72 (dt, *J* = 8.0 Hz, 1.2 Hz, 4H), 7.46 (ddd, *J* = 9.9 Hz, 7.3 Hz, 5.8 Hz, 2H), 7.40 (dd, *J* = 11.2 Hz, 4.3 Hz, 5H), 7.37–7.24 (m, 10H), 4.59 (dd, *J* = 8.5 Hz, 7.2 Hz, 1H), 4.16 (qd, *J* = 6.2 Hz, 2.3 Hz, 1H), 4.10–4.00 (m, 1H), 2.49 (ddd, *J* = 13.7 Hz, 4.8 Hz, 1.9 Hz, 1H), 2.40–2.24 (m, 2H), 1.01 (m, *J* = 10.6 Hz, 5.0 Hz, 18H); ^{13}C NMR (125 MHz, acetone-d_6) δ 170.09, 164.12, 138.89, 138.77, 138.65, 137.02, 135.89, 134.06, 133.40, 133.03, 132.88, 132.35, 132.20, 131.47, 131.21, 129.82, 129.74, 128.67, 128.40, 128.39, 128.34, 128.30, 128.27, 127.70, 127.53, 124.77, 124.45, 122.28, 71.16, 71.10, 59.52, 51.88, 51.75, 30.98, 30.87, 30.57, 26.59, 19.15, 19.13, 18.98, 17.81; ^{31}P NMR (202 MHz, acetone-d_6) δ -22.68; HRMS (ESI) m/z calcd for $C_{46}H_{50}F_6N_2O_3PSi$ $[M + H]^+$ = 851.3227, found = 851.3223.

Catalyst **4g**: a white solid; ^{1}H NMR (500 MHz, acetone-d_6) δ 8.55 (s, 2H), 8.25 (s, 1H), 8.07 (d, *J* = 7.6 Hz, 1H), 7.74–7.67 (m, 4H), 7.52–7.44 (m, 2H), 7.44–7.36 (m, 8H), 7.35–7.27 (m, 6H), 7.19 (d, *J* = 9.1 Hz, 1H), 4.64 (dd, *J* = 7.6 Hz, 4.2 Hz, 1H), 4.49–4.40 (m, 1H), 4.21 (qd, *J* = 6.2 Hz, 2.7 Hz, 1H), 4.18–4.07 (m, 1H), 2.59 (dd, *J* = 13.8 Hz, 4.2 Hz, 1H), 2.30 (ddd, *J* = 13.7 Hz, 9.8 Hz, 1.8 Hz, 1H), 1.22 (d, *J* = 6.3 Hz, 3H), 1.05–0.99 (m, 12H), 0.91 (s, 9H), 0.17 (d, *J* = 7.1 Hz, 6H); ^{13}C NMR (125 MHz, acetone-d_6) δ 168.30, 163.88, 138.73, 138.61, 138.49, 136.82, 135.87,

133.97, 133.28, 132.94, 132.78, 132.55, 132.40, 131.58, 131.31, 129.86, 129.78, 128.72, 128.57, 128.48, 128.43, 128.15, 127.72, 127.57, 124.96, 124.40, 122.24, 70.66, 70.60, 68.47, 59.09, 52.10, 51.98, 30.21, 30.10, 26.64, 25.46, 19.14, 18.96, 18.74, 17.72, -5.23, -5.36; ^{31}P NMR (202 MHz, Acetone-d_6) δ -22.60; HRMS (ESI) m/z calcd for $C_{51}H_{62}F_6N_2O_4PSi_2$ $[M + H]^+$ = 967.3884, found = 967.3877.

D. Analytical Data and HPLC Chromatogram of the Products

tert-Butyl (2*R*,2'*S*) 3-oxo-2'-phenyl-3*H*-spiro[benzofuran-2,1'-cyclopentan]-3'-ene-3'-carboxylate **5a**

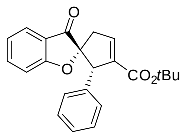

A white solid; $[\alpha]^{25}_{D}$ = 182.4 (c 1.0, $CHCl_3$); 1H NMR (500 MHz, $CDCl_3$) δ 7.63–7.58 (m, 1H), 7.45–7.40 (m, 1H), 7.14–7.10 (m, 3H), 7.01 (dd, *J* = 7.4 Hz, 1.7 Hz, 2H), 6.97 (t, *J* = 7.4 Hz, 1H), 6.93 (q, *J* = 2.5 Hz, 1H), 6.69 (d, *J* = 8.4 Hz, 1H), 4.54 (d, *J* = 1.9 Hz, 1H), 3.16 (dt, *J* = 18.9 Hz, 2.0 Hz, 1H), 2.90 (ddd, *J* = 18.9 Hz, 2.5 Hz, 1.5 Hz, 1H), 1.22 (s, 9H)·; ^{13}C NMR (125 MHz, $CDCl_3$) δ 202.59, 171.22, 163.13, 139.25, 138.53, 138.14, 135.45, 128.72, 127.50, 126.89, 124.08, 121.76, 120.74, 112.88, 95.44, 80.75, 59.82, 41.90, 27.71; HRMS (ESI) m/z calcd for $C_{23}H_{22}NaO_4$ $[M + Na]^+$ = 385.1410, found = 385.1417; The ee value was 94%, t_R (major) = 6.831 min, t_R (minor) = 19.539 min (Chiralpak IC, λ = 254 nm, 10% *i*-PrOH/hexane, flow rate = 1.0 mL/min).

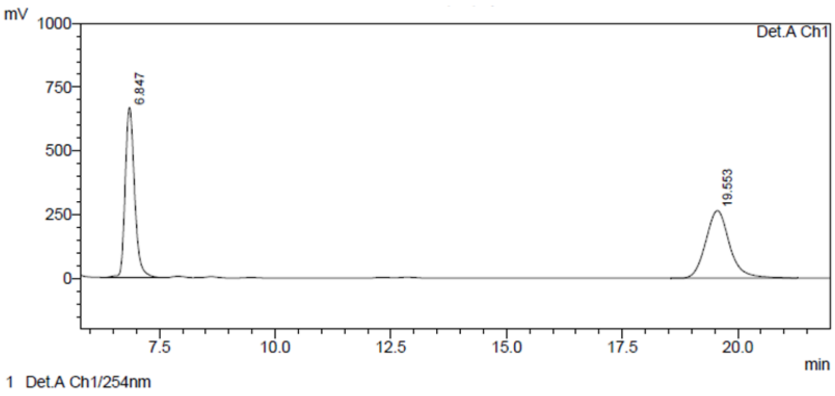

PeakTable

Detector A Ch1 254nm

Peak#	Ret. Time	Area	Height	Area %	Height %
1	6.847	9434122	666863	49.753	71.543
2	19.553	9527706	265246	50.247	28.457
Total		18961828	932110	100.000	100.000

racemic **5a**

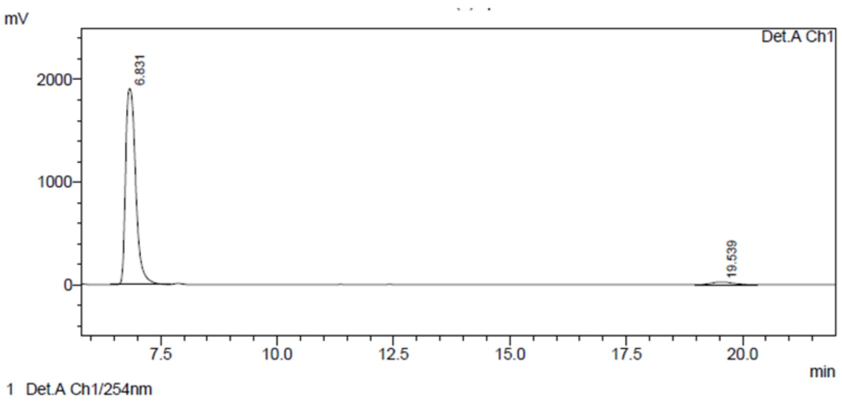

PeakTable

Detector A Ch1 254nm

Peak#	Ret. Time	Area	Height	Area %	Height %
1	6.831	28727966	1908107	97.164	98.662
2	19.539	838576	25868	2.836	1.338
Total		29566543	1933975	100.000	100.000

enantiomeric enriched **5a**

<u>*tert*-Butyl (2*R*,2'*S*) 2'-(4-chlorophenyl)-3-oxo-3*H*-spiro[benzofuran-2,1'-cyclopentan]-3'-ene-3'-carboxylate</u> **5b**

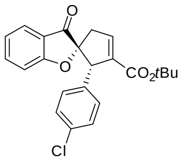

A white solid; $[\alpha]^{25}{}_{D}$ = 212.6 (c 1.0, $CHCl_3$); ^{1}H NMR (500 MHz, $CDCl_3$) δ 7.61 (d, *J* = 7.7 Hz, 1H), 7.49–7.44 (m, 1H), 7.12 (d, *J* = 8.4 Hz, 2H), 7.00 (t, *J* = 7.4 Hz, 1H), 6.96 (d, *J* = 8.3 Hz, 2H), 6.93 (q, *J* = 2.5 Hz, 1H), 6.73 (d, *J* = 8.4 Hz, 1H), 4.51 (d, *J* = 1.5 Hz, 1H), 3.15 (dt, *J* = 18.9 Hz, 2.0 Hz, 1H), 2.89 (ddd, *J* = 18.9 Hz, 2.5 Hz, 1.5 Hz, 1H); ^{13}C NMR (125 MHz, Acetone-d_6) δ 196.35, 163.14, 162.16, 161.20, 148.73, 137.47, 136.85, 136.83, 132.87, 132.27, 131.24, 129.49, 129.43, 128.72, 128.63, 128.58, 128.16, 119.13, 118.16, 115.58, 115.40, 88.79, 38.93, 31.46, 13.12; HRMS (ESI) m/z calcd for $C_{23}H_{21}ClNaO_4$ $[M + Na]^+$ = 419.1021, found = 419.1027; The ee value was 93%, t_R (major) = 14.390 min, t_R (minor) = 17.577 min (Chiralpak IC, λ = 254 nm, 1% *i*-PrOH/hexane, flow rate = 1.0 mL/min).

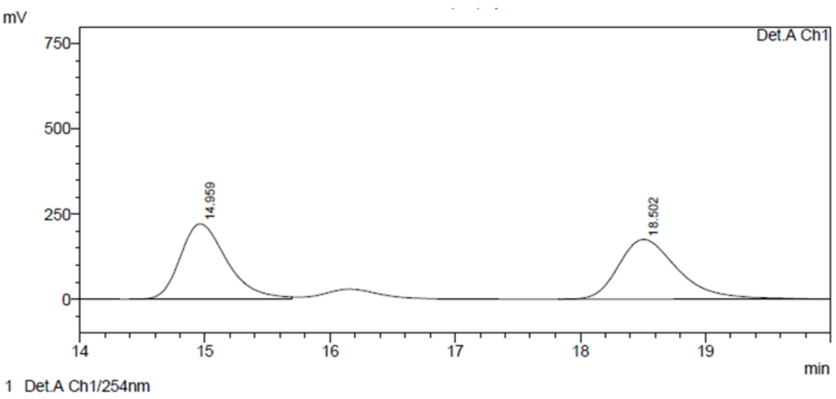

PeakTable

Detector A Ch1 254nm

Peak#	Ret. Time	Area	Height	Area %	Height %
1	14.959	5774469	221384	50.045	55.811
2	18.502	5764106	175285	49.955	44.189
Total		11538575	396669	100.000	100.000

Racemic **5b**

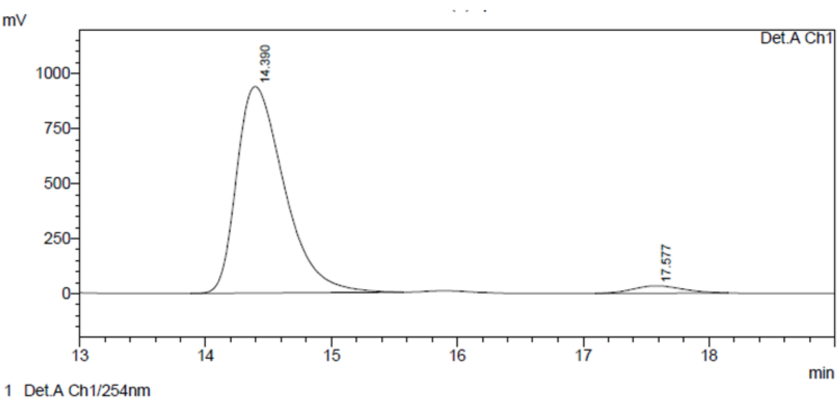

PeakTable

Detector A Ch1 254nm

Peak#	Ret. Time	Area	Height	Area %	Height %
1	14.390	24712315	938720	96.445	96.577
2	17.577	910874	33275	3.555	3.423
Total		25623189	971995	100.000	100.000

Enantioenriched **5b**

<u>*tert*-Butyl (2*R*,2'*S*) 2'-(3-chlorophenyl)-3-oxo-3*H*-spiro[benzofuran-2,1'-cyclopentan]-3'-ene-3'-carboxylate **5c**</u>

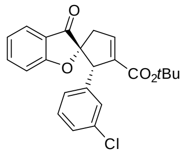

A white solid; $[\alpha]^{25}_{D}$ = 193.5 (c 1.0, $CHCl_3$); ^{1}H NMR (500 MHz, $CDCl_3$) δ 7.65–7.59 (m, 1H), 7.51–7.42 (m, 1H), 7.13–7.08 (m, 1H), 7.05 (t, *J* = 7.7 Hz, 2H), 7.00 (t, *J* = 7.5 Hz, 1H), 6.95 (q, *J* = 2.5 Hz, 1H), 6.88 (d, *J* = 7.6 Hz, 1H), 6.74 (dd, *J* = 8.5 Hz, 0.5 Hz, 1H), 4.50 (d, *J* = 1.7 Hz, 1H), 3.16 (dt, *J* = 18.9 Hz, 2.0 Hz, 1H), 2.94–2.86 (m, 1H), 1.25

(s, 9H); ^{13}C NMR (125 MHz, $CDCl_3$) δ 202.16, 171.12, 162.80, 140.02, 138.41, 137.89, 137.68, 133.47, 128.90, 128.76, 127.12, 126.98, 124.21, 122.03, 120.62, 112.95, 95.12, 81.05, 59.22, 41.98, 27.74; HRMS (ESI) m/z calcd for $C_{23}H_{21}ClNaO_4$ $[M + Na]^+$ = 419.1021, found = 419.1026; The ee value was 91%, t_R (major) = 6.095 min, t_R (minor) = 7.988 min (Chiralpak IC, λ = 254 nm, 10% *i*-PrOH/hexane, flow rate = 1.0 mL/min).

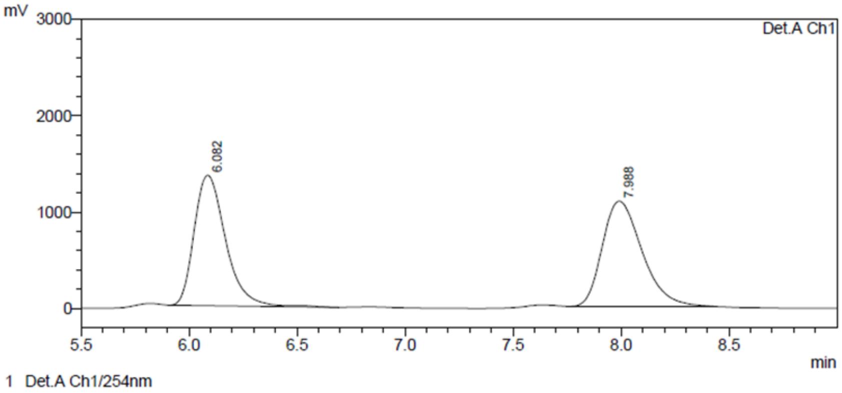

PeakTable

Detector A Ch1 254nm

Peak#	Ret. Time	Area	Height	Area %	Height %
1	6.082	13733811	1352842	49.552	55.328
2	7.988	13982086	1092276	50.448	44.672
Total		27715898	2445118	100.000	100.000

Racemic **5c**

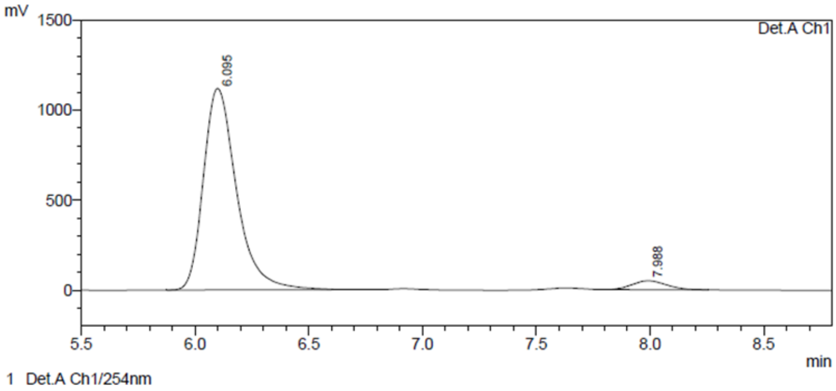

PeakTable

Detector A Ch1 254nm

Peak#	Ret. Time	Area	Height	Area %	Height %
1	6.095	11338304	1119632	95.627	95.776
2	7.988	518541	49380	4.373	4.224
Total		11856845	1169013	100.000	100.000

Enantioenriched **5c**

tert-Butyl (2*R*,2'*S*) 2'-(2-chlorophenyl)-3-oxo-3*H*-spiro[benzofuran-2,1'-cyclopentan]-3'-ene-3'-carboxylate **5d**

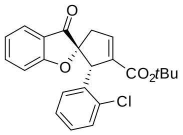

Yellow oil; $[\alpha]^{25}_{D}$ = 145.6 (c 1.0, $CHCl_3$); ^{1}H NMR (500 MHz, $CDCl_3$) δ 7.67 (dd, *J* = 7.7, 0.7 Hz, 1H), 7.46 (ddd, *J* = 8.5 Hz, 7.3 Hz, 1.4 Hz, 1H), 7.25 (dd, *J* = 7.7 Hz, 1.6 Hz, 1H), 7.22–7.17 (m, 1H), 7.14 (dd, *J* = 7.9 Hz, 1.3 Hz, 1H), 7.12–7.07 (m, 1H), 7.05–7.01 (m, 1H), 6.99 (q, *J* = 2.5 Hz, 1H), 6.70 (d, *J* = 8.4 Hz, 1H), 5.15 (d, *J* = 2.2 Hz, 1H), 3.25–3.16 (m, 1H), 2.99–2.87 (m, 1H), 1.22 (s, 9H); ^{13}C NMR (125 MHz, $CDCl_3$) δ 202.47, 171.01, 162.68, 139.94, 138.24, 138.01, 134.34, 134.09, 130.30, 128.75, 128.13, 125.90, 124.39, 122.01, 120.59, 112.70, 94.08, 80.87, 55.21, 42.30, 27.67; HRMS (ESI) m/z calcd for $C_{23}H_{21}ClNaO_4$ $[M + Na]^+$ = 419.1021, found = 419.1028; The ee value was 91%, t_R (major) = 10.081 min, t_R (minor) = 12.981 min (Chiralpak IC, λ = 254 nm, 5% *i*-PrOH/hexane, flow rate = 1.0 mL/min).

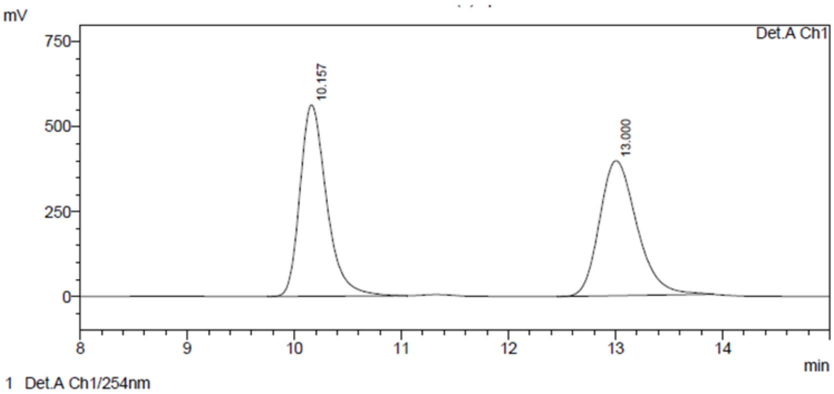

PeakTable

Detector A Ch1 254nm

Peak#	Ret. Time	Area	Height	Area %	Height %
1	10.157	9745767	563129	50.173	58.625
2	13.000	9678567	397429	49.827	41.375
Total		19424334	960557	100.000	100.000

Racemic **5d**

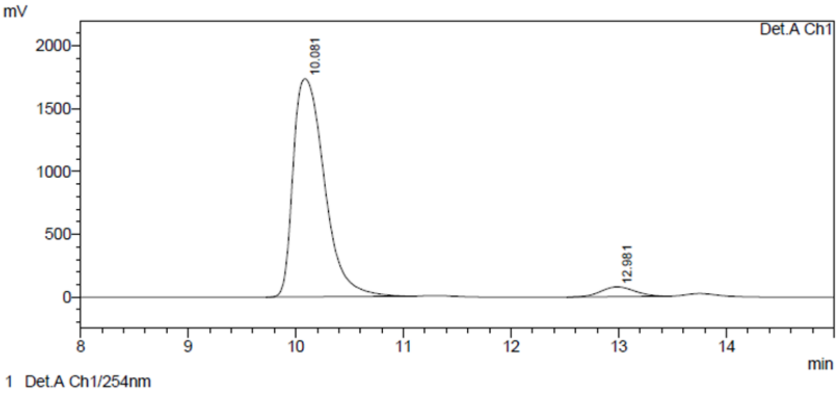

PeakTable

Detector A Ch1 254nm

Peak#	Ret. Time	Area	Height	Area %	Height %
1	10.081	35089769	1736320	95.409	95.728
2	12.981	1688321	77492	4.591	4.272
Total		36778090	1813812	100.000	100.000

Enantioenriched **5d**

tert-Butyl (2*R*,2'*S*)-2'-(4-fluorophenyl)-3-oxo-3*H*-spiro[benzofuran-2,1'-cyclopentan]-3'-ene-3'-carboxylate **5e**

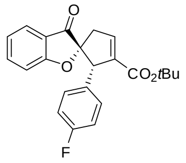

A white solid; $[\alpha]^{25}_{D}$ = 183.2 (c 1, $CHCl_3$); ^{1}H NMR (500 MHz, $CDCl_3$) δ 7.60 (dd, *J* = 7.7, 0.6 Hz, 1H), 7.45 (dd, *J* = 11.5, 4.0 Hz, 1H), 7.01 – 6.96 (m, 3H), 6.92 (q, *J* = 2.4 Hz, 1H), 6.83 (t, *J* = 8.6 Hz, 2H), 6.71 (d, *J* = 8.5 Hz, 1H), 4.53 (d, *J* = 1.7 Hz, 1H), 3.15 (dt, *J* = 18.9, 1.9 Hz, 1H), 2.89 (dd, *J* = 18.9, 1.7 Hz, 1H), 1.24 (s, 11H); ^{13}C NMR (125 MHz, $CDCl_3$) δ 202.32, 171.10, 162.95, 161.88 (d, *J* = 245.0 Hz), 139.55, 138.32, 138.23, 131.27 (d, *J* = 3.2 Hz), 130.22 (d, *J* = 8.1 Hz), 130.19, 124.10, 121.92, 120.70, 114.40 (d, *J* = 21.3 Hz), 112.88, 95.30, 80.89, 59.10, 41.80, 27.76; HRMS (ESI) m/z calcd for $C_{23}H_{21}FNaO_4$ $[M + Na]^+$ = 403.1316, found = 403.1320; The ee value was 94%, t_R (major) = 6.229 min, t_R (minor) = 8.018 min (Chiralpak IC, λ = 254 nm, 10% *i*-PrOH/hexane, flow rate = 1.0 mL/min).

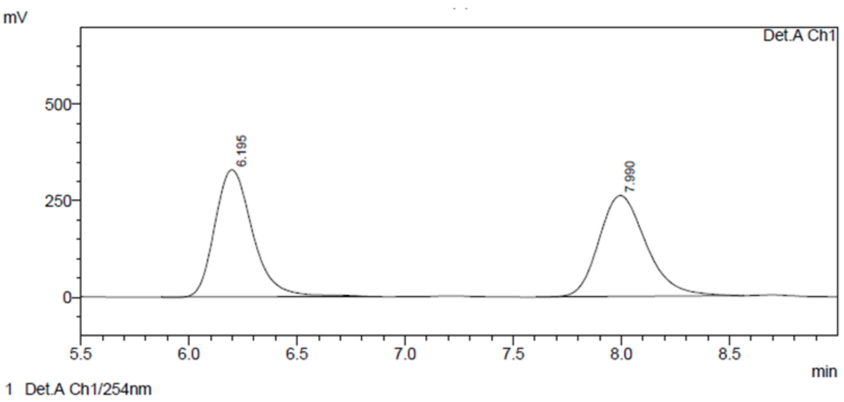

PeakTable

Detector A Ch1 254nm

Peak#	Ret. Time	Area	Height	Area %	Height %
1	6.195	4012491	329313	50.227	55.775
2	7.990	3976159	261119	49.773	44.225
Total		7988650	590433	100.000	100.000

Racemic **5e**

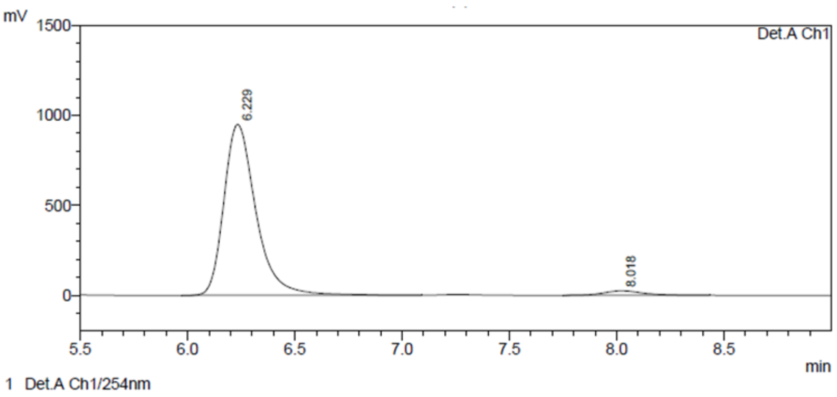

PeakTable

Detector A Ch1 254nm

Peak#	Ret. Time	Area	Height	Area %	Height %
1	6.229	9867509	949963	97.049	97.497
2	8.018	300092	24385	2.951	2.503
Total		10167601	974348	100.000	100.000

Enantioenriched **5e**

tert-Butyl (2*R*,2'*S*) 2'-(4-methoxyphenyl)-3-oxo-3*H*-spiro[benzofuran-2,1'-cyclopentan]-3'-ene-3'-carboxylate **5f**

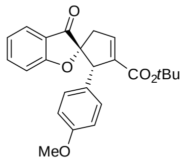

A white solid; $[\alpha]^{25}{}_{D}$ = 250.6 (c 1, $CHCl_3$); ^{1}H NMR (500 MHz, $CDCl_3$) δ 7.64–7.56 (m, 1H), 7.44 (t, *J* = 7.8 Hz, 1H), 6.98 (t, *J* = 7.5 Hz, 1H), 6.93 (d, *J* = 8.4 Hz, 2H), 6.90 (q, *J* = 2.2 Hz, 1H), 6.73 (d, *J* = 8.4 Hz, 1H), 6.68 (d, *J* = 8.4 Hz, 2H), 4.50 (d, *J* = 1.4 Hz, 1H), 3.72 (s, 3H), 3.14 (d, *J* = 18.9 Hz, 1H), 2.88 (d, *J* = 18.9 Hz, 1H), 1.25 (s, 10H); ^{13}C NMR

(125 MHz, $CDCl_3$) δ 202.74, 171.24, 163.20, 158.50, 138.94, 138.67, 138.18, 129.74, 127.60, 124.06, 121.74, 120.73, 112.98, 112.96, 95.54, 80.71, 59.10, 55.12, 41.78, 27.78; HRMS (ESI) m/z calcd for $C_{24}H_{24}NaO_5$ $[M + Na]^+$ = 415.1516, found = 415.1524; The ee value was 96%, t_R (major) = 14.121 min, t_R (minor) = 19.503 min (Chiralpak IC, λ = 254 nm, 5% *i*-PrOH/hexane, flow rate = 1.0 mL/min).

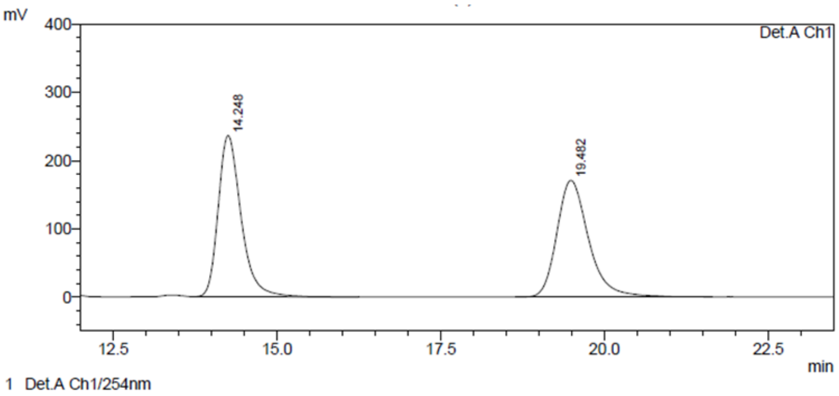

PeakTable

Detector A Ch1 254nm

Peak#	Ret. Time	Area	Height	Area %	Height %
1	14.248	5732678	236195	49.857	58.022
2	19.482	5765518	170883	50.143	41.978
Total		11498195	407079	100.000	100.000

Racemic **5f**

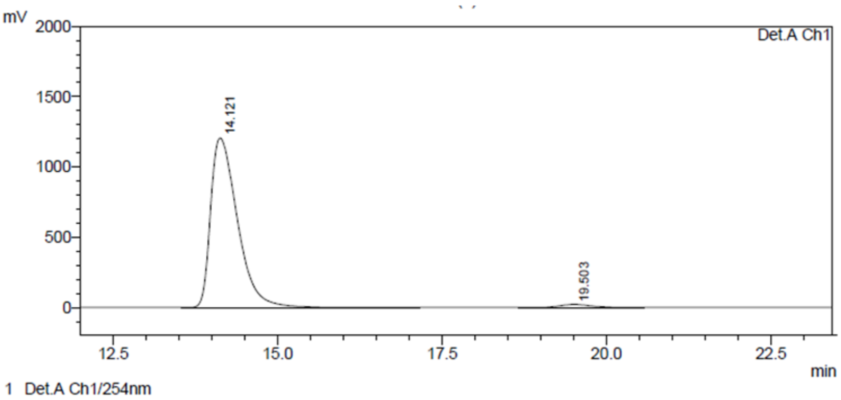

PeakTable

Detector A Ch1 254nm

Peak#	Ret. Time	Area	Height	Area %	Height %
1	14.121	34171710	1205566	97.991	98.261
2	19.503	700518	21333	2.009	1.739
Total		34872228	1226899	100.000	100.000

Enantioenriched **5f**

<u>*tert*-Butyl (2*R*,2'*S*) 3-oxo-2'-(p-tolyl)-3*H*-spiro[benzofuran-2,1'-cyclopentan]-3'-ene-3'-carboxylate **5g**</u>

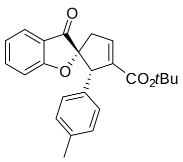

A white solid; $[\alpha]^{25}_{D}$ = 212.4 (c 1, $CHCl_3$); ^1H NMR (500 MHz, $CDCl_3$) δ 7.64–7.57 (m, 1H), 7.44 (ddd, *J* = 8.5 Hz, 7.3 Hz, 1.4 Hz, 1H), 6.98 (t, *J* = 7.4 Hz, 1H), 6.94 (d, *J* = 8.0 Hz, 2H), 6.92–6.86 (m, 3H), 6.73 (d, *J* = 8.4 Hz, 1H), 4.51 (d, *J* = 2.0 Hz, 1H), 3.14 (dt, *J* = 18.9, 2.0 Hz, 1H), 2.89 (ddd, *J* = 18.8, 2.6, 1.6 Hz, 1H), 2.23 (s, 3H), 1.25 (s, 9H); ^{13}C NMR (125 MHz, $CDCl_3$) δ 202.76, 171.28, 163.20, 138.96, 138.68, 138.12, 136.33, 132.31, 128.54, 128.24, 124.09, 121.73, 120.69, 112.98, 95.46, 80.70, 59.29, 41.89, 27.75, 21.07; HRMS (APCI) m/z calcd for $C_{24}H_{24}NaO_4$ [M + Na]$^+$ = 399.1567, found = 399.1573; The ee value was 95%, t_R (major) = 9.294 min, t_R (minor) = 17.952 min (Chiralpak IC, λ = 254 nm, 5% *i*-PrOH/hexane, flow rate = 1.0 mL/min).

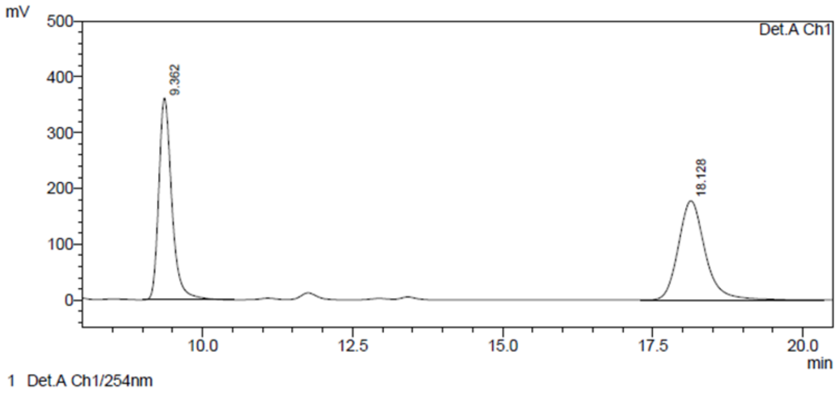

PeakTable

Detector A Ch1 254nm

Peak#	Ret. Time	Area	Height	Area %	Height %
1	9.362	5465747	361771	49.830	67.068
2	18.128	5502939	177636	50.170	32.932
Total		10968686	539407	100.000	100.000

Racemic **5g**

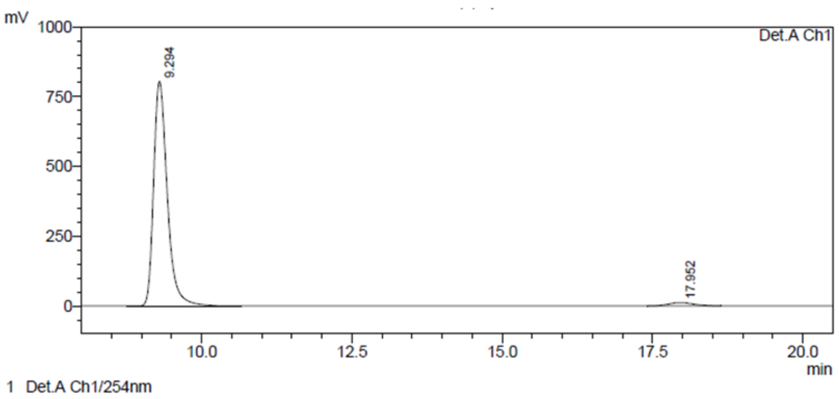

PeakTable

Detector A Ch1 254nm

Peak#	Ret. Time	Area	Height	Area %	Height %
1	9.294	12666720	804906	97.305	98.495
2	17.952	350802	12302	2.695	1.505
Total		13017522	817208	100.000	100.000

Enantioenriched **5g**

<u>*tert*-Butyl (2*R*,2'*S*) 3-oxo-2'-(o-tolyl)-3*H*-spiro[benzofuran-2,1'-cyclopentan]-3'-ene-3'-carboxylate **5h**</u>

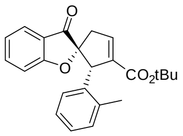

A white solid; $[\alpha]^{25}{}_{D}$ = 221.9 (c 1, $CHCl_3$); ^{1}H NMR (500 MHz, $CDCl_3$) δ 7.62 (d, *J* = 7.7 Hz, 1H), 7.47–7.41 (m, 1H), 7.19–7.14 (m, 1H), 7.10 (t, *J* = 7.4 Hz, 1H), 7.05–6.96 (m, 2H), 6.91 (dt, *J* = 5.8 Hz, 2.8 Hz, 2H), 6.72 (d, *J* = 8.4 Hz, 1H), 4.84 (d, *J* = 2.1 Hz, 1H), 3.30–3.07 (m, 1H), 3.03–2.83 (m, 1H), 2.02 (s, 3H), 1.17 (s, 9H); ^{13}C NMR (125 MHz, $CDCl_3$) δ 203.18, 171.28, 163.08, 139.48, 138.55, 138.23, 136.12, 134.07, 129.62, 129.06, 126.70, 125.15, 124.05, 121.86, 120.46, 113.00, 95.02, 80.61, 54.85, 42.19, 27.62, 19.49; HRMS (ESI) m/z calcd for $C_{24}H_{24}NaO_4$ $[M + Na]^+$ = 399.1567, found = 399.1571; The ee value was 97%, t_R (major) = 6.336 min, t_R (minor) = 10.811 min (Chiralpak IC, λ = 254 nm, 10% *i*-PrOH/hexane, flow rate = 1.0 mL/min).

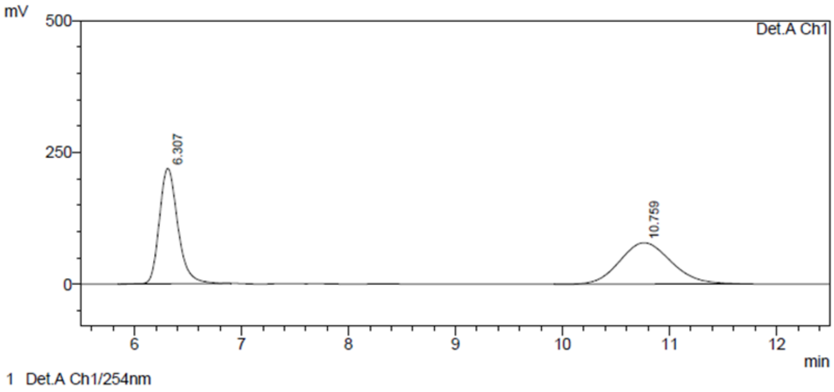

PeakTable

Detector A Ch1 254nm

Peak#	Ret. Time	Area	Height	Area %	Height %
1	6.307	2630812	219035	50.143	73.693
2	10.759	2615764	78193	49.857	26.307
Total		5246576	297228	100.000	100.000

Racemic **5h**

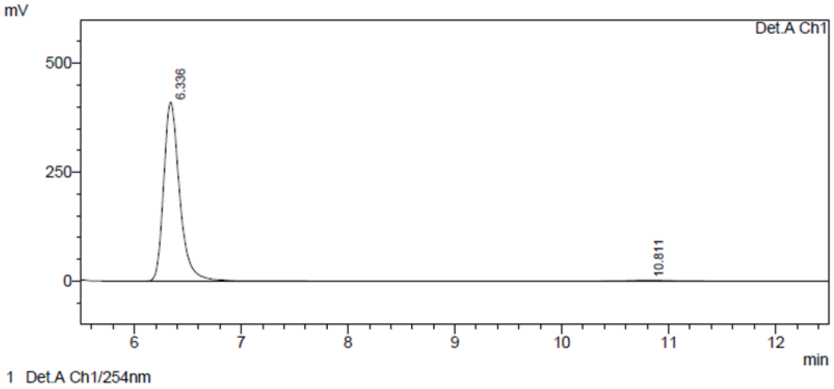

PeakTable

Detector A Ch1 254nm

Peak#	Ret. Time	Area	Height	Area %	Height %
1	6.336	4287405	411205	98.549	99.535
2	10.811	63143	1923	1.451	0.465
Total		4350548	413128	100.000	100.000

Enantioenriched **5h**

tert-Butyl (2*R*,2'*S*)-2'-(4-cyanophenyl)-3-oxo-3*H*-spiro[benzofuran-2,1'-cyclopentan]-3'-ene-3'-carboxylate **5i**

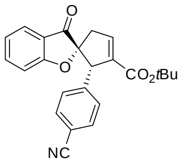

A white solid; $[\alpha]^{25}{}_{D}$ = 236.7 (c 1, $CHCl_3$); ^1H NMR (500 MHz, $CDCl_3$) δ 7.62 (d, *J* = 7.6 Hz, 1H), 7.51–7.43 (m, 3H), 7.15 (d, *J* = 8.1 Hz, 2H), 7.05–6.98 (m, 2H), 6.69 (d, *J* = 8.4 Hz, 1H), 4.59 (s, 1H), 3.19 (dt, *J* = 19.0 Hz, 1.9 Hz, 1H), 2.96–2.87 (m, 1H), 1.25 (s, 9H); ^{13}C NMR (125 MHz, $CDCl_3$) δ 201.71, 170.88, 162.51, 141.38, 140.82, 138.67,

137.23, 131.38, 129.49, 124.26, 122.29, 120.47, 118.82, 112.83, 110.84, 94.96, 81.30, 59.52, 42.12, 27.77; HRMS (ESI) m/z calcd for $C_{24}H_{21}NNaO_4$ $[M + Na]^+$ = 410.1363, found = 410.1369; The ee value was 93%, t_R (major) = 27.377 min, t_R (minor) = 30.255 min (Chiralpak IC, λ = 254 nm, 5% *i*-PrOH/hexane, flow rate = 1.0 mL/min).

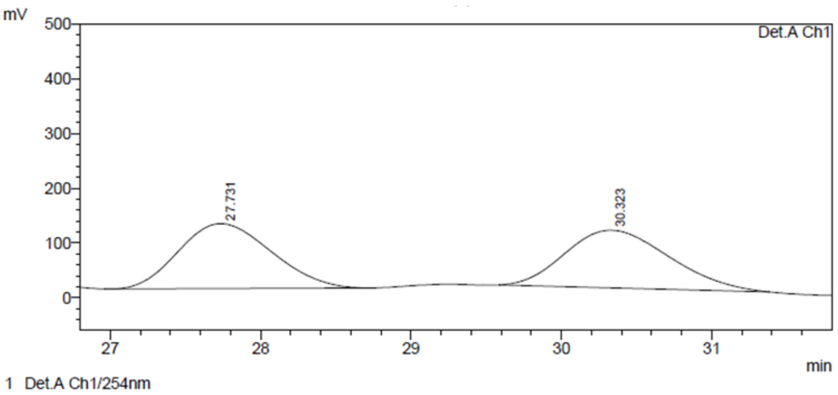

PeakTable

Detector A Ch1 254nm

Peak#	Ret. Time	Area	Height	Area %	Height %
1	27.731	5060455	118301	49.988	52.948
2	30.323	5062806	105127	50.012	47.052
Total		10123261	223428	100.000	100.000

Racemic **5i**

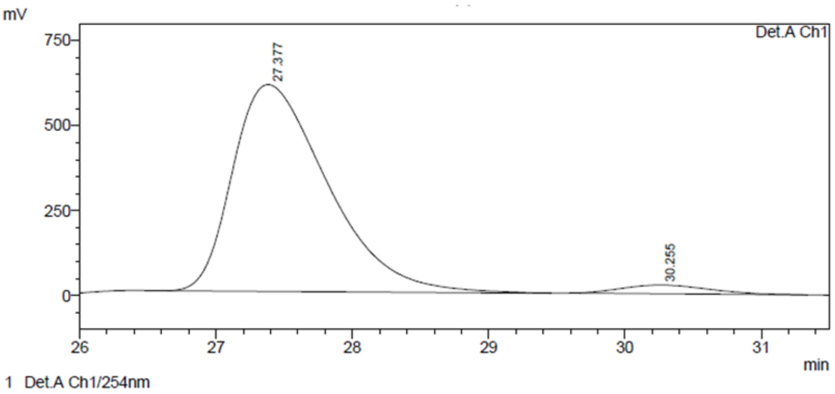

PeakTable

Detector A Ch1 254nm

Peak#	Ret. Time	Area	Height	Area %	Height %
1	27.377	29458338	608252	96.286	95.982
2	30.255	1136148	25464	3.714	4.018
Total		30594486	633715	100.000	100.000

Enantioenriched **5i**

tert-Butyl (2*R*,2'*S*)-2'-(naphthalen-2-yl)-3-oxo-3*H*-spiro[benzofuran-2,1'-cyclopentan]-3'-ene-3'-carboxylate **5j**

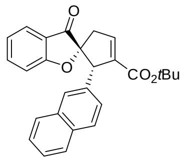

A white solid; $[\alpha]^{25}{}_{D}$ = 250.8 (c 1, $CHCl_3$); ^1H NMR (500 MHz, $CDCl_3$) δ 7.77–7.70 (m, 1H), 7.66 (m, 2H), 7.61 (dd, *J* = 7.8 Hz, 0.6 Hz, 1H), 7.47 (s, 1H), 7.43–7.36 (m, 2H), 7.36–7.30 (m, 1H), 7.21 (dd, *J* = 8.4 Hz, 1.1 Hz, 1H), 6.99 (q, *J* = 2.5 Hz, 1H), 6.92 (t, *J* = 7.5 Hz, 1H), 6.62 (d, *J* = 8.5 Hz, 1H), 4.74 (d, *J* = 1.7 Hz, 1H), 3.20 (dt, *J* = 18.8, 1.9 Hz, 1H), 3.01–2.90 (m, 1H), 1.15 (s, 9H); ^{13}C NMR (125 MHz, $CDCl_3$) δ 202.63, 171.23, 163.12, 139.47, 138.56, 138.23, 133.31, 133.02, 132.56, 127.70, 127.56, 127.45, 127.02, 126.99, 125.62, 125.43, 124.12, 121.84, 120.59, 112.95, 95.65, 80.81, 59.65, 42.21, 27.69; HRMS (ESI) m/z calcd for $C_{27}H_{24}NaO_4$ $[M + Na]^+$ = 435.1567, found = 435.1573; The ee value was 94%, t_R (major) = 9.854 min, t_R (minor) = 16.055 min (Chiralpak IC, λ = 254 nm, 5% *i*-PrOH/hexane, flow rate = 1.0 mL/min).

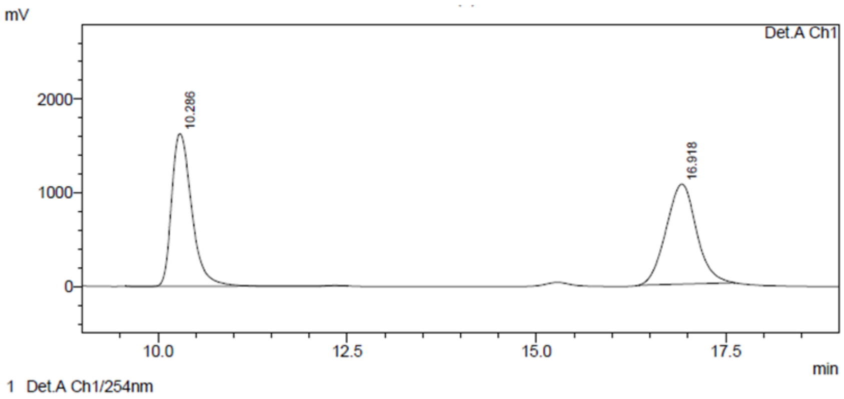

PeakTable

Detector A Ch1 254nm

Peak#	Ret. Time	Area	Height	Area %	Height %
1	10.286	29960839	1628412	50.132	60.421
2	16.918	29802593	1066683	49.868	39.579
Total		59763433	2695094	100.000	100.000

Racemic **5j**

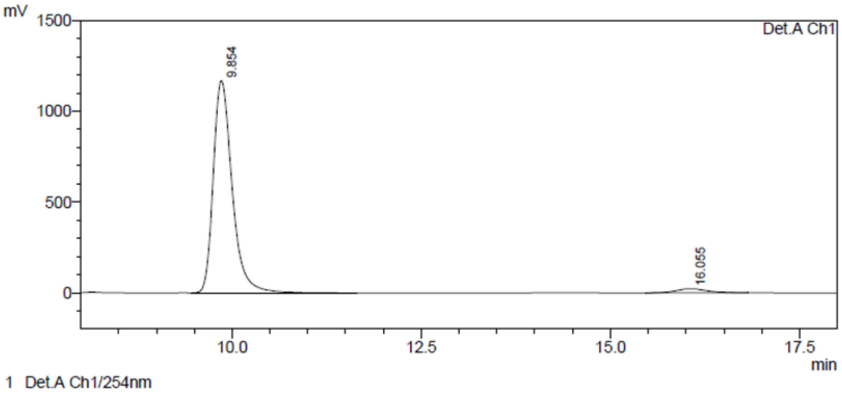

PeakTable

Detector A Ch1 254nm

Peak#	Ret. Time	Area	Height	Area %	Height %
1	9.854	20244165	1168286	97.079	98.125
2	16.055	609108	22327	2.921	1.875
Total		20853273	1190613	100.000	100.000

Enantioenriched **5j**

tert-Butyl (2*R*,2'*S*)-2'-(3,4-dimethoxyphenyl)-3-oxo-3*H*-spiro[benzofuran-2,1'-cyclopentan]-3'-ene-3'-carboxylate **5k**

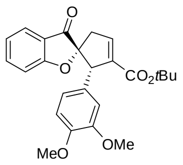

A white solid; $[\alpha]^{25}_{D}$ = 99.8 (c 1, $CHCl_3$); ^1H NMR (500 MHz, $CDCl_3$) δ 7.60 (d, *J* = 7.6 Hz, 1H), 7.47–7.39 (m, 1H), 6.98 (t, *J* = 7.5 Hz, 1H), 6.91–6.87 (m, 1H), 6.71 (d, *J* = 8.4 Hz, 1H), 6.62 (d, *J* = 8.2 Hz, 1H), 6.54 (dd, *J* = 8.2 Hz, 1.6 Hz, 1H), 6.51 (s, 1H), 4.50 (s, 1H), 3.78 (s, 3H), 3.71 (s, 3H), 3.14 (d, *J* = 18.9 Hz, 1H), 2.92–2.81 (m, 1H), 1.26 (s, 9H); ^{13}C NMR (125 MHz, $CDCl_3$) δ 202.72, 171.31, 163.26, 148.16, 147.91, 139.11, 138.44, 138.26, 127.99, 124.03, 121.79, 121.00, 120.78, 112.97, 111.87, 110.27, 95.62, 80.79, 59.55, 55.79, 55.72, 41.75, 27.81; HRMS (ESI) m/z calcd for $C_{25}H_{26}NaO_6$ $[M + Na]^+$ = 445.1622, found = 445.1628; The ee value was 96%, t_R (minor) = 12.741 min, t_R (major) = 13.965 min (Chiralpak IC, λ = 254 nm, 20% *i*-PrOH/hexane, flow rate = 1.0 mL/min).

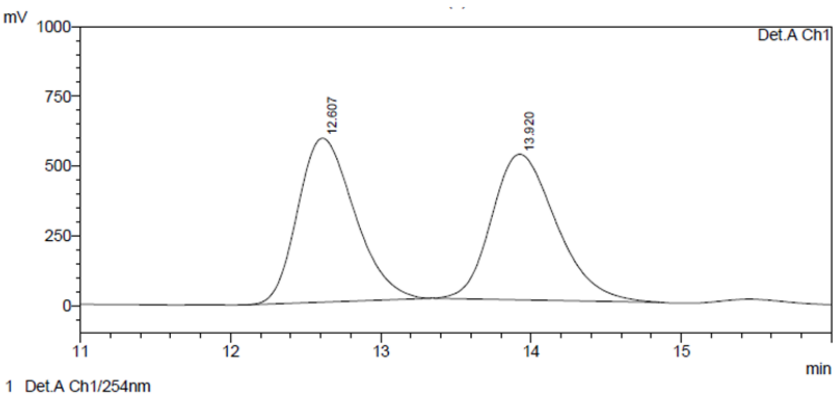

PeakTable

Detector A Ch1 254nm

Peak#	Ret. Time	Area	Height	Area %	Height %
1	12.607	15467900	587727	49.740	52.965
2	13.920	15629613	521932	50.260	47.035
Total		31097513	1109659	100.000	100.000

Racemic **5k**

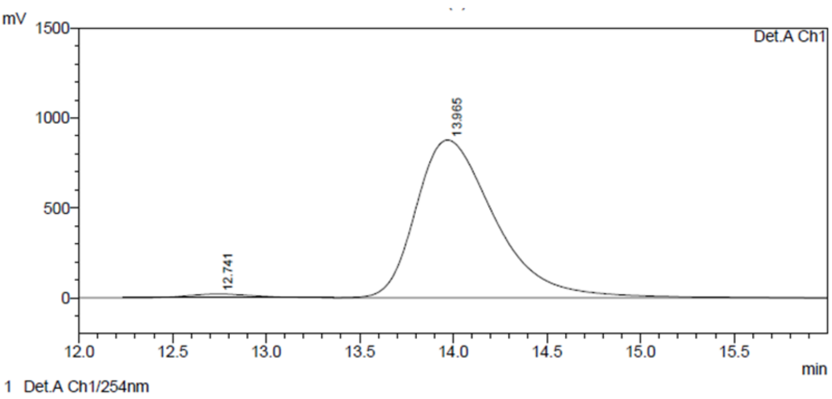

PeakTable

Detector A Ch1 254nm

Peak#	Ret. Time	Area	Height	Area %	Height %
1	12.741	523596	20907	1.985	2.330
2	13.965	25851855	876409	98.015	97.670
Total		26375451	897316	100.000	100.000

Enantioenriched **5k**

tert-Butyl (2*R*,2'*S*)-3-oxo-2'-(thiophen-2-yl)-3*H*-spiro[benzofuran-2,1'-cyclopentan]-3'-ene-3'-carboxylate **5l**

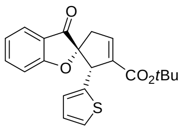

A yellow solid; $[\alpha]^{25}_{D}$ = 181.9 (c 1, $CHCl_3$); ^1H NMR (500 MHz, $CDCl_3$) δ 7.66–7.59 (m, 1H), 7.54–7.47 (m, 1H), 7.11 (d, *J* = 5.1 Hz, 1H), 7.02 (t, *J* = 7.5 Hz, 1H), 6.90–6.88 (m, 1H), 6.87 (d, *J* = 8.5 Hz, 1H), 6.80 (dd, *J* = 5.0 Hz, 3.6 Hz, 1H), 6.72 (d, *J* = 3.4 Hz, 1H), 4.84 (d, *J* = 1.7 Hz, 1H), 3.13 (dt, *J* = 18.8 Hz, 1.9 Hz, 1H), 2.96–2.84 (m, 1H), 1.29 (s, 9H); ^{13}C NMR (125 MHz, $CDCl_3$) δ 202.11, 197.73, 171.47, 162.81, 139.05, 138.41, 138.29, 137.95, 126.79, 125.92, 124.84, 124.28, 121.99, 120.58, 113.14, 94.85, 80.96, 54.29, 41.39, 27.78; HRMS (ESI) m/z calcd for $C_{21}H_{20}NaO_4S$ $[M + Na]^+$ = 391.0975, found = 391.0982; The ee value was 95%, t_R (major) = 9.974 min, t_R (minor) = 26.548 min (Chiralpak IC, λ = 254 nm, 5% *i*-PrOH/hexane, flow rate = 1.0 mL/min).

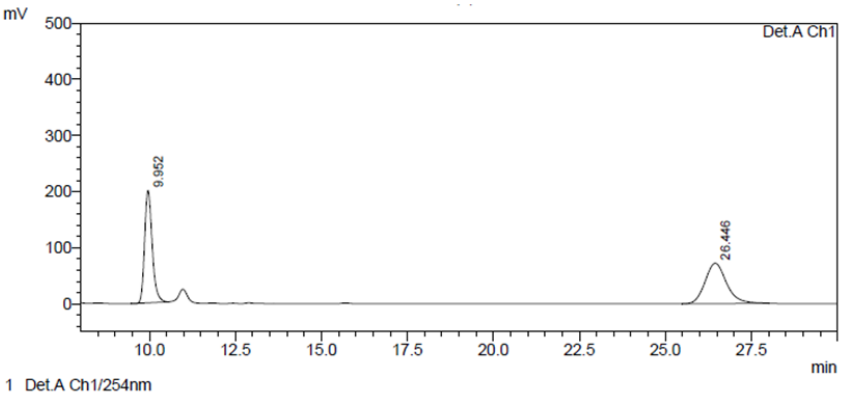

PeakTable

Detector A Ch1 254nm

Peak#	Ret. Time	Area	Height	Area %	Height %
1	9.952	3078660	200318	49.657	73.574
2	26.446	3121217	71951	50.343	26.426
Total		6199877	272269	100.000	100.000

Racemic **5l**

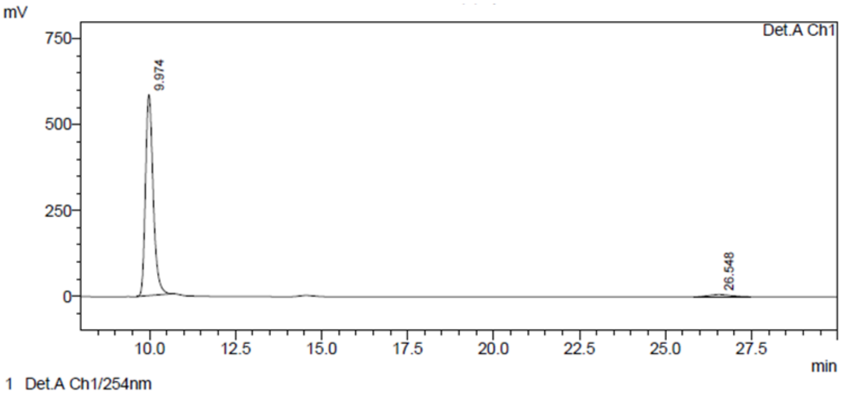

PeakTable

Detector A Ch1 254nm

Peak#	Ret. Time	Area	Height	Area %	Height %
1	9.974	9155134	585304	97.400	98.988
2	26.548	244341	5982	2.600	1.012
Total		9399475	591286	100.000	100.000

Enantioenriched **5l**

<u>*tert*-Butyl (2*R*,2'*S*)-2'-cyclohexyl-3-oxo-3*H*-spiro[benzofuran-2,1'-cyclopentan]-3'-ene-3'-carboxylate **5m**</u>

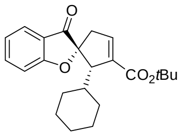

Colorless oil; $[\alpha]^{25}{}_{D}$ = 52.3 (c 1, $CHCl_3$); ^{1}H NMR (500 MHz, $CDCl_3$) δ 7.70 (d, *J* = 7.7 Hz, 1H), 7.68–7.62 (m, 1H), 7.11 (dd, *J* = 7.9 Hz, 5.1 Hz, 2H), 6.67 (q, *J* = 2.4 Hz, 1H), 3.33 (d, *J* = 1.6 Hz, 1H), 2.83 (dd, *J* = 18.6 Hz, 1.4 Hz, 1H), 2.78–2.70 (m, 1H), 1.99–1.90 (m, 1H), 1.75–1.54 (m, 6H), 1.53 (s, 9H), 1.21–1.09 (m, 2H), 1.06–0.96 (m, 1H), 0.88 (qd, *J* = 12.4 Hz, 3.3 Hz, 1H); ^{13}C NMR (125 MHz, $CDCl_3$) δ 203.42, 171.37, 164.09, 138.79, 138.13, 137.85, 124.82,

122.02, 120.05, 113.14, 96.41, 80.78, 56.05, 43.00, 38.62, 30.47, 30.41, 28.14, 27.20, 27.09, 26.53; HRMS (ESI) m/z calcd for $C_{23}H_{28}NaO_4$ $[M + Na]^+$ = 391.1880, found = 391.1885; The ee value was 95%, t_R (major) = 12.231 min, t_R (minor) = 13.820 min (Chiralpak IC, λ = 254 nm, 1% *i*-PrOH/hexane, flow rate = 1.0 mL/min).

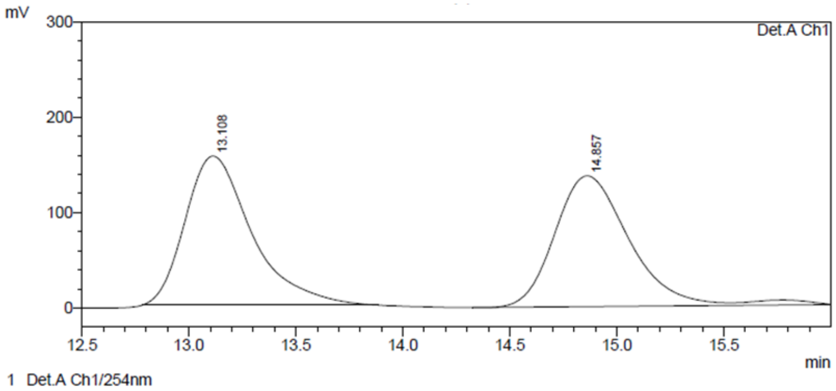

PeakTable

Detector A Ch1 254nm

Peak#	Ret. Time	Area	Height	Area %	Height %
1	13.108	3384433	155961	50.048	53.236
2	14.857	3377992	136998	49.952	46.764
Total		6762425	292959	100.000	100.000

Racemic **5m**

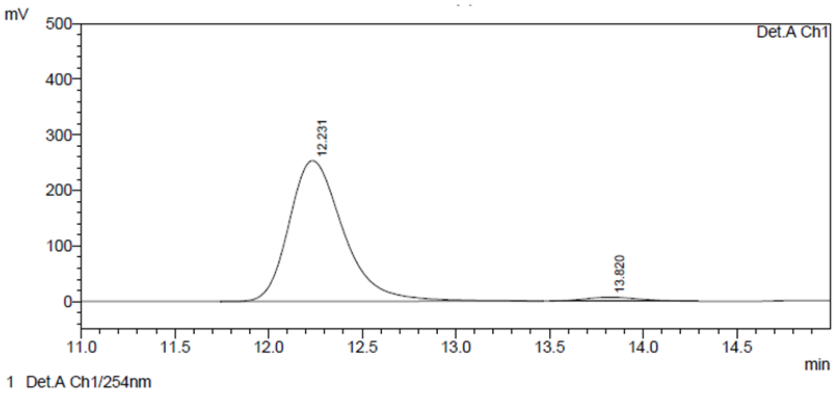

PeakTable

Detector A Ch1 254nm

Peak#	Ret. Time	Area	Height	Area %	Height %
1	12.231	5110587	253167	97.354	97.330
2	13.820	138882	6946	2.646	2.670
Total		5249469	260113	100.000	100.000

Enantioenriched **5m**

<u>*tert*-Butyl (2*R*,2'*S*)-2'-isopropyl-3-oxo-3*H*-spiro[benzofuran-2,1'-cyclopentan]-3'-ene-3'-carboxylate **5n**</u>

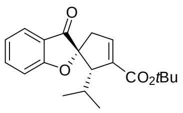

A white solid; $[\alpha]^{25}_{D}$ = 31.7 (c 1, $CHCl_3$); ^1H NMR (500 MHz, $CDCl_3$) δ 7.71 (dd, *J* = 7.9 Hz, 1.1 Hz, 1H), 7.67–7.61 (m, 1H), 7.11 (dd, *J* = 8.2 Hz, 6.2 Hz, 2H), 6.70 (q, *J* = 2.4 Hz, 1H), 3.37 (dd, *J* = 3.5 Hz, 1.8 Hz, 1H), 2.88–2.80 (m, 1H), 2.79–2.70 (m, 1H), 2.36 (dtd, *J* = 14.2 Hz, 7.1 Hz, 4.0 Hz, 1H), 1.52 (s, 9H), 1.05 (d, *J* = 7.1 Hz, 3H), 0.81 (d, *J* = 7.1 Hz, 3H); ^{13}C NMR (125 MHz, $CDCl_3$) δ 203.58, 171.40, 163.95, 138.88, 138.08, 124.80, 122.02, 119.96, 113.19, 96.55, 80.76, 56.47, 43.05, 28.13, 27.99, 19.96, 19.64; HRMS (ESI) m/z calcd for $C_{20}H_{24}NaO_4$ $[M + Na]^+$ = 351.1567, found = 351.1572; The ee value was 94%, t_R (major) = 6.941 min, t_R (minor) = 7.976 min (Chiralpak IC, λ = 254 nm, 5% *i*-PrOH/hexane, flow rate = 1.0 mL/min).

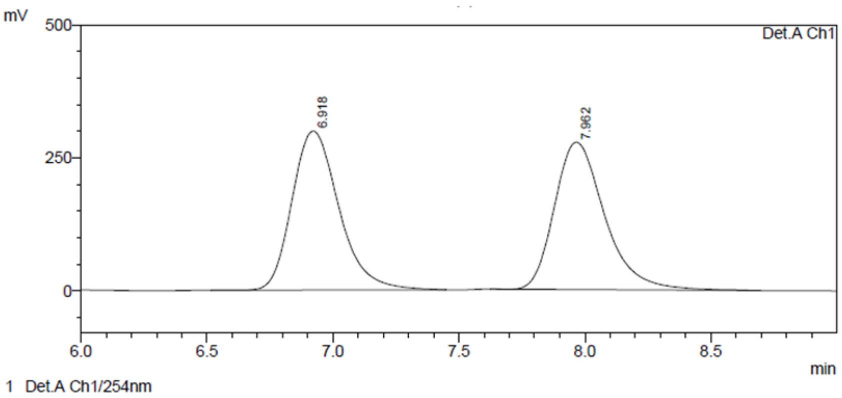

PeakTable

Detector A Ch1 254nm

Peak#	Ret. Time	Area	Height	Area %	Height %
1	6.918	3875108	298854	50.197	51.872
2	7.962	3844616	277280	49.803	48.128
Total		7719724	576134	100.000	100.000

Racemic **5n**

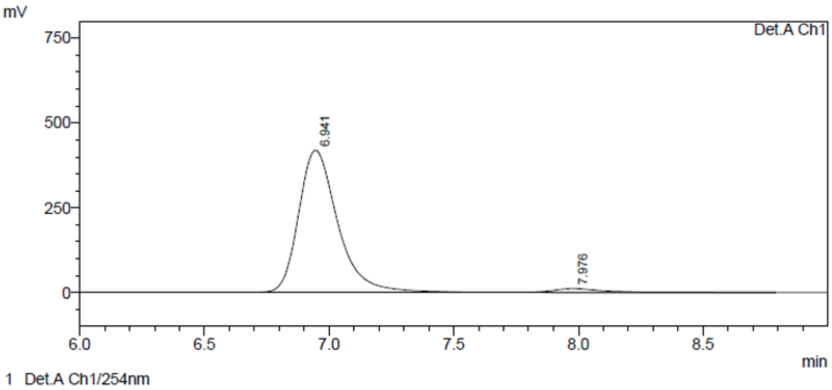

PeakTable

Detector A Ch1 254nm

Peak#	Ret. Time	Area	Height	Area %	Height %
1	6.941	4572453	418292	96.846	97.350
2	7.976	148889	11385	3.154	2.650
Total		4721342	429677	100.000	100.000

Enantioenriched **5n**

<u>*tert*-Butyl (2*R*,2'*S*)-2'-butyl-3-oxo-3*H*-spiro[benzofuran-2,1'-cyclopentan]-3'-ene-3'-carboxylate **5o**</u>

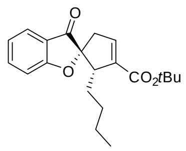

Colorless oil; $[\alpha]^{25}_{D}$ = -21 (c 1, $CHCl_3$); ^1H NMR (500 MHz, $CDCl_3$) δ 7.70 (dd, *J* = 7.9 Hz, 1.1 Hz, 1H), 7.64 (ddd, *J* = 8.5 Hz, 7.3 Hz, 1.4 Hz, 1H), 7.16–7.05 (m, 2H), 6.66 (q, *J* = 2.5 Hz, 1H), 3.43–3.33 (m, 1H), 2.94 (ddd, *J* = 18.9 Hz, 2.3 Hz, 1.6 Hz, 1H), 2.80–2.70 (m, 1H), 1.84–1.76 (m, 1H), 1.71 (ddd, *J* = 18.6 Hz, 9.5Hz, 4.7 Hz, 1H), 1.52 (s, 9H), 1.26–1.07 (m, 4H), 0.94–0.85 (m, 1H), 0.74 (t, *J* = 7.2 Hz, 3H); ^{13}C NMR (125 MHz, $CDCl_3$) δ 203.55, 171.79, 163.56, 139.12, 138.14, 137.45, 124.61, 121.99, 120.36, 113.10, 95.82, 80.76, 52.31, 43.18, 29.76, 28.16, 26.55, 22.68, 13.74; HRMS (ESI) m/z calcd for $C_{21}H_{26}NaO_4$ $[M + Na]^+$ = 365.1723, found = 365.1728; The ee value was 94%, t_R (major) = 19.418 min, t_R (minor) = 21.814 min (Chiralpak IC, λ = 254 nm, 0.5% *i*-PrOH/hexane, flow rate = 1.0 mL/min).

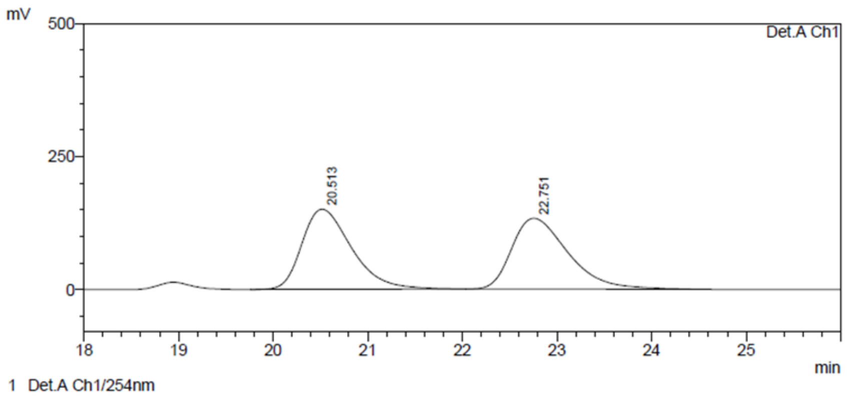

PeakTable

Detector A Ch1 254nm

Peak#	Ret. Time	Area	Height	Area %	Height %
1	20.513	5493933	150497	49.872	53.055
2	22.751	5522031	133165	50.128	46.945
Total		11015964	283662	100.000	100.000

Racemic **5o**

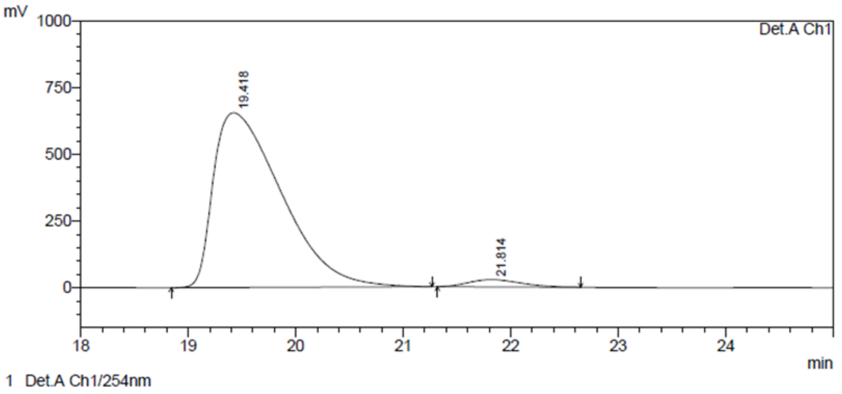

PeakTable

Detector A Ch1 254nm

Peak#	Ret. Time	Area	Height	Area %	Height %
1	19.418	28757375	653590	96.923	96.034
2	21.814	912940	26989	3.077	3.966
Total		29670315	680579	100.000	100.000

Enantioenriched **5o**

tert-Butyl (2*R*,2'*S*)-2'-ethyl-3-oxo-3*H*-spiro[benzofuran-2,1'-cyclopentan]-3'-ene-3'-carboxylate **5p**

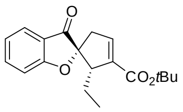

Colorless oil; $[\alpha]^{25}{}_{D}$ = -4.8 (c 1, $CHCl_3$); ^{1}H NMR (500 MHz, $CDCl_3$) δ 7.71 (d, *J* = 7.6 Hz, 1H), 7.65 (dd, *J* = 12.0 Hz, 4.7 Hz, 1H), 7.12 (dd, *J* = 11.9 Hz, 5.2 Hz, 2H), 6.68 (q, *J* = 2.3 Hz, 1H), 3.39–3.29 (m, 1H), 2.94 (dd, *J* = 18.9 Hz, 1.5 Hz, 1H), 2.76 (dd, *J* = 18.9 Hz, 1.8 Hz, 1H), 1.86 (dqd, *J* = 15.0 Hz, 7.5 Hz, 3.2 Hz, 1H), 1.81–1.70 (m, 1H), 1.66 (t, *J* = 5.0 Hz, 1H), 0.71 (t, *J* = 7.4 Hz, 3H); ^{13}C NMR (125 MHz, $CDCl_3$) δ 203.57, 171.81, 163.56, 138.84, 138.15, 137.72, 124.65, 122.01, 120.29, 113.16, 95.82, 80.77, 53.63, 43.21, 28.15, 20.13, 12.14; HRMS (ESI) m/z calcd for $C_{19}H_{22}NaO_4$ $[M + Na]^+$ = 337.1410, found = 337.1413; The ee value was 96%, t_R (major) = 14.552 min, t_R (minor) = 18.752 min (Chiralpak IC, λ = 254 nm, 1% *i*-PrOH/hexane, flow rate = 1.0 mL/min).

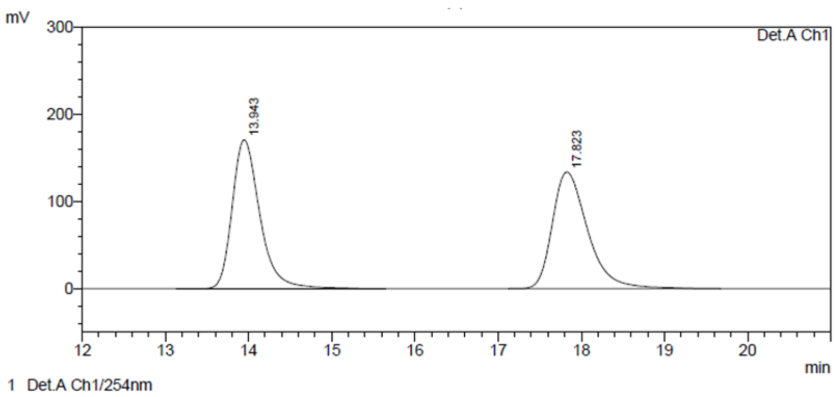

PeakTable

Detector A Ch1 254nm

Peak#	Ret. Time	Area	Height	Area %	Height %
1	13.943	3946691	170455	50.012	56.070
2	17.823	3944803	133549	49.988	43.930
Total		7891494	304004	100.000	100.000

Racemic **5p**

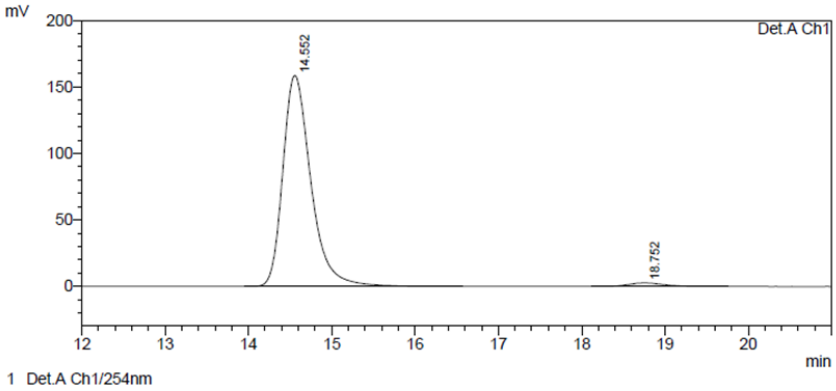

PeakTable

Detector A Ch1 254nm

Peak#	Ret. Time	Area	Height	Area %	Height %
1	14.552	3723774	158713	97.857	98.294
2	18.752	81546	2755	2.143	1.706
Total		3805320	161468	100.000	100.000

Enantioenriched **5p**

<u>*tert*-Butyl (2*R*,5'*S*)-3-oxo-5'-phenyl-3*H*-spiro[benzofuran-2,1'-cyclopentan]-2'-ene-2'-carboxylate **6a**</u>

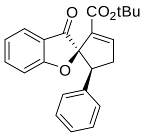

A white solid; $[\alpha]^{25}{}_{D}$ = -132.3 (c 1, $CHCl_3$); ^1H NMR (500 MHz, $CDCl_3$) δ 7.57 (dd, *J* = 7.7 Hz, 0.8 Hz, 1H), 7.44 (t, *J* = 2.4 Hz, 1H), 7.40 (ddd, *J* = 8.5 Hz, 7.3 HZ, 1.4 Hz, 1H), 7.21–7.11 (m, 5H), 6.92 (t, *J* = 7.5 Hz, 1H), 6.78 (d, *J* = 8.3 Hz, 1H), 3.99 (t, *J* = 8.0 Hz, 1H), 3.08 (qdd, *J* = 18.8 Hz, 8.1 Hz, 2.5 Hz, 2H), 1.17 (s, 9H); ^{13}C NMR (125 MHz, $CDCl_3$) δ 201.97, 170.92, 161.42, 151.65, 137.56, 136.05, 135.34, 129.03, 127.88, 127.34, 123.85, 122.12, 121.36, 112.50,

96.87, 81.58, 54.23, 37.96, 27.61; HRMS (ESI) m/z calcd for $C_{23}H_{22}NaO_4$ $[M + Na]^+$ = 385.1410, found = 385.1415; The ee value was 98%, t_R (minor) = 8.364 min, t_R (major) = 25.197 min (Chiralpak IC, λ = 254 nm, 10% *i*-PrOH/hexane, flow rate = 1.0 mL/min).

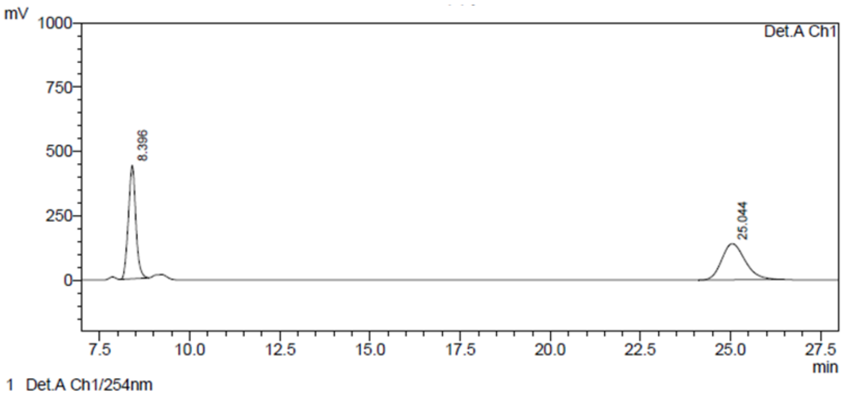

PeakTable

Detector A Ch1 254nm

Peak#	Ret. Time	Area	Height	Area %	Height %
1	8.396	6353479	440724	50.120	75.843
2	25.044	6322973	140376	49.880	24.157
Total		12676453	581100	100.000	100.000

Racemic **6a**

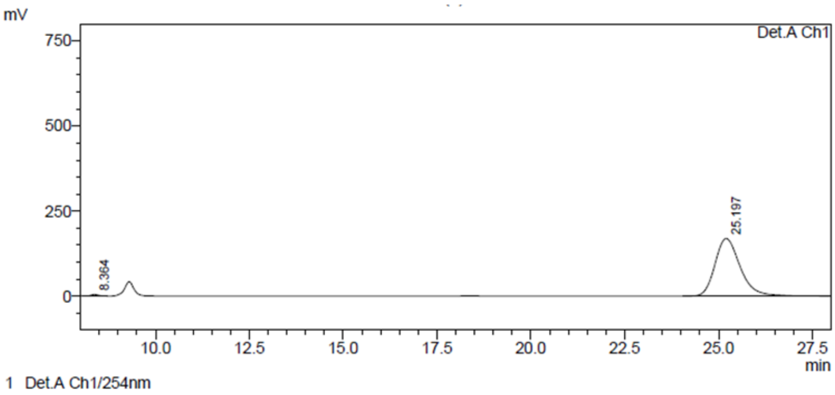

PeakTable

Detector A Ch1 254nm

Peak#	Ret. Time	Area	Height	Area %	Height %
1	8.364	59646	4420	0.743	2.541
2	25.197	7967113	169533	99.257	97.459
Total		8026759	173953	100.000	100.000

Enantioenriched **5q**

<u>*tert*-Butyl (2*R*,5'*S*)-5'-(4-chlorophenyl)-3-oxo-3*H*-spiro[benzofuran-2,1'-cyclopentan]-2'-ene-2'-carboxylate **6b**</u>

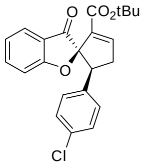

A white solid; $[\alpha]^{25}{}_D$ = -177.0 (c 0.5, $CHCl_3$); ^1H NMR (500 MHz, $CDCl_3$) δ 7.58 (d, *J* = 7.6 Hz, 1H), 7.48–7.43 (m, 1H), 7.42 (t, *J* = 2.4 Hz, 1H), 7.15–7.10 (m, 4H), 6.96 (t, *J* = 7.4 Hz, 1H), 6.81 (d, *J* = 8.3 Hz, 1H), 3.95 (t, *J* = 8.1 Hz, 1H), 3.11–2.98 (m, 2H), 1.16 (s, 9H); ^{13}C NMR (125 MHz, $CDCl_3$) δ 201.70, 170.83, 161.29, 151.38, 137.85, 136.07, 133.95, 133.18, 130.39, 128.10, 123.92, 121.95, 121.63, 112.57, 96.59, 81.72, 53.48, 38.16, 27.60; HRMS (ESI) m/z calcd for $C_{23}H_{21}ClNaO_4$ $[M + Na]^+$ = 419.1021, found = 419.1029; The ee value was 98%, t_R (minor) = 8.250 min, t_R (major) = 31.016 min (Chiralpak IC, λ = 254 nm, 10% *i*-PrOH/hexane, flow rate = 1.0 mL/min).

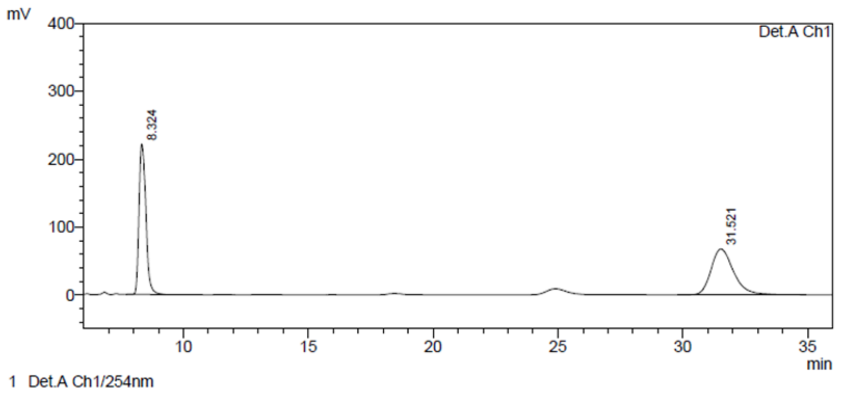

PeakTable

Detector A Ch1 254nm

Peak#	Ret. Time	Area	Height	Area %	Height %
1	8.324	4234156	221438	49.860	76.597
2	31.521	4257968	67657	50.140	23.403
Total		8492125	289096	100.000	100.000

Racemic **6b**

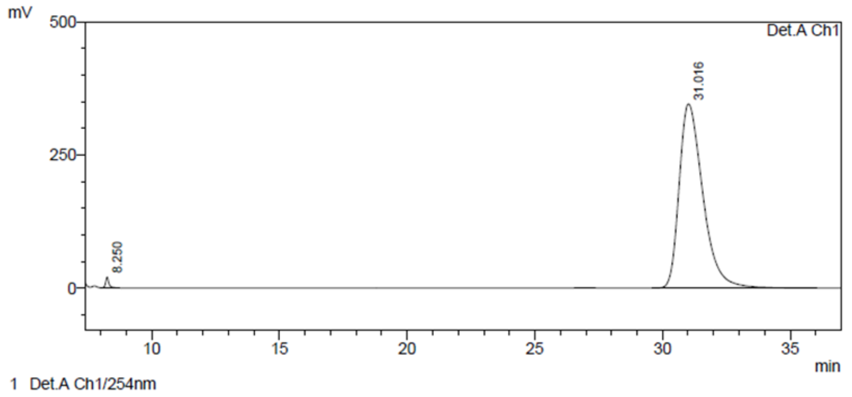

PeakTable

Detector A Ch1 254nm

Peak#	Ret. Time	Area	Height	Area %	Height %
1	8.250	175265	20304	0.779	5.547
2	31.016	22311680	345727	99.221	94.453
Total		22486945	366031	100.000	100.000

Enantioenriched **6b**

tert-Butyl (2*R*,5'*S*)-5'-(3-chlorophenyl)-3-oxo-3*H*-spiro[benzofuran-2,1'-cyclopentan]-2'-ene-2'-carboxylate **6c**

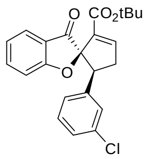

A white solid; $[\alpha]^{25}_{D}$ = -151.9 (c 1, $CHCl_3$); ^1H NMR (500 MHz, $CDCl_3$) δ 7.60 (d, *J* = 7.6 Hz, 1H), 7.48–7.43 (m, 1H), 7.42 (t, *J* = 2.4 Hz, 1H), 7.24 (s, 1H), 7.13–7.03 (m, 3H), 6.96 (t, *J* = 7.5 Hz, 1H), 6.84 (d, *J* = 8.3 Hz, 1H), 3.95 (t, *J* = 8.0 Hz, 1H), 3.14–3.00 (m, 2H), 1.18 (s, 9H); ^{13}C NMR (125 MHz, $CDCl_3$) δ 201.55, 170.84, 161.24, 151.22, 137.79, 137.53, 136.07, 133.78, 129.15, 129.10, 127.56, 127.32, 123.97, 121.99, 121.61, 112.55, 96.54, 81.72, 53.66, 38.06, 27.61; HRMS (ESI) m/z calcd for $C_{27}H_{24}NaO_4$ $[M + Na]^+$ = 435.1567, found = 435.1568; The ee value was 98%, t_R (minor) = 7.919 min, t_R (minor) = 25.253 min (Chiralpak IC, λ = 254 nm, 10% *i*-PrOH/hexane, flow rate = 1.0 mL/min).

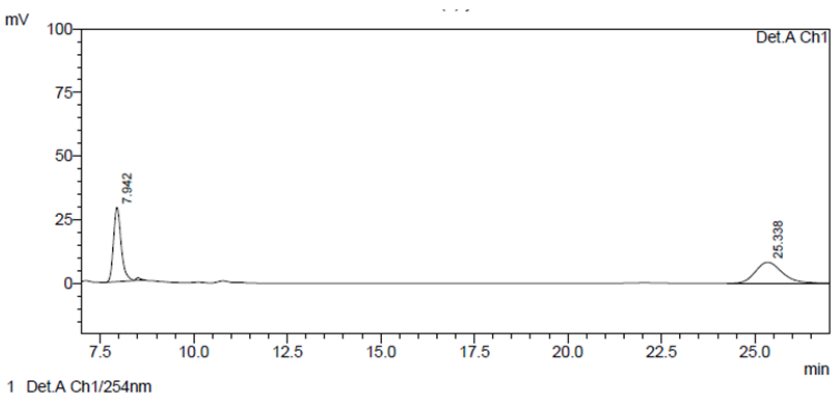

PeakTable

Detector A Ch1 254nm

Peak#	Ret. Time	Area	Height	Area %	Height %
1	7.942	424108	29191	50.121	77.824
2	25.338	422056	8318	49.879	22.176
Total		846165	37509	100.000	100.000

Racemic **6c**

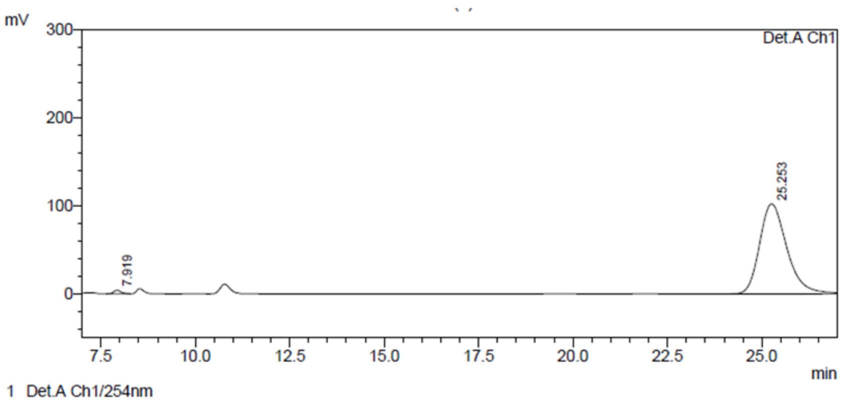

PeakTable

Detector A Ch1 254nm

Peak#	Ret. Time	Area	Height	Area %	Height %
1	7.919	53508	4246	1.024	3.993
2	25.253	5172696	102091	98.976	96.007
Total		5226204	106337	100.000	100.000

Enantioenriched **6c**

tert-Butyl (2*R*,5'*S*)-3-oxo-5'-(o-tolyl)-3*H*-spiro[benzofuran-2,1'-cyclopentan]-2'-ene-2'-carboxylate **6d**

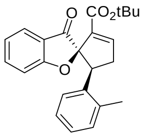

A white solid; $[\alpha]^{25}_{D}$ = -221.9 (c 1, $CHCl_3$); ^1H NMR (500 MHz, $CDCl_3$) δ 7.58 (d, *J* = 7.6 Hz, 1H), 7.45 (t, *J* = 2.4 Hz, 1H), 7.44–7.38 (m, 2H), 7.13 (t, *J* = 7.4 Hz, 1H), 7.01 (t, *J* = 7.4 Hz, 1H), 6.93 (t, *J* = 7.4 Hz, 2H), 6.81 (d, *J* = 8.4 Hz, 1H), 4.35 (t, *J* = 7.8 Hz, 1H), 3.06 (dddd, *J* = 19.1 Hz, 9.9 Hz, 7.8 Hz, 2.5 Hz, 2H), 2.16 (s, 3H), 1.17 (s, 9H); ^{13}C NMR (125 MHz, $CDCl_3$) δ 202.44, 171.02, 161.47, 151.74, 137.66, 136.64, 136.01, 134.14, 129.94, 129.72, 126.93, 125.38, 123.84, 121.80, 121.44, 112.59, 96.72, 81.55, 48.56, 39.54, 27.61, 19.88; HRMS (ESI) m/z calcd for $C_{24}H_{24}NaO_4$ $[M + Na]^+$ = 399.1567, found = 399.1559; The ee value was 99%, t_R (minor) = 9.952 min, t_R (major) = 38.990 min (Chiralpak IC, λ = 254 nm, 5% *i*-PrOH/hexane, flow rate = 1.0 mL/min).

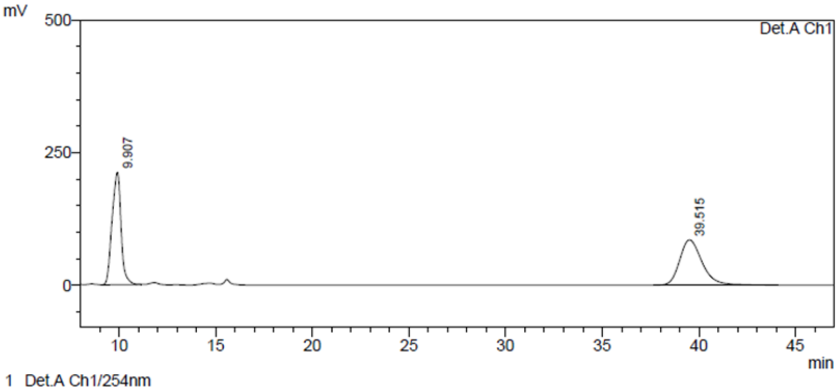

PeakTable

Detector A Ch1 254nm

Peak#	Ret. Time	Area	Height	Area %	Height %
1	9.907	6875226	212132	49.943	71.331
2	39.515	6890854	85259	50.057	28.669
Total		13766081	297391	100.000	100.000

Racemic **6d**

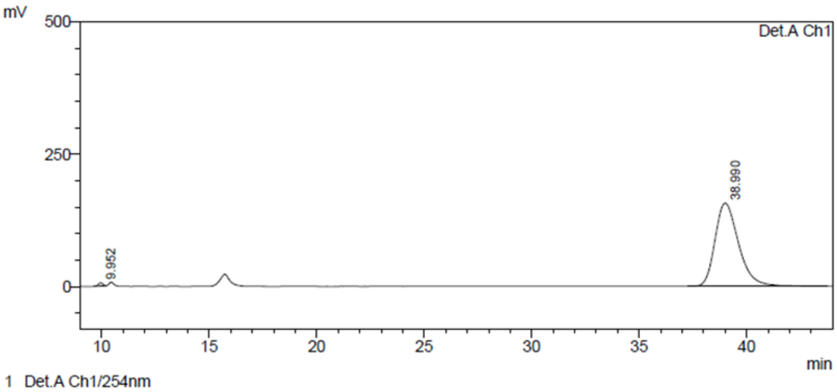

PeakTable

Detector A Ch1 254nm

Peak#	Ret. Time	Area	Height	Area %	Height %
1	9.952	85435	6078	0.703	3.721
2	38.990	12066715	157251	99.297	96.279
Total		12152150	163329	100.000	100.000

Enantioenriched **6d**

tert-Butyl (2*R*,5'*S*)-5'-(4-bromophenyl)-3-oxo-3*H*-spiro[benzofuran-2,1'-cyclopentan]-2'-ene-2'-carboxylate **6e**

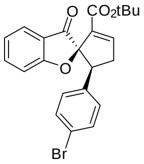

A white solid; $[\alpha]^{25}_{D}$ = -167.7 (c 1, $CHCl_3$); ^1H NMR (500 MHz, $CDCl_3$) δ 7.58 (d, *J* = 7.6 Hz, 1H), 7.49–7.43 (m, 1H), 7.41 (t, *J* = 2.4 Hz, 1H), 7.29 (d, *J* = 8.3 Hz, 3H), 7.07 (d, *J* = 8.4 Hz, 2H), 6.96 (t, *J* = 7.4 Hz, 1H), 6.82 (d, *J* = 8.2 Hz, 1H), 3.94 (t, *J* = 8.0 Hz, 1H), 3.11–2.97 (m, 2H), 1.17 (s, 9H); ^{13}C NMR (125 MHz, $CDCl_3$) δ 201.65, 170.84, 161.27,

151.31, 137.85, 136.11, 134.51, 131.06, 130.74, 123.93, 121.94, 121.64, 121.36, 112.59, 96.53, 81.72, 53.51, 38.14, 27.60; HRMS (ESI) m/z calcd for $C_{23}H_{21}BrNaO_4$ $[M + Na]^+$ = 463.0515, found = 463.0520; The ee value was 98%, t_R (minor) = 6.628 min, t_R (major) = 21.869 min (Chiralpak IC, λ = 254 nm, 20% *i*-PrOH/hexane, flow rate = 1.0 mL/min).

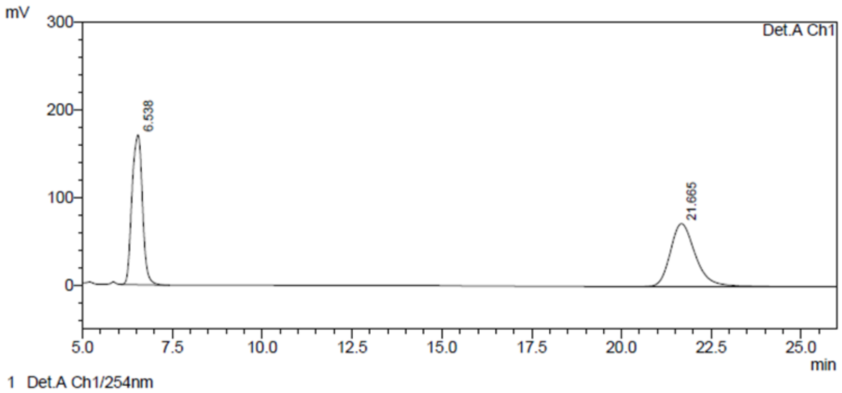

PeakTable

Detector A Ch1 254nm

Peak#	Ret. Time	Area	Height	Area %	Height %
1	6.538	3456680	170351	50.048	70.449
2	21.665	3450039	71458	49.952	29.551
Total		6906720	241809	100.000	100.000

Racemic **6e**

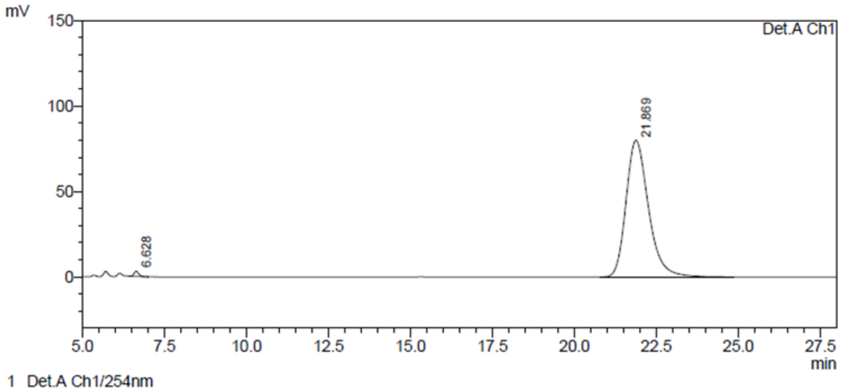

PeakTable

Detector A Ch1 254nm

Peak#	Ret. Time	Area	Height	Area %	Height %
1	6.628	31796	2988	0.825	3.602
2	21.869	3821560	79952	99.175	96.398
Total		3853356	82940	100.000	100.000

Enantioenriched **6e**

tert-Butyl (2*R*,5'*S*)-5'-(4-methoxyphenyl)-3-oxo-3*H*-spiro[benzofuran-2,1'-cyclopentan]-2'-ene-2'-carboxylate **6f**

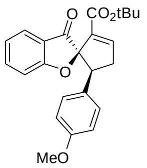

A white solid; $[\alpha]^{25}_{D}$ = -161.1 (c 1, $CHCl_3$); ^{1}H NMR (500 MHz, $CDCl_3$) δ 7.56 (dd, *J* = 7.7 Hz, 0.8 Hz, 1H), 7.41 (ddd, *J* = 9.9 Hz, 6.0 Hz, 1.4 Hz, 2H), 7.12–7.08 (m, 2H), 6.92 (dd, *J* = 11.4 Hz, 4.1 Hz, 1H), 6.81 (d, *J* = 8.3 Hz, 1H), 6.71–6.66 (m, 2H), 3.94 (t, *J* = 8.1 Hz, 1H), 3.69 (s, 3H), 3.02 (dd, *J* = 8.1 Hz, 2.5 Hz, 2H), 1.16 (s, 9H); ^{13}C NMR (125 MHz, $CDCl_3$) δ 202.17, 170.96, 161.48, 158.73, 151.79, 137.62, 136.04, 130.09, 127.37, 123.83, 122.10, 121.35, 113.29, 112.58, 96.90, 81.56, 55.10, 53.63, 38.33, 27.60; HRMS (ESI) m/z calcd for $C_{24}H_{24}NaO_5$ $[M + Na]^+$ = 415.1516, found = 415.1521; The ee value was 99%, t_R (minor) = 19.056 min, t_R (major) = 101.184 min (Chiralpak IC, λ = 254 nm, 5% *i*-PrOH/hexane, flow rate = 1.0 mL/min).

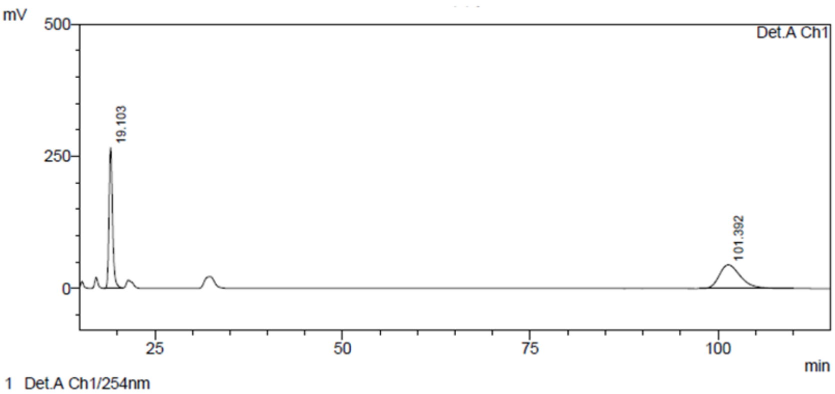

PeakTable

Detector A Ch1 254nm

Peak#	Ret. Time	Area	Height	Area %	Height %
1	19.103	8757516	265749	49.721	85.547
2	101.392	8855933	44898	50.279	14.453
Total		17613449	310647	100.000	100.000

Racemic **6f**

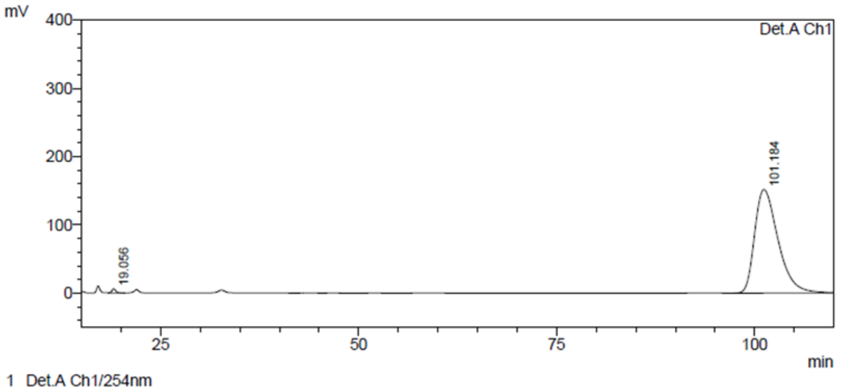

PeakTable

Detector A Ch1 254nm

Peak#	Ret. Time	Area	Height	Area %	Height %
1	19.056	203294	6271	0.648	3.969
2	101.184	31147041	151734	99.352	96.031
Total		31350335	158005	100.000	100.000

Enantioenriched **6f**

<u>*tert*-Butyl (2*R*,5'*S*)-3-oxo-5'-(p-tolyl)-3*H*-spiro[benzofuran-2,1'-cyclopentan]-2'-ene-2'-carboxylate **6g**</u>

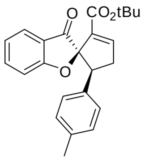

A white solid; $[\alpha]^{25}{}_{D}$ = -160.6 (c 1, $CHCl_3$); ^{1}H NMR (500 MHz, $CDCl_3$) δ 7.60–7.55 (m, 1H), 7.45–7.39 (m, 2H), 7.07 (d, *J* = 8.0 Hz, 2H), 6.96 (d, *J* = 8.0 Hz, 2H), 6.93 (t, *J* = 7.5 Hz, 1H), 6.81 (d, *J* = 8.2 Hz, 1H), 3.96 (t, *J* = 8.0 Hz, 1H), 3.11–2.97 (m, 2H), 2.21 (s, 3H), 1.17 (s, 9H); ^{13}C NMR (125 MHz, $CDCl_3$) δ 202.09, 171.00, 161.47, 151.74, 137.54, 136.90, 136.09, 132.29, 128.89, 128.61, 123.85, 122.12, 121.32, 112.58, 96.91, 81.53, 53.90, 38.20, 27.61, 20.99; HRMS (ESI) m/z calcd for $C_{24}H_{24}NaO_4$ $[M + Na]^+$ = 399.1567, found = 399.1572; The ee value was 99%, t_R (minor) = 9.199 min, t_R (major) = 37.032 min (Chiralpak IC, λ = 254 nm, 10% *i*-PrOH/hexane, flow rate = 1.0 mL/min).

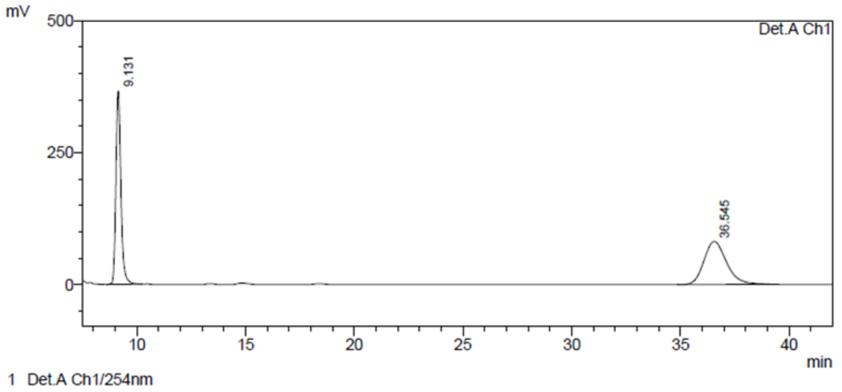

PeakTable

Detector A Ch1 254nm

Peak#	Ret. Time	Area	Height	Area %	Height %
1	9.131	5868080	366667	50.091	81.851
2	36.545	5846709	81301	49.909	18.149
Total		11714789	447968	100.000	100.000

Racemic **6g**

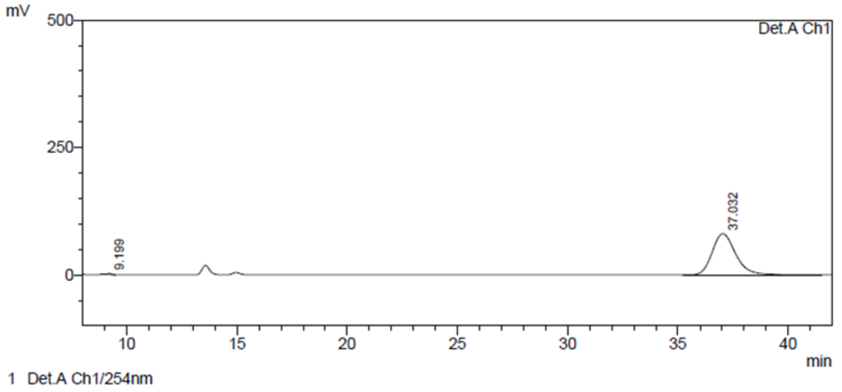

PeakTable

Detector A Ch1 254nm

Peak#	Ret. Time	Area	Height	Area %	Height %
1	9.199	25278	2023	0.416	2.436
2	37.032	6046185	81033	99.584	97.564
Total		6071463	83057	100.000	100.000

Enantioenriched **6g**

tert-Butyl (2*R*,5'*S*)-3-oxo-5'-(m-tolyl)-3*H*-spiro[benzofuran-2,1'-cyclopentan]-2'-ene-2'-carboxylate **6h**

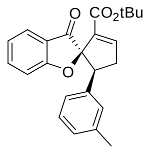

A white solid; $[\alpha]^{25}_{D}$ = -129.0 (c 1, $CHCl_3$); ^1H NMR (500 MHz, $CDCl_3$) δ 7.58 (d, *J* = 7.4 Hz, 1H), 7.42 (dd, *J* = 8.3 Hz, 7.3 Hz, 2H), 7.04 (t, *J* = 7.7 Hz, 1H), 6.99 (d, *J* = 2.9 Hz, 2H), 6.93 (t, *J* = 7.2 Hz, 2H), 6.79 (d, *J* = 8.3 Hz, 1H), 3.95 (t, *J* = 8.0 Hz, 1H), 3.15–2.99 (m, 2H), 2.22 (s, 3H), 1.17 (s, 9H); ^{13}C NMR (125 MHz, $CDCl_3$) δ 202.07, 170.98, 161.45,

151.73, 137.56, 137.41, 136.04, 135.25, 129.79, 128.04, 127.74, 126.04, 123.85, 122.17, 121.33, 112.50, 96.91, 81.56, 54.17, 38.02, 27.62, 21.23; HRMS (ESI) m/z calcd for $C_{24}H_{24}NaO_4$ $[M + Na]^+$ = 399.1567, found = 399.1575; The ee value was 98%, t_R (minor) = 8.494 min, t_R (major) = 23.996 min (Chiralpak IC, λ = 254 nm, 10% *i*-PrOH/hexane, flow rate = 1.0 mL/min).

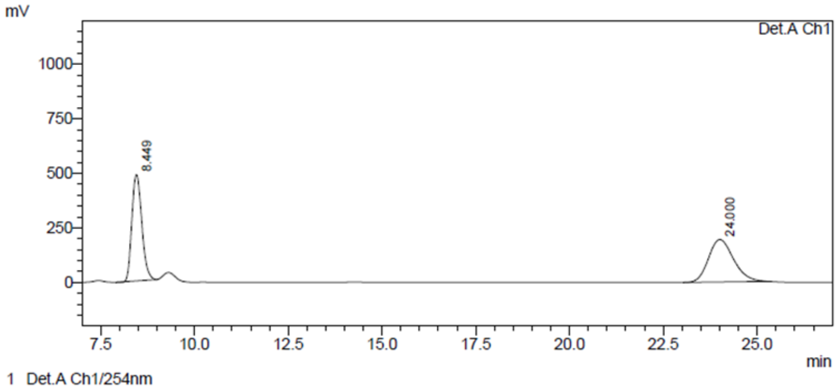

PeakTable

Detector A Ch1 254nm

Peak#	Ret. Time	Area	Height	Area %	Height %
1	8.449	8983306	486847	49.875	71.457
2	24.000	9028208	194464	50.125	28.543
Total		18011514	681311	100.000	100.000

Racemic **6h**

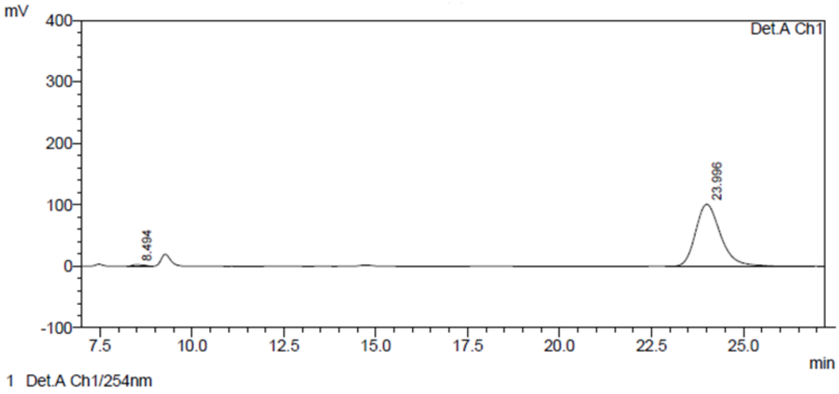

PeakTable

Detector A Ch1 254nm

Peak#	Ret. Time	Area	Height	Area %	Height %
1	8.494	50316	2447	1.041	2.370
2	23.996	4784908	100784	98.959	97.630
Total		4835224	103231	100.000	100.000

Enantioenriched **6h**

tert-Butyl (2*R*,5'*S*)-5'-(4-fluorophenyl)-3-oxo-3*H*-spiro[benzofuran-2,1'-cyclopentan]-2'-ene-2'-carboxylate **6i**

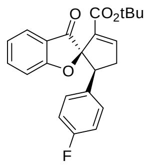

A white solid; $[\alpha]^{25}_{D}$ = -149.9 (c 1, $CHCl_3$); ^{1}H NMR (500 MHz, $CDCl_3$) δ 7.57 (dd, *J* = 7.7 Hz, 0.9 Hz, 1H), 7.43 (ddd, *J* = 9.0 Hz, 6.0 Hz, 1.9 Hz, 2H), 7.20–7.11 (m, 2H), 6.98–6.91 (m, 1H), 6.88–6.77 (m, 3H), 3.96 (t, *J* = 8.1 Hz, 1H), 3.11–2.97 (m, 2H), 1.17 (s, 9H); ^{13}C NMR (125 MHz, $CDCl_3$) δ 201.76, 170.82, 162.03 (d, *J* = 245.9 Hz), 161.32, 151.41, 137.72, 136.08, 131.09 (d, *J* = 3.2 Hz), 130.58 (d, *J* = 8.0 Hz), 123.87, 122.04, 121.52, 114.77 (d, *J* = 21.3 Hz), 112.51, 96.67, 81.66, 78.41, 53.48, 38.23, 27.61; HRMS (ESI) m/z calcd for $C_{23}H_{21}FNaO_4$ $[M + Na]^+$ = 403.1316, found = 403.1324; The ee value was 96%, t_R (minor) = 6.333 min, t_R (major) = 20.415 min (Chiralpak IC, λ = 254 nm, 20% *i*-PrOH/hexane, flow rate = 1.0 mL/min).

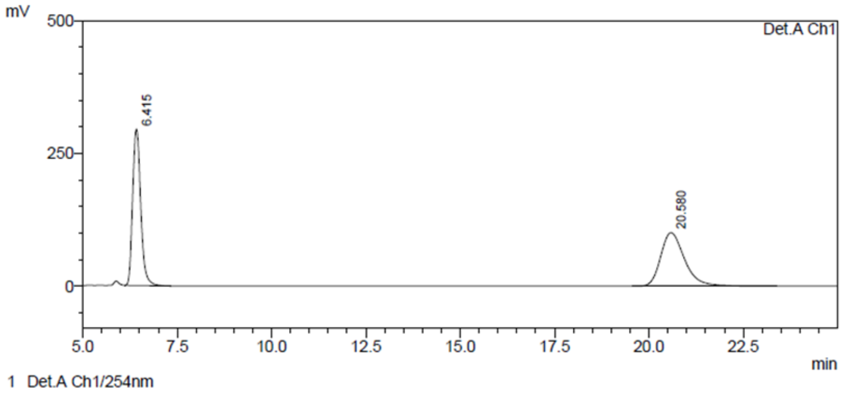

PeakTable

Detector A Ch1 254nm

Peak#	Ret. Time	Area	Height	Area %	Height %
1	6.415	4388530	294385	49.649	74.476
2	20.580	4450543	100887	50.351	25.524
Total		8839074	395272	100.000	100.000

Racemic **6i**

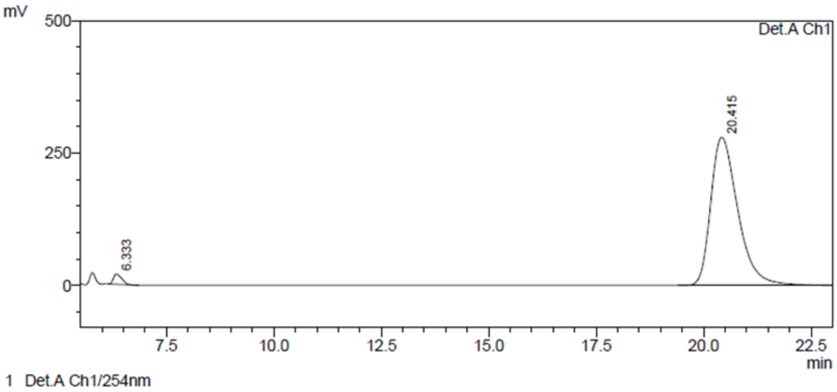

PeakTable

Detector A Ch1 254nm

Peak#	Ret. Time	Area	Height	Area %	Height %
1	6.333	261673	19739	2.087	6.587
2	20.415	12278554	279926	97.913	93.413
Total		12540227	299665	100.000	100.000

Enantioenriched **6i**

tert-Butyl (2*R*,5'*S*)-5'-(naphthalen-2-yl)-3-oxo-3*H*-spiro[benzofuran-2,1'-cyclopentan]-2'-ene-2'-carboxylate **6j**

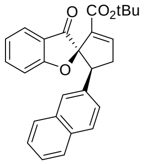

A white solid; $[\alpha]^{25}_{D}$ = -141.5 (c 1, $CHCl_3$); ^{1}H NMR (500 MHz, $CDCl_3$) δ 7.72 (dd, *J* = 9.0 Hz, 4.8 Hz, 2H), 7.68–7.62 (m, 2H), 7.60–7.54 (m, 1H), 7.48 (t, *J* = 2.4 Hz, 1H), 7.39 (ddd, *J* = 10.1 Hz, 8.1 Hz, 3.1 Hz, 3H), 7.35–7.30 (m, 1H), 6.87 (t, *J* = 7.4 Hz, 1H), 6.75 (d, *J* = 8.3 Hz, 1H), 4.18 (t, *J* = 8.0 Hz, 1H), 3.17 (dddd, *J* = 21.7 Hz, 18.8 Hz, 8.0 Hz, 2.5 Hz, 2H), 1.18 (s, 9H); ^{13}C NMR (125 MHz, $CDCl_3$) δ 202.06, 170.98, 161.44, 151.65, 137.66, 136.20, 133.17, 133.00, 132.63, 128.09, 127.77, 127.45, 127.01, 125.88, 125.77, 123.90, 121.98, 121.45, 112.53, 97.09, 81.64, 54.27, 38.38, 27.62; HRMS (ESI) m/z calcd for $C_{27}H_{24}NaO_4$ $[M + Na]^+$ = 435.1567, found = 435.1571; The ee value was 98%, t_R (minor) = 15.870 min, t_R (major) = 61.146 min (Chiralpak IC, λ = 254 nm, 5% *i*-PrOH/hexane, flow rate = 1.0 mL/min).

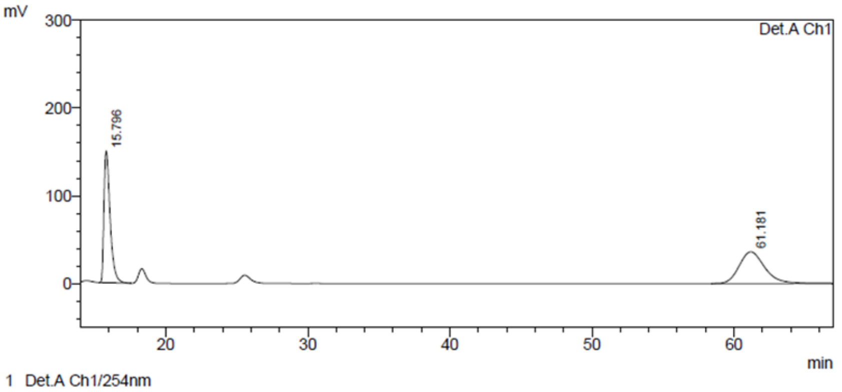

PeakTable

Detector A Ch1 254nm

Peak#	Ret. Time	Area	Height	Area %	Height %
1	15.796	4534624	150336	49.908	80.604
2	61.181	4551308	36176	50.092	19.396
Total		9085931	186512	100.000	100.000

Racemic **6j**

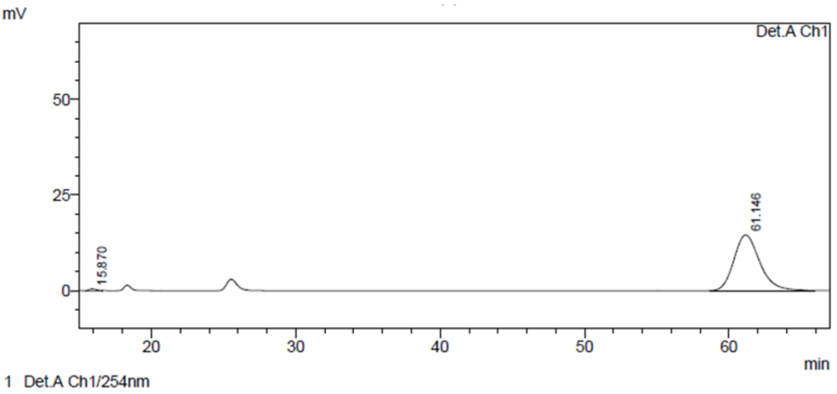

PeakTable

Detector A Ch1 254nm

Peak#	Ret. Time	Area	Height	Area %	Height %
1	15.870	15614	500	0.853	3.325
2	61.146	1813855	14551	99.147	96.675
Total		1829468	15052	100.000	100.000

Enantioenriched **6j**

<u>*tert*-Butyl (2*R*,5'*S*)-5'-(3,4-dimethoxyphenyl)-3-oxo-3*H*-spiro[benzofuran-2,1'-cyclopentan]-2'-ene-2'-carboxylate **6k**</u>

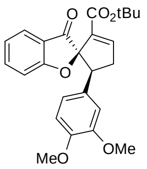

A white solid; $[\alpha]^{25}_{D}$ = -156.6 (c 1, $CHCl_3$); ^1H NMR (500 MHz, $CDCl_3$) δ 7.57 (d, *J* = 7.8 Hz, 1H), 7.42 (ddd, *J* = 6.0 Hz, 4.9 Hz, 1.4 Hz, 2H), 6.94 (t, *J* = 7.4 Hz, 1H), 6.80 (d, *J* = 8.3 Hz, 1H), 6.73 (dd, *J* = 8.2 Hz, 1.8 Hz, 1H), 6.68 (d, *J* =

1.7 Hz, 1H), 6.65 (d, *J* = 8.3 Hz, 1H), 3.94 (t, *J* = 8.1 Hz, 1H), 3.77 (s, 3H), 3.73 (s, 3H), 3.11–2.97 (m, 2H), 1.16 (s, 9H); ^{13}C NMR (125 MHz, $CDCl_3$) δ 202.14, 170.98, 161.42, 151.68, 148.30, 148.19, 137.72, 136.06, 127.84, 123.82, 122.17, 121.46, 121.23, 112.54, 112.11, 110.47, 96.96, 81.62, 55.81, 55.71, 53.94, 38.08, 27.60; HRMS (ESI) m/z calcd for $C_{25}H_{26}NaO_6$ $[M + Na]^+$ = 445.1622, found = 445.1630; The ee value was 99%, t_R (minor) = 16.631 min, t_R (major) = 41.715 min (Chiralpak IC, λ = 254 nm, 20% *i*-PrOH/hexane, flow rate = 1.0 mL/min).

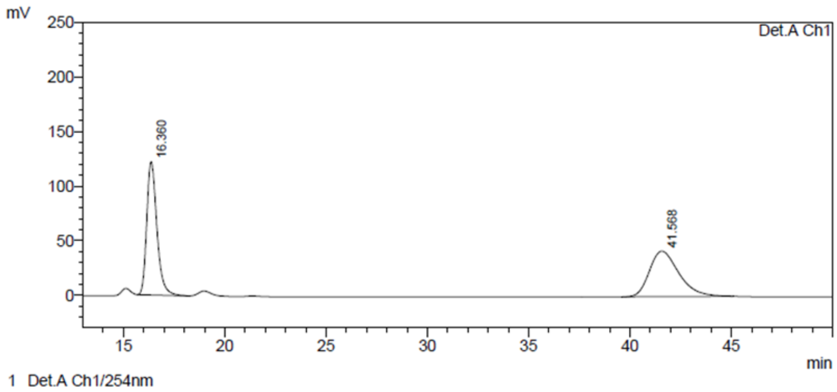

PeakTable

Detector A Ch1 254nm

Peak#	Ret. Time	Area	Height	Area %	Height %
1	16.360	4249277	122049	50.150	74.563
2	41.568	4223885	41637	49.850	25.437
Total		8473162	163687	100.000	100.000

Racemic **6k**

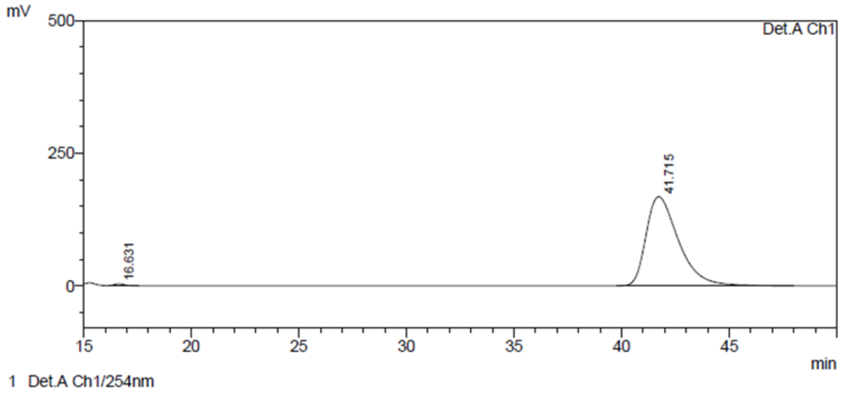

PeakTable

Detector A Ch1 254nm

Peak#	Ret. Time	Area	Height	Area %	Height %
1	16.631	112590	3525	0.633	2.055
2	41.715	17673295	167996	99.367	97.945
Total		17785885	171521	100.000	100.000

Enantioenriched **6k**

tert-Butyl (2*R*,5'*R*)-3-oxo-5'-(thiophen-2-yl)-3*H*-spiro[benzofuran-2,1'-cyclopentan]-2'-ene-2'-carboxylate **6l**

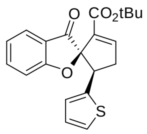

A yellow solid; $[\alpha]^{25}{}_{D}$ = -226.6 (c 1, $CHCl_3$); ^{1}H NMR (500 MHz, $CDCl_3$) δ 7.65–7.60 (m, 1H), 7.52–7.46 (m, 1H), 7.39 (t, *J* = 2.4 Hz, 1H), 7.12–7.08 (m, 1H), 6.99 (t, *J* = 7.4 Hz, 1H), 6.92 (d, *J* = 8.4 Hz, 1H), 6.78 (d, *J* = 3.4 Hz, 2H), 4.31 (t, *J* = 8.1 Hz, 1H), 3.09 (dddd, *J* = 20.6 Hz, 18.6 Hz, 8.1Hz, 2.5 Hz, 2H), 1.18 (s, 9H); ^{13}C NMR (125 MHz, $CDCl_3$) δ 201.44, 171.22, 161.23, 151.01, 138.04, 137.72, 136.37, 126.50, 126.16, 125.06, 123.98, 122.18, 121.59, 112.80, 96.17, 81.72, 49.25, 39.92, 27.62; HRMS (ESI) m/z calcd for $C_{21}H_{20}NaO_4S$ $[M + Na]^+$ = 391.0975, found = 391.0984; The ee value was 99%, t_R (minor) = 8.576 min, t_R (major) = 29.776 min (Chiralpak IC, λ = 254 nm, 10% *i*-PrOH/hexane, flow rate = 1.0 mL/min).

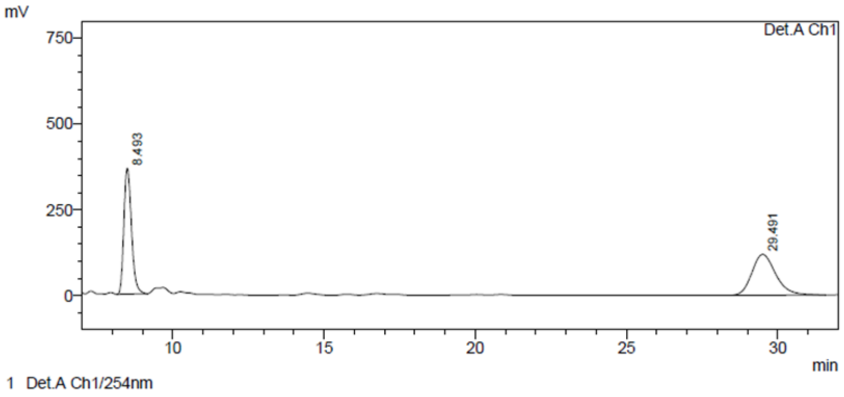

PeakTable

Detector A Ch1 254nm

Peak#	Ret. Time	Area	Height	Area %	Height %
1	8.493	6434772	367050	49.605	75.491
2	29.491	6537352	119165	50.395	24.509
Total		12972124	486215	100.000	100.000

Racemic **6l**

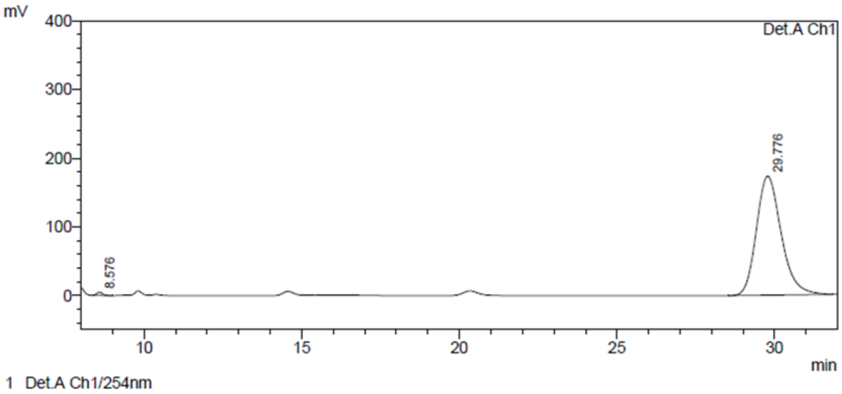

PeakTable

Detector A Ch1 254nm

Peak#	Ret. Time	Area	Height	Area %	Height %
1	8.576	59326	4637	0.623	2.607
2	29.776	9470876	173251	99.377	97.393
Total		9530202	177889	100.000	100.000

Enantioenriched **6l**

<u>*tert*-Butyl (2*R*,5'*S*)-5'-cyclohexyl-3-oxo-3*H*-spiro[benzofuran-2,1'-cyclopentan]-2'-ene-2'-carboxylate **6m**</u>

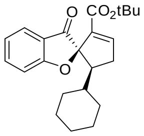

Colorless oil; $[\alpha]^{25}{}_{D}$ = -32.1 (c 1, $CHCl_3$); ^{1}H NMR (500 MHz, $CDCl_3$) δ 7.70 (dd, *J* = 7.8 Hz, 1.2 Hz, 1H), 7.60 (ddd, *J* = 8.6 Hz, 7.3 Hz, 1.4 Hz, 1H), 7.31–7.28 (m, 1H), 7.11–7.04 (m, 2H), 2.72 (ddd, *J* = 17.8 Hz, 7.0 Hz, 3.0 Hz, 1H), 2.67–2.59 (m, 1H), 2.54 (ddd, *J* = 17.8 Hz, 7.9 Hz, 2.1 Hz, 1H), 1.77 (d, *J* = 12.8 Hz, 1H), 1.73–1.66 (m, 1H), 1.62–1.54 (m, 2H), 1.46–1.39 (m, 1H), 1.25–1.17 (m, 1H), 1.12 (s, 9H), 1.09–0.92 (m, 4H), 0.83–0.71 (m, 1H); ^{13}C NMR (125 MHz, $CDCl_3$) δ 202.75, 171.63, 161.64, 151.46, 137.40, 136.98, 124.28, 122.56, 121.47, 112.47, 97.30, 81.39, 54.29, 37.18, 35.39, 32.48, 31.29, 27.59, 26.14, 26.11, 25.79; HRMS (ESI) m/z calcd for $C_{23}H_{28}NaO_4$ $[M + Na]^+$ = 391.1880, found = 391.1889; The ee value was 98%, t_R (minor) = 13.070 min, t_R (major) = 25.798 min (Chiralpak IC, λ = 254 nm, 3% *i*-PrOH/hexane, flow rate = 1.0 mL/min).

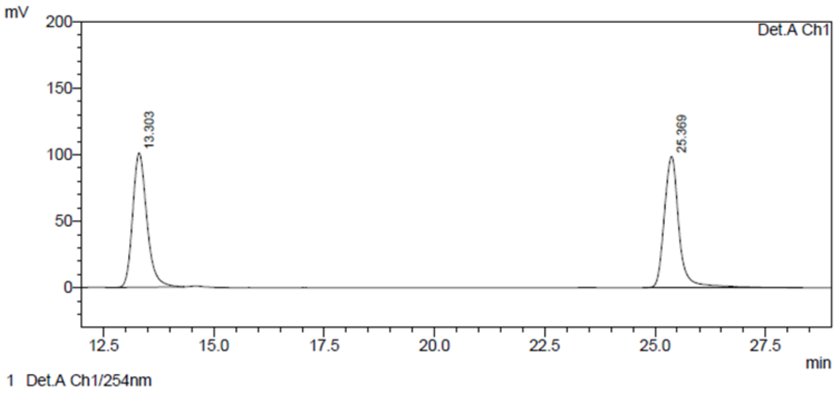

PeakTable

Detector A Ch1 254nm

Peak#	Ret. Time	Area	Height	Area %	Height %
1	13.303	2257567	100913	49.646	50.577
2	25.369	2289749	98612	50.354	49.423
Total		4547316	199524	100.000	100.000

Racemic **6m**

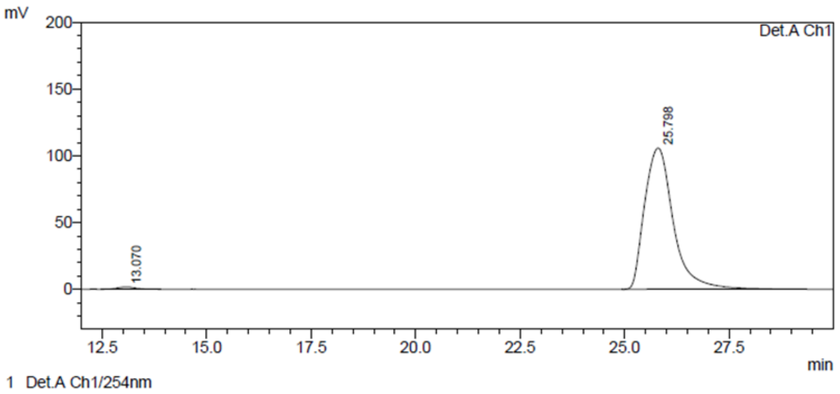

PeakTable

Detector A Ch1 254nm

Peak#	Ret. Time	Area	Height	Area %	Height %
1	13.070	46310	1672	0.908	1.557
2	25.798	5051665	105691	99.092	98.443
Total		5097975	107363	100.000	100.000

Enantioenriched **6m**

tert-Butyl (2*R*,5'*S*)-5'-isopropyl-3-oxo-3*H*-spiro[benzofuran-2,1'-cyclopentan]-2'-ene-2'-carboxylate **6n**

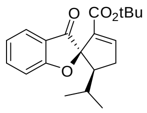

A while solid; $[\alpha]^{25}_{D}$ = -47.4 (c 1, $CHCl_3$); ^1H NMR (500 MHz, $CDCl_3$) δ 7.70 (dd, *J* = 7.9, 1.1 Hz, 1H), 7.63–7.57 (m, 1H), 7.33–7.25 (m, 1H), 7.11–7.02 (m, 2H), 2.79–2.68 (m, 1H), 2.61–2.49 (m, 2H), 1.95–1.83 (m, 1H), 1.12 (s, 9H), 0.94 (d, *J* = 6.7 Hz, 3H), 0.60 (d, *J* = 6.6 Hz, 3H); ^{13}C NMR (125 MHz, $CDCl_3$) δ 202.90, 171.71, 161.59, 151.51, 137.45, 136.97, 124.26, 122.53, 121.49, 112.56, 97.40, 81.40, 55.52, 35.76, 27.85, 27.58, 22.19, 21.17; HRMS

(ESI) m/z calcd for $C_{20}H_{24}NaO_4$ $[M + Na]^+$ = 351.1567, found = 351.1570; The ee value was 98%, t_R (minor) = 8.778 min, t_R (major) = 13.051 min (Chiralpak IC, λ = 254 nm, 5% *i*-PrOH/hexane, flow rate = 1.0 mL/min).

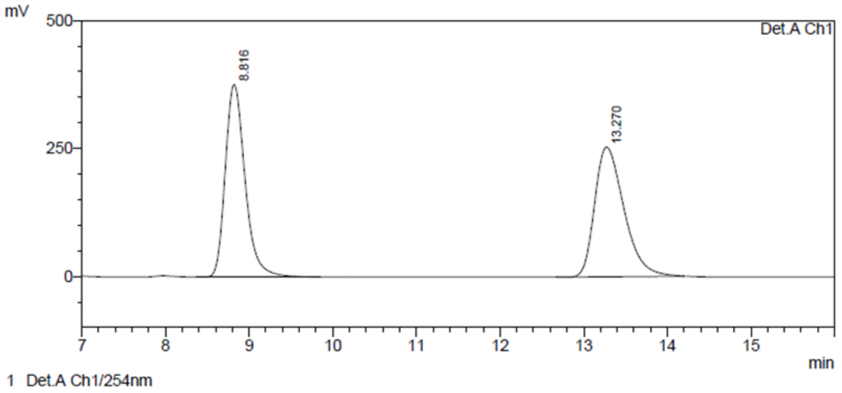

PeakTable

Detector A Ch1 254nm

Peak#	Ret. Time	Area	Height	Area %	Height %
1	8.816	6195970	375959	50.019	59.744
2	13.270	6191372	253326	49.981	40.256
Total		12387342	629285	100.000	100.000

Racemic **6n**

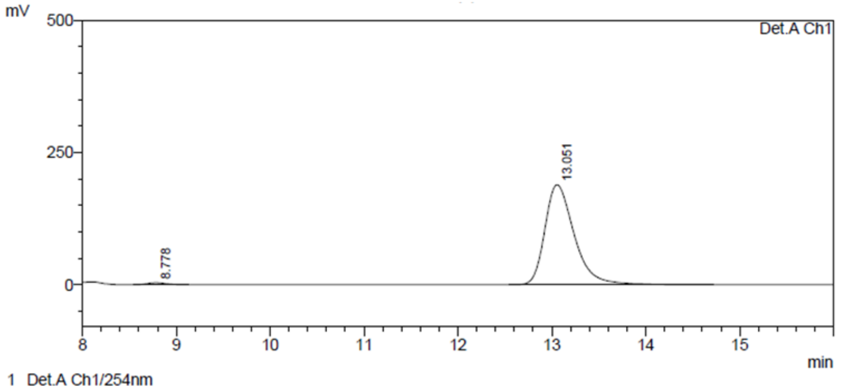

PeakTable

Detector A Ch1 254nm

Peak#	Ret. Time	Area	Height	Area %	Height %
1	8.778	46883	3709	1.128	1.927
2	13.051	4110486	188820	98.872	98.073
Total		4157369	192529	100.000	100.000

Enantioenriched **6n**

<u>*tert*-Butyl (2*R*,5'*R*)-5'-butyl-3-oxo-3*H*-spiro[benzofuran-2,1'-cyclopentan]-2'-ene-2'-carboxylate **6o**</u>

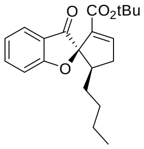

Colorless oil; $[\alpha]^{25}{}_{D}$ = -100.2 (c 0.5, $CHCl_3$); ^{1}H NMR (500 MHz, $CDCl_3$) δ 7.70 (d, *J* = 7.6 Hz, 1H), 7.65–7.58 (m, 1H), 7.29 (t, *J* = 2.4 Hz, 1H), 7.14–7.05 (m, 2H), 2.83 (ddd, *J* = 18.4 Hz, 7.5 Hz, 3.0 Hz, 1H), 2.73 (dtd, *J* = 9.8 Hz, 7.5 Hz, 5.1 Hz, 1H), 2.42 (ddd, *J* = 18.4 Hz, 7.5, 2.0 Hz, 1H), 1.51–1.42 (m, 2H), 1.25–1.18 (m, 3H), 1.15 (s, 9H), 1.11–1.04 (m, 1H), 0.79 (t, *J* = 7.1 Hz, 3H); ^{13}C NMR (125 MHz, $CDCl_3$) δ 202.64, 171.76, 161.54, 151.73, 137.65, 136.57, 124.23, 122.21, 121.47, 112.73, 97.73, 81.38, 48.48, 37.63, 30.05, 27.88, 27.59, 22.63, 13.86; HRMS (ESI) m/z calcd for C 21 H 26 Na O 4 $[M + Na]^+$ = 365.1723, found = 365.1726; The ee value was 97%, t_R (minor) = 8.766 min, t_R (major) = 16.894 min (Chiralpak IC, λ = 254 nm, 5% *i*-PrOH/hexane, flow rate = 1.0 mL/min).

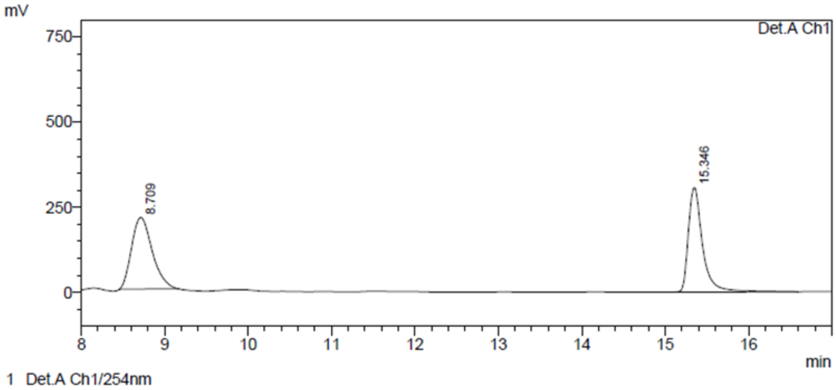

PeakTable

Detector A Ch1 254nm

Peak#	Ret. Time	Area	Height	Area %	Height %
1	8.709	3630073	210077	49.771	40.593
2	15.346	3663488	307446	50.229	59.407
Total		7293560	517523	100.000	100.000

Racemic **6o**

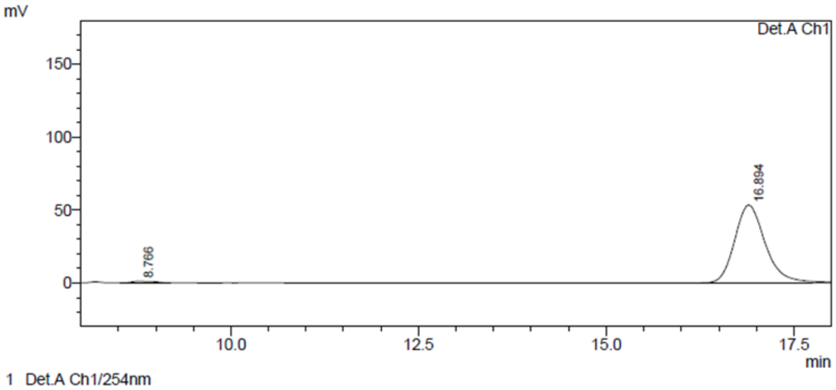

PeakTable

Detector A Ch1 254nm

Peak#	Ret. Time	Area	Height	Area %	Height %
1	8.766	22438	1058	1.444	1.943
2	16.894	1531803	53371	98.556	98.057
Total		1554241	54429	100.000	100.000

Enantioenriched **6o**

tert-Butyl (2*R*,5'*R*)-5'-ethyl-3-oxo-3*H*-spiro[benzofuran-2,1'-cyclopentan]-2'-ene-2'-carboxylate **6p**

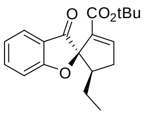

Colorless oil; $[\alpha]^{25}_{D}$ = -98.2 (c 1, $CHCl_3$); ^1H NMR (500 MHz, $CDCl_3$) δ 7.74–7.67 (m, 1H), 7.66–7.57 (m, 1H), 7.30 (t, *J* = 2.4 Hz, 1H), 7.12–7.05 (m, 2H), 2.85 (ddd, *J* = 18.5 Hz, 7.5 Hz, 3.0 Hz, 1H), 2.72–2.63 (m, 1H), 2.42 (ddd, *J* = 18.6 Hz, 7.6 Hz, 2.0 Hz, 1H), 1.54–1.48 (m, 1H), 1.40–1.30 (m, 1H), 1.15 (s, 9H), 0.80 (t, *J* = 7.4 Hz, 3H); ^{13}C NMR (125 MHz, $CDCl_3$) δ 202.68, 171.78, 161.54, 151.67, 137.65, 136.56, 124.25, 122.17, 121.49, 112.71, 97.65, 81.39, 50.21, 37.33, 27.59, 21.43, 12.35; HRMS (ESI) m/z calcd for $C_{19}H_{22}NaO_4$ $[M + Na]^+$ = 337.1410, found = 337.1415; The ee value was 98%, t_R (minor) = 9.615 min, t_R (major) = 17.757 min (Chiralpak IC, λ = 254 nm, 5% *i*-PrOH/hexane, flow rate = 1.0 mL/min).

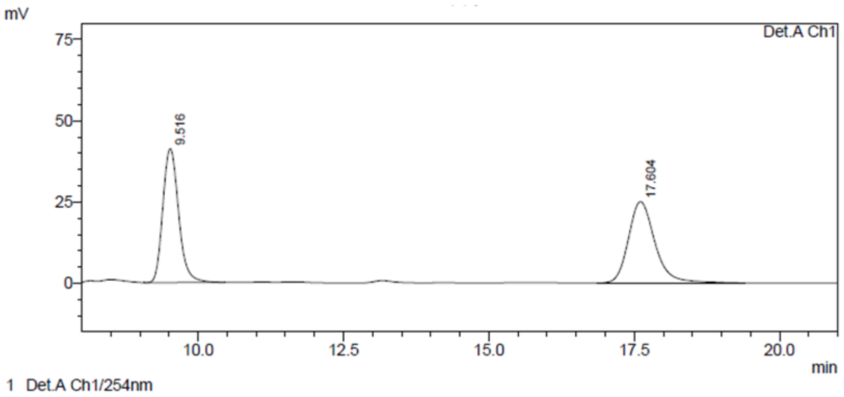

PeakTable

Detector A Ch1 254nm

Peak#	Ret. Time	Area	Height	Area %	Height %
1	9.516	779865	41221	49.769	62.264
2	17.604	787097	24983	50.231	37.736
Total		1566962	66204	100.000	100.000

Racemic **6p**

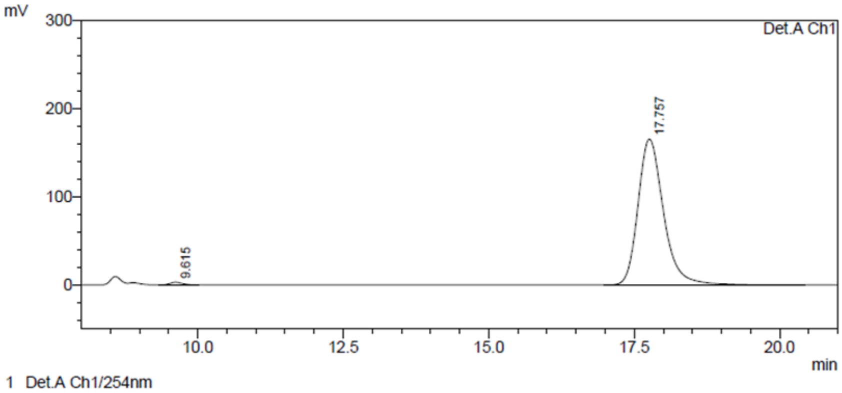

PeakTable

Detector A Ch1 254nm

Peak#	Ret. Time	Area	Height	Area %	Height %
1	9.615	41234	2977	0.816	1.773
2	17.757	5014952	164954	99.184	98.227
Total		5056186	167931	100.000	100.000

Enantioenriched **6p**

E. Synthesis of product 8

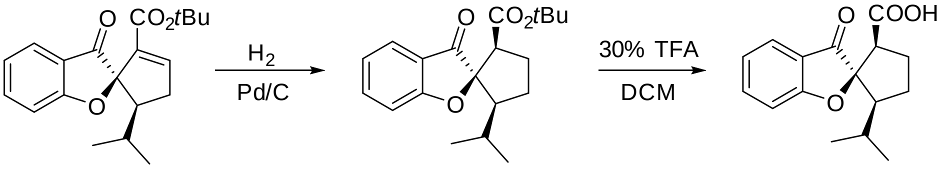

6n (98% ee)

7: dr > 19:1, 90% yield, 97 % ee

8: dr > 19:1, 94% yield, 98 % ee

To **6n** (65.7 mg, 0.2 mmol) in methanol (2 mL) was added Pd/C (6.6 mg, 10%), and H_2 was introduced to the system. After 12 hour, Pd/C was removed by filtration. The mixture was concentrated, and the residue was purified by flash column chromatography (hexane/ether = 10 : 1) to afford **7** as a colorless oil (59.5 mg, >19:1 dr, 90% yield).

7: $[\alpha]^{25}_{D}$ = 50.8 (c 1, $CHCl_3$); ^{1}H NMR (500 MHz, $CDCl_3$) δ 7.70 (d, *J* = 7.5 Hz, 1H), 7.64–7.56 (m, 1H), 7.09 (t, *J* = 7.8 Hz, 2H), 3.38 (dd, *J* = 10.3 Hz, 8.2 Hz, 1H), 2.48–2.35 (m, 1H), 2.21 (dt, *J* = 11.9 Hz, 8.0 Hz, 1H), 2.12–1.95 (m, 2H), 1.86 (ddd, *J* = 18.4 Hz, 11.6 Hz, 7.2 Hz, 1H), 1.61 (dq, *J* = 13.5, 6.7 Hz, 1H), 0.97 (s, 9H), 0.84 (d, *J* = 6.7 Hz, 3H), 0.58 (d, *J* = 6.7 Hz, 3H); ^{13}C NMR (125 MHz, $CDCl_3$) δ 202.74, 171.68, 168.89, 137.35, 124.04, 123.31, 121.64, 113.05, 97.90, 81.01, 57.14, 54.79, 28.33, 27.60, 27.39, 23.83, 23.00, 21.31; HRMS (ESI) m/z calcd for $C_{20}H_{26}NaO_4$

[M + Na]$^+$ = 353.1723, found = 353.1729; The ee value was 97%, t_R (minor) = 5.421 min, t_R (major) = 6.734 min (Chiralpak IC, λ = 254 nm, 5% *i*-PrOH/hexane, flow rate = 1.0 mL/min).

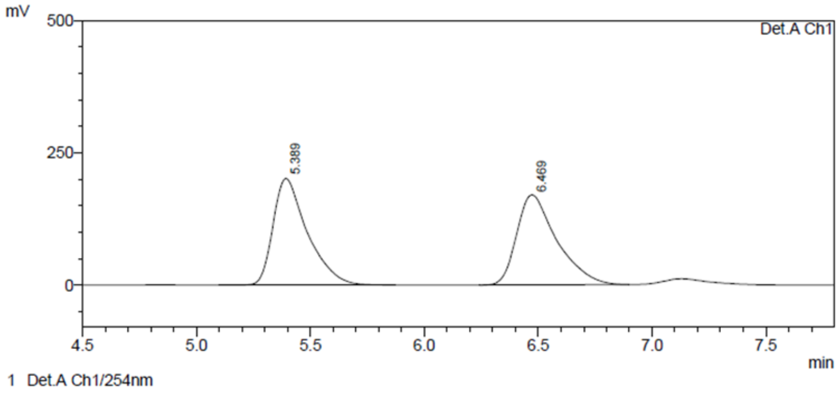

PeakTable

Detector A Ch1 254nm

Peak#	Ret. Time	Area	Height	Area %	Height %
1	5.389	2098596	201321	50.246	54.218
2	6.469	2078016	169994	49.754	45.782
Total		4176613	371315	100.000	100.000

Racemic **7**

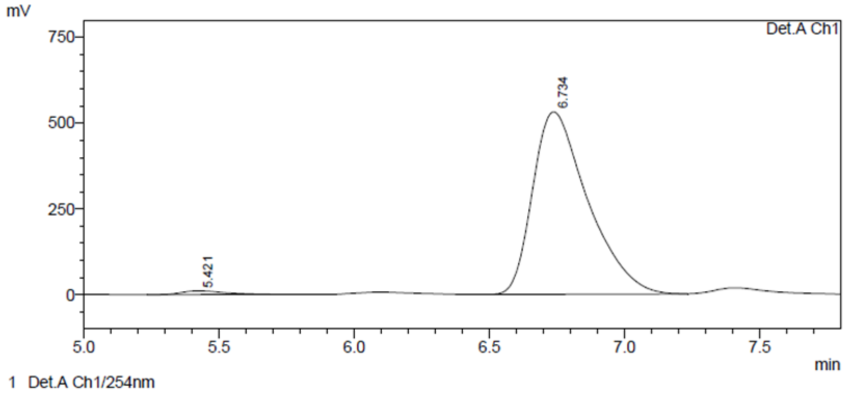

PeakTable

Detector A Ch1 254nm

Peak#	Ret. Time	Area	Height	Area %	Height %
1	5.421	113146	10878	1.478	2.007
2	6.734	7541252	531231	98.522	97.993
Total		7654398	542109	100.000	100.000

Enantioenriched **7**

To a CH_2Cl_2 (1.5 mL) solution of **7** (59.5 mg, 0.18 mmol) at 0 °C was added trifluoroacetic acid (0.75 mL, 30%) dropwise. The mixture was stirred at room temperature for 2 h, and the solvent was removed. The crude product was then purified by flash column chromatography (hexane/ethyl acetate = 1 : 2) to afford **8** as a colorless oil (46.4 mg, >19:1 dr, 94% yield).

8: $[\alpha]^{25}{}_{D}$ = 54.2 (c 1, $CHCl_3$); ^{1}H NMR (500 MHz, $CDCl_3$) δ 7.67–7.60 (m, 1H), 7.60–7.52 (m, 1H), 7.09–6.99 (m, 2H), 3.41 (t, *J* = 9.0 Hz, 1H), 2.36–2.23 (m, 1H), 2.16 (dt, *J* = 11.7 Hz, 8.0 Hz, 1H), 2.10–1.96 (m, 2H), 1.90–1.76 (m, 1H), 1.58 (tt, *J* = 13.5 Hz, 6.7 Hz, 1H), 0.82 (d, *J* = 6.7 Hz, 3H), 0.54 (d, *J* = 6.7 Hz, 3H); ^{13}C NMR (125 MHz, $CDCl_3$) δ 202.50, 171.55, 137.72, 124.14, 122.59, 121.94, 112.75, 97.16, 56.83, 53.80, 28.40, 27.58, 24.23, 22.87, 21.30; HRMS (ESI) m/z calcd for $C_{16}H_{18}NaO_4$ $[M + Na]^+$ = 297.1097, found = 297.1104; The ee value was 98%, t_R (major) = 6.900 min, t_R (minor) = 14.462 min (Chiralpak IC, λ = 254 nm, 30% *i*-PrOH/hexane, flow rate = 1.0 mL/min).

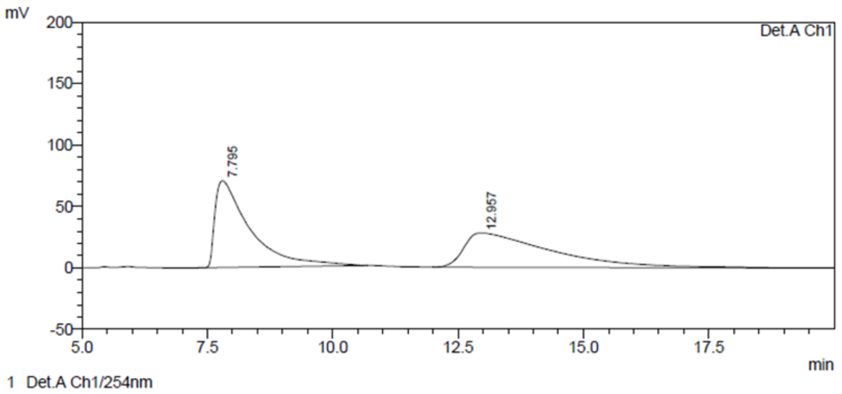

PeakTable

Detector A Ch1 254nm

Peak#	Ret. Time	Area	Height	Area %	Height %
1	7.795	3452025	70682	50.688	71.701
2	12.957	3358324	27897	49.312	28.299
Total		6810349	98580	100.000	100.000

Racemic **8**

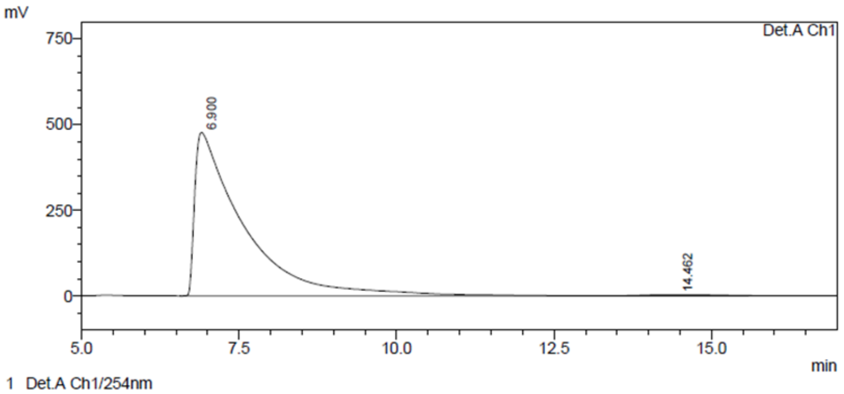

PeakTable

Detector A Ch1 254nm

Peak#	Ret. Time	Area	Height	Area %	Height %
1	6.900	25856126	476861	98.984	99.391
2	14.462	265318	2920	1.016	0.609
Total		26121444	479781	100.000	100.000

Enantioenriched **8**

F. Determination of stereochemistry of product 8 by 2D NMR

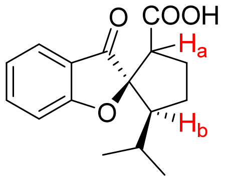

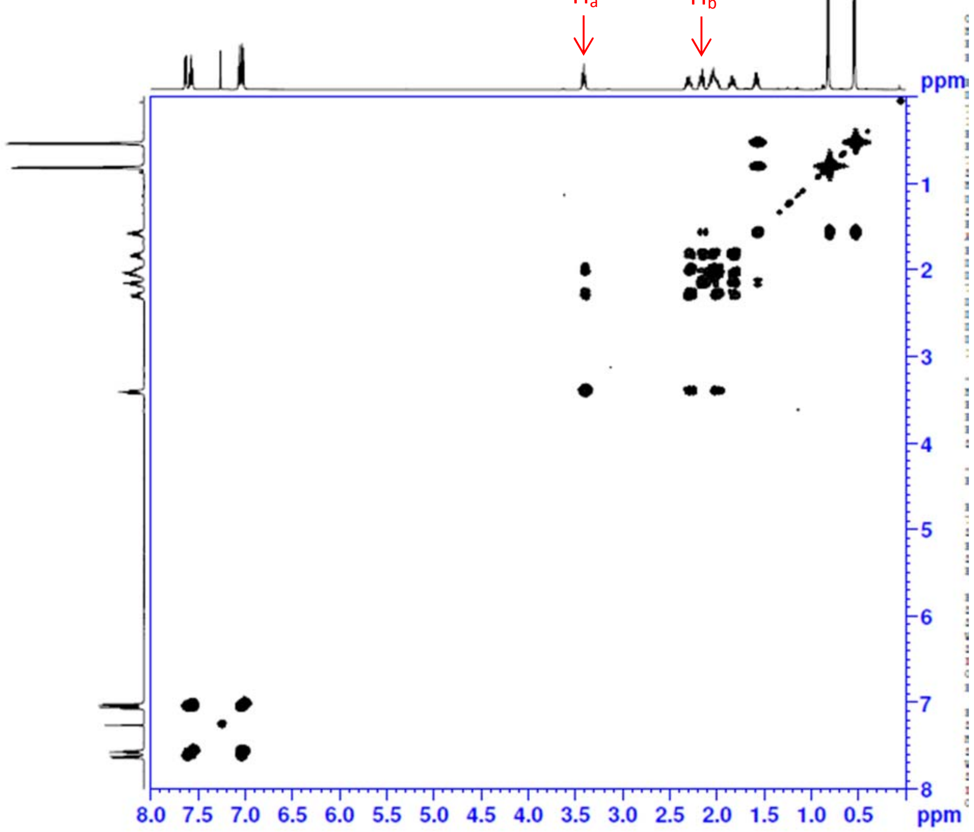

Figure S1. COSY spectrum for product **8**

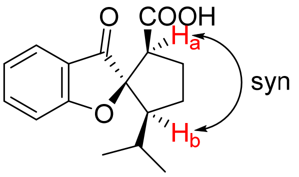

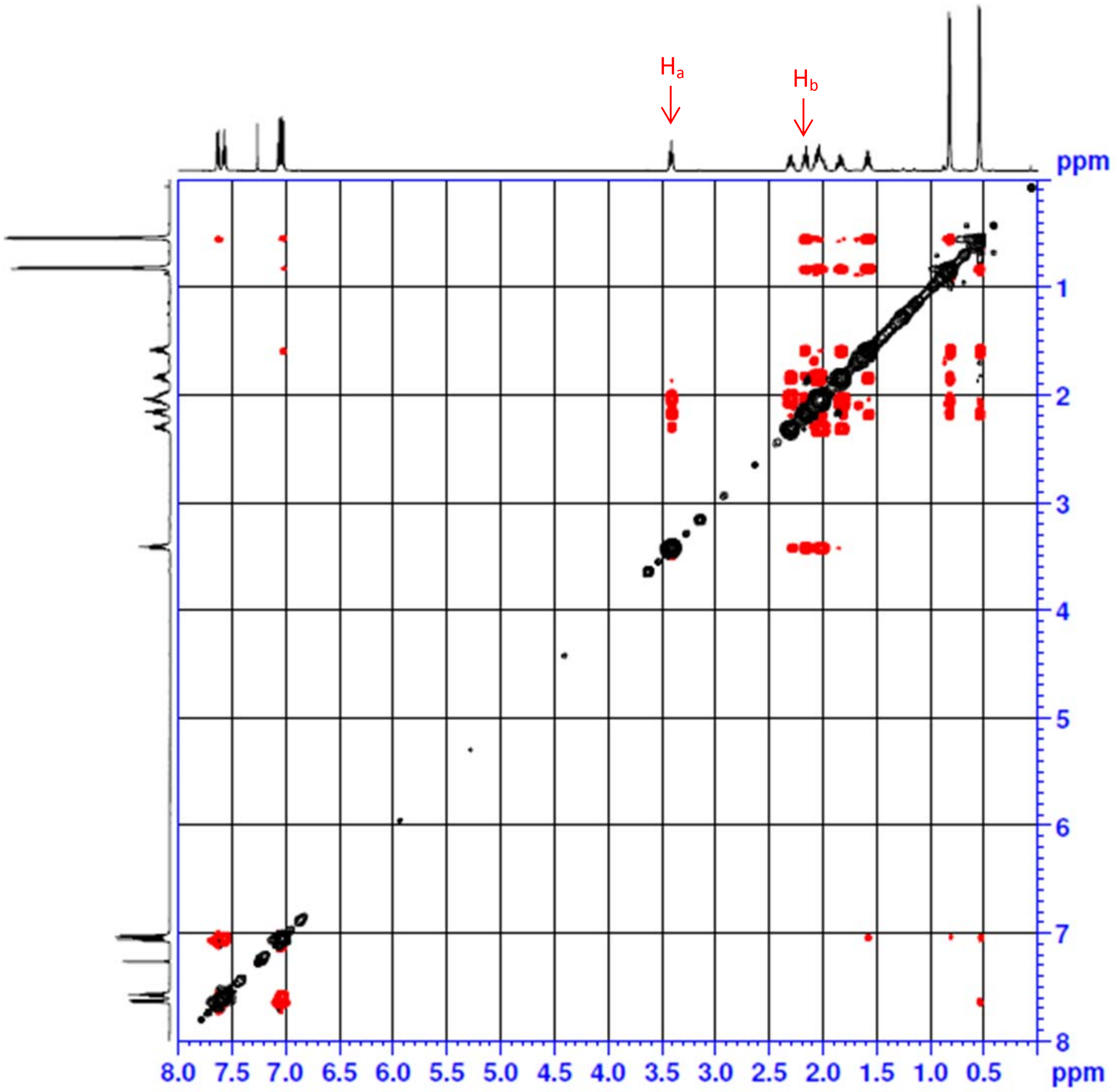

Figure S2. NOSEY spectrum of product **8**

Proton H_a and H_b were identified using COSY. NOSEY showed the two protons had correlations, which suggested they were on the same side of the cyclopentane ring. Therefore, the stereochemistry of the newly installed chiral center in product **8** could be determined as shown in the structure.

G. Computational methods

Density functional theory (DFT) methods, as implemented in the Gaussian 09 program[3], have been employed to study the phosphine catalyzed [3+2] annulation. Molecular geometries of the complexes were optimized via DFT calculations using the B3LYP[4]/6-31G(d)[5] level of theory. The vibrational frequencies were computed at the same

level of theory to determine whether the optimized structure was at an energy minimun. Solvent effects were determined within SMD[6] model (toluene) at the M11[7]/6-311+G(d)[8] level of theory using gas-phase-optimized geometries.

H. Proposed Reaction Cycle and DFT studies

The plausible mechanism for the phosphine-catalyzed [3+2] annulation of aurone with allene is shown in Scheme S1. Based on the previous exploration of phosphine-catalyzed [3+2] annulation reactions, we proposed that the reaction is initially undergoes the nucleophilic attack of the phosphine catalyst **A** on allene **2a** to give a zwitterionic species **B**. The allenoate moiety of intermediate **B** exists two resonance structures (**C** or **G**), in which the negative charge could be attributed to the α- or γ-position, respectively. The followed nucleophilic addition could take place with either α- or γ-position with aurone **1** to yield zwitterionic intermediate **D** or **H**, respectively. Subsequently, the stepwise [3+2] annulation yields phosphorus ylide **E** or **I**. Then the proton transfer affords α-annulation product **5** or γ-annulation product **6**, respectively, and regenerates the active catalyst **A**.

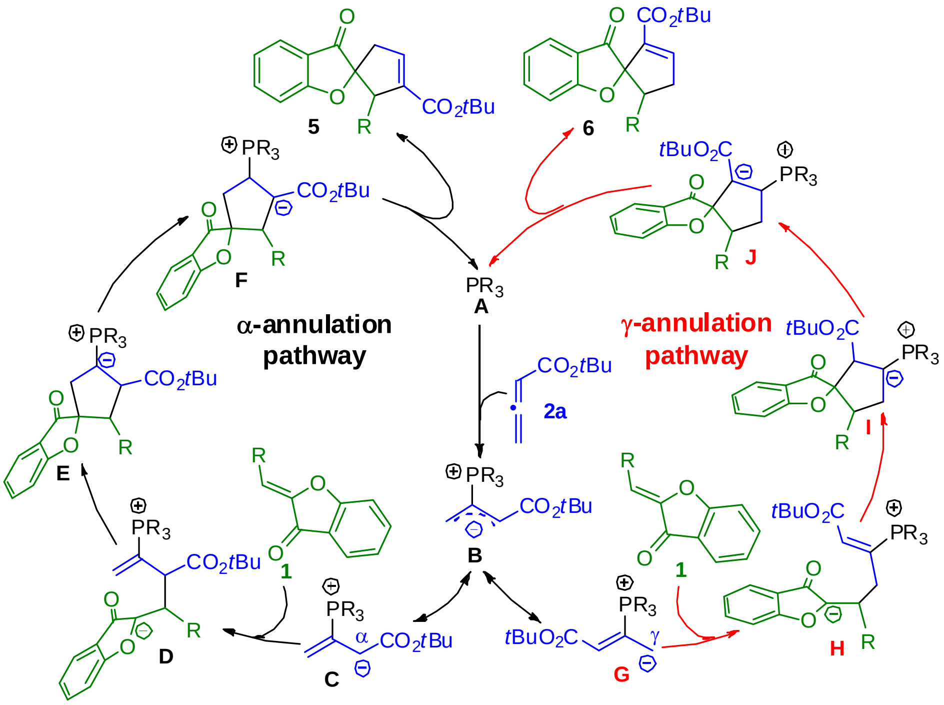

Scheme S1. Proposed mechanism for the phosphine-catalyzed [3+2] annulation of aurones with allenes.

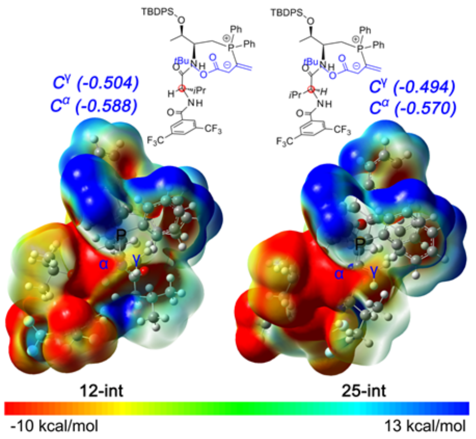

Figure S3. The B3LYP calculated NPA charge distributions for intermediate **12-int** and **25-int**.

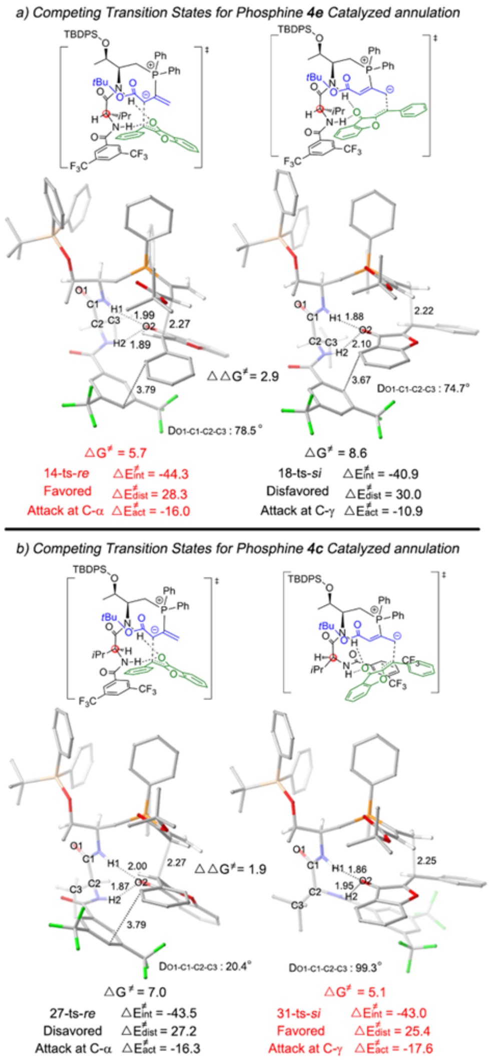

Figure S4. Optimized transition states **14-ts-*re* *(indicated as TS-1 in the main article)***, **18-ts-*si* *(TS-2)***, **27-ts-*re* *(TS-3)*** and **31-ts-*si* *(TS-4)***. The relative free energies are given in kilocalories per mole.

I. Calculated Gibbs free energy profiles for the phosphine-catalyzed [3+2] annulation of aurone 1a and allene 2a.

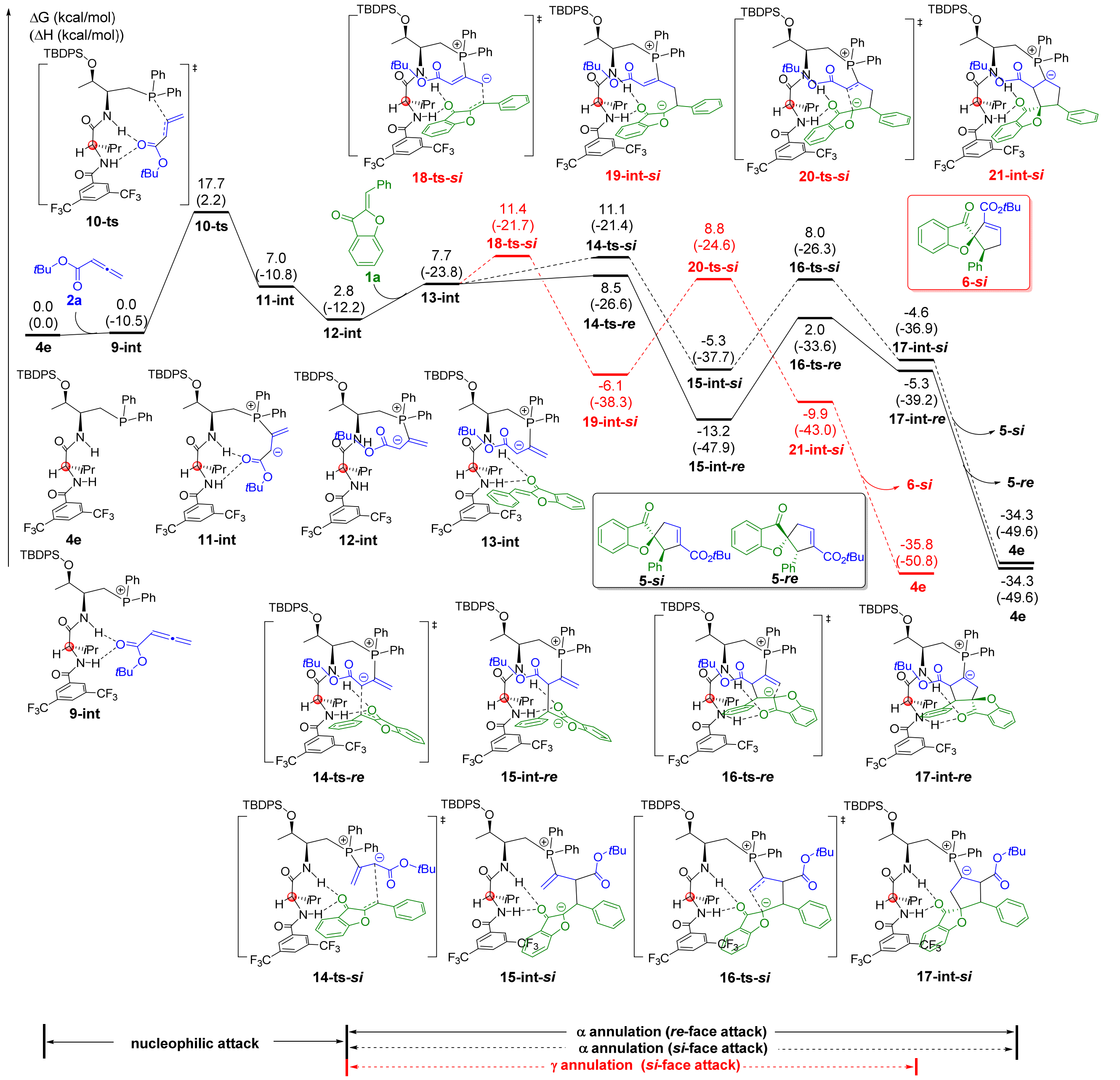

Figure S5. The DFT computed energy surfaces of the catalyst **4e** catalyzed [3+2] annulation of aurone **1a** and allene **2a**.

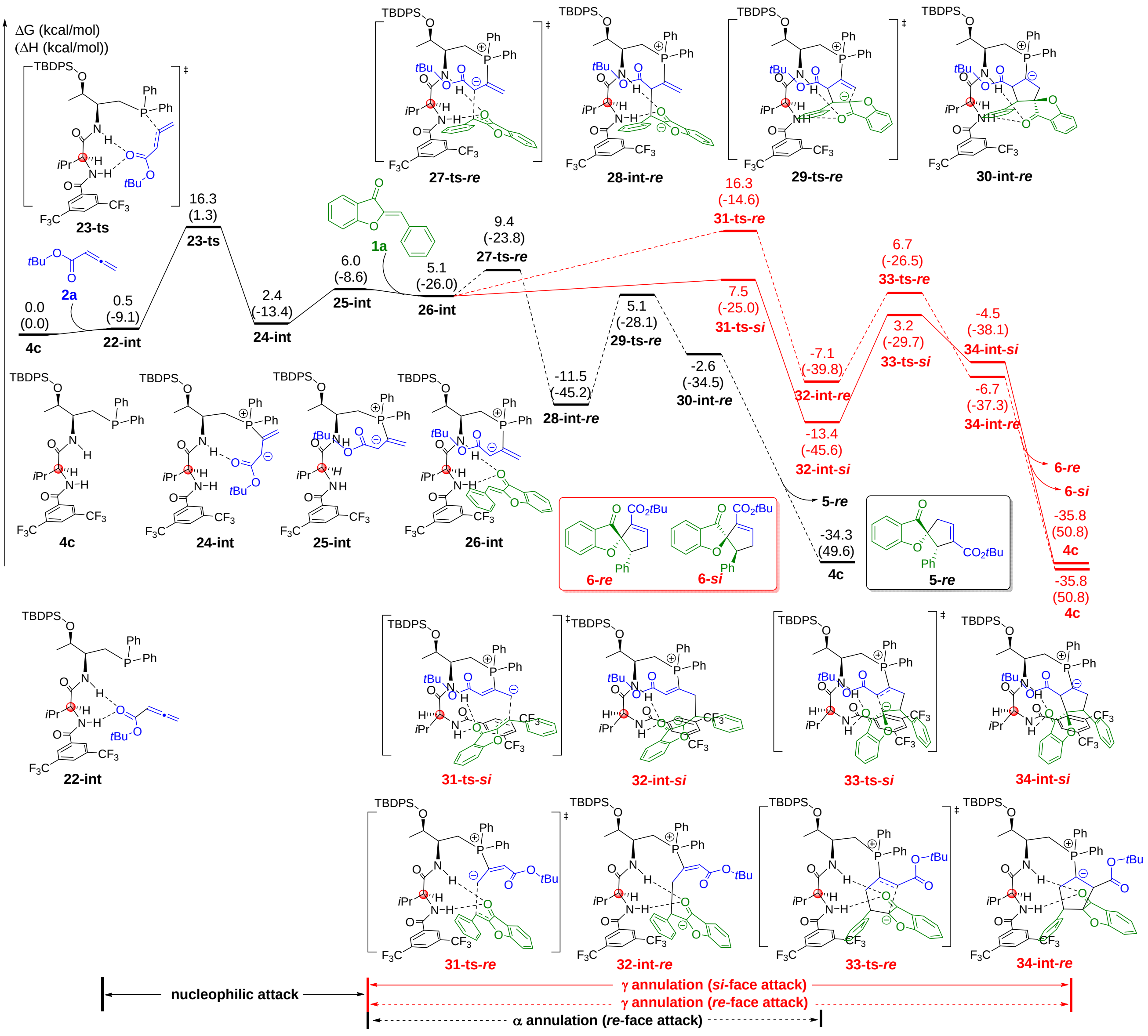

Figure S6. The DFT computed energy surfaces of the catalyst **4c** catalyzed [3+2] annulation of aurone **1a** and allene **2a**.

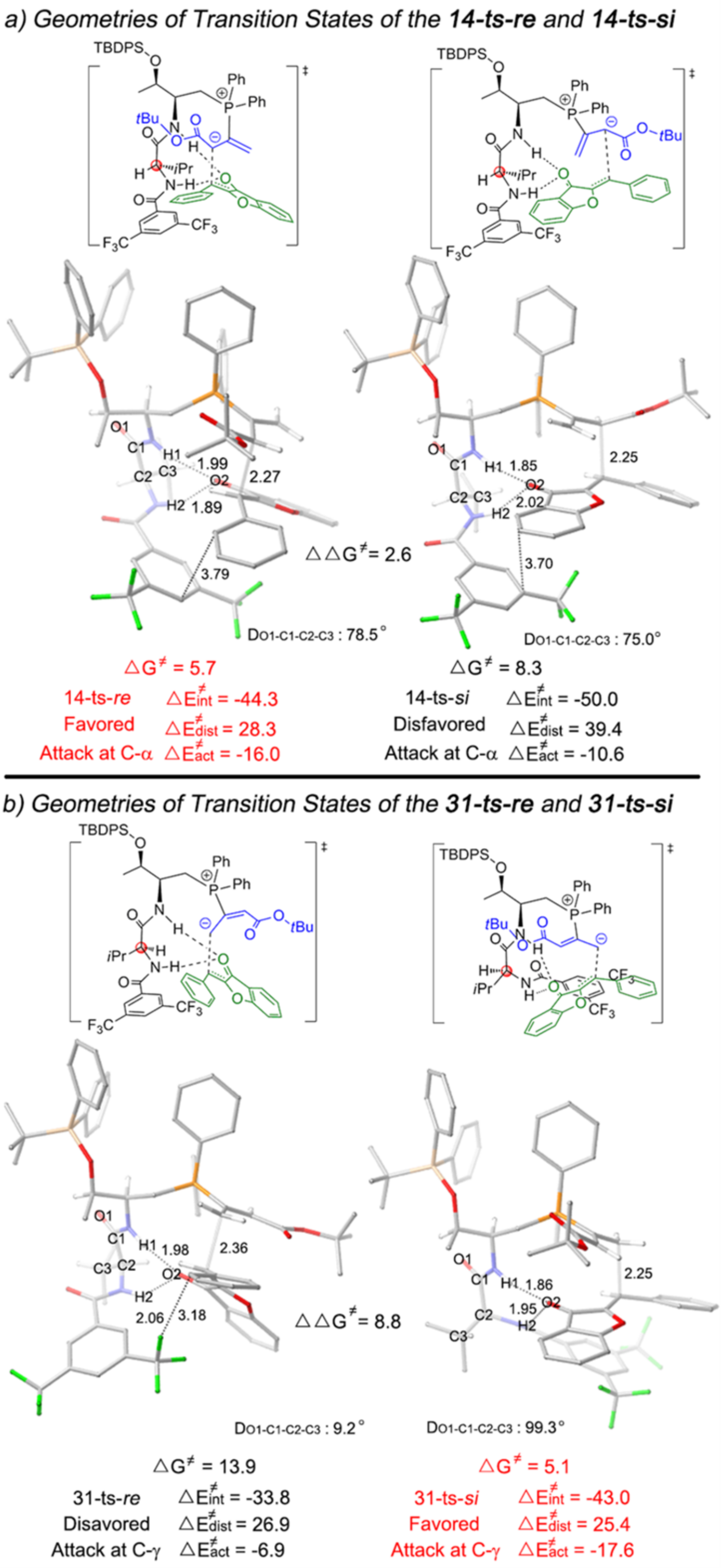

Figure S7. Optimized transition states **14-ts-*re***, **14-ts-*si***, **31-ts-*re*** and **31-ts-*si***. The relative free energies are given in kilocalories per mole.

J. B3LYP and M11 absolute calculation energies, enthalpies, and free energies.

Geometry	$E_{B3LYP(elec)}$ [1]	$E_{M11(toluene)}$ [2]	Correction of $H_{(gas\ phase)}$ [3]	Correction of $G_{(gas\ phase)}$ [4]	IF[5]
**4e**	-3347.558016	-3347.000467	0.928058	0.764175	-
**4c**	-3347.556026	-3347.000912	0.927932	0.765270	-
**2a**	-462.477481	-462.350509	0.196598	0.147071	-
**1a**	-728.044138	-727.825435	0.222091	0.168347	-
**5-*re***	-1190.582764	-1190.260076	0.423846	0.344874	-
**5-*si***	-1190.582764	-1190.260076	0.423846	0.344874	-
**6-*re***	-1190.583418	-1190.262196	0.424002	0.344547	-
**6-*si***	-1190.583418	-1190.262196	0.424001	0.344567	-
**9-int**	-3810.043834	-3809.369528	1.126470	0.929852	-
**10-ts**	-3810.020460	-3809.348553	1.125663	0.937091	-175.9
**11-int**	-3810.034457	-3809.371082	1.127619	0.942490	-
**12-int**	-3810.037234	-3809.373023	1.127207	0.937705	-
**13-int**	-4538.092965	-4537.219536	1.352010	1.134911	-
**14-ts-*re*(Ts-1)**	-4538.089472	-4537.223962	1.351894	1.140620	-199.7
**15-int-*re***	-4538.104124	-4537.260430	1.354382	1.142627	-
**16-ts-*re***	-4538.091498	-4537.236596	1.353453	1.143039	-334.8
**17-int-*re***	-4538.106525	-4537.246960	1.354859	1.141670	-
**14-ts-*si***	-4538.078578	-4537.215402	1.351617	1.136197	-233.2
**15-int-*si***	-4538.089827	-4537.243842	1.354131	1.138549	-
**16-ts-*si***	-4538.081625	-4537.225042	1.353409	1.140948	-321.6
**17-int-*si***	-4538.103189	-4537.243528	1.355080	1.139458	-
**18-ts-*si*(Ts-2)**	-4538.084950	-4537.215874	1.351636	1.137221	-315.4
**19-int-*si***	-4538.102559	-4537.245158	1.354390	1.138639	-
**20-ts-*si***	-4538.091201	-4537.222197	1.353335	1.139425	-264.2
**21-int-*si***	-4538.112030	-4537.252738	1.354495	1.140203	-
**22-int**	-3810.043531	-3809.367771	1.126402	0.929534	--
**23-ts**	-3810.023437	-3809.350542	1.125707	0.937517	-178.9

**24-int**	-3810.038959	-3809.375815	1.127497	0.940540	-
**25-int**	-3810.038678	-3809.368168	1.127628	0.938659	-
**26-int**	-4538.095333	-4537.223741	1.352080	1.135698	-
**27-ts-*re*(Ts-3)**	-4538.085904	-4537.219600	1.351381	1.138415	-200.3
**28-int-*re***	-4538.100801	-4537.256523	1.354199	1.142013	-
**29-ts-*re***	-4538.087976	-4537.227978	1.352992	1.139908	-321.0
**30-int-*re***	-4538.104990	-4537.239763	1.354595	1.139429	-
**31-ts-*re***	-4538.080092	-4537.204617	1.351129	1.134420	-170.3
**32-int-*re***	-4538.101517	-4537.248162	1.354554	1.140672	-
**33-ts-*re***	-4538.088533	-4537.225440	1.353021	1.139891	-282.4
**34-int-*re***	-4538.107356	-4537.244492	1.354810	1.137646	-
**31-ts-*si*(Ts-4)**	-4538.088897	-4537.221670	1.351588	1.137408	-286.3
**32-int-*si***	-4538.106180	-4537.257429	1.354518	1.139924	-
**33-ts-*si***	-4538.088677	-4537.230659	1.353085	1.139631	-302.9
**34-int-*si***	-4538.099215	-4537.245332	1.354397	1.142048	-

[1]The electronic energy calculated by B3LYP in gas phase. [2]The electronic energy calculated by M11 in toluene. [3]The thermal correction to enthalpy calculated by B3LYP in gas phase. [4]The thermal correction to Gibbs free energy calculated by B3LYP in gas phase. [5]The B3LYP calculated imaginary frequencies for the transition states in gas phase.

K. B3LYP geometries for all the optimized compounds and transition states.

4e

P	0.07543100	0.24899000	1.68448100
C	1.05201700	0.34832600	0.06653100
H	2.10693800	0.49454900	0.31454000
H	0.73391500	1.22570700	-0.50865100
C	0.96195400	-0.93271500	-0.77018800
H	1.29736800	-1.77352100	-0.15628700
C	1.87110300	-0.91781900	-2.03185000
H	1.64904000	-1.84231000	-2.57605200
C	1.63273800	0.27082700	-2.96237300
H	1.94059200	1.20729900	-2.48644600
H	0.57551700	0.34829200	-3.24030600
H	2.22000300	0.15300700	-3.87820500
C	0.83384400	1.65694300	2.62245700
C	0.05956700	2.62093200	3.28869300
C	2.22742100	1.68077600	2.81202500
C	0.66058500	3.59362400	4.09074800
H	-1.02053100	2.61869500	3.17997000
C	2.82763300	2.65605000	3.60812500
H	2.85665200	0.92709000	2.34491700
C	2.04647300	3.62027000	4.24873900
H	0.04014700	4.33265100	4.59146100
H	3.90759900	2.65806100	3.73105000
H	2.51345700	4.37911100	4.87073400
C	-1.55369600	0.94395200	1.11974000
C	-2.68609400	0.12059400	1.22918500
C	-1.70491000	2.21069600	0.52461100
C	-3.93212800	0.53829600	0.74997600
H	-2.59539300	-0.86418600	1.67889700
C	-2.94949000	2.63698500	0.06020300
H	-0.84749200	2.87296000	0.43630000
C	-4.06467400	1.79963300	0.16848500
H	-4.78030000	-0.13498600	0.82376400
H	-3.04903300	3.62125800	-0.39054800
H	-5.03128400	2.13085400	-0.20202000
O	3.23220600	-0.90079000	-1.62105500
Si	4.41143900	-2.09227800	-1.47536400
C	4.17689700	-3.04379900	0.14828500
C	3.03210400	-3.83814500	0.37628700
C	5.11560100	-2.95133600	1.19395900
C	2.84454000	-4.51267200	1.58405200
H	2.26031400	-3.92392700	-0.38372600
C	4.93458100	-3.63035600	2.40144900
H	5.99963400	-2.33266400	1.07176300
C	3.79884900	-4.41582400	2.59881800
H	1.95038500	-5.11288800	1.73178600
H	5.67872900	-3.53953700	3.18897400

H	3.65385300	-4.94291300	3.53843000
C	5.99405100	-1.05556200	-1.40587100
C	5.93561500	0.33151400	-1.63952100
C	7.26237500	-1.61272300	-1.14423600
C	7.08528700	1.12429100	-1.61630400
H	4.97299100	0.79276600	-1.83677900
C	8.41520300	-0.82533900	-1.11875900
H	7.36004800	-2.67796700	-0.95055100
C	8.32931800	0.54784900	-1.35645900
H	7.00854700	2.19321300	-1.80040600
H	9.37900500	-1.28419000	-0.91261500
H	9.22541700	1.16302700	-1.33763300
C	4.43340200	-3.26598200	-3.01143200
C	3.27687300	-4.29120900	-3.01151600
H	3.31661100	-4.89024100	-3.93339600
H	2.28287900	-3.83296500	-2.96477600
H	3.35631900	-4.98641600	-2.16940300
C	5.75941000	-4.06226900	-3.02575000
H	5.75984500	-4.76467900	-3.87171800
H	5.89315200	-4.65567700	-2.11222900
H	6.63111100	-3.40998400	-3.14026100
C	4.36197500	-2.41492900	-4.29903300
H	5.15384000	-1.65724700	-4.33364800
H	3.40057100	-1.89903300	-4.39999600
H	4.48110100	-3.05827400	-5.18294800
N	-0.42963800	-1.22170900	-1.14327200
H	-1.11790000	-0.48650900	-1.04770000
C	-0.86578300	-2.48175200	-1.38441900
O	-0.12033900	-3.46475600	-1.42582300
C	-2.37946500	-2.66568800	-1.59635600
H	-2.94495900	-1.82025100	-1.19356200
C	-2.72625800	-2.85390700	-3.09957400
C	-2.30638000	-1.63109400	-3.92974700
H	-2.57146200	-1.77883500	-4.98228800
H	-1.22823100	-1.44715400	-3.88234300
H	-2.82170600	-0.72445500	-3.58527700
C	-4.22130200	-3.14775300	-3.28412400
H	-4.83447600	-2.31132500	-2.92698200
H	-4.52663100	-4.04487900	-2.73793200
H	-4.44954600	-3.30457300	-4.34450500
N	-2.74510400	-3.83892600	-0.80658500
H	-2.02928400	-4.55653600	-0.79250600
C	-3.68840600	-3.79892600	0.17794000
O	-4.38808700	-2.81176900	0.40327300
H	-2.15000000	-3.72235800	-3.44862300
C	-3.85032100	-5.05786300	0.99366400
C	-4.54693700	-4.94529500	2.20158900

C	-3.37737600	-6.31176900	0.59095000
C	-4.74610600	-6.06548800	3.00658200
H	-4.93666400	-3.97431400	2.48482800
C	-3.58469000	-7.43205900	1.39771800
H	-2.87906700	-6.44093900	-0.36362900
C	-4.26509700	-7.31428700	2.60988000
H	-4.43722800	-8.18849800	3.22700300
C	-3.02919400	-8.77076800	0.98282600
C	-5.44509100	-5.91749000	4.33477800
F	-1.79393500	-8.96903900	1.49453500
F	-3.80381100	-9.78839300	1.41404200
F	-2.92350900	-8.87482000	-0.35950000
F	-4.57387100	-5.58690700	5.31376600
F	-6.04858500	-7.06530400	4.71168700
F	-6.38572100	-4.95165600	4.29806100

4c

P	-3.81031400	1.88149500	-1.74118500
C	-3.64596300	1.20650500	-3.48925900
H	-3.48542300	2.00807300	-4.21738100
H	-4.62585400	0.76875600	-3.71886200
C	-2.54352200	0.14736300	-3.64979800
H	-1.59506400	0.53626300	-3.26826500
C	-2.28128500	-0.25688500	-5.12858000
H	-1.51001400	-1.03456500	-5.08818600
C	-3.50997600	-0.81409700	-5.84540000
H	-4.26935400	-0.03701300	-5.97937000
H	-3.95025200	-1.64381200	-5.28160800
H	-3.23345500	-1.18598900	-6.83693300
C	-2.43318800	3.11402400	-1.61961100
C	-2.27057300	3.74695600	-0.37365600
C	-1.53016000	3.42603000	-2.64683300
C	-1.25637600	4.68026800	-0.16874600
H	-2.94906500	3.50606000	0.44217400
C	-0.50078600	4.34840900	-2.43658100
H	-1.61070600	2.95138700	-3.61900200
C	-0.36442800	4.98153400	-1.20169700
H	-1.15517500	5.16465400	0.79922300
H	0.19689400	4.55997200	-3.24176900
H	0.43597700	5.69897200	-1.04062200
C	-5.28183100	2.98633300	-1.98691300
C	-6.43940100	2.70450700	-1.24761600
C	-5.30069000	4.07476700	-2.87478500
C	-7.59574000	3.47382900	-1.40021000
H	-6.43432100	1.88490100	-0.53508000
C	-6.45299500	4.84522800	-3.02930100
H	-4.40593900	4.32933200	-3.43727800
C	-7.60448400	4.54378900	-2.29497500
H	-8.47865800	3.23454800	-0.81427800
H	-6.45279900	5.68509000	-3.71969000
H	-8.50054200	5.14701600	-2.41623000
O	-1.80394500	0.88111100	-5.84319500
Si	-0.34614200	1.27444000	-6.57707800
C	0.99734600	1.51574600	-5.26091800
C	1.17565100	0.56988800	-4.23013200
C	1.85069900	2.63668200	-5.26131500
C	2.15854100	0.73449000	-3.25217300
H	0.53375700	-0.30246600	-4.15019800
C	2.84370900	2.79939800	-4.29224900
H	1.73477100	3.40631500	-6.01899200
C	3.00074600	1.84676800	-3.28410500
H	2.25319700	-0.00728700	-2.46384800
H	3.48889400	3.67431600	-4.32183300
H	3.76778000	1.97550300	-2.52449600
C	-0.76852800	2.91045500	-7.43160300
C	-2.08349700	3.41053000	-7.36967900
C	0.17287600	3.66628600	-8.15952000
C	-2.44025500	4.60643500	-7.99613600
H	-2.83637100	2.84899400	-6.82536900
C	-0.17740600	4.86347700	-8.78737400
H	1.20134200	3.32525100	-8.24365400
C	-1.48745100	5.33816200	-8.70641100
H	-3.46426600	4.96613200	-7.93013000
H	0.57304500	5.42350200	-9.33977500
H	-1.76317300	6.26961600	-9.19431500
C	0.19728900	-0.05258000	-7.86948100
C	0.38407000	-1.43936900	-7.21435000
H	0.76547400	-2.15446500	-7.95795700
H	-0.55773000	-1.84918400	-6.83264200
H	1.10416700	-1.41343400	-6.38840000
C	1.54825900	0.36118100	-8.49779100
H	1.88760400	-0.41725000	-9.19657300
H	2.33292800	0.49005400	-7.74309500
H	1.46789200	1.29159200	-9.07058200
C	-0.85868800	-0.16207700	-8.99083400
H	-0.99726900	0.79106600	-9.51334200
H	-1.83629100	-0.47339700	-8.60461700
H	-0.54550900	-0.90825700	-9.73565000
N	-2.86185000	-1.03767700	-2.85503300
H	-3.83575300	-1.27100100	-2.66941200
C	-1.90882300	-1.78748400	-2.24855600
O	-0.69912500	-1.62426200	-2.40233800
C	-2.41850200	-2.92862600	-1.33277800
C	-2.91937100	-4.16912300	-2.12402100
C	-3.26513000	-5.32323000	-1.17145800
H	-3.62795900	-6.18670100	-1.73985500
H	-4.04407800	-5.04697700	-0.45423200
H	-2.38075600	-5.64620000	-0.60616000
C	-1.86365700	-4.61508300	-3.14858300
H	-0.91498500	-4.86771600	-2.65935400

H	-1.65432800	-3.84083200	-3.89248600
H	-2.21566900	-5.50573200	-3.68064100
N	-3.37599000	-2.47088200	-0.30567300
H	-2.97835400	-2.24866600	0.59570100
C	-4.67808200	-2.13804100	-0.50734200
O	-5.21096100	-2.11578000	-1.62307400
H	-3.82854700	-3.88112800	-2.66162300
C	-5.48403100	-1.78581800	0.71566500
C	-6.66049600	-1.05834700	0.50736700
C	-5.14199600	-2.17691900	2.01494600
C	-7.46255900	-0.69422500	1.58786400
H	-6.93078100	-0.79045400	-0.50767700
C	-5.95469700	-1.81974700	3.09216600
H	-4.26025900	-2.78036600	2.20308800
C	-7.11333100	-1.07019800	2.88554300
H	-7.73914500	-0.78899900	3.72433800
C	-5.61794800	-2.30622800	4.47910800
C	-8.68682500	0.15204800	1.34519300
F	-6.02688000	-1.43578800	5.42476100
F	-6.21244000	-3.49030500	4.74330200
F	-4.28810400	-2.48757900	4.63307100
F	-8.35670400	1.46139800	1.20977800
F	-9.56687400	0.07090000	2.36330400
F	-9.32483600	-0.20968900	0.21508400
H	-1.52657000	-3.23010300	-0.77628400

2a

C	-1.00043100	1.60715900	0.13825900
C	-2.29991400	1.60298600	0.24100200
H	-2.86520600	0.67330500	0.25788200
H	-2.86566900	2.52870200	0.32535500
C	0.30744500	1.61207400	0.01480600
H	0.77620500	1.64801100	-0.96580300
C	1.27157700	1.57175000	1.14958500
O	2.47529000	1.57987500	0.97026900
O	0.64818300	1.52707900	2.34134100
C	1.40387500	1.48286500	3.60604600
C	2.25344300	0.20848900	3.66570300
H	3.04092000	0.22353200	2.91070100
H	2.71558100	0.12279600	4.65575300
H	1.62559800	-0.67522800	3.50655900
C	0.29023200	1.44286700	4.65625700
H	0.72216200	1.40700100	5.66206600
H	-0.34346400	2.33251800	4.58159800
H	-0.34006900	0.55864200	4.51677700
C	2.24828900	2.75320900	3.75755500
H	1.61714600	3.64354800	3.66031600
H	2.70852500	2.77053600	4.75208600
H	3.03746900	2.79552600	3.00527800

1a

C	-1.65574500	-0.28064200	0.00712700
C	-1.67694200	1.11605500	-0.00338800
C	-2.89226200	1.80336200	0.01748000
C	-4.06993900	1.05866400	0.04861200
C	-4.02377200	-0.34663600	0.05859100
C	-2.81371900	-1.04648000	0.03807400
C	0.50018900	0.28131900	-0.04533900
C	-0.27319300	1.56266300	-0.03803100
H	-2.90221300	2.88930500	0.00914600
H	-5.03209700	1.56165700	0.06538800
H	-4.95397500	-0.90776000	0.08293900
H	-2.77495800	-2.13057400	0.04562500
O	-0.37729400	-0.79561800	-0.01680800
O	0.19049300	2.69445400	-0.05736500
C	1.84321700	0.18324200	-0.07578300
H	2.32980500	1.15693200	-0.09500000
C	2.71442500	-0.98278300	-0.08710500
C	2.24252200	-2.31294100	-0.06390800
C	4.10785000	-0.76871700	-0.12328900
C	3.13805900	-3.37833500	-0.07693600
H	1.17621300	-2.50076600	-0.03572200
C	4.99932000	-1.83752700	-0.13627700
H	4.48474700	0.25095700	-0.14111000
C	4.51691100	-3.14824000	-0.11313900
H	2.75884200	-4.39661900	-0.05879300
H	6.06901500	-1.64903500	-0.16432600
H	5.20975700	-3.98525300	-0.12316900

5-*re*

C	0.84103100	0.26092600	-0.62599700
C	0.10003700	0.80850100	0.42766200
C	-1.20166100	0.37001600	0.69468500
C	-1.73962700	-0.62500400	-0.11411900
C	-0.98050700	-1.16622800	-1.17060100
C	0.31714100	-0.73756400	-1.44774900
C	2.27360400	1.79131100	0.27468100
C	0.94672400	1.81658600	1.06256500
H	-1.76252900	0.80638400	1.51608900
H	-2.74624200	-0.99083700	0.06391400
H	-1.41890100	-1.94397300	-1.79048600
H	0.90123900	-1.15646600	-2.26005300
O	2.08142500	0.79787600	-0.76743200
O	0.72408700	2.55001800	2.01126600
C	3.50760000	1.42537700	1.18109000
H	3.27503300	1.81861900	2.18041800
C	3.77308000	-0.06271800	1.31426100
C	3.36472600	-0.74323800	2.46729300
C	4.41816600	-0.78563200	0.30097600
C	3.59683900	-2.11305400	2.61133700

H	2.86609700	-0.19419600	3.26291400
C	4.65587400	-2.15250600	0.44312800
H	4.73414100	-0.27305300	-0.60315400
C	4.24615700	-2.82169400	1.59969300
H	3.27390500	-2.62273300	3.51536900
H	5.16005300	-2.69674800	-0.35139900
H	4.43243700	-3.88672200	1.71024400
C	4.14053200	3.19618700	-0.27409100
C	2.64417000	3.13670100	-0.39236800
H	2.27404100	3.17651400	-1.42282500
H	2.18036900	3.96709500	0.15957800
C	4.61810600	2.27789600	0.57644700
C	6.05554500	2.22610700	0.95724800
O	6.94029400	2.76698800	0.31796300
O	6.21824100	1.53221300	2.09860700
C	7.54969300	1.28462600	2.68274600
C	8.40462300	0.48199800	1.69583000
H	8.64466000	1.07198600	0.80959300
H	9.33985600	0.18194700	2.18211800
H	7.87334400	-0.42467700	1.38680600
C	7.21828400	0.44804500	3.92155200
H	8.13911800	0.17625800	4.44857900
H	6.57952900	1.01264900	4.60878400
H	6.69203700	-0.46882300	3.63813400
C	8.20345900	2.61330200	3.07695400
H	7.54231800	3.18105500	3.74103900
H	9.13708400	2.41567900	3.61607100
H	8.42581900	3.21891700	2.19706300
H	4.75511200	3.94152700	-0.76782500

5-*si*

C	0.84103100	0.26092600	0.62599700
C	0.10003700	0.80850100	-0.42766200
C	-1.20166100	0.37001600	-0.69468500
C	-1.73962700	-0.62500400	0.11411900
C	-0.98050700	-1.16622800	1.17060100
C	0.31714100	-0.73756400	1.44774900
C	2.27360400	1.79131100	-0.27468100
C	0.94672400	1.81658600	-1.06256500
H	-1.76252900	0.80638400	-1.51608900
H	-2.74624200	-0.99083700	-0.06391400
H	-1.41890100	-1.94397300	1.79048600
H	0.90123900	-1.15646600	2.26005300
O	2.08142500	0.79787600	0.76743200
O	0.72408700	2.55001800	-2.01126600
C	3.50760000	1.42537700	-1.18109000
H	3.27503300	1.81861900	-2.18041800
C	3.77308000	-0.06271800	-1.31426100
C	3.36472600	-0.74323800	-2.46729300
C	4.41816600	-0.78563200	-0.30097600

C	3.59683900	-2.11305400	-2.61133700
H	2.86609700	-0.19419600	-3.26291400
C	4.65587400	-2.15250600	-0.44312800
H	4.73414100	-0.27305300	0.60315400
C	4.24615700	-2.82169400	-1.59969300
H	3.27390500	-2.62273300	-3.51536900
H	5.16005300	-2.69674800	0.35139900
H	4.43243700	-3.88672200	-1.71024400
C	4.14053200	3.19618700	0.27409100
C	2.64417000	3.13670100	0.39236800
H	2.27404100	3.17651400	1.42282500
H	2.18036900	3.96709500	-0.15957800
C	4.61810600	2.27789600	-0.57644700
C	6.05554500	2.22610700	-0.95724800
O	6.94029400	2.76698800	-0.31796300
O	6.21824100	1.53221300	-2.09860700
C	7.54969300	1.28462600	-2.68274600
C	8.40462300	0.48199800	-1.69583000
H	8.64466000	1.07198600	-0.80959300
H	9.33985600	0.18194700	-2.18211800
H	7.87334400	-0.42467700	-1.38680600
C	7.21828400	0.44804500	-3.92155200
H	8.13911800	0.17625800	-4.44857900
H	6.57952900	1.01264900	-4.60878400
H	6.69203700	-0.46882300	-3.63813400
C	8.20345900	2.61330200	-3.07695400
H	7.54231800	3.18105500	-3.74103900
H	9.13708400	2.41567900	-3.61607100
H	8.42581900	3.21891700	-2.19706300
H	4.75511200	3.94152700	0.76782500

6-*re*

C	1.25003200	0.69842800	-0.99964400
C	0.11365500	0.58745400	-0.19243500
C	-0.99967000	-0.14399400	-0.61574200
C	-0.95035500	-0.75711200	-1.86416500
C	0.19965100	-0.63341000	-2.66725300
C	1.31777100	0.09176900	-2.25394700
C	1.82753200	1.87702400	0.87642400
C	0.38127800	1.34127000	1.03177400
H	-1.87243900	-0.22210000	0.02621100
H	-1.79616100	-1.33474600	-2.22462700
H	0.22018400	-1.11983100	-3.63910000
H	2.20434600	0.18369200	-2.87226300
O	2.24939400	1.43939600	-0.45172600
O	-0.30937100	1.49957200	2.02231500
C	2.75141700	1.31907600	2.01641100
H	2.09304200	1.32276600	2.89333200
C	3.28097800	-0.08532100	1.82574200
C	2.77844100	-1.13111600	2.61229100

C	4.27526700	-0.38084300	0.88011800
C	3.25467700	-2.43526300	2.46522700
H	2.00570000	-0.91956900	3.34778900
C	4.75361800	-1.68245400	0.73127400
H	4.66873000	0.40972800	0.24831400
C	4.24606900	-2.71463600	1.52421000
H	2.85201700	-3.22987200	3.08782300
H	5.52432800	-1.89134600	-0.00623100
H	4.62158600	-3.72791400	1.40870100
C	3.12327500	3.66221600	1.71624400
C	3.80328000	2.43682400	2.24863000
H	4.07513400	2.52238100	3.30783100
H	4.73968700	2.25155200	1.70415600
C	2.03722300	3.37372600	0.98347800
C	1.22826300	4.41969900	0.30457600
O	1.46734600	5.60988300	0.39710700
O	0.22371400	3.86974700	-0.40280400
C	-0.74585900	4.69699200	-1.15111600
C	-1.50272100	5.61208800	-0.18251200
H	-0.84040100	6.35959700	0.25688800
H	-2.30617700	6.12694400	-0.72159700
H	-1.95532000	5.02180000	0.62153500
C	-1.68483500	3.65158000	-1.75739900
H	-2.45714200	4.14709700	-2.35547900
H	-1.13345200	2.96120000	-2.40337800
H	-2.17417800	3.06879600	-0.97081300
C	-0.01765000	5.47926400	-2.24934800
H	0.54836600	4.79580300	-2.89185800
H	-0.75223400	6.00158700	-2.87288000
H	0.66687200	6.21451500	-1.82367200
H	3.47539600	4.67574300	1.88062800

6-*si*

C	1.25003200	0.69842800	0.99964400
C	0.11365500	0.58745400	0.19243500
C	-0.99967000	-0.14399400	0.61574200
C	-0.95035500	-0.75711200	1.86416500
C	0.19965100	-0.63341000	2.66725300
C	1.31777100	0.09176900	2.25394700
C	1.82753200	1.87702400	-0.87642400
C	0.38127800	1.34127000	-1.03177400
H	-1.87243900	-0.22210000	-0.02621100
H	-1.79616100	-1.33474600	2.22462700
H	0.22018400	-1.11983100	3.63910000
H	2.20434600	0.18369200	2.87226300
O	2.24939400	1.43939600	0.45172600
O	-0.30937100	1.49957200	-2.02231500
C	2.75141700	1.31907600	-2.01641100
H	2.09304200	1.32276600	-2.89333200
C	3.28097800	-0.08532100	-1.82574200

C	2.77844100	-1.13111600	-2.61229100
C	4.27526700	-0.38084300	-0.88011800
C	3.25467700	-2.43526300	-2.46522700
H	2.00570000	-0.91956900	-3.34778900
C	4.75361800	-1.68245400	-0.73127400
H	4.66873000	0.40972800	-0.24831400
C	4.24606900	-2.71463600	-1.52421000
H	2.85201700	-3.22987200	-3.08782300
H	5.52432800	-1.89134600	0.00623100
H	4.62158600	-3.72791400	-1.40870100
C	3.12327500	3.66221600	-1.71624400
C	3.80328000	2.43682400	-2.24863000
H	4.07513400	2.52238100	-3.30783100
H	4.73968700	2.25155200	-1.70415600
C	2.03722300	3.37372600	-0.98347800
C	1.22826300	4.41969900	-0.30457600
O	1.46734600	5.60988300	-0.39710700
O	0.22371400	3.86974700	0.40280400
C	-0.74585900	4.69699200	1.15111600
C	-1.50272100	5.61208800	0.18251200
H	-0.84040100	6.35959700	-0.25688800
H	-2.30617700	6.12694400	0.72159700
H	-1.95532000	5.02180000	-0.62153500
C	-1.68483500	3.65158000	1.75739900
H	-2.45714200	4.14709700	2.35547900
H	-1.13345200	2.96120000	2.40337800
H	-2.17417800	3.06879600	0.97081300
C	-0.01765000	5.47926400	2.24934800
H	0.54836600	4.79580300	2.89185800
H	-0.75223400	6.00158700	2.87288000
H	0.66687200	6.21451500	1.82367200
H	3.47539600	4.67574300	-1.88062800

9-int

P	0.13734100	0.18316300	1.78570400
C	1.12683400	-0.00118000	0.19507500
H	2.20072400	0.09195500	0.38339800
H	0.84282600	0.87107200	-0.40628600
C	0.87906500	-1.29410300	-0.59250700
H	1.10691100	-2.15648700	0.04081800
C	1.79797600	-1.43080200	-1.84323700
H	1.51925200	-2.37947200	-2.31158700
C	1.62917500	-0.30857400	-2.86527800
H	1.99368100	0.64414400	-2.46737800
H	0.57686100	-0.19869100	-3.14932300
H	2.20213000	-0.53276000	-3.77042600
C	0.91598700	-1.03497700	2.93926800
C	0.21420300	-1.30595900	4.12749500
C	2.12022600	-1.71834000	2.70804700
C	0.70976200	-2.21085800	5.06525500

H	-0.73103500	-0.80146700	4.31674000
C	2.60903300	-2.64007400	3.63775100
H	2.68666900	-1.54883900	1.79813900
C	1.90901600	-2.88406900	4.81959200
H	0.15478400	-2.39955000	5.98071300
H	3.53169000	-3.17216500	3.42576000
H	2.29020600	-3.60138400	5.54165400
C	0.86508100	1.79593300	2.35851500
C	0.09160900	2.95625900	2.19277100
C	2.14141700	1.91856000	2.93042500
C	0.58602400	4.20793300	2.56875100
H	-0.91012600	2.87840900	1.77652400
C	2.63290000	3.16680300	3.31517400
H	2.75105800	1.03275900	3.08556100
C	1.85848200	4.31525700	3.13207500
H	-0.02796100	5.09497900	2.43388500
H	3.62197900	3.24273800	3.75993800
H	2.24224900	5.28623400	3.43412400
O	3.15773200	-1.46458600	-1.41174200
Si	4.35678700	-2.63934400	-1.39612900
C	3.90651900	-4.03422400	-0.19341800
C	2.63943300	-4.65247300	-0.24546100
C	4.81589300	-4.50709400	0.77353300
C	2.30359200	-5.69640900	0.61917300
H	1.87848800	-4.31440000	-0.94274900
C	4.48778800	-5.55944500	1.63221800
H	5.79352300	-4.04401700	0.87134700
C	3.22947600	-6.15924600	1.55532600
H	1.31392900	-6.14099000	0.55748600
H	5.21466900	-5.90686000	2.36286000
H	2.97080600	-6.97587700	2.22481700
C	5.84996700	-1.65876100	-0.76386100
C	5.72197900	-0.27984200	-0.50851300
C	7.11646300	-2.23780300	-0.54415600
C	6.79968100	0.48217400	-0.05243100
H	4.76240500	0.19943900	-0.67512000
C	8.19787700	-1.48159900	-0.08694800
H	7.27321300	-3.29717400	-0.72925400
C	8.04197900	-0.11695800	0.16125400
H	6.66851100	1.54519900	0.13537300
H	9.16141000	-1.95891100	0.07365500
H	8.88232200	0.47413800	0.51654200
C	4.69652100	-3.34112800	-3.16062000
C	3.44792400	-4.03976400	-3.74401200
H	3.68933000	-4.47487600	-4.72490100
H	2.61431900	-3.34609500	-3.90047400
H	3.09251300	-4.85591100	-3.10488700
C	5.83837300	-4.38094000	-3.09642300
H	5.99960200	-4.81921100	-4.09202800
H	5.61022300	-5.20445700	-2.40915200

H	6.78691600	-3.92891700	-2.78691000
C	5.12168000	-2.19438300	-4.10308800
H	6.03134700	-1.69481900	-3.75091700
H	4.34027500	-1.43165900	-4.19771400
H	5.32541500	-2.58629900	-5.11021700
N	-0.53010600	-1.41576600	-0.97596500
H	-1.09609900	-0.57537600	-1.01058700
C	-1.08086300	-2.62885700	-1.25998300
O	-0.42517800	-3.67029500	-1.25694000
C	-2.59881200	-2.68547000	-1.51566000
H	-2.74476400	-3.57394400	-2.13279800
C	-3.39331900	-2.84884300	-0.18608100
C	-4.88406600	-3.07991500	-0.47680200
H	-5.44846100	-3.16047700	0.45987200
H	-5.03159200	-4.01275600	-1.03483700
H	-5.31959200	-2.26654800	-1.06583900
C	-2.83760300	-3.98524300	0.68691000
H	-3.44960600	-4.09225700	1.59013900
H	-1.80502600	-3.80713700	0.99754300
H	-2.85936000	-4.93974300	0.14823400
N	-3.10924100	-1.56536400	-2.30350300
H	-3.11101700	-0.63553200	-1.89648000
C	-3.55214400	-1.74421500	-3.58421700
O	-3.45864200	-2.80780400	-4.19103100
C	-3.99666800	1.26703900	1.03565500
C	-4.38554100	0.21328800	1.69418600
H	-3.77898600	-0.19469200	2.50021200
H	-5.31515000	-0.29892800	1.45481500
C	-3.61152100	2.35345300	0.40321600
H	-3.91219600	3.33627200	0.75767500
C	-2.72382300	2.32042700	-0.78729900
O	-2.38688900	3.57150000	-1.11006400
O	-2.35333400	1.30479100	-1.36925100
C	-1.50535300	3.92920100	-2.24682500
H	-3.29032500	-1.90664600	0.37127500
C	-1.44545700	5.45504700	-2.13954100
H	-2.44715800	5.88825900	-2.22273400
H	-0.82378400	5.86140500	-2.94388000
H	-1.01374700	5.75900900	-1.18055900
C	-2.15454600	3.49713400	-3.56319400
H	-1.55475600	3.87058500	-4.40066400
H	-3.16115800	3.91617100	-3.65346000
H	-2.21364000	2.40996100	-3.64005600
C	-0.11983500	3.31272400	-2.04498100
H	-0.14848300	2.22819500	-2.15947100
H	0.26985100	3.55862100	-1.05134600
H	0.57017900	3.72004900	-2.79217500
C	-4.20215000	-0.55356400	-4.24898400
C	-4.28179600	-0.57426900	-5.64575300
C	-4.76526100	0.51850500	-3.54907700

C	-4.88550100	0.47764300	-6.33351100
H	-3.86243200	-1.42363100	-6.17314600
C	-5.36976400	1.56875100	-4.24212900
H	-4.74134700	0.54961300	-2.46693800
C	-5.42936700	1.55722400	-5.63597600
H	-5.88324900	2.38264400	-6.17250900
C	-4.99827700	0.42435600	-7.83623900
C	-5.99409600	2.71221200	-3.48800200
F	-5.06939200	1.66231000	-8.37456600
F	-3.94420900	-0.20487600	-8.39444300
F	-6.10942700	-0.23982300	-8.22327400
F	-5.48177600	2.83532700	-2.24023800
F	-7.32870200	2.56381000	-3.35356000
F	-5.79095300	3.89179100	-4.12119500

10-ts

P	0.27096700	0.25736200	2.27724700
C	0.89938600	0.17883500	0.53487300
H	1.98503000	0.30785900	0.51858200
H	0.47096100	1.06691700	0.05352500
C	0.55645800	-1.09343000	-0.25671900
H	0.86058400	-1.97716100	0.30822800
C	1.33239800	-1.16247700	-1.60535800
H	1.00185300	-2.09015700	-2.08349500
C	1.05073800	0.00930400	-2.54416700
H	1.47107500	0.93958200	-2.14744800
H	-0.02602300	0.14335300	-2.69021400
H	1.50568000	-0.17093400	-3.52284900
C	0.99671200	-1.16657700	3.19422500
C	0.41539800	-1.49347800	4.43197800
C	2.08435600	-1.92671200	2.73435900
C	0.91298600	-2.54982400	5.19380700
H	-0.42622300	-0.91519000	4.80577600
C	2.57266600	-2.99192700	3.49322200
H	2.56002700	-1.70061200	1.78607000
C	1.98965000	-3.30424500	4.72239800
H	0.45549000	-2.78761700	6.15038600
H	3.40361900	-3.57790500	3.11248600
H	2.37080700	-4.13508400	5.30990200
C	1.15280200	1.71626300	2.98501700
C	0.44507000	2.61135800	3.80152500
C	2.52637300	1.92756100	2.78253400
C	1.09102800	3.69943400	4.39204900
H	-0.61526200	2.45565400	3.97857300
C	3.17069700	3.01523800	3.37172700
H	3.10643500	1.23815500	2.17566200
C	2.45384200	3.90519700	4.17528200
H	0.52776700	4.38438700	5.01993100
H	4.23423600	3.16472000	3.20630200
H	2.95749600	4.75230500	4.63296900
O	2.73106600	-1.20988500	-1.32330600
Si	3.94717700	-2.33949400	-1.58054100
C	3.69106100	-3.83928200	-0.44841400
C	2.42911800	-4.46405800	-0.35870300
C	4.73897500	-4.39013600	0.31576600
C	2.22920400	-5.58929800	0.44389300
H	1.56740900	-4.07116000	-0.89024600
C	4.54660300	-5.52322000	1.11073100
H	5.72099400	-3.92653000	0.30399100
C	3.28979500	-6.12890300	1.17348200
H	1.23951300	-6.03433700	0.49694300
H	5.37825500	-5.92970000	1.68165600
H	3.13735100	-7.00985000	1.79219100
C	5.49243400	-1.35995400	-1.08605800
C	5.37513000	0.00037600	-0.74064100
C	6.78761900	-1.91576000	-1.06903800
C	6.48962200	0.76537900	-0.39043600
H	4.39405000	0.46511900	-0.75690500
C	7.90631100	-1.15702600	-0.71787700
H	6.93890700	-2.95891200	-1.33298800
C	7.76028900	0.18818100	-0.37613200
H	6.36585300	1.81467100	-0.13278300
H	8.89125400	-1.61717300	-0.71414100
H	8.62963700	0.78155600	-0.10449700
C	4.05243500	-2.89504900	-3.42481800
C	2.73464200	-3.55126300	-3.89394300
H	2.84775500	-3.91766600	-4.92460200
H	1.89463900	-2.84837000	-3.89717400
H	2.45446000	-4.40992000	-3.27314700
C	5.18642200	-3.93340100	-3.58896400
H	5.22598200	-4.27620200	-4.63293000
H	5.03165500	-4.81740500	-2.95929400
H	6.17000000	-3.51196900	-3.35296000
C	4.36175500	-1.67751200	-4.32289900
H	5.31310500	-1.20460600	-4.05386000
H	3.58062100	-0.91122700	-4.25972100
H	4.43084900	-1.99019600	-5.37490100
N	-0.87563500	-1.22717900	-0.48981400
H	-1.44541700	-0.38892600	-0.61538600
C	-1.43224800	-2.45666800	-0.68998600
O	-0.80366700	-3.50638800	-0.57059100
C	-2.93563800	-2.47781200	-1.04465600
H	-3.08910800	-3.46287800	-1.49197000
C	-3.84300400	-2.32619600	0.20256600
C	-5.31934100	-2.53858800	-0.16768300
H	-5.96031500	-2.38125000	0.70750100
H	-5.48658100	-3.56277000	-0.52460500
H	-5.64849200	-1.85344200	-0.95498400
C	-3.42782500	-3.29532200	1.32034700
H	-4.10081800	-3.18715800	2.17889800

H	-2.40538500	-3.11760200	1.66607000
H	-3.48217900	-4.33570500	0.97846500
N	-3.29429400	-1.48740000	-2.06036700
H	-3.46033700	-0.53456600	-1.74800900
C	-2.96537400	-1.70605200	-3.37001600
O	-2.36971000	-2.70850800	-3.75497300
C	-1.92307300	1.04310700	2.06844800
C	-2.69257200	0.23007300	2.77606200
H	-2.33129100	-0.66980400	3.26102700
H	-3.75311000	0.46090900	2.87052600
C	-1.92697800	2.17280800	1.28791600
H	-1.60266000	3.12156500	1.70413200
C	-2.29878200	2.16981300	-0.10343700
O	-2.29246600	3.43704400	-0.58607000
O	-2.59631400	1.17512400	-0.79144500
C	-2.77246500	3.79820500	-1.92322500
H	-3.71984300	-1.29922500	0.57500500
C	-2.55158800	5.31446900	-1.95761200
H	-3.10848100	5.80317100	-1.15166400
H	-2.89266700	5.72408400	-2.91435700
H	-1.48989200	5.55335500	-1.83599900
C	-4.26417600	3.47129000	-2.03944600
H	-4.65082800	3.81103000	-3.00494000
H	-4.82559400	3.97915200	-1.24756200
H	-4.43724200	2.39739100	-1.95492300
C	-1.93274400	3.11462700	-3.00896700
H	-2.09158600	2.03610400	-3.01583500
H	-0.86765700	3.31331200	-2.84555000
H	-2.20534800	3.51640700	-3.99145700
C	-3.40823100	-0.65717400	-4.35928600
C	-2.68688500	-0.55431200	-5.55244100
C	-4.53442300	0.15128700	-4.16453400
C	-3.06449500	0.37179900	-6.52509100
H	-1.83494900	-1.20811500	-5.70218800
C	-4.91723000	1.06419800	-5.14773700
H	-5.12744700	0.05648200	-3.26239400
C	-4.18021200	1.18513500	-6.32760600
H	-4.47142000	1.90609100	-7.08359000
C	-2.29705300	0.44994900	-7.82040000
C	-6.14503000	1.91942500	-4.97702200
F	-2.41558100	1.66553700	-8.39877800
F	-0.98176500	0.21174800	-7.63502400
F	-2.74283400	-0.45957200	-8.71432900
F	-6.68079600	1.80845000	-3.74393900
F	-7.10632700	1.59299400	-5.86741100
F	-5.86121600	3.23039400	-5.17906300

11-int

P	0.55178600	0.41964400	2.13188900
C	1.10456900	0.18309600	0.39371200
H	2.19708700	0.21999600	0.36448600
H	0.72941900	1.08251400	-0.10733500
C	0.67009800	-1.09291700	-0.34928700
H	0.92296200	-1.97465700	0.24490400
C	1.47418500	-1.23542800	-1.67925000
H	1.12149300	-2.16707400	-2.12994800
C	1.25165500	-0.08858800	-2.66238200
H	1.68214800	0.84624700	-2.28728800
H	0.18324100	0.06140700	-2.84584600
H	1.73119600	-0.31416700	-3.61960700
C	1.07632000	-0.96905300	3.18578600
C	0.52463200	-1.07710300	4.47586600
C	2.05237200	-1.88939400	2.77257100
C	0.93468000	-2.10137000	5.32712100
H	-0.22839200	-0.36983900	4.80966300
C	2.45812100	-2.91250700	3.63088900
H	2.50278900	-1.82279100	1.78863800
C	1.89881900	-3.02036900	4.90484100
H	0.50014300	-2.18151900	6.31949100
H	3.20393800	-3.62446200	3.29184600
H	2.21385800	-3.81958900	5.57000600
C	1.42130500	1.89166100	2.77329200
C	0.74999300	2.78718800	3.62038800
C	2.78542700	2.08703100	2.50538800
C	1.43143000	3.87011500	4.17621200
H	-0.30526300	2.64694400	3.83249000
C	3.46121700	3.17137300	3.06429700
H	3.33216100	1.39681300	1.86959300
C	2.78548800	4.06476500	3.89810100
H	0.90092500	4.56248900	4.82344000
H	4.51582000	3.31602500	2.84824200
H	3.31387600	4.90960400	4.33084100
O	2.86366900	-1.32041900	-1.35919300
Si	4.04208700	-2.50636400	-1.53124900
C	3.70685500	-3.92665100	-0.31826000
C	2.42674500	-4.51659100	-0.24321100
C	4.71060400	-4.45516600	0.51768500
C	2.16986100	-5.59074300	0.61177100
H	1.59541300	-4.13475000	-0.82878700
C	4.46091200	-5.53772400	1.36571900
H	5.70351400	-4.01514200	0.51839200
C	3.18878100	-6.11262400	1.41074300
H	1.16915400	-6.01232900	0.64870800
H	5.26052700	-5.93029300	1.98968900
H	2.99244700	-6.95618400	2.06800500
C	5.61273100	-1.55697700	-1.05717300
C	5.54356500	-0.17266300	-0.80706200
C	6.88265800	-2.16159500	-0.96538300
C	6.67878800	0.56890300	-0.47337600
H	4.58427400	0.32936600	-0.88909000

C	8.02187900	-1.42659100	-0.62982800
H	6.99753200	-3.22460700	-1.15925000
C	7.92289000	-0.05700700	-0.38060500
H	6.59297800	1.63781500	-0.29209100
H	8.98630000	-1.92425400	-0.56675300
H	8.80843500	0.51776700	-0.12176800
C	4.16294400	-3.17029700	-3.33720900
C	2.83189700	-3.80243900	-3.80197700
H	2.95597200	-4.23117500	-4.80685300
H	2.02013800	-3.07025100	-3.86747500
H	2.50384900	-4.61300500	-3.14147400
C	5.26015900	-4.25749000	-3.41245900
H	5.31147700	-4.66095700	-4.43389000
H	5.05646400	-5.09763000	-2.73822300
H	6.25329400	-3.85975400	-3.17490400
C	4.53916300	-2.01816500	-4.29412400
H	5.50345900	-1.56843500	-4.03168500
H	3.78799900	-1.22008800	-4.29018800
H	4.61584200	-2.39246000	-5.32507200
N	-0.75997300	-1.16412200	-0.62126300
H	-1.35683600	-0.33033300	-0.60351000
C	-1.32935800	-2.38293200	-0.85296600
O	-0.70304300	-3.43863100	-0.75608400
C	-2.82188000	-2.39550300	-1.25020900
H	-2.94491100	-3.35552400	-1.75758500
C	-3.77764400	-2.33344800	-0.03174400
C	-5.23620100	-2.52232400	-0.47793200
H	-5.91148600	-2.44659100	0.38224000
H	-5.38036200	-3.51186000	-0.93074700
H	-5.54099400	-1.77094100	-1.21236100
C	-3.40622600	-3.38621300	1.02405300
H	-4.10404100	-3.33371500	1.86792100
H	-2.39331400	-3.24699300	1.41257200
H	-3.45998900	-4.39819200	0.60468300
N	-3.15735800	-1.35524000	-2.22231000
H	-3.34538900	-0.42388700	-1.85506400
C	-2.80104700	-1.52488500	-3.52985700
O	-2.18282000	-2.50916200	-3.93201400
C	-1.24221200	0.74670100	2.07941600
C	-2.07599300	-0.23213400	2.50272900
H	-1.72913700	-1.17968200	2.90011900
H	-3.14832200	-0.09981800	2.39654300
C	-1.54097700	1.94476000	1.33737200
H	-1.08835300	2.88612400	1.62363500
C	-2.38807200	1.97274800	0.20910000
O	-2.66596200	3.26919800	-0.15153500
O	-2.81758700	0.98673400	-0.44970800
C	-3.40565900	3.63059100	-1.34889000
H	-3.67453700	-1.33469800	0.41087400
C	-3.44469000	5.16266500	-1.27676900
H	-3.95258200	5.49032200	-0.36365000
H	-3.98144400	5.57097200	-2.14013700
H	-2.42958400	5.57362200	-1.27028900
C	-4.83449900	3.07169500	-1.29367200
H	-5.41366800	3.42974100	-2.15062400
H	-5.33123500	3.40959100	-0.37717300
H	-4.82791500	1.98100000	-1.29623900
C	-2.64425000	3.18680400	-2.60462400
H	-2.54372100	2.10120600	-2.63678100
H	-1.64191200	3.63023100	-2.61273600
H	-3.17495200	3.51715700	-3.50356900
C	-3.23738800	-0.46217300	-4.50805900
C	-2.52418600	-0.37412300	-5.70817900
C	-4.34876100	0.36431000	-4.30800300
C	-2.89877500	0.54736300	-6.68519400
H	-1.68385900	-1.04208300	-5.86022100
C	-4.72822300	1.27469500	-5.29627000
H	-4.93552600	0.28589600	-3.40014300
C	-4.00370900	1.37492400	-6.48498600
H	-4.30001200	2.08556400	-7.24837200
C	-2.14504200	0.60187900	-7.98916300
C	-5.89942700	2.19695100	-5.08589900
F	-2.25902900	1.81052000	-8.58292100
F	-0.82945700	0.35479300	-7.81484900
F	-2.60681500	-0.31556900	-8.86754500
F	-6.69241800	1.79553400	-4.07206100
F	-6.66504800	2.29277600	-6.19412700
F	-5.48738700	3.45827400	-4.79349000

12-int

P	0.89705500	1.72551100	-0.08908600
C	1.71238300	1.32891400	-1.70107800
H	2.77741700	1.19727500	-1.48832700
H	1.59351900	2.23932800	-2.29193500
C	1.24037400	0.07621500	-2.45328700
H	1.44732200	-0.81609500	-1.85563000
C	2.03925800	-0.11105200	-3.77903700
H	1.67234200	-1.04890300	-4.20751200
C	1.83973800	1.00714400	-4.79877200
H	2.25290200	1.95581000	-4.44097200
H	0.77647100	1.14376100	-5.02074900
H	2.35049300	0.75178800	-5.73250400
C	1.19113300	0.27771800	0.99871800
C	2.04860300	0.37261400	2.10578000
C	0.60468000	-0.96422900	0.69364600
C	2.31825400	-0.75262500	2.88711400
H	2.50674800	1.32087000	2.36285200
C	0.88179200	-2.08466000	1.47437000
H	-0.07888500	-1.05614500	-0.14270300
C	1.73991000	-1.98152200	2.57133500

H	2.98191600	-0.66347400	3.74251900
H	0.43184400	-3.03872100	1.21820600
H	1.95534900	-2.85721600	3.17716800
C	1.76264600	3.11799100	0.70285000
C	1.04722100	3.94551500	1.57960700
C	3.13175300	3.33949400	0.49837700
C	1.69777100	4.98653300	2.24132400
H	-0.01473000	3.78076700	1.73314800
C	3.77520600	4.38373000	1.16321500
H	3.70332300	2.71007200	-0.17614200
C	3.06070300	5.20824300	2.03428500
H	1.13593000	5.62802000	2.91430500
H	4.83507500	4.55311800	0.99584400
H	3.56438900	6.02219200	2.54829000
O	3.42537700	-0.20863000	-3.45747900
Si	4.50116400	-1.49846200	-3.40363700
C	3.88959200	-2.83930200	-2.20853400
C	2.63821600	-3.46484600	-2.39298200
C	4.65812800	-3.25252400	-1.10258800
C	2.18805900	-4.46438900	-1.52799600
H	1.97770300	-3.16243300	-3.19913100
C	4.21642300	-4.25801500	-0.23977800
H	5.61455700	-2.77904800	-0.90206600
C	2.98153600	-4.87210300	-0.45410300
H	1.21632500	-4.92006200	-1.69926500
H	4.83717600	-4.55875600	0.60087200
H	2.63821700	-5.65889500	0.21345700
C	6.06840700	-0.66691000	-2.74261000
C	6.10191400	0.73106300	-2.57540000
C	7.24278600	-1.38128400	-2.43120700
C	7.24680800	1.38354900	-2.11287300
H	5.21887900	1.31260200	-2.82281900
C	8.39002800	-0.73481500	-1.96570400
H	7.27132100	-2.46177000	-2.54782300
C	8.39449600	0.65159100	-1.80379500
H	7.24429400	2.46505700	-1.99896000
H	9.27966700	-1.31425500	-1.73186900
H	9.28691400	1.15725700	-1.44382800
C	4.81339800	-2.20567700	-5.16857000
C	3.52329500	-2.77129800	-5.80304200
H	3.74661900	-3.17309100	-6.80210800
H	2.74903800	-2.00658700	-5.93342100
H	3.09672500	-3.59009400	-5.21340100
C	5.85589000	-3.34391700	-5.09830800
H	6.01779300	-3.76620900	-6.10065700
H	5.52798100	-4.16270000	-4.44643600
H	6.82737400	-2.98780200	-4.73793500
C	5.36071300	-1.07827600	-6.07219000
H	6.30538100	-0.67278300	-5.69257100
H	4.65288000	-0.24576100	-6.15944300

H	5.54705900	-1.46262700	-7.08546900
N	-0.19807500	0.11760400	-2.69917500
H	-0.68015500	1.01277100	-2.62161900
C	-0.90718000	-1.01183100	-2.97420100
O	-0.37990000	-2.11951100	-3.07550400
C	-2.43832800	-0.86009300	-3.09246600
H	-2.72537300	-1.52989300	-3.90716500
C	-3.16796100	-1.29929400	-1.78664500
C	-4.69234000	-1.18299400	-1.95218900
H	-5.19639200	-1.48669900	-1.02777400
H	-5.04568400	-1.83966000	-2.75720400
H	-5.01144000	-0.16426100	-2.19033800
C	-2.80437500	-2.73135300	-1.36291400
H	-3.33324800	-2.98597800	-0.43709900
H	-1.73285900	-2.86305500	-1.20056500
H	-3.10589100	-3.45248200	-2.13223700
N	-2.86504900	0.47758000	-3.48907400
H	-2.88056700	1.21041300	-2.78701200
C	-3.01138900	0.81559900	-4.80780500
O	-2.70924300	0.05911500	-5.72628900
C	-0.91182400	2.04417900	-0.19255800
C	-1.65839600	1.45768900	0.78910600
H	-1.25246900	0.79058200	1.53682600
H	-2.72757800	1.65152500	0.83473900
C	-1.42113300	2.85094600	-1.26877400
H	-2.50109600	2.98039800	-1.29927400
C	-0.63490500	3.83290600	-1.92612500
O	-1.35570100	4.60771500	-2.79444800
O	0.59575600	3.98413100	-1.76496700
C	-0.77948300	5.81786600	-3.38344300
H	-2.85161900	-0.61208100	-0.98614100
C	-0.37845400	6.80085500	-2.27439100
H	-1.24274100	7.02830000	-1.63993000
H	-0.02598700	7.73942700	-2.71794900
H	0.41690400	6.38461300	-1.65382600
C	-1.93820400	6.39287300	-4.20571000
H	-1.67045700	7.38350000	-4.58973600
H	-2.83758000	6.48530600	-3.58974400
H	-2.16922800	5.75082500	-5.06134400
C	0.39918500	5.47958800	-4.30689600
H	0.10332300	4.70501800	-5.02355100
H	1.25737300	5.12329100	-3.73690600
H	0.69009900	6.37099700	-4.87577700
C	-3.61761500	2.16640200	-5.09065500
C	-3.40114300	2.71062300	-6.36209300
C	-4.44378300	2.84208500	-4.18812700
C	-3.98899700	3.92264200	-6.71425000
H	-2.78546800	2.16067200	-7.06444900
C	-5.04611200	4.04847700	-4.55325400
H	-4.66229000	2.42011300	-3.21356000

C	-4.82111100	4.59437200	-5.81478300
H	-5.29651700	5.52560000	-6.10048500
C	-3.67908100	4.56138500	-8.04254200
C	-5.90509100	4.78536700	-3.55906500
F	-2.67314400	5.46083800	-7.92334700
F	-3.29725300	3.65725000	-8.96569400
F	-4.74484900	5.22926400	-8.53594400
F	-5.16618000	5.59619800	-2.76595400
F	-6.56359400	3.93483400	-2.74110600
F	-6.82491400	5.56226100	-4.16901500

13-int

P	0.56146800	1.98587500	-0.18234000
C	0.75985900	1.55030900	-1.97968200
H	1.71818900	1.98567900	-2.25639600
H	0.00961500	2.14877600	-2.50365600
C	0.72000600	0.08051200	-2.42910800
H	1.12612900	-0.57868400	-1.65469400
C	1.62158400	-0.12168600	-3.68820700
H	1.50216600	-1.16862300	-3.97106800
C	1.24201400	0.75720200	-4.87748800
H	1.35818700	1.82182900	-4.64694900
H	0.21034900	0.56192500	-5.18703400
H	1.89384500	0.52854300	-5.72617700
C	0.12608700	0.45219200	0.72876400
C	1.03863700	-0.11731100	1.63118000
C	-1.10779300	-0.18671000	0.50625200
C	0.72217300	-1.29769000	2.30554300
H	1.99918700	0.35188000	1.80780500
C	-1.41889500	-1.36029700	1.19242100
H	-1.82691400	0.22995300	-0.19115000
C	-0.50782200	-1.91818500	2.09214500
H	1.44211400	-1.72834600	2.99504400
H	-2.37800500	-1.84076100	1.02103400
H	-0.75638400	-2.83423700	2.62120100
C	2.14058400	2.53339000	0.53897900
C	2.10678900	3.33291300	1.69063300
C	3.37047800	2.12840300	0.00415000
C	3.29973700	3.72536100	2.29803900
H	1.15479600	3.65370300	2.10257300
C	4.55932800	2.52737600	0.61595500
H	3.41434500	1.51713500	-0.89044800
C	4.52535500	3.32431400	1.76219600
H	3.26923200	4.35118000	3.18564100
H	5.50752300	2.22046300	0.18498000
H	5.45290500	3.63621900	2.23468600
O	2.97795400	0.14244600	-3.31726500
Si	4.28950000	-0.89231700	-3.10767700
C	4.01295800	-1.98035500	-1.57743200
C	2.88617800	-2.82688900	-1.49137400
C	4.89756100	-1.97353700	-0.48132200
C	2.66881700	-3.63583900	-0.37408900
H	2.14198900	-2.84909000	-2.28223100
C	4.68789100	-2.78774800	0.63436100
H	5.76305100	-1.31801000	-0.48755100
C	3.57405700	-3.62644800	0.68861600
H	1.78729500	-4.27053000	-0.33823800
H	5.39417700	-2.76293300	1.46087000
H	3.41014700	-4.26387800	1.55420500
C	5.70571500	0.33578300	-2.83167400
C	5.49688800	1.70743300	-3.07553600
C	6.99908600	-0.05662400	-2.42954300
C	6.52284100	2.64243400	-2.91626000
H	4.51530000	2.04506800	-3.39324800
C	8.02842800	0.87329200	-2.26705900
H	7.21689500	-1.10401700	-2.23711400
C	7.79220200	2.22809700	-2.50919100
H	6.32850100	3.69417600	-3.11093500
H	9.01433400	0.53871300	-1.95396000
H	8.59223300	2.95361900	-2.38532800
C	4.65308900	-1.95520700	-4.67715800
C	3.52386700	-2.95733900	-5.00400000
H	3.80487800	-3.55494800	-5.88352200
H	2.57589700	-2.46429200	-5.24422500
H	3.34038300	-3.65723300	-4.18175600
C	5.95106100	-2.76277300	-4.44149200
H	6.15678600	-3.39746900	-5.31543000
H	5.87360500	-3.42327200	-3.56908700
H	6.82117200	-2.11207800	-4.30256500
C	4.86327300	-1.02135700	-5.88953900
H	5.67765100	-0.30781700	-5.71999300
H	3.96021400	-0.44660700	-6.12431800
H	5.11831800	-1.61182000	-6.78137700
N	-0.65430500	-0.33358900	-2.71604400
H	-1.39273400	0.36060600	-2.66907600
C	-0.97741500	-1.61798600	-3.02794200
O	-0.15979500	-2.53835300	-3.02621100
C	-2.45084700	-1.89952300	-3.41059000
H	-2.37658300	-2.58015400	-4.26343800
C	-3.25751200	-2.59084600	-2.27560400
C	-4.64609200	-3.00906500	-2.78771600
H	-5.23714600	-3.44504000	-1.97406200
H	-4.55413700	-3.76732200	-3.57560800
H	-5.20600300	-2.16500600	-3.20079800
C	-2.53263000	-3.80314600	-1.67159000
H	-3.17085000	-4.26910800	-0.91103900
H	-1.58212300	-3.53231700	-1.20796400
H	-2.31950000	-4.55544500	-2.43949400
N	-3.16762500	-0.71050200	-3.86010700
H	-3.52417500	-0.09679700	-3.12870400

C	-2.93814400	-0.17487300	-5.09563400
O	-2.18680400	-0.68500300	-5.92474900
C	-0.72663900	3.26199200	0.13739900
C	-1.71299500	2.94014800	1.03751200
H	-1.76838000	1.99680900	1.55972700
H	-2.44085700	3.69759000	1.31319500
C	-0.63960800	4.52017000	-0.51657300
H	-1.36040000	5.27605400	-0.22712800
C	0.44866600	4.92930400	-1.33470800
O	0.35421600	6.23709300	-1.70646500
O	1.39387600	4.19359000	-1.68045400
C	1.39823000	6.89617500	-2.48750000
H	-3.39589700	-1.84073600	-1.48124100
C	2.72538300	6.89150400	-1.71591700
H	2.58494000	7.33189500	-0.72227000
H	3.46879400	7.49295700	-2.25259000
H	3.10739700	5.87634200	-1.59868600
C	0.86446600	8.32682300	-2.62307700
H	1.56428600	8.94029100	-3.20143100
H	0.73594500	8.78312900	-1.63589200
H	-0.10576000	8.32837700	-3.12971600
C	1.53453500	6.24376800	-3.86995100
H	0.56063700	6.22083800	-4.37064400
H	1.91240600	5.22387400	-3.78422600
H	2.22416800	6.82789500	-4.49096500
C	-3.66137600	1.11553900	-5.39482800
C	-3.12339400	1.93703200	-6.39110200
C	-4.83515900	1.50962800	-4.74360100
C	-3.72956300	3.15435000	-6.70088000
H	-2.23467900	1.60215900	-6.91496200
C	-5.44841500	2.72048900	-5.07076200
H	-5.29482500	0.86856600	-3.99998100
C	-4.89408800	3.55377600	-6.04223300
H	-5.36910300	4.49453300	-6.29254100
C	-3.08290500	4.06596500	-7.71093600
C	-6.68852300	3.14823700	-4.32831200
F	-2.06297900	4.77148900	-7.15418500
F	-2.55736500	3.37776000	-8.74367400
F	-3.95290500	4.96576700	-8.21353800
F	-6.38356600	3.77347300	-3.16818300
F	-7.46580400	2.08959400	-4.01021300
F	-7.43821800	4.00399500	-5.05398400
C	-5.30043500	3.17496900	0.20202600
C	-4.81271900	1.89718600	-0.10247900
C	-5.35190500	0.77307900	0.53295400
C	-6.36158200	0.96544100	1.47202900
C	-6.82628800	2.26046400	1.76902300
C	-6.30335900	3.39200300	1.13840300
C	-3.76259700	3.52229100	-1.36384900
C	-3.78217200	2.07985200	-1.12450600
H	-4.98556700	-0.22118800	0.29344100
H	-6.79976800	0.11221400	1.98128500
H	-7.61599500	2.38537400	2.50470400
H	-6.66270800	4.39180300	1.35786400
O	-4.67520200	4.15481200	-0.52166600
O	-3.12991600	1.20275400	-1.74215900
C	-3.04533600	4.17085300	-2.31950500
H	-2.42687000	3.50221700	-2.91111800
C	-3.13713600	5.54473100	-2.78857100
C	-3.80546200	6.57336700	-2.09082300
C	-2.56858500	5.84983700	-4.04294700
C	-3.89550500	7.85105200	-2.63577300
H	-4.25107700	6.36371100	-1.12620500
C	-2.67738800	7.12405000	-4.59242100
H	-2.05752300	5.06989700	-4.59912600
C	-3.33897400	8.13331800	-3.88748200
H	-4.40968300	8.63275500	-2.08291100
H	-2.25232700	7.32240600	-5.57243500
H	-3.42386300	9.13061200	-4.31060100

14-ts-*re*

P	0.47354100	1.96199000	-0.31035800
C	0.62707800	1.50962700	-2.10680300
H	1.57225300	1.95197200	-2.41761400
H	-0.14611700	2.08053500	-2.62514500
C	0.59217200	0.03005300	-2.52578400
H	1.04426200	-0.60677800	-1.75711800
C	1.44562700	-0.18163500	-3.81582100
H	1.32975900	-1.23496500	-4.07442800
C	1.00814200	0.66831800	-5.00575900
H	1.12127400	1.73903300	-4.80272200
H	-0.03306300	0.45547100	-5.26729800
H	1.62707500	0.42883400	-5.87588900
C	0.11386900	0.44723000	0.65418300
C	1.08258600	-0.08245600	1.52174700
C	-1.12062900	-0.21307400	0.50889400
C	0.82092500	-1.25072200	2.23880300
H	2.04195500	0.40781700	1.63845800
C	-1.37423600	-1.37429300	1.23871500
H	-1.88095300	0.17688500	-0.16273300
C	-0.40811600	-1.89513100	2.10273200
H	1.58187800	-1.65312800	2.90069300
H	-2.33255100	-1.87347800	1.12841600
H	-0.61350700	-2.80167800	2.66557600
C	2.07052700	2.57702300	0.31757700
C	2.07545300	3.41289600	1.44407600
C	3.28305700	2.18876600	-0.26846600
C	3.28604300	3.86007600	1.97330700
H	1.13807100	3.71962700	1.89810000
C	4.49026400	2.64242500	0.26499600

H	3.30039300	1.54588900	-1.14164600
C	4.49306600	3.47711700	1.38427200
H	3.28379200	4.51297000	2.84164800
H	5.42283700	2.34747100	-0.20629800
H	5.43443400	3.83154000	1.79528800
O	2.81257200	0.10783300	-3.50140500
Si	4.13486700	-0.91942600	-3.32087100
C	3.90660900	-2.00014400	-1.77734900
C	2.80063000	-2.87080600	-1.66292900
C	4.80956000	-1.96149600	-0.69675400
C	2.62230700	-3.67360200	-0.53430700
H	2.04102400	-2.91539500	-2.43796000
C	4.63861100	-2.76913400	0.43025400
H	5.65924700	-1.28619900	-0.72510000
C	3.54592000	-3.63308900	0.51179300
H	1.75644700	-4.32792300	-0.47672100
H	5.35871500	-2.72020200	1.24371300
H	3.41241900	-4.26623700	1.38571000
C	5.55177800	0.31895900	-3.09225300
C	5.33493100	1.68391000	-3.36527400
C	6.85443100	-0.06106500	-2.70885500
C	6.36185300	2.62449700	-3.25085800
H	4.34633600	2.01120200	-3.67238700
C	7.88476200	0.87452900	-2.59031800
H	7.07877800	-1.10330000	-2.49688600
C	7.64048900	2.22263800	-2.86021700
H	6.16178800	3.67058600	-3.46908600
H	8.87790400	0.54935500	-2.29053900
H	8.44154600	2.95219000	-2.77187100
C	4.46612200	-1.98302900	-4.89605200
C	3.32897700	-2.98255000	-5.20295400
H	3.59207500	-3.57847200	-6.08903300
H	2.37769800	-2.48703200	-5.42383100
H	3.16007200	-3.68439700	-4.37930300
C	5.76722600	-2.79248300	-4.68560600
H	5.95769700	-3.42397500	-5.56526500
H	5.70390100	-3.45609600	-3.81449100
H	6.64036600	-2.14314800	-4.55897600
C	4.65367400	-1.04835500	-6.11170100
H	5.47858400	-0.34252900	-5.96219200
H	3.74997800	-0.46510700	-6.32213700
H	4.88037600	-1.63964200	-7.01054800
N	-0.78823400	-0.39540300	-2.74653500
H	-1.53184200	0.28688800	-2.61334400
C	-1.11846900	-1.68268400	-3.03953800
O	-0.29581300	-2.59806300	-3.08741200
C	-2.61168100	-1.96681700	-3.33550500
H	-2.58698600	-2.66996600	-4.17265400
C	-3.35716900	-2.62158800	-2.13958000
C	-4.77470700	-3.04152600	-2.56246600

H	-5.32397100	-3.45001000	-1.70620500
H	-4.73150600	-3.82151200	-3.33329100
H	-5.34799000	-2.20281900	-2.96777200
C	-2.60523100	-3.82383800	-1.54830100
H	-3.19899200	-4.26647200	-0.73921400
H	-1.62690500	-3.54881700	-1.14845200
H	-2.44211900	-4.59720400	-2.30774800
N	-3.34235000	-0.78175700	-3.77457500
H	-3.61557100	-0.13150000	-3.03274600
C	-3.14826400	-0.27463300	-5.02743300
O	-2.45406500	-0.82455100	-5.88194200
C	-0.82012500	3.21252200	0.04959500
C	-1.73747800	2.91305800	1.01219800
H	-1.74568000	1.98815600	1.57132800
H	-2.47905600	3.65625300	1.28929900
C	-0.90366800	4.45267900	-0.68918200
H	-1.48476400	5.22683700	-0.19930000
C	0.21883900	4.94704800	-1.46499700
O	0.14511300	6.27858300	-1.66793500
O	1.13108300	4.23200600	-1.89671900
C	1.20269600	7.02500900	-2.36755900
H	-3.44532100	-1.85012800	-1.35925100
C	2.51817000	6.90994700	-1.58708500
H	2.37385100	7.22969400	-0.54894900
H	3.27306400	7.56209100	-2.04146100
H	2.89129700	5.88430900	-1.59095700
C	0.67415000	8.46221800	-2.32982900
H	1.38057600	9.13608000	-2.82675400
H	0.54372200	8.79769400	-1.29551800
H	-0.29243000	8.52965800	-2.83828600
C	1.34595900	6.54176900	-3.81543200
H	0.38069500	6.59471400	-4.32826700
H	1.71343800	5.51531400	-3.85407200
H	2.05242500	7.18995700	-4.34718800
C	-3.83149300	1.03993000	-5.31759200
C	-3.31666300	1.80920500	-6.36591500
C	-4.94111100	1.50883300	-4.60544400
C	-3.87929100	3.04858100	-6.67061900
H	-2.47902200	1.41694700	-6.93212500
C	-5.51307900	2.74057600	-4.92815300
H	-5.38084100	0.91307100	-3.81416200
C	-4.98009100	3.52114900	-5.95415800
H	-5.42364000	4.47858400	-6.19972600
C	-3.24676500	3.90620200	-7.73406900
C	-6.68139900	3.25730600	-4.12773000
F	-2.17532100	4.58712500	-7.24366300
F	-2.79388200	3.17290500	-8.77043100
F	-4.10282200	4.82727100	-8.22243100
F	-6.27305500	3.98850600	-3.06710600
F	-7.44141300	2.25009700	-3.64675900

F	-7.48099900	4.05400900	-4.87079600
C	-5.33544500	3.24035700	0.01678600
C	-4.93250300	1.93083700	-0.28319700
C	-5.64868800	0.84618100	0.23662200
C	-6.74679100	1.10704300	1.05189800
C	-7.12666200	2.43126900	1.34459600
C	-6.42590300	3.52415100	0.83210100
C	-3.57957100	3.45467400	-1.31948800
C	-3.76153000	2.04764400	-1.15368100
H	-5.34836600	-0.17188300	0.00384500
H	-7.32195800	0.28438100	1.46726600
H	-7.98886200	2.60835000	1.98175900
H	-6.71687500	4.54719800	1.04718200
O	-4.52876400	4.16728400	-0.57744700
O	-3.09877800	1.10416000	-1.69753900
C	-2.63390400	4.09947600	-2.11674400
H	-2.09210000	3.38852400	-2.73430500
C	-2.82201900	5.42559100	-2.73733600
C	-3.42713500	6.51174200	-2.07877200
C	-2.42262600	5.59651100	-4.07501100
C	-3.62403800	7.72141300	-2.74193800
H	-3.75010800	6.40225600	-1.05005800
C	-2.63548200	6.80195200	-4.74298200
H	-1.96587200	4.76727000	-4.60766300
C	-3.23392700	7.87319500	-4.07582600
H	-4.09308400	8.54910200	-2.21620000
H	-2.34415200	6.89206600	-5.78561800
H	-3.40236400	8.81485400	-4.59160400

14-ts-*si*

P	0.61334600	2.82491000	-0.11970000
C	1.02399600	2.19869500	-1.82302400
H	1.87674900	2.79970900	-2.15054700
H	0.16488500	2.45746700	-2.44754200
C	1.38236000	0.71322100	-2.01052700
H	1.96434900	0.34246500	-1.15963700
C	2.28590100	0.51808100	-3.26696700
H	2.44622200	-0.55984400	-3.34082900
C	1.66018200	1.02042500	-4.56590900
H	1.54204600	2.10956300	-4.55828100
H	0.68092800	0.55874900	-4.72880700
H	2.30256000	0.76288600	-5.41331500
C	0.40339500	1.49597400	1.10819900
C	1.31245200	1.34398400	2.16631100
C	-0.72166600	0.65548400	1.02502700
C	1.10230300	0.35836600	3.13138800
H	2.17754400	1.99170600	2.24569200
C	-0.92825900	-0.31462600	2.00534000
H	-1.42443200	0.77205600	0.20330300
C	-0.01900300	-0.46719700	3.05537400
H	1.81310100	0.24508700	3.94461900
H	-1.80323500	-0.95515800	1.94776200
H	-0.18753700	-1.22726300	3.81325500
C	2.04155100	3.84186200	0.38721500
C	1.84400000	5.14911100	0.85734200
C	3.34531000	3.32572700	0.28296700
C	2.94324100	5.92630600	1.22665600
H	0.84359600	5.56546200	0.91459700
C	4.43638300	4.11079900	0.65336300
H	3.52071800	2.31876200	-0.08479300
C	4.23602500	5.40991500	1.12678500
H	2.78470500	6.93916600	1.58533700
H	5.43939700	3.70619900	0.56105800
H	5.08872300	6.02094900	1.40965600
O	3.52179300	1.19874900	-3.05313000
Si	5.08211400	0.65815100	-2.72498500
C	5.14941200	-0.05779800	-0.96659100
C	4.28085900	-1.10100600	-0.57722200
C	6.04367900	0.43153500	0.00649900
C	4.31253500	-1.62872100	0.71566600
H	3.54619600	-1.50544300	-1.26820100
C	6.08351800	-0.09953200	1.29838200
H	6.72080100	1.24419300	-0.24074900
C	5.21822400	-1.13425300	1.65613300
H	3.62506200	-2.42707400	0.98241900
H	6.78967400	0.29714200	2.02398200
H	5.24779800	-1.54999200	2.66029000
C	6.07732000	2.26578900	-2.82967800
C	5.45181300	3.44827000	-3.26959800
C	7.44831000	2.34480200	-2.50885100
C	6.15534100	4.64948800	-3.38129000
H	4.39839300	3.42227700	-3.53002400
C	8.15700800	3.54343700	-2.61639300
H	7.98173900	1.46169900	-2.16716900
C	7.51102600	4.70111300	-3.05383200
H	5.64390200	5.54497300	-3.72562000
H	9.21346600	3.57103100	-2.36134300
H	8.06099900	5.63467400	-3.14099500
C	5.71210500	-0.61598200	-4.02843100
C	4.88917100	-1.92330100	-4.02387000
H	5.31552900	-2.63066600	-4.74982200
H	3.84435100	-1.76443500	-4.31077300
H	4.89876400	-2.41633800	-3.04586500
C	7.18177000	-0.97844700	-3.71049800
H	7.53904100	-1.73460000	-4.42407700
H	7.29484000	-1.40011900	-2.70429600
H	7.84784800	-0.11334600	-3.79773100
C	5.64993100	0.01535900	-5.43695600
H	6.24527400	0.93339800	-5.50049000
H	4.62329500	0.26338100	-5.73023300

H	6.04369900	-0.68875200	-6.18391200
N	0.16741000	-0.08473400	-2.12406600
H	-0.71986100	0.41141500	-2.23785600
C	0.20121100	-1.44138400	-2.03990000
O	1.24554600	-2.06986600	-1.85063700
C	-1.13686600	-2.19850500	-2.19026700
H	-0.90060600	-3.04973700	-2.83434400
C	-1.68561900	-2.73825100	-0.83371300
C	-2.92615600	-3.61389600	-1.07286700
H	-3.33624300	-3.96042700	-0.11717200
H	-2.66820700	-4.49957700	-1.66673200
H	-3.71593200	-3.07348500	-1.60229600
C	-0.63875300	-3.52217500	-0.02737800
H	-1.09458300	-3.90227000	0.89507400
H	0.22699300	-2.91332400	0.23932200
H	-0.27123600	-4.38180800	-0.59961200
N	-2.16381300	-1.43734100	-2.89373800
H	-2.49950300	-0.56670300	-2.47902600
C	-2.54736600	-1.76802500	-4.16314400
O	-2.02451700	-2.66360500	-4.82273400
C	-0.84567600	3.89660500	-0.17786900
C	-1.01911900	4.61360900	-1.32088500
H	-0.33214000	4.56614900	-2.15965400
H	-1.81180000	5.34786200	-1.38217500
C	-1.70957200	3.96797900	0.98206300
H	-1.53164000	3.25087900	1.77555900
C	-2.07403500	5.30195300	1.49077200
O	-2.36339600	5.22580500	2.81717500
O	-2.09161900	6.33880500	0.83911900
C	-2.57646100	6.42621100	3.63618700
H	-1.99507900	-1.86396300	-0.24223400
C	-1.29160700	7.26474800	3.65286200
H	-0.43977700	6.65248500	3.97098900
H	-1.39875300	8.09327100	4.36235100
H	-1.08453400	7.67830000	2.66363400
C	-2.85744700	5.83434200	5.02115300
H	-3.03371300	6.63503500	5.74767700
H	-2.00903300	5.23303300	5.36518000
H	-3.74339000	5.19244800	4.98566000
C	-3.77917600	7.24098600	3.14764100
H	-4.67207400	6.61191600	3.09650500
H	-3.59153100	7.66544100	2.16081600
H	-3.97242500	8.05647300	3.85520500
C	-3.68565300	-0.96230800	-4.73728300
C	-3.75025700	-0.84397500	-6.13126100
C	-4.68218200	-0.37518300	-3.95418400
C	-4.77388000	-0.11036400	-6.72508400
H	-2.99105300	-1.33476700	-6.72991400
C	-5.72338300	0.33610400	-4.55801800
H	-4.66325700	-0.47089200	-2.87369900
C	-5.76992100	0.48033400	-5.94146900
H	-6.57086600	1.04280100	-6.40532500
C	-4.76666500	0.11538800	-8.21371600
C	-6.79350500	0.93142900	-3.68042100
F	-4.05673200	1.22584200	-8.53799300
F	-4.20598500	-0.91465800	-8.87892000
F	-6.01256400	0.29249300	-8.70342700
F	-6.25942400	1.68425200	-2.69336000
F	-7.52558500	-0.03446200	-3.07795700
F	-7.64815700	1.71097700	-4.37343100
C	-4.00428500	4.06149200	-3.19000000
C	-3.18047300	2.94484400	-3.40231500
C	-2.80554200	2.60089400	-4.70739200
C	-3.27187600	3.38061200	-5.76256000
C	-4.09730100	4.49618500	-5.52080300
C	-4.47619300	4.85885700	-4.22732300
C	-3.61239400	3.22759500	-1.17463400
C	-2.92034300	2.37388300	-2.08095900
H	-2.17596800	1.73467200	-4.88828400
H	-3.01914300	3.11415300	-6.78439800
H	-4.45057400	5.08810800	-6.36075700
H	-5.11193400	5.71637000	-4.03213200
O	-4.26258000	4.25587800	-1.86510900
O	-2.26111900	1.31785900	-1.77822900
C	-3.63906500	3.10682800	0.22156800
H	-3.26092900	2.13684800	0.52622000
C	-4.69512600	3.63182200	1.10972500
C	-5.51371000	4.72929900	0.78331200
C	-4.93369100	2.96489700	2.32662400
C	-6.54985400	5.11688500	1.63275800
H	-5.34656600	5.26551600	-0.14172500
C	-5.96456000	3.35737200	3.17645100
H	-4.30419600	2.12081700	2.59875100
C	-6.78410300	4.43489900	2.82903700
H	-7.17959900	5.95831000	1.35537100
H	-6.13526400	2.81704300	4.10405500
H	-7.59665000	4.73872200	3.48370600

15-int-*re*

P	0.36790200	1.86943100	-0.46618600
C	0.46371600	1.45737000	-2.27296200
H	1.39766500	1.90487200	-2.61158600
H	-0.33084400	2.01793200	-2.76562800
C	0.41420100	-0.02374100	-2.68912300
H	0.89963100	-0.65820000	-1.93812200
C	1.21846000	-0.23645500	-4.00902600
H	1.09473700	-1.29132700	-4.25824700
C	0.73523600	0.60666900	-5.18525300
H	0.84818400	1.67924000	-4.99067000
H	-0.31324100	0.38630300	-5.40778500

H	1.32458800	0.36785200	-6.07590700
C	0.02702400	0.36496600	0.50871700
C	1.03319800	-0.19840200	1.30999000
C	-1.24057600	-0.24426200	0.43730700
C	0.77461200	-1.35839900	2.04080900
H	2.01557300	0.25679100	1.36321900
C	-1.48592500	-1.39742000	1.18269100
H	-2.02456200	0.17417700	-0.19562500
C	-0.48508500	-1.95470800	1.98261100
H	1.56099000	-1.79243700	2.65071700
H	-2.46606000	-1.86227000	1.13283500
H	-0.68730600	-2.85474300	2.55693300
C	1.99505400	2.49042700	0.08045600
C	2.06763700	3.32333900	1.20781600
C	3.17326100	2.10351100	-0.57501500
C	3.30650600	3.76814500	1.66849700
H	1.15991700	3.62980000	1.71839600
C	4.40962900	2.55478600	-0.10977300
H	3.14310300	1.45988700	-1.44729500
C	4.47741000	3.38612600	1.00953200
H	3.35469400	4.41644700	2.53886500
H	5.31308600	2.25865800	-0.63364400
H	5.44113100	3.73795500	1.36724800
O	2.59684900	0.05780200	-3.74501200
Si	3.91846400	-0.97602300	-3.59732600
C	3.73247400	-2.04294000	-2.03823100
C	2.64061900	-2.92701700	-1.89252000
C	4.65561500	-1.97982900	-0.97585700
C	2.49700300	-3.72110200	-0.75275200
H	1.86385000	-2.98742800	-2.64931100
C	4.51899900	-2.77816700	0.16242800
H	5.49498200	-1.29323700	-1.02860300
C	3.44125100	-3.65748000	0.27373800
H	1.64159200	-4.38642700	-0.67131000
H	5.25481000	-2.71137000	0.96045100
H	3.33524700	-4.28508100	1.15540600
C	5.34687300	0.25955000	-3.42077900
C	5.13299300	1.61842700	-3.72530900
C	6.65526900	-0.11897400	-3.05605400
C	6.16786500	2.55485700	-3.65753400
H	4.14027600	1.94353300	-4.02171200
C	7.69353400	0.81267800	-2.98301600
H	6.87794900	-1.15717600	-2.82368800
C	7.45195700	2.15497800	-3.28270800
H	5.97049500	3.59592700	-3.90111600
H	8.69093100	0.48863700	-2.69652300
H	8.25937200	2.88101400	-3.23102300
C	4.20340800	-2.04776500	-5.17460400
C	3.05217000	-3.03963300	-5.45304900
H	3.29072400	-3.63823400	-6.34411400
H	2.09970000	-2.53762700	-5.65266200
H	2.89742400	-3.73958600	-4.62513100
C	5.50336400	-2.86570800	-4.99122900
H	5.67086400	-3.49886300	-5.87427500
H	5.45372800	-3.52838300	-4.11855900
H	6.38359900	-2.22240600	-4.88349000
C	4.36858300	-1.11795300	-6.39741700
H	5.20586600	-0.42185900	-6.27340200
H	3.46621800	-0.52460400	-6.58433700
H	4.56295200	-1.71452200	-7.30024600
N	-0.97377300	-0.43780700	-2.85198500
H	-1.71119700	0.24436300	-2.65260600
C	-1.31890200	-1.72717600	-3.12038600
O	-0.49869400	-2.64401400	-3.19839300
C	-2.82506900	-2.00129400	-3.35151500
H	-2.84122200	-2.72703700	-4.16935600
C	-3.53178100	-2.61269600	-2.11032600
C	-4.97261100	-3.01460500	-2.46568200
H	-5.49396100	-3.39495900	-1.57931800
H	-4.97569500	-3.81046300	-3.22162100
H	-5.54449100	-2.17195700	-2.86438300
C	-2.77644600	-3.81645300	-1.52648400
H	-3.33776900	-4.22901500	-0.67904100
H	-1.77389000	-3.55343300	-1.18142600
H	-2.66619400	-4.61065100	-2.27427100
N	-3.55141200	-0.81496300	-3.79391800
H	-3.73711600	-0.11829200	-3.05568400
C	-3.39752200	-0.36594600	-5.07212200
O	-2.76302500	-0.97272200	-5.93697900
C	-0.89598900	3.12304100	-0.03525700
C	-1.64729800	2.90083800	1.05805600
H	-1.56010500	2.01704100	1.67804500
H	-2.39881000	3.62721300	1.35592800
C	-1.20345900	4.34970500	-0.87621300
H	-1.63684400	5.09671400	-0.20754900
C	0.02388100	4.96581300	-1.52382400
O	0.05112100	6.28705200	-1.35548500
O	0.85840600	4.30542700	-2.12557900
C	1.09640000	7.15052600	-1.96064600
H	-3.57449700	-1.82099200	-1.34787500
C	2.46024300	6.79803000	-1.35940500
H	2.42034000	6.84055900	-0.26543400
H	3.20529900	7.52591400	-1.70020800
H	2.78398100	5.80044100	-1.66126400
C	0.64697700	8.54858700	-1.53059300
H	1.33873200	9.30131400	-1.92297900
H	0.62706900	8.63170200	-0.43888000
H	-0.35600500	8.76067800	-1.91329100
C	1.07415700	7.01565400	-3.48565400
H	0.07203900	7.21807500	-3.87598400

H	1.38147400	6.01748000	-3.80189700
H	1.76585400	7.74688100	-3.91942500
C	-4.04665900	0.96005800	-5.38985400
C	-3.58015500	1.63869100	-6.51979200
C	-5.07565200	1.52549400	-4.62747800
C	-4.11016000	2.88220600	-6.86309600
H	-2.80494400	1.17219300	-7.11760600
C	-5.61889100	2.75918800	-4.99005900
H	-5.47100000	1.00803400	-3.76165600
C	-5.13387400	3.44807900	-6.10213200
H	-5.55570500	4.40753900	-6.37683500
C	-3.51530500	3.64545300	-8.01449000
C	-6.70135400	3.38605500	-4.14818100
F	-2.38904600	4.30932700	-7.63182600
F	-3.15473900	2.83524100	-9.02989900
F	-4.36265400	4.57270300	-8.50784900
F	-6.18857700	4.22349900	-3.22193800
F	-7.42919600	2.45840600	-3.49390500
F	-7.55513900	4.11512200	-4.90541100
C	-5.40730000	3.32772300	-0.21021000
C	-5.09444000	1.99809500	-0.54063900
C	-5.94981700	0.96900900	-0.12772400
C	-7.08993800	1.30220500	0.60096100
C	-7.37990300	2.64296800	0.91838000
C	-6.53878000	3.68229900	0.51673000
C	-3.52599600	3.39043400	-1.36497000
C	-3.83772300	2.04245300	-1.29109400
H	-5.72321000	-0.06514200	-0.37445600
H	-7.76950800	0.51997800	0.92912400
H	-8.27755600	2.87547400	1.48515400
H	-6.75487100	4.72020600	0.75087000
O	-4.45876500	4.18633600	-0.68408500
O	-3.16488500	1.02423300	-1.75666600
C	-2.34204700	4.03459700	-1.97921000
H	-1.92468100	3.27638100	-2.64815500
C	-2.65082300	5.27018200	-2.81814200
C	-3.19448800	6.43484200	-2.25452400
C	-2.39987600	5.25159000	-4.19635600
C	-3.46990600	7.54791400	-3.04862400
H	-3.42177900	6.46543900	-1.19414100
C	-2.68035300	6.36272900	-4.99625200
H	-1.99189100	4.35609000	-4.65864300
C	-3.21210100	7.51801200	-4.42203600
H	-3.89784400	8.43809300	-2.59448000
H	-2.49144200	6.31172700	-6.06420900
H	-3.43384800	8.38460700	-5.03942100

15-int-*si*

P	0.35634400	2.71794000	-0.17355100
C	0.79548800	2.11722000	-1.87410300
H	1.63296100	2.73898500	-2.20100900
H	-0.07165900	2.35625700	-2.49379000
C	1.17059600	0.63487700	-2.07216400
H	1.72781300	0.24896300	-1.21033900
C	2.11778400	0.47503100	-3.29942800
H	2.28612700	-0.59939300	-3.39455700
C	1.53679100	1.00833100	-4.60624900
H	1.42747400	2.09823000	-4.58184800
H	0.55792800	0.56014200	-4.80429400
H	2.20017700	0.76100300	-5.44043600
C	0.07788200	1.39188500	1.04035700
C	0.93793900	1.26021500	2.14358900
C	-1.01898800	0.52287000	0.89429300
C	0.69833000	0.27331100	3.09945700
H	1.78907000	1.92100600	2.26138600
C	-1.25400000	-0.44849100	1.86802000
H	-1.67187100	0.61676600	0.02718900
C	-0.40030900	-0.57602900	2.96654200
H	1.36957900	0.17402100	3.94735200
H	-2.10674000	-1.11237500	1.76381800
H	-0.59133400	-1.33887400	3.71625800
C	1.77169600	3.70109100	0.42877100
C	1.54278200	4.74898700	1.33638700
C	3.08165000	3.40648900	0.02007800
C	2.61648900	5.49236700	1.82407400
H	0.53334800	4.98621100	1.65950500
C	4.15010300	4.16204700	0.50641000
H	3.27964800	2.60214900	-0.68027500
C	3.91853300	5.20244600	1.40741400
H	2.43443800	6.30399100	2.52278500
H	5.15703500	3.93715700	0.16918700
H	4.75137500	5.79095400	1.78213900
O	3.34491600	1.15495200	-3.01534600
Si	4.89395800	0.56630700	-2.70320500
C	4.91403400	-0.30835800	-1.01915700
C	4.05155100	-1.39704700	-0.76328600
C	5.76231500	0.10067700	0.02864800
C	4.05232100	-2.04933800	0.47162400
H	3.33987100	-1.73779500	-1.50970500
C	5.77081000	-0.55434800	1.26246800
H	6.42494700	0.94938200	-0.11089800
C	4.91766000	-1.63568100	1.48594800
H	3.37095900	-2.88005700	0.63521800
H	6.44222400	-0.21670600	2.04842400
H	4.92332100	-2.14841100	2.44470200
C	5.90284700	2.17118300	-2.65172700
C	5.31949200	3.37785000	-3.08514500
C	7.24998400	2.22211200	-2.23766300
C	6.03775800	4.57586800	-3.09559700
H	4.28769700	3.37389700	-3.42265600

C	7.97301400	3.41730500	-2.24238600
H	7.75326400	1.31806500	-1.90533500
C	7.36718500	4.60001900	-2.67082900
H	5.55892900	5.48990600	-3.43814400
H	9.00979200	3.42247000	-1.91548200
H	7.92859200	5.53076800	-2.67888600
C	5.55336400	-0.57294800	-4.11184500
C	4.72215500	-1.86468200	-4.27602000
H	5.17300400	-2.49644700	-5.05494000
H	3.69069200	-1.66615500	-4.58616600
H	4.68964700	-2.45814100	-3.35602700
C	7.00836000	-0.98114900	-3.78148000
H	7.39407300	-1.64606700	-4.56736400
H	7.07866900	-1.52349100	-2.83074900
H	7.67928800	-0.11631700	-3.73336600
C	5.54558500	0.20402800	-5.44700500
H	6.15658300	1.11234700	-5.39615700
H	4.53265600	0.50054600	-5.74258300
H	5.95197600	-0.42523800	-6.25170200
N	-0.04414200	-0.15279500	-2.24093500
H	-0.93346200	0.35780200	-2.32937300
C	-0.01341300	-1.51119300	-2.20362500
O	1.03292900	-2.14746300	-2.04614000
C	-1.35015000	-2.26622800	-2.35800800
H	-1.12559900	-3.09057600	-3.04038800
C	-1.86123600	-2.86893200	-1.01062200
C	-3.09111400	-3.75652800	-1.26090600
H	-3.47177600	-4.15375100	-0.31258100
H	-2.83280000	-4.60842600	-1.90181200
H	-3.90295100	-3.20657500	-1.74484500
C	-0.78931800	-3.66345600	-0.24893100
H	-1.22589600	-4.08760600	0.66382600
H	0.06869700	-3.04912200	0.03031800
H	-0.41293200	-4.49318000	-0.85828500
N	-2.40128100	-1.48680800	-3.00252700
H	-2.68481600	-0.59217800	-2.58597200
C	-2.91620900	-1.86900600	-4.20780400
O	-2.47680900	-2.80437200	-4.87556600
C	-1.08117300	3.81341400	-0.31090000
C	-1.19164900	4.53400500	-1.44379800
H	-0.45493400	4.50562800	-2.24085900
H	-2.01687400	5.22548600	-1.57204700
C	-2.17240600	3.83924400	0.73879000
H	-1.85485200	3.31693200	1.64290500
C	-2.47366300	5.28270600	1.14078100
O	-2.13615000	5.47736200	2.43244000
O	-2.92979100	6.12775000	0.39788800
C	-2.36707700	6.77092300	3.11482700
H	-2.17666700	-2.02237000	-0.38355000
C	-1.51834200	7.86437200	2.45790400
H	-0.45880600	7.58240100	2.45582600
H	-1.61709600	8.79521200	3.02772500
H	-1.83771000	8.04775000	1.43079800
C	-1.88372600	6.48249100	4.53843600
H	-1.99729400	7.37600600	5.16130500
H	-0.82774700	6.19146700	4.54019300
H	-2.46596300	5.67077900	4.98586900
C	-3.86064000	7.11143200	3.10814400
H	-4.44638900	6.27732000	3.50713100
H	-4.21418400	7.33463700	2.10067400
H	-4.03253500	7.98797100	3.74343200
C	-4.10363500	-1.07978800	-4.69966900
C	-4.30591800	-1.01748500	-6.08393300
C	-5.02295300	-0.46719200	-3.84461200
C	-5.38922100	-0.31313100	-6.60206400
H	-3.60484100	-1.52756400	-6.73493000
C	-6.12901100	0.20732100	-4.37029500
H	-4.88864500	-0.51062800	-2.76913100
C	-6.31161700	0.29757000	-5.74691600
H	-7.16045300	0.83574500	-6.15052600
C	-5.53041400	-0.14077900	-8.09056600
C	-7.12897900	0.81292900	-3.42017100
F	-4.88838800	0.97451900	-8.52004400
F	-5.00733800	-1.17990700	-8.77360800
F	-6.82281500	-0.01379100	-8.46381700
F	-6.52544600	1.55153700	-2.46582000
F	-7.83609900	-0.14834300	-2.77793000
F	-8.01874500	1.60594700	-4.05301600
C	-4.32925800	3.93414400	-3.19709800
C	-3.53985300	2.80056200	-3.46302600
C	-3.27948900	2.44931000	-4.79463400
C	-3.82998100	3.22460600	-5.81285000
C	-4.62695100	4.34755200	-5.51597800
C	-4.88777100	4.72340300	-4.19727700
C	-3.74034200	3.11633700	-1.23435900
C	-3.16534000	2.25398500	-2.15933100
H	-2.67101000	1.57943700	-5.02428400
H	-3.66336400	2.94774700	-6.85012100
H	-5.04743400	4.93384000	-6.32861100
H	-5.49689400	5.58859800	-3.95440700
O	-4.44900400	4.14859900	-1.85511100
O	-2.43175200	1.20314200	-1.88886800
C	-3.52008600	3.06249900	0.22729000
H	-3.24857600	2.01684900	0.39359900
C	-4.68490000	3.36445500	1.16125200
C	-5.63952900	4.35961900	0.90347600
C	-4.79681400	2.62513700	2.34914300
C	-6.67784700	4.59559900	1.80721200
H	-5.57168200	4.93972100	-0.00812300
C	-5.82860300	2.86593000	3.25662200

H	-4.06907200	1.84397100	2.56113800
C	-6.77687600	3.85470300	2.98653200
H	-7.41492000	5.36281300	1.58412200
H	-5.89740000	2.27519200	4.16663200
H	-7.58880400	4.04072500	3.68480400

16-ts-*re*

P	0.31799200	1.86597900	-0.30709600
C	0.42132800	1.35199300	-2.10932200
H	1.36578200	1.76402600	-2.46401500
H	-0.35591700	1.92059000	-2.62343100
C	0.34120200	-0.13405700	-2.49987700
H	0.80720900	-0.76753900	-1.73699700
C	1.12949900	-0.39783600	-3.82139900
H	0.97426900	-1.45327900	-4.05145500
C	0.66196800	0.43819600	-5.00998000
H	0.80884300	1.50950000	-4.83377100
H	-0.39464900	0.24736300	-5.22301700
H	1.23704500	0.16546100	-5.90030800
C	0.01944200	0.37887400	0.72325700
C	0.99115400	-0.05597900	1.63805100
C	-1.18363700	-0.34175200	0.60620800
C	0.76414500	-1.18622300	2.42490700
H	1.92721800	0.48070700	1.73886400
C	-1.40492400	-1.46506200	1.40288400
H	-1.94760700	-0.02444000	-0.09640800
C	-0.43388100	-1.89044800	2.31200400
H	1.52859400	-1.51309000	3.12375700
H	-2.34006200	-2.00953000	1.30844200
H	-0.61107700	-2.76841800	2.92726600
C	1.99556800	2.45190500	0.14728400
C	2.12049800	3.40192900	1.17206100
C	3.15059700	1.93994200	-0.46211900
C	3.38270400	3.83738100	1.57687700
H	1.22561700	3.80018100	1.64085600
C	4.41142500	2.38055500	-0.05567000
H	3.08282500	1.20589400	-1.25764100
C	4.52898800	3.32842800	0.96280500
H	3.46865900	4.57617700	2.36913600
H	5.29610000	1.98625800	-0.54604700
H	5.51176700	3.67109200	1.27548000
O	2.51742200	-0.13970900	-3.57850000
Si	3.81463900	-1.20056400	-3.41924900
C	3.62394900	-2.21642100	-1.82755200
C	2.51478200	-3.07031900	-1.64043300
C	4.55648700	-2.13232100	-0.77521900
C	2.36143800	-3.81412200	-0.46860300
H	1.73313000	-3.14413700	-2.39101000
C	4.41001900	-2.88063000	0.39517600
H	5.40909600	-1.46569600	-0.86066300
C	3.31312600	-3.72895100	0.54931700
H	1.49211200	-4.45623500	-0.35412700
H	5.15173900	-2.79698800	1.18606900
H	3.19728000	-4.31472000	1.45803200
C	5.27617600	0.00199900	-3.29782100
C	5.08919200	1.35819800	-3.63006600
C	6.58131800	-0.40008100	-2.94684300
C	6.14768100	2.26966900	-3.60436600
H	4.09843200	1.70138800	-3.91172700
C	7.64311500	0.50708100	-2.91560200
H	6.78271500	-1.43723900	-2.69120200
C	7.42854100	1.84715100	-3.24422700
H	5.97050300	3.30977800	-3.86669900
H	8.63734500	0.16574300	-2.63818600
H	8.25396900	2.55428900	-3.22408300
C	4.04918700	-2.32440200	-4.96922900
C	2.86996400	-3.29436800	-5.20339900
H	3.08127400	-3.92485700	-6.07933400
H	1.92792100	-2.77397300	-5.40543400
H	2.70896700	-3.96501300	-4.35276400
C	5.33063500	-3.16966800	-4.78078200
H	5.47056200	-3.83062700	-5.64823400
H	5.27609600	-3.80662500	-3.88940600
H	6.22777900	-2.54598400	-4.70179400
C	4.21906600	-1.43419700	-6.22041900
H	5.07307800	-0.75387400	-6.12700700
H	3.32771600	-0.82594400	-6.41243200
H	4.38788800	-2.06039800	-7.10838800
N	-1.05757400	-0.53398400	-2.65674600
H	-1.77641400	0.17491000	-2.53710000
C	-1.43012300	-1.81947000	-2.89954600
O	-0.63246100	-2.75684300	-2.94985200
C	-2.93930200	-2.07675300	-3.13447100
H	-2.96105400	-2.78214400	-3.96987900
C	-3.64948900	-2.71605300	-1.90778700
C	-5.08870900	-3.11328800	-2.27641700
H	-5.61400000	-3.50671600	-1.39839000
H	-5.08619300	-3.89868900	-3.04290400
H	-5.66123900	-2.26745400	-2.66834800
C	-2.89896400	-3.92985100	-1.33835600
H	-3.47061800	-4.35637900	-0.50501400
H	-1.90072700	-3.67262000	-0.97826700
H	-2.78002900	-4.71007900	-2.09898200
N	-3.66831700	-0.88269400	-3.54897200
H	-3.88881800	-0.21441200	-2.80612800
C	-3.56832300	-0.41091700	-4.82626900
O	-2.95283600	-0.99241700	-5.71920200
C	-0.85259000	3.11475300	0.14446200
C	-2.01932800	2.82278500	0.86373300
H	-2.19624700	1.84435100	1.29796400

H	-2.49493800	3.62958100	1.42146600
C	-1.09603900	4.39068700	-0.62711500
H	-1.39530800	5.18267200	0.06824000
C	0.04273000	4.92569500	-1.46797000
O	0.10697500	6.25820100	-1.40531600
O	0.78180900	4.21168300	-2.12824500
C	1.07750300	7.04548800	-2.20024300
H	-3.69699400	-1.94190200	-1.12615700
C	2.50272600	6.70406200	-1.75431800
H	2.60896300	6.83997000	-0.67252600
H	3.21060500	7.37614300	-2.25276900
H	2.76164000	5.67404300	-2.00517300
C	0.71190000	8.48395400	-1.82619100
H	1.36030000	9.18675500	-2.35995600
H	0.83447300	8.64749500	-0.75042800
H	-0.32845500	8.69581000	-2.09184600
C	0.85451700	6.79916400	-3.69572800
H	-0.19289500	6.97649400	-3.96028700
H	1.12046600	5.77852900	-3.97577000
H	1.47701200	7.49277400	-4.27246300
C	-4.25790300	0.90536700	-5.09495500
C	-3.83687200	1.62993600	-6.21522700
C	-5.28162800	1.42060400	-4.29254400
C	-4.40957400	2.86718200	-6.50698700
H	-3.06314000	1.20513600	-6.84485600
C	-5.86051000	2.65395000	-4.59690100
H	-5.64805900	0.86452800	-3.43816500
C	-5.42459200	3.38585100	-5.70037300
H	-5.87469200	4.34372900	-5.93297600
C	-3.87421400	3.69335700	-7.64508300
C	-6.91264600	3.23389500	-3.68686400
F	-2.86726400	4.50658100	-7.22401400
F	-3.37057700	2.93284900	-8.63600700
F	-4.82024700	4.49492800	-8.17877900
F	-6.35726300	3.98836400	-2.71288200
F	-7.63824800	2.27020800	-3.08083000
F	-7.77227700	4.02994900	-4.36084900
C	-5.40313400	3.24858400	0.28280300
C	-5.18179200	1.96351000	-0.23400100
C	-6.14050400	0.96064100	-0.04483400
C	-7.29387300	1.27351900	0.66900300
C	-7.48849300	2.56856800	1.18746800
C	-6.54403600	3.57960200	1.00783900
C	-3.39284000	3.33438700	-0.66972900
C	-3.89639700	2.01721300	-0.93095700
H	-5.97870600	-0.03762200	-0.44310200
H	-8.05411000	0.51495800	0.83179500
H	-8.39849100	2.78802100	1.73933100
H	-6.68995200	4.58053100	1.40066100
O	-4.37475100	4.09625400	-0.00323600
O	-3.31339700	1.09149500	-1.57527400
C	-2.39905500	4.07045200	-1.52739500
H	-2.06666000	3.33151700	-2.26432300
C	-2.92435700	5.27734100	-2.28305900
C	-3.40628200	6.41884000	-1.62363100
C	-2.88922800	5.28517000	-3.68456100
C	-3.83967400	7.52994200	-2.34694800
H	-3.45350000	6.43482100	-0.53978800
C	-3.32210500	6.39625500	-4.41249400
H	-2.51994400	4.41311400	-4.21857700
C	-3.79823400	7.52488000	-3.74374500
H	-4.21430800	8.40207300	-1.81706500
H	-3.28549900	6.36912600	-5.49759100
H	-4.13728500	8.39195900	-4.30452900

16-ts-*si*

P	-0.06521900	2.55987900	0.50244200
C	0.86635900	2.52419700	-1.11477800
H	1.70972900	3.20658100	-0.98004100
H	0.19585800	2.95635600	-1.86030400
C	1.42023900	1.18976900	-1.64448100
H	1.81261600	0.57590200	-0.82730100
C	2.61693800	1.44330700	-2.61496200
H	2.92193400	0.45350700	-2.96218300
C	2.25906100	2.30821600	-3.82199300
H	2.02200000	3.33437900	-3.52078700
H	1.40234000	1.88990600	-4.35995900
H	3.10593100	2.35306000	-4.51323800
C	-0.42314800	0.90148900	1.18385000
C	0.12961800	0.46620300	2.39833500
C	-1.35285600	0.08636900	0.51308300
C	-0.23646300	-0.77012600	2.93282600
H	0.83801400	1.08782300	2.93348000
C	-1.72026900	-1.14179000	1.06211800
H	-1.78530200	0.42142600	-0.42597900
C	-1.16288800	-1.57317600	2.26859100
H	0.19915200	-1.09799100	3.87229500
H	-2.44489100	-1.76463800	0.54618500
H	-1.45416500	-2.53148400	2.68972500
C	1.12248800	3.34585100	1.66336300
C	0.72752600	4.47392200	2.39361400
C	2.43371700	2.85686500	1.79860900
C	1.62898200	5.10254100	3.25544400
H	-0.27777200	4.86505500	2.27102400
C	3.33010400	3.48953200	2.65901300
H	2.76515200	1.98560900	1.24056900
C	2.92833400	4.61169900	3.38896400
H	1.31572000	5.97988000	3.81465600
H	4.34227700	3.10743400	2.74925500
H	3.63107200	5.10549700	4.05451900

O	3.67434400	2.07078700	-1.89101100
Si	5.18193800	1.53801700	-1.36825300
C	4.96022600	0.24394900	0.00378400
C	4.15529300	-0.89688500	-0.20681600
C	5.57229500	0.37621200	1.26623200
C	3.98023000	-1.85700600	0.79247000
H	3.62914400	-1.04634100	-1.14583100
C	5.40529100	-0.58619100	2.26539100
H	6.18626300	1.24616200	1.48134500
C	4.60992900	-1.70842100	2.02954800
H	3.34843300	-2.71956500	0.59775300
H	5.89529300	-0.45673100	3.22751300
H	4.47838500	-2.45868500	2.80531000
C	5.94121200	3.13028200	-0.67992600
C	5.26640000	4.35476700	-0.84732900
C	7.18569900	3.16753000	-0.01804800
C	5.80204000	5.55506300	-0.37582700
H	4.30892600	4.36388300	-1.35865900
C	7.72609300	4.36436200	0.45754400
H	7.75161400	2.25220300	0.13399800
C	7.03426800	5.56367100	0.27945600
H	5.25647100	6.48409900	-0.52186500
H	8.68833400	4.35909900	0.96355000
H	7.45388800	6.49702300	0.64614200
C	6.25664000	0.84880700	-2.81326000
C	5.64006300	-0.41424500	-3.45478200
H	6.31371900	-0.79899400	-4.23414600
H	4.67717700	-0.21556100	-3.93771400
H	5.49189800	-1.21888200	-2.72634000
C	7.65450200	0.47091100	-2.27064700
H	8.26133900	0.03806100	-3.07887100
H	7.59859900	-0.27552200	-1.46901500
H	8.19805400	1.34286700	-1.89138300
C	6.42298000	1.93610700	-3.89778900
H	6.89138600	2.84307800	-3.49876400
H	5.46207600	2.22431200	-4.33918700
H	7.06025700	1.56268500	-4.71220700
N	0.37162300	0.43187100	-2.32005200
H	-0.49343000	0.91926000	-2.55931700
C	0.54719200	-0.87910000	-2.63598100
O	1.57728800	-1.49580800	-2.35303900
C	-0.59746700	-1.60882800	-3.36813500
H	-0.09672800	-2.19775900	-4.14115000
C	-1.39103900	-2.58401500	-2.44197500
C	-2.37691300	-3.42009400	-3.27408000
H	-2.96706800	-4.07215300	-2.61996300
H	-1.84000100	-4.05785300	-3.98693100
H	-3.07340400	-2.79570200	-3.84102100
C	-0.48962300	-3.51276100	-1.61334400
H	-1.11259800	-4.19448600	-1.02142400
H	0.16444800	-2.96515800	-0.93231200
H	0.14939600	-4.12045400	-2.26404000
N	-1.51325700	-0.72124500	-4.07816600
H	-2.03982600	-0.02077000	-3.55496200
C	-1.63680300	-0.77251000	-5.43810500
O	-0.90341700	-1.43472300	-6.16860500
C	-1.53236000	3.50727000	0.35184200
C	-1.74568200	4.37275100	-0.72157500
H	-0.99039900	4.53699000	-1.48647300
H	-2.41991400	5.21394800	-0.59059200
C	-2.81369900	3.18350100	1.08206600
H	-2.70852700	2.34527100	1.77284100
C	-3.30072600	4.37661700	1.90145800
O	-3.40224300	4.03305600	3.20004100
O	-3.53220300	5.48412900	1.45114600
C	-3.82814200	4.99506500	4.23615700
H	-1.97491000	-1.96074600	-1.74860800
C	-2.80250200	6.12925500	4.34044100
H	-1.80089300	5.72147100	4.52008600
H	-3.05893100	6.78018800	5.18400400
H	-2.78526500	6.72958300	3.42899200
C	-3.82261100	4.13702100	5.50434500
H	-4.11361400	4.74178600	6.36981800
H	-2.82516900	3.72425900	5.68819700
H	-4.52782100	3.30522800	5.40891100
C	-5.23918200	5.51236900	3.93728600
H	-5.92595800	4.67555800	3.77415900
H	-5.24931600	6.14946700	3.05203100
H	-5.60123400	6.09255900	4.79402900
C	-2.77001800	0.03219300	-6.02406000
C	-2.61836000	0.51469100	-7.32951500
C	-3.97391200	0.25100700	-5.34877100
C	-3.64239900	1.24495000	-7.92868400
H	-1.69629500	0.30347400	-7.85973900
C	-5.01037400	0.95418800	-5.96840900
H	-4.12229600	-0.13925800	-4.34764000
C	-4.84684600	1.46333800	-7.25378000
H	-5.64877600	2.01498200	-7.72899200
C	-3.42457500	1.86526600	-9.28320400
C	-6.28645000	1.19455100	-5.20370500
F	-2.84266400	3.08756800	-9.17318200
F	-2.61456300	1.11731900	-10.05882600
F	-4.58450700	2.04429800	-9.94999100
F	-6.12584100	2.14237800	-4.25598000
F	-6.69559500	0.07224800	-4.56885800
F	-7.29187800	1.59654300	-6.00970200
C	-4.02924600	4.57148100	-3.20537700
C	-3.14497000	3.57141800	-3.63942500
C	-2.72062300	3.55501300	-4.97361100
C	-3.20012400	4.53531800	-5.83823700

C	-4.08953900	5.52561500	-5.37729000
C	-4.51561100	5.56544400	-4.04924600
C	-3.55029000	3.38665600	-1.39372100
C	-2.89836100	2.71626900	-2.47721800
H	-2.03729500	2.78705100	-5.32184900
H	-2.89986900	4.52606500	-6.88182500
H	-4.45311300	6.27815700	-6.07184000
H	-5.19715700	6.32708900	-3.68465000
O	-4.32540600	4.44858200	-1.87882900
O	-2.22792700	1.63373600	-2.39390200
C	-3.86941700	2.74129900	-0.07453000
H	-3.59969800	1.69294000	-0.23359800
C	-5.31906300	2.75154500	0.38371600
C	-6.10780600	3.91273500	0.42216400
C	-5.88950600	1.54751400	0.82501900
C	-7.42447000	3.86083200	0.88347500
H	-5.69146600	4.85461700	0.08887700
C	-7.20334300	1.49560600	1.29117800
H	-5.29433800	0.63696000	0.80095800
C	-7.97824100	2.65592400	1.32086300
H	-8.02090500	4.76959900	0.89695600
H	-7.62128900	0.54901100	1.62423200
H	-9.00450200	2.62104700	1.67712100

17-int-*re*

P	0.52899500	1.95978100	-0.38693700
C	0.66050100	1.38411300	-2.19619100
H	1.60751200	1.78364400	-2.56005200
H	-0.10579500	1.96168100	-2.71852800
C	0.57100500	-0.09851200	-2.58554000
H	1.02541900	-0.73580900	-1.82051200
C	1.34583000	-0.38694800	-3.91006900
H	1.15367500	-1.43441000	-4.14873700
C	0.90637300	0.47502200	-5.09130200
H	1.07553800	1.53999000	-4.89967600
H	-0.15297200	0.30926500	-5.31264700
H	1.47928100	0.20040300	-5.98215700
C	0.36089200	0.46979500	0.67509900
C	1.32285400	0.18517500	1.65620900
C	-0.74934700	-0.38329900	0.54990100
C	1.17631800	-0.92103800	2.49461500
H	2.18967700	0.82599800	1.77007900
C	-0.89511200	-1.48480500	1.39387200
H	-1.50116200	-0.19150800	-0.20710100
C	0.06716300	-1.75680300	2.36824400
H	1.93362300	-1.12699100	3.24580200
H	-1.76072600	-2.13241100	1.28542000
H	-0.04716400	-2.61690800	3.02256700
C	2.21930300	2.62103100	-0.04938500
C	2.35366600	3.63489800	0.91016000
C	3.37146000	2.10928300	-0.66725300
C	3.61388400	4.14140600	1.23394100
H	1.45850300	4.01598100	1.39291400
C	4.63058800	2.61623200	-0.34131700
H	3.30251400	1.31635800	-1.40436000
C	4.75374300	3.63469300	0.60661900
H	3.70349900	4.93002000	1.97669700
H	5.51029400	2.21747100	-0.83797800
H	5.73517000	4.02945900	0.85656300
O	2.74181600	-0.18493700	-3.66973000
Si	4.01823100	-1.27077900	-3.52620500
C	3.91889000	-2.16328600	-1.85504300
C	2.78303800	-2.93306000	-1.52204700
C	4.94220100	-2.07351400	-0.89226900
C	2.68690100	-3.59068200	-0.29429500
H	1.94322600	-3.00983300	-2.20766100
C	4.85299100	-2.73710500	0.33392300
H	5.82131300	-1.46825000	-1.09203000
C	3.72544200	-3.50155000	0.63471200
H	1.79555400	-4.16879300	-0.06516500
H	5.66269500	-2.65089800	1.05474200
H	3.65321300	-4.01873500	1.58843000
C	5.51257200	-0.10554100	-3.58852200
C	5.32680200	1.24517700	-3.94222900
C	6.83301200	-0.52676900	-3.32970200
C	6.40250000	2.13284000	-4.02681800
H	4.32335600	1.60191000	-4.15260500
C	7.91231400	0.35587000	-3.41085800
H	7.03225800	-1.56036400	-3.05885600
C	7.69937100	1.69097000	-3.75956300
H	6.22610600	3.16952000	-4.30315300
H	8.91840400	-0.00017200	-3.20334700
H	8.53794400	2.37967300	-3.82551700
C	4.10838600	-2.53076000	-4.98841200
C	2.95007700	-3.55310900	-4.98323500
H	3.07299800	-4.25178000	-5.82402000
H	1.96552400	-3.08773400	-5.09660000
H	2.93302700	-4.14995800	-4.06520000
C	5.43249100	-3.32324800	-4.87652100
H	5.48192400	-4.07493300	-5.67745900
H	5.51303600	-3.85860600	-3.92210800
H	6.31034700	-2.67732400	-4.98302500
C	4.10404600	-1.76553000	-6.32993300
H	4.91348800	-1.02808700	-6.38335100
H	3.15905600	-1.23710400	-6.49735100
H	4.23889400	-2.46753600	-7.16558800
N	-0.83382800	-0.49679800	-2.73313500
H	-1.54161000	0.22782000	-2.73414000
C	-1.21286800	-1.78315100	-2.94996000
O	-0.42544400	-2.72989100	-2.94845300

C	-2.71787400	-2.03959700	-3.20500300
H	-2.72696800	-2.76126900	-4.02727900
C	-3.44973200	-2.65719500	-1.97902400
C	-4.87886000	-3.07421600	-2.36597800
H	-5.41530500	-3.45669300	-1.49028100
H	-4.85542600	-3.87255600	-3.11840100
H	-5.45610700	-2.24288600	-2.78216400
C	-2.70540100	-3.85352300	-1.36605900
H	-3.29325400	-4.26129500	-0.53486700
H	-1.71566000	-3.58335600	-0.99310700
H	-2.56749700	-4.65080200	-2.10515000
N	-3.44776200	-0.85742900	-3.65351200
H	-3.78148300	-0.21635600	-2.93524300
C	-3.31604100	-0.37484600	-4.92566000
O	-2.59798100	-0.89007700	-5.77908400
C	-0.64347700	3.10957300	0.11471200
C	-1.97476800	2.76532700	0.71791600
H	-2.11927800	1.71910400	0.99299200
H	-2.17862800	3.36303800	1.61986000
C	-0.91943100	4.43639500	-0.57228700
H	-1.11527400	5.21507500	0.17793300
C	0.11805600	4.98762300	-1.53112800
O	0.32826100	6.29216600	-1.31315000
O	0.65436200	4.32521700	-2.40433500
C	1.21651900	7.10289500	-2.17363500
H	-3.51824400	-1.86762300	-1.21376600
C	2.65545600	6.58719400	-2.07061900
H	2.97242500	6.54055900	-1.02336300
H	3.32631500	7.27282200	-2.60111500
H	2.75271000	5.59246800	-2.50801300
C	1.08755400	8.49657000	-1.55321600
H	1.70351900	9.21339500	-2.10649800
H	1.41837400	8.48745400	-0.50947600
H	0.04690800	8.83502000	-1.58251000
C	0.69376300	7.09918300	-3.61418200
H	-0.35788700	7.40333500	-3.64093900
H	0.78508000	6.11054900	-4.06705500
H	1.27273800	7.81259600	-4.21186900
C	-4.12788400	0.86017300	-5.22709700
C	-3.62913200	1.76520000	-6.16795900
C	-5.37012500	1.10343900	-4.63062100
C	-4.34721500	2.92249400	-6.47312900
H	-2.68430900	1.54942500	-6.65461100
C	-6.09730000	2.24681100	-4.96045300
H	-5.78882800	0.38593500	-3.93528800
C	-5.58423200	3.16738000	-5.87509000
H	-6.14898200	4.05668500	-6.13141200
C	-3.75210500	3.95070700	-7.39842300
C	-7.42749800	2.52677200	-4.30950500
F	-2.98119300	4.83408900	-6.71019500
F	-2.96331300	3.39265500	-8.33616900
F	-4.70104200	4.67260500	-8.03041100
F	-7.32327200	3.48304200	-3.36281700
F	-7.94023900	1.42671800	-3.71251900
F	-8.33636300	2.96215600	-5.20928300
C	-5.27192600	3.41105100	0.27454000
C	-5.21337200	2.25601000	-0.51926500
C	-6.36530200	1.49318000	-0.75592400
C	-7.56134400	1.90966200	-0.18668700
C	-7.60063400	3.07150000	0.61124900
C	-6.46481200	3.83940900	0.85952400
C	-3.07139000	3.27794800	-0.31545600
C	-3.83561600	2.13252900	-0.96262300
H	-6.31550600	0.59897500	-1.36957500
H	-8.47292200	1.34607300	-0.35811300
H	-8.54793500	3.38047500	1.04507200
H	-6.49647700	4.73399100	1.47195500
O	-4.08101300	4.03535400	0.40922200
O	-3.32441800	1.27605800	-1.69706100
C	-2.28214200	4.15992800	-1.32871700
H	-2.02143000	3.47922200	-2.14701800
C	-2.97602700	5.36535100	-1.92422200
C	-3.39161500	6.45640600	-1.14258600
C	-3.14640800	5.44410000	-3.31459100
C	-3.96556000	7.58085400	-1.73550200
H	-3.27034100	6.42344300	-0.06509000
C	-3.71827300	6.56950600	-3.91142500
H	-2.81832700	4.62064700	-3.94379600
C	-4.13199000	7.64273700	-3.12151200
H	-4.28244600	8.41320900	-1.11207300
H	-3.83049100	6.60074500	-4.99123300
H	-4.57780200	8.52139500	-3.58036800

17-int-*si*

P	0.01040700	2.76606600	0.65457800
C	1.08532200	2.70760400	-0.87535300
H	2.01582100	3.23568500	-0.65248300
H	0.54728700	3.31156000	-1.61262800
C	1.48544100	1.35697700	-1.48273800
H	1.89923200	0.71215500	-0.70338400
C	2.61808500	1.54752900	-2.54207100
H	2.81045900	0.55473300	-2.95560100
C	2.24581000	2.49508800	-3.68195400
H	2.13950000	3.52437000	-3.32333700
H	1.30778200	2.18654500	-4.15617300
H	3.03088400	2.48858100	-4.44392300
C	-0.02711900	1.17953400	1.57416700
C	0.63714600	1.00489700	2.79706500
C	-0.79439100	0.12035700	1.06100000
C	0.53980800	-0.20458400	3.48805200

H	1.22452300	1.81160400	3.21948100
C	-0.89400700	-1.08399400	1.75570700
H	-1.31730700	0.25088300	0.12015700
C	-0.22542500	-1.24968100	2.97118300
H	1.06022800	-0.32341000	4.43424700
H	-1.49493500	-1.89217300	1.34927200
H	-0.30512000	-2.18842400	3.51281100
C	1.00368400	3.91383300	1.73500100
C	0.33184100	4.99254200	2.32480600
C	2.38741400	3.77302700	1.94202800
C	1.02614100	5.91083300	3.11757700
H	-0.73372300	5.11753700	2.15093500
C	3.07791300	4.68937100	2.73559500
H	2.93320800	2.94430400	1.49686900
C	2.39781400	5.76003100	3.32392900
H	0.49455300	6.74537300	3.56694100
H	4.14683700	4.56921100	2.88980400
H	2.93921500	6.47618500	3.93648500
O	3.78436000	2.04589300	-1.89528500
Si	5.23194900	1.33582200	-1.41692300
C	4.99546900	0.50970500	0.27606700
C	4.10073500	-0.57127400	0.43361400
C	5.66991700	0.96154900	1.42697900
C	3.90602800	-1.17807300	1.67581400
H	3.53259200	-0.94379300	-0.41476000
C	5.48306700	0.35217200	2.67077300
H	6.35060400	1.80491100	1.35703900
C	4.60332600	-0.72326000	2.79719600
H	3.20585600	-2.00428000	1.76594700
H	6.02398700	0.72010800	3.53943200
H	4.45784000	-1.20064300	3.76320400
C	6.36923500	2.84248100	-1.26352000
C	5.93429300	4.09730400	-1.73051900
C	7.66672300	2.77253500	-0.71634300
C	6.75192900	5.22726500	-1.65637300
H	4.93865700	4.18564300	-2.15395100
C	8.48803600	3.89902500	-0.63701300
H	8.05024600	1.82667900	-0.34215800
C	8.03178000	5.13154800	-1.10837100
H	6.38812800	6.18283500	-2.02625300
H	9.48365700	3.81329300	-0.20872900
H	8.66984900	6.00970500	-1.04917000
C	5.91746500	0.09765500	-2.73150800
C	5.16192400	-1.25053400	-2.75371900
H	5.56858300	-1.88447600	-3.55531600
H	4.08610700	-1.15175400	-2.93226100
H	5.28869000	-1.79684000	-1.81304400
C	7.40019500	-0.20573900	-2.41056900
H	7.78748100	-0.94787100	-3.12329300
H	7.52544600	-0.62710200	-1.40481600

H	8.03119700	0.68535800	-2.48940800
C	5.84357800	0.74972700	-4.13043900
H	6.35167700	1.72126500	-4.15778200
H	4.80998800	0.90140000	-4.46035300
H	6.32972800	0.10117400	-4.87346300
N	0.36144600	0.63323600	-2.08339800
H	-0.45647700	1.15542200	-2.38508500
C	0.49658700	-0.69384400	-2.36678000
O	1.50867100	-1.32754800	-2.05957700
C	-0.65958200	-1.43134200	-3.07321700
H	-0.16592900	-2.06653600	-3.81350400
C	-1.47175500	-2.34615200	-2.10160500
C	-2.53265100	-3.14340200	-2.87845500
H	-3.11314200	-3.76919600	-2.19136500
H	-2.05783200	-3.80465300	-3.61367800
H	-3.23220000	-2.49562100	-3.41451500
C	-0.58302500	-3.31226800	-1.30155000
H	-1.21217000	-3.94955400	-0.66814200
H	0.13771400	-2.79410300	-0.66733300
H	-0.01613800	-3.96508000	-1.97527000
N	-1.55083600	-0.56409800	-3.83182400
H	-2.10573000	0.13337200	-3.34865700
C	-1.62486400	-0.60875400	-5.19641600
O	-0.85340000	-1.24753400	-5.90580600
C	-1.56411800	3.22793800	0.23851600
C	-1.89284500	4.30897400	-0.75260100
H	-1.11878400	4.51103600	-1.50300400
H	-2.15371800	5.27136200	-0.29163000
C	-2.87594000	2.80949000	0.87305200
H	-2.84469600	1.81579500	1.32406600
C	-3.31769600	3.77646400	1.97322800
O	-3.48561100	3.11280000	3.13430000
O	-3.44756300	4.98320400	1.83840700
C	-3.87587100	3.78982100	4.38525300
H	-1.99437900	-1.68573700	-1.39085100
C	-2.80751500	4.81073400	4.79209300
H	-1.81746700	4.34218900	4.81257000
H	-3.02650800	5.18778700	5.79785100
H	-2.78477500	5.65401300	4.09990000
C	-3.92646600	2.63055300	5.38458800
H	-4.21054900	2.99836300	6.37640500
H	-2.94851400	2.14395500	5.46057800
H	-4.65953100	1.88150000	5.06823400
C	-5.25777300	4.43221300	4.22390500
H	-5.98196500	3.69531300	3.86108200
H	-5.22369500	5.26526700	3.52024500
H	-5.60372500	4.80490800	5.19504700
C	-2.76864700	0.16380200	-5.80679700
C	-2.60667300	0.67438900	-7.10116100
C	-3.99620400	0.32094800	-5.16053100

C	-3.65148700	1.36083700	-7.71625000
H	-1.66048900	0.52425700	-7.60868400
C	-5.05129600	0.98274600	-5.79491600
H	-4.14892000	-0.08155000	-4.16511600
C	-4.88452000	1.50926400	-7.07146000
H	-5.69840500	2.03440100	-7.55709500
C	-3.44169400	2.04121800	-9.04255800
C	-6.35931600	1.11365700	-5.05910300
F	-3.16988000	3.36184300	-8.86581400
F	-2.41225900	1.51559500	-9.73211100
F	-4.54123700	1.97399100	-9.82267800
F	-6.17337600	1.64564200	-3.83008400
F	-6.94301100	-0.09280000	-4.87556900
F	-7.23993000	1.89389900	-5.71608600
C	-4.13104800	5.07056900	-3.06023700
C	-3.43092900	4.07889800	-3.76303800
C	-3.36657500	4.09904600	-5.16273900
C	-4.02110600	5.12075000	-5.83801900
C	-4.72926000	6.10496200	-5.11679000
C	-4.79616400	6.10346200	-3.72439000
C	-3.22088200	3.79450200	-1.40799200
C	-2.89702200	3.17011500	-2.76529600
H	-2.81176600	3.33421900	-5.69557500
H	-3.99002000	5.15758900	-6.92242900
H	-5.23808000	6.89421300	-5.66412100
H	-5.33708500	6.86431700	-3.17230500
O	-4.07994100	4.92350100	-1.71694000
O	-2.27031300	2.11764100	-2.92569800
C	-3.83250400	2.77794100	-0.38750800
H	-3.61029000	1.80019900	-0.82783500
C	-5.33322400	2.82436900	-0.16756500
C	-6.01141600	3.96723100	0.28905100
C	-6.08475800	1.66661800	-0.41959600
C	-7.39135900	3.93889400	0.49480400
H	-5.45667900	4.87699900	0.48098800
C	-7.46498100	1.63676000	-0.21563600
H	-5.58082500	0.77419200	-0.78480300
C	-8.12412700	2.77621600	0.24588400
H	-7.89686600	4.83449700	0.84736200
H	-8.02184100	0.72640900	-0.42173200
H	-9.19921400	2.76090400	0.40517400

18-ts-*si*

P	1.64179900	2.48081500	0.16208000
C	1.67866100	1.71782600	-1.53419600
H	2.59587000	2.09556200	-1.98271800
H	0.85717500	2.18457300	-2.08049200
C	1.62450000	0.18558000	-1.67280000
H	2.09385300	-0.31141400	-0.81721300
C	2.41693800	-0.28128600	-2.93224700
H	2.26248600	-1.36117600	-2.98756700
C	1.94352600	0.35743400	-4.23593700
H	2.13133500	1.43650600	-4.24487100
H	0.87301600	0.18170700	-4.38411200
H	2.47872600	-0.08195700	-5.08320700
C	1.27741000	1.18043100	1.39711800
C	2.24508500	0.78855900	2.33396000
C	0.01161100	0.56723500	1.41024600
C	1.95010600	-0.19657100	3.27824500
H	3.22850800	1.24439700	2.32993600
C	-0.27751800	-0.40574500	2.36604200
H	-0.73555400	0.85287400	0.67602200
C	0.68826900	-0.78877100	3.30074200
H	2.71057300	-0.49701400	3.99280900
H	-1.25872600	-0.87092200	2.37752900
H	0.45624800	-1.55039400	4.04013500
C	3.30218600	3.11344600	0.57067500
C	3.42855400	4.15036100	1.50722000
C	4.45088500	2.54231400	0.00492200
C	4.69385600	4.61067700	1.87009500
H	2.54196500	4.60036400	1.94287400
C	5.71389800	3.01046400	0.37047300
H	4.37418200	1.74400200	-0.72506300
C	5.83650100	4.04267100	1.30227200
H	4.78479400	5.41853200	2.59077600
H	6.59550000	2.57043100	-0.08534500
H	6.82096800	4.40728700	1.58269500
O	3.80240800	0.01694300	-2.73954900
Si	5.13202700	-0.97408500	-2.44534400
C	5.02139400	-1.68769600	-0.69050400
C	3.90764800	-2.46343500	-0.30170500
C	6.01854500	-1.46173200	0.27836100
C	3.80869900	-2.99890800	0.98421000
H	3.08203300	-2.63862900	-0.98570600
C	5.92652100	-2.00213200	1.56333300
H	6.88097600	-0.84786600	0.03632200
C	4.82242700	-2.77807900	1.91842000
H	2.93425400	-3.58630200	1.25140400
H	6.71692200	-1.81338300	2.28599100
H	4.75036000	-3.20317000	2.91668900
C	6.56545100	0.25600800	-2.59710300
C	6.32828200	1.54527300	-3.11287200
C	7.89572400	-0.06645900	-2.25817000
C	7.36315400	2.46996300	-3.27354200
H	5.31702800	1.82572000	-3.39072800
C	8.93448600	0.85409900	-2.41464300
H	8.13507300	-1.05084500	-1.86463500
C	8.67017500	2.12761900	-2.92273000
H	7.14673300	3.45741900	-3.67348200
H	9.94932700	0.57499800	-2.14234300

H	9.47724500	2.84507400	-3.04758800
C	5.33321400	-2.36695500	-3.76628900
C	4.16483500	-3.37727900	-3.75805900
H	4.35622100	-4.16887200	-4.49724500
H	3.20726200	-2.91812900	-4.02529700
H	4.04454400	-3.86511300	-2.78491900
C	6.63719900	-3.14611800	-3.47479300
H	6.75201900	-3.96158600	-4.20324800
H	6.63324300	-3.59869300	-2.47574700
H	7.52503300	-2.51013100	-3.55954400
C	5.43965100	-1.73067400	-5.16985800
H	6.27856300	-1.02881700	-5.23797200
H	4.52806200	-1.18733700	-5.44359900
H	5.59703300	-2.51185500	-5.92744300
N	0.23527300	-0.25483600	-1.75920200
H	-0.47897200	0.46278700	-1.87556400
C	-0.11516600	-1.56294700	-1.66119800
O	0.70371500	-2.46717300	-1.48229200
C	-1.62019300	-1.89601000	-1.77924900
H	-1.65409900	-2.77831100	-2.42393400
C	-2.27381200	-2.24988200	-0.40981300
C	-3.73101400	-2.69697200	-0.61160100
H	-4.19979200	-2.91089900	0.35579000
H	-3.77488600	-3.61346500	-1.21342000
H	-4.33097200	-1.93533600	-1.11744100
C	-1.49995200	-3.32918000	0.36307000
H	-2.02157400	-3.55370200	1.30164500
H	-0.47765800	-3.02650000	0.59548100
H	-1.44266400	-4.25672100	-0.21844500
N	-2.39832200	-0.86642100	-2.46072200
H	-2.53143500	0.02987800	-1.99346500
C	-2.67713400	-0.95945800	-3.79631900
O	-2.25485300	-1.85936200	-4.51824600
C	0.43712300	3.84056300	0.41764900
C	-0.35838500	3.83913300	1.58712300
H	-0.21888600	3.08951400	2.35644400
H	-0.66309200	4.81256900	1.95573000
C	0.26266900	4.83317300	-0.53960600
H	-0.48758700	5.59081100	-0.34204800
C	1.11843700	5.06809800	-1.68200300
O	0.68847800	6.12649600	-2.40322100
O	2.12505700	4.40514200	-1.95271300
C	1.39082100	6.59298800	-3.60624800
H	-2.28150400	-1.32869400	0.19374800
C	2.80406500	7.06259800	-3.24017600
H	2.75999200	7.81887000	-2.44845900
H	3.27950100	7.51655000	-4.11732900
H	3.41956000	6.22912000	-2.89784200
C	0.52559400	7.77436600	-4.05649200
H	0.94223700	8.22346000	-4.96457600

H	0.48633400	8.54181700	-3.27630900
H	-0.49624200	7.44253400	-4.26551300
C	1.40166700	5.49506400	-4.67687200
H	0.38357500	5.13511700	-4.85878000
H	2.02653400	4.65389900	-4.37200500
H	1.79434300	5.90243700	-5.61574900
C	-3.58350900	0.10837300	-4.35570600
C	-3.48445200	0.38895900	-5.72329100
C	-4.55124000	0.76527900	-3.58967900
C	-4.31766900	1.34277200	-6.30437100
H	-2.75819200	-0.15678800	-6.31516500
C	-5.40404400	1.69835700	-4.18396100
H	-4.66943600	0.53371300	-2.53696600
C	-5.28566100	1.99837100	-5.53932300
H	-5.94716500	2.72356700	-5.99819700
C	-4.13123800	1.72188200	-7.74970200
C	-6.41854700	2.42172700	-3.33617200
F	-3.23862200	2.73771400	-7.87800100
F	-3.66234800	0.69568500	-8.48654400
F	-5.28320700	2.14293600	-8.31383100
F	-5.88013400	3.50126200	-2.72853400
F	-6.90788400	1.62385300	-2.36096500
F	-7.46490000	2.85932500	-4.06958600
C	-3.01942700	5.00044200	-2.12935000
C	-2.44136100	3.81380100	-2.60560200
C	-2.28834500	3.62363900	-3.98362800
C	-2.72636800	4.62551900	-4.84753500
C	-3.30426100	5.80648500	-4.34231000
C	-3.45850200	6.01593800	-2.96982200
C	-2.58232900	3.79675400	-0.32112800
C	-2.14824800	3.00035400	-1.42534300
H	-1.85061300	2.70640100	-4.36554500
H	-2.64477800	4.48260600	-5.92091800
H	-3.64550900	6.57086400	-5.03527200
H	-3.90577000	6.92123900	-2.57246600
O	-3.09276500	5.01935800	-0.76237700
O	-1.65982400	1.82633100	-1.34707500
C	-2.46281100	3.43831000	1.02326200
H	-2.29649500	2.37159100	1.12827400
C	-3.15612200	4.05935600	2.16833900
C	-3.61924000	5.38933300	2.16958400
C	-3.34817500	3.28959800	3.33157100
C	-4.25759600	5.91655000	3.29138800
H	-3.48752700	6.00217300	1.28610700
C	-3.98682300	3.81848800	4.45068400
H	-2.99019800	2.26254700	3.35052200
C	-4.44599200	5.13763500	4.43546800
H	-4.61239900	6.94376400	3.26982700
H	-4.12793800	3.20077600	5.33381000
H	-4.94538200	5.55402400	5.30611500

19-int-*si*

P	1.85311400	2.67562900	0.02410500
C	1.79848500	1.65719700	-1.53275100
H	2.78513200	1.79808600	-1.97804300
H	1.08680100	2.17172200	-2.18044700
C	1.49023600	0.15404400	-1.48977800
H	1.93866900	-0.32314700	-0.61391900
C	2.13659200	-0.54601000	-2.72775200
H	1.83364600	-1.59424600	-2.65927800
C	1.68386200	0.02068200	-4.07170500
H	2.07440500	1.03221500	-4.22651400
H	0.59191700	0.05447800	-4.13449700
H	2.05433800	-0.60807800	-4.88709100
C	1.87722900	1.62149100	1.51998000
C	2.79700100	1.88994400	2.54785300
C	0.96453300	0.56360600	1.67531400
C	2.79996800	1.11539500	3.70783700
H	3.51233700	2.69797500	2.44638900
C	0.97809200	-0.20895200	2.83564500
H	0.25304500	0.33311200	0.89023000
C	1.89317700	0.06529900	3.85333000
H	3.51702600	1.33262600	4.49414900
H	0.27561900	-1.03074000	2.93639800
H	1.90139500	-0.54021300	4.75520100
C	3.43085200	3.59144700	0.06394900
C	3.48575100	4.89636700	0.57299100
C	4.61137100	2.95496600	-0.34829500
C	4.70991800	5.56023200	0.65862600
H	2.57642800	5.39709900	0.88974900
C	5.83109400	3.62669400	-0.26435800
H	4.59637000	1.94134600	-0.73693600
C	5.88198800	4.92842100	0.23839200
H	4.74412900	6.57361000	1.04841300
H	6.73487700	3.12847500	-0.60201900
H	6.83301200	5.44988800	0.30149200
O	3.55697600	-0.42326900	-2.62767500
Si	4.77244300	-1.52833400	-2.26340100
C	4.61918800	-2.09803800	-0.46068000
C	3.42868900	-2.69813700	0.00338700
C	5.66615300	-1.94153700	0.46858900
C	3.30211300	-3.13495400	1.32359300
H	2.56615600	-2.80860900	-0.64725600
C	5.54595800	-2.38452400	1.78772900
H	6.59053600	-1.45798100	0.16754300
C	4.36382300	-2.98839000	2.21794400
H	2.36877900	-3.58845800	1.64658900
H	6.37550800	-2.25397800	2.47868200
H	4.26901100	-3.33635000	3.24373700
C	6.32989800	-0.47571500	-2.51330200
C	6.22595700	0.80581900	-3.08846200
C	7.62373200	-0.92721300	-2.18163300
C	7.35309200	1.60156500	-3.31135100
H	5.24693900	1.17978300	-3.37207100
C	8.75415400	-0.13652300	-2.39924400
H	7.76212300	-1.91264600	-1.74488000
C	8.62182900	1.13337200	-2.96397700
H	7.23920100	2.58392500	-3.76349300
H	9.73750100	-0.51437400	-2.13077800
H	9.50066400	1.74880600	-3.13907700
C	4.78450000	-3.02336200	-3.47986000
C	3.49380500	-3.86731800	-3.39048500
H	3.56574600	-4.72567900	-4.07390600
H	2.60101200	-3.30362200	-3.68155700
H	3.32868600	-4.26683500	-2.38410700
C	5.98147500	-3.94121800	-3.14051200
H	5.98680500	-4.80983200	-3.81460400
H	5.92880300	-4.32433800	-2.11415600
H	6.94219600	-3.42973200	-3.26660100
C	4.94624200	-2.50427800	-4.92580500
H	5.87186500	-1.93160600	-5.05442800
H	4.11159800	-1.85935200	-5.22388400
H	4.97726300	-3.34873300	-5.62914000
N	0.04750800	-0.06746300	-1.46908900
H	-0.57442400	0.69384700	-1.79143300
C	-0.47015300	-1.32156700	-1.30246700
O	0.19709500	-2.27021700	-0.88793500
C	-1.96284300	-1.50489100	-1.67104800
H	-2.05450000	-2.57669200	-1.86041800
C	-2.93109500	-1.10886000	-0.52654400
C	-4.38921600	-1.38632300	-0.92245900
H	-5.06584700	-1.08590600	-0.11437500
H	-4.54453700	-2.45606200	-1.11308700
H	-4.67519500	-0.83937900	-1.82500900
C	-2.58608000	-1.85760900	0.77043000
H	-3.26933800	-1.55710200	1.57254000
H	-1.55970000	-1.67031100	1.10356200
H	-2.68469600	-2.94103200	0.63095500
N	-2.30759900	-0.82946500	-2.92082400
H	-2.37722100	0.19545200	-2.89082300
C	-2.13337400	-1.48410300	-4.10456400
O	-1.70911700	-2.63711300	-4.18463500
C	0.44816400	3.82977200	0.17624400
C	-0.57701100	3.63176400	1.25185300
H	-0.19464800	3.08697100	2.11658200
H	-0.92268900	4.60898900	1.60467200
C	0.16612300	4.76936900	-0.76744700
H	-0.68011800	5.42628900	-0.59537400
C	0.92396400	5.03730000	-2.00650000
O	0.37348100	6.05845500	-2.66782300

O	1.91293800	4.40158200	-2.35657900
C	0.84862700	6.49910600	-3.99910500
H	-2.82729200	-0.02885100	-0.35515000
C	2.28548100	7.01858500	-3.88690900
H	2.35052100	7.80027000	-3.12189000
H	2.58845000	7.45648100	-4.84462700
H	2.98064200	6.21602800	-3.63409600
C	-0.12470000	7.63136900	-4.33462900
H	0.11453100	8.04877400	-5.31846500
H	-0.05533500	8.43340200	-3.59224800
H	-1.15450800	7.26165100	-4.35086600
C	0.71294500	5.34845300	-5.00104900
H	-0.31304700	4.96678700	-5.00103000
H	1.39793000	4.53119200	-4.76742300
H	0.94216900	5.71713000	-6.00719100
C	-2.51537400	-0.74335600	-5.36519500
C	-1.98882700	-1.24098100	-6.56133900
C	-3.40904100	0.33447200	-5.40406000
C	-2.32996700	-0.65866900	-7.78159900
H	-1.33064500	-2.10111500	-6.51403300
C	-3.76734000	0.89562800	-6.63197700
H	-3.84863500	0.72102800	-4.49187800
C	-3.22605600	0.40820500	-7.82330300
H	-3.51642200	0.84206000	-8.77354900
C	-1.69531500	-1.16162100	-9.05195500
C	-4.73404100	2.04774900	-6.70981400
F	-0.48573700	-0.58992500	-9.25774300
F	-1.49850200	-2.49582400	-9.02048000
F	-2.45128100	-0.88424200	-10.13728200
F	-4.09071100	3.20504700	-7.01878600
F	-5.39075000	2.25050800	-5.55360400
F	-5.65660900	1.85615100	-7.67920400
C	-3.37494300	5.01152800	-1.77307100
C	-2.94866200	4.01436200	-2.66190600
C	-3.17597800	4.17436000	-4.03257600
C	-3.82987600	5.32566400	-4.47098900
C	-4.24964300	6.31005100	-3.55600700
C	-4.02655800	6.16884300	-2.18383700
C	-2.41657700	3.43669600	-0.53992100
C	-2.30760800	2.96979500	-1.85441700
H	-2.84864400	3.41462500	-4.73420000
H	-4.03444200	5.45419900	-5.52966300
H	-4.76337200	7.19528100	-3.92153700
H	-4.34716800	6.92032600	-1.46871700
O	-3.05115900	4.68870200	-0.48387900
O	-1.77132900	1.87893800	-2.29924000
C	-1.87791300	2.85643800	0.70846500
H	-1.54339500	1.85363100	0.42270400
C	-2.84654200	2.71215000	1.88058000
C	-3.65713900	3.77168300	2.31655100
C	-2.89796400	1.50300900	2.58812800
C	-4.50178500	3.61525700	3.41666100
H	-3.64272300	4.71329700	1.77954700
C	-3.74119900	1.34527000	3.68965800
H	-2.26987300	0.67352200	2.27459500
C	-4.54923600	2.40296500	4.10797300
H	-5.12835200	4.44573900	3.73215300
H	-3.76643000	0.39542000	4.21800800
H	-5.20940500	2.28427700	4.96306000

20-ts-*si*

P	1.87887400	2.58491200	-0.02418100
C	1.88685600	1.52712100	-1.57328000
H	2.90759800	1.59723000	-1.95447200
H	1.26187800	2.07846400	-2.27641800
C	1.50407600	0.04374700	-1.54314100
H	1.90505600	-0.45410300	-0.65638900
C	2.13667700	-0.69837200	-2.76438900
H	1.78451300	-1.73112900	-2.69682000
C	1.73814100	-0.12346100	-4.12191200
H	2.15281000	0.87972800	-4.26539400
H	0.64947000	-0.07135900	-4.22027600
H	2.12006700	-0.76229200	-4.92412200
C	2.06397900	1.49668700	1.43853200
C	3.09943100	1.70156600	2.36267600
C	1.13504400	0.46773500	1.67173500
C	3.20485000	0.89101500	3.49419000
H	3.82353700	2.49246000	2.20319000
C	1.24627000	-0.34049900	2.80191000
H	0.32394300	0.29531400	0.97166600
C	2.28170900	-0.13089900	3.71485000
H	4.01235900	1.06032800	4.20088800
H	0.52551500	-1.13661700	2.96364900
H	2.36882000	-0.76381900	4.59354900
C	3.45676000	3.52599500	-0.12699500
C	3.48642900	4.87467300	0.24916000
C	4.64955900	2.89718600	-0.51986600
C	4.68488300	5.58969500	0.21692300
H	2.56693900	5.35731300	0.56477000
C	5.84643100	3.61316400	-0.54731600
H	4.66328200	1.84753600	-0.79805000
C	5.86531900	4.96158300	-0.18316900
H	4.69380500	6.63754600	0.50453900
H	6.75820600	3.11248100	-0.85996900
H	6.79782800	5.51903700	-0.20850400
O	3.55878700	-0.64398400	-2.64104700
Si	4.71887600	-1.81024000	-2.29407900
C	4.51496200	-2.43764700	-0.51622900
C	3.28861700	-2.98950300	-0.08764600
C	5.55922100	-2.37842700	0.42686400

C	3.12503600	-3.47630900	1.21067000
H	2.42806800	-3.02232100	-0.74918600
C	5.40201200	-2.87173400	1.72399000
H	6.51042900	-1.93114100	0.15483400
C	4.18467100	-3.42847200	2.11808600
H	2.16481700	-3.89026600	1.50699800
H	6.23003200	-2.81587800	2.42673900
H	4.06083800	-3.81505200	3.12685400
C	6.32607700	-0.82537200	-2.49790300
C	6.28841000	0.47093300	-3.04721100
C	7.59388700	-1.34535600	-2.16654600
C	7.45455200	1.21457500	-3.24779300
H	5.33075600	0.89802600	-3.32842500
C	8.76295200	-0.60617300	-2.36031900
H	7.68107200	-2.34498400	-1.74912900
C	8.69652600	0.67876000	-2.90156700
H	7.39182300	2.20969700	-3.68177800
H	9.72467400	-1.03605600	-2.09172500
H	9.60531400	1.25434400	-3.05838000
C	4.68032100	-3.26699000	-3.55892400
C	3.34016100	-4.03466500	-3.53232300
H	3.38294400	-4.87765200	-4.23726700
H	2.49142900	-3.41157100	-3.83423900
H	3.12005500	-4.45024300	-2.54296100
C	5.81041800	-4.26483900	-3.21679800
H	5.78723600	-5.10952600	-3.92042800
H	5.70322700	-4.67706700	-2.20630900
H	6.80229500	-3.80619900	-3.29659000
C	4.91538900	-2.71670500	-4.98300800
H	5.87933800	-2.20247700	-5.06949100
H	4.13347100	-2.00929100	-5.28243800
H	4.91110000	-3.54006500	-5.71181800
N	0.04995000	-0.11891600	-1.55232100
H	-0.52378100	0.66580600	-1.86465100
C	-0.53191300	-1.34122100	-1.37861800
O	0.08155600	-2.32333100	-0.96321200
C	-2.03559900	-1.45082500	-1.72903500
H	-2.17545700	-2.51613800	-1.92619500
C	-2.96721100	-1.02396200	-0.56706200
C	-4.43871600	-1.29322900	-0.91766000
H	-5.09332400	-0.95276700	-0.10724000
H	-4.61315600	-2.36698200	-1.06234800
H	-4.74056800	-0.77833200	-1.83426200
C	-2.59308200	-1.74334800	0.73875500
H	-3.26987900	-1.44057200	1.54606800
H	-1.56769600	-1.53106500	1.05629900
H	-2.67680300	-2.83021400	0.62086700
N	-2.38141700	-0.75046100	-2.96611700
H	-2.48691300	0.26324700	-2.92442100
C	-2.16629800	-1.36466600	-4.16795900
O	-1.68117600	-2.48951200	-4.26955900
C	0.53779100	3.67271000	0.30376700
C	-0.43972600	3.46381300	1.42443600
H	-0.15579700	2.71425900	2.16458600
H	-0.60552700	4.40418800	1.96879400
C	-0.05831900	4.58401700	-0.60539700
H	-0.52089900	5.46602100	-0.16266800
C	0.50741500	4.89936400	-1.94703100
O	-0.08200500	5.98472400	-2.46886400
O	1.41057600	4.26638600	-2.47794900
C	0.31133800	6.54318900	-3.78111200
H	-2.84201400	0.05851000	-0.42486600
C	1.77151800	7.00563500	-3.72487000
H	1.92039800	7.69896800	-2.88959300
H	2.02183500	7.53347600	-4.65202600
H	2.45226600	6.16074400	-3.60878000
C	-0.63457600	7.73781700	-3.92724900
H	-0.44633600	8.24953200	-4.87717800
H	-0.48199500	8.45347700	-3.11248900
H	-1.67812900	7.40931300	-3.90772400
C	0.06700100	5.51498000	-4.89003100
H	-0.97405300	5.17762000	-4.87433800
H	0.72345800	4.65034300	-4.78046400
H	0.26086000	5.98068700	-5.86307300
C	-2.58762900	-0.61461700	-5.40840400
C	-1.99046400	-1.00268600	-6.61148600
C	-3.59154600	0.36172300	-5.42359000
C	-2.36844100	-0.40399400	-7.81346400
H	-1.24715000	-1.79182900	-6.58766300
C	-3.98302100	0.93895900	-6.63317000
H	-4.09580700	0.64762900	-4.50747500
C	-3.36938300	0.56583200	-7.83098200
H	-3.68349900	1.01044200	-8.76846200
C	-1.66367100	-0.79229500	-9.08825100
C	-5.06370100	1.98638700	-6.67878100
F	-0.46808800	-0.16980000	-9.19738900
F	-1.42294000	-2.11849000	-9.13899100
F	-2.38412600	-0.46354500	-10.18239300
F	-4.53826100	3.21903200	-6.91409200
F	-5.75411300	2.06484400	-5.52482900
F	-5.94603100	1.75170300	-7.67262700
C	-3.35416100	5.17891700	-1.71647900
C	-3.06028000	4.12177700	-2.58742400
C	-3.57522500	4.12243100	-3.88828400
C	-4.37511600	5.19095600	-4.28456100
C	-4.65554300	6.24343300	-3.39135300
C	-4.14618800	6.26021400	-2.09129100
C	-2.00267000	3.83118100	-0.55039900
C	-2.24124700	3.17653600	-1.82970200
H	-3.34245400	3.31189800	-4.56988000

H	-4.79199300	5.20672500	-5.28595600
H	-5.28912500	7.06302600	-3.71994300
H	-4.36073900	7.06705800	-1.39830400
O	-2.78481900	5.00644800	-0.49059300
O	-1.81846000	2.04925200	-2.19870200
C	-1.81926300	3.06261200	0.74092600
H	-1.70248200	2.02025000	0.42381900
C	-2.97962000	3.13328400	1.72034900
C	-3.36141100	4.33946000	2.32984400
C	-3.66400600	1.96560900	2.08320200
C	-4.39744700	4.37266700	3.26311100
H	-2.85451200	5.26122600	2.06359800
C	-4.70127100	1.99472200	3.01740000
H	-3.37801400	1.01975700	1.63133000
C	-5.07311200	3.20085700	3.61138800
H	-4.67837900	5.31861700	3.71923400
H	-5.21609100	1.07399700	3.28005100
H	-5.88013400	3.22878200	4.33870900

21-int-*si*

P	1.81777500	2.29727200	-0.14304600
C	2.35997100	1.35072300	-1.64717600
H	3.45143500	1.34066900	-1.64717900
H	2.04299800	1.94027000	-2.51158100
C	1.89700800	-0.10807600	-1.78407500
H	2.15404700	-0.65746000	-0.87319300
C	2.64841600	-0.83761600	-2.94019100
H	2.28784500	-1.87028400	-2.91919200
C	2.38578500	-0.26002300	-4.32829100
H	2.76105900	0.76601000	-4.41249700
H	1.31496100	-0.27263700	-4.55174100
H	2.89728300	-0.86526200	-5.08349700
C	2.05538500	1.23831800	1.33262700
C	3.05977600	1.49531400	2.27722000
C	1.19975500	0.14019900	1.52805700
C	3.20676400	0.66836100	3.39274200
H	3.72554900	2.34145500	2.14943800
C	1.35155400	-0.68309600	2.64221900
H	0.41383700	-0.06432900	0.80939700
C	2.35605200	-0.42131900	3.57655200
H	3.98819500	0.88012300	4.11740700
H	0.68761800	-1.53228400	2.77463300
H	2.47550400	-1.06611500	4.44287000
C	3.17613800	3.58017000	-0.03367200
C	2.78062400	4.89195500	0.26769600
C	4.54144200	3.32213100	-0.25136100
C	3.71940700	5.92326900	0.34798000
H	1.72899800	5.10207500	0.44096000
C	5.48007000	4.35134000	-0.16961400
H	4.89068800	2.31651300	-0.46880200
C	5.07058300	5.65444500	0.12682100
H	3.39461800	6.93383500	0.58118600
H	6.53150800	4.13207600	-0.33611800
H	5.80318000	6.45498800	0.18619600
O	4.05145200	-0.78770300	-2.67319900
Si	5.17210200	-1.92165800	-2.15191400
C	4.66988700	-2.67292100	-0.48512200
C	3.40310300	-3.26960700	-0.31037700
C	5.53942100	-2.67455600	0.62329400
C	3.03402400	-3.85308400	0.90326900
H	2.66566600	-3.26255200	-1.10660000
C	5.17793700	-3.26510700	1.83608500
H	6.51290200	-2.19917000	0.54974600
C	3.92475400	-3.86242200	1.97771800
H	2.04579800	-4.29424200	1.00209000
H	5.87495100	-3.25322500	2.67075000
H	3.64186000	-4.32410900	2.92074700
C	6.73449500	-0.86187300	-1.97724700
C	6.71235500	0.48706800	-2.38206000
C	7.95475300	-1.36256600	-1.48103000
C	7.84567600	1.29840100	-2.28715800
H	5.79385000	0.89914900	-2.78856100
C	9.09101300	-0.55628600	-1.38068000
H	8.02938200	-2.39896600	-1.16227900
C	9.03931900	0.77936000	-1.78241100
H	7.79779900	2.33410400	-2.61605700
H	10.01642500	-0.97310800	-0.99096300
H	9.92325200	1.40780700	-1.70800200
C	5.43516900	-3.29104100	-3.48402200
C	4.13476000	-4.08356900	-3.74113400
H	4.31800300	-4.86893200	-4.48914200
H	3.33071800	-3.45035800	-4.13245300
H	3.76666600	-4.57765400	-2.83524800
C	6.52198600	-4.28360600	-3.01430900
H	6.65087300	-5.07880100	-3.76299500
H	6.25712700	-4.76637500	-2.06592400
H	7.49653000	-3.79790500	-2.89009700
C	5.89279000	-2.63387100	-4.80455200
H	6.83663400	-2.08998300	-4.68322500
H	5.14816100	-1.92616700	-5.18685400
H	6.04798500	-3.40130200	-5.57686000
N	0.45312000	-0.19969900	-1.96666700
H	-0.02970800	0.62375400	-2.31857000
C	-0.22354600	-1.35259100	-1.73674900
O	0.31523400	-2.38082200	-1.32178400
C	-1.75295300	-1.31096400	-1.95642000
H	-2.00172900	-2.31926600	-2.30119800
C	-2.51933900	-1.02932600	-0.63455700
C	-4.03721700	-0.94493400	-0.87001100
H	-4.55779000	-0.82697000	0.08797500

H	-4.40932000	-1.86470400	-1.33854600
H	-4.31773300	-0.10149900	-1.50653200
C	-2.21889100	-2.09701000	0.43048000
H	-2.71807300	-1.83487000	1.37057300
H	-1.14913000	-2.21001400	0.61850200
H	-2.59710200	-3.07622700	0.11158000
N	-2.16315000	-0.38241100	-3.00813500
H	-2.48639700	0.54764200	-2.75313300
C	-1.92865500	-0.71351500	-4.30915600
O	-1.28582000	-1.71544100	-4.62478300
C	0.20361800	2.84571900	-0.16594900
C	-0.58237500	3.05929900	1.11971500
H	-0.45706400	2.26338600	1.85892400
H	-0.36418900	4.00969000	1.64053800
C	-0.41364300	3.80622700	-1.17241300
H	-0.05286900	4.83979100	-1.06599800
C	-0.28278200	3.51327100	-2.66912100
O	-0.28819400	4.67087200	-3.34456500
O	-0.19788200	2.41008800	-3.18518000
C	-0.16107500	4.75649600	-4.81859400
H	-2.17443600	-0.05646200	-0.25528600
C	1.18900300	4.17704600	-5.25248500
H	2.00609400	4.65100200	-4.69725400
H	1.34395300	4.37629400	-6.31867700
H	1.22888700	3.09772600	-5.09479600
C	-0.21048500	6.26696000	-5.06133800
H	-0.12562000	6.47654700	-6.13272700
H	0.61146700	6.77064200	-4.54222300
H	-1.15543300	6.68476200	-4.69969100
C	-1.34023200	4.05414800	-5.49244100
H	-2.29242700	4.44964700	-5.12605700
H	-1.31377200	2.97855000	-5.31500400
H	-1.29569600	4.22880600	-6.57338500
C	-2.53241000	0.15678700	-5.38549700
C	-2.04350800	-0.03186000	-6.68405900
C	-3.59231800	1.04514800	-5.17406800
C	-2.59489600	0.66705400	-7.75551200
H	-1.24627100	-0.75087400	-6.83311400
C	-4.15326100	1.72769300	-6.25761800
H	-3.99138300	1.21176500	-4.18066900
C	-3.65987000	1.54553900	-7.54919000
H	-4.11045800	2.06593900	-8.38668000
C	-2.01566700	0.51430700	-9.13805900
C	-5.27115000	2.70912100	-6.03470200
F	-1.05283600	1.43778400	-9.37035300
F	-1.45461800	-0.69715000	-9.32075100
F	-2.95588500	0.68104900	-10.09481600
F	-4.80209300	3.97142700	-5.85843300
F	-5.99834000	2.41015100	-4.93243700
F	-6.12145200	2.75625100	-7.08105100

C	-3.28005100	5.73729900	-1.14531900
C	-3.85045200	4.71846700	-1.91892800
C	-5.00054900	4.95531300	-2.68455300
C	-5.55268900	6.23012100	-2.65833400
C	-4.96304900	7.24554100	-1.87829200
C	-3.82269100	7.02227100	-1.10887600
C	-1.93349000	3.94029600	-0.71710800
C	-3.03521500	3.53073500	-1.71606200
H	-5.43947500	4.15914100	-3.27446400
H	-6.44359100	6.45015600	-3.23827300
H	-5.41345600	8.23472800	-1.87285100
H	-3.37304100	7.80210700	-0.50392600
O	-2.17861900	5.35063400	-0.45979100
O	-3.18394600	2.40190600	-2.17952100
C	-2.03488000	3.10146800	0.59583200
H	-2.26330500	2.08955300	0.24339900
C	-3.11624700	3.52301100	1.56890000
C	-3.00322500	4.67498900	2.36374300
C	-4.26731900	2.73355900	1.70872200
C	-4.00707700	5.02007100	3.26916000
H	-2.12763800	5.30879100	2.26980400
C	-5.27507000	3.07776500	2.61097900
H	-4.37392700	1.83689600	1.10222800
C	-5.14719500	4.22360000	3.39685000
H	-3.89789000	5.91494700	3.87669800
H	-6.15679700	2.44855700	2.70082200
H	-5.92769600	4.49384900	4.10321500

22-int

P	0.62189800	-0.62658300	2.87116100
C	1.24494700	-0.32513800	1.11620700
H	2.32581200	-0.15907700	1.11986600
H	0.78325200	0.61509200	0.78829300
C	0.94292900	-1.44740100	0.11821400
H	1.31410300	-2.39620500	0.51673200
C	1.66897600	-1.23948000	-1.24579800
H	1.36883500	-2.08392600	-1.87419900
C	1.29571600	0.06427600	-1.95054400
H	1.68379300	0.92888700	-1.40207200
H	0.20842100	0.16215900	-2.04092200
H	1.72531400	0.08761700	-2.95692600
C	1.59596400	-2.10348800	3.39698600
C	0.91152000	-3.09503800	4.11756400
C	2.95552100	-2.30538000	3.10022500
C	1.56596500	-4.25471500	4.53888900
H	-0.14363600	-2.95963700	4.34112600
C	3.60791700	-3.46683500	3.51328600
H	3.51240800	-1.55623300	2.54430300
C	2.91402000	-4.44264000	4.23392300
H	1.01946800	-5.01255100	5.09386600

H	4.65311500	-3.61638500	3.25932800
H	3.42336400	-5.34941100	4.54902100
C	1.47325800	0.79150600	3.71508200
C	1.07635000	2.09696200	3.37046300
C	2.42271700	0.63587500	4.73638800
C	1.63279100	3.20821900	4.00305400
H	0.31981900	2.25175800	2.60364600
C	2.97137800	1.74913800	5.37822200
H	2.74111400	-0.35875100	5.03111300
C	2.58448600	3.03829000	5.01147000
H	1.31676000	4.20744700	3.71386500
H	3.70669500	1.60442000	6.16579900
H	3.01442000	3.90288800	5.51005200
O	3.07605600	-1.25561500	-1.02703400
Si	4.33838100	-2.28012400	-1.44061900
C	4.16104000	-3.95520100	-0.57442200
C	2.92760500	-4.63919300	-0.56790300
C	5.24796700	-4.57833000	0.06979900
C	2.79029300	-5.88517600	0.04761200
H	2.04036900	-4.20373300	-1.01813200
C	5.11766300	-5.82965100	0.67648800
H	6.21119100	-4.07844300	0.11504600
C	3.88677400	-6.48803800	0.66551800
H	1.82049900	-6.37529800	0.04573000
H	5.97714300	-6.28656600	1.16169200
H	3.78199800	-7.46033700	1.14068200
C	5.83911900	-1.32795900	-0.78323700
C	5.65528800	-0.07199100	-0.17320700
C	7.16166700	-1.80623500	-0.88128600
C	6.73289400	0.66558400	0.32177900
H	4.65145100	0.33298700	-0.09248200
C	8.24371500	-1.07376200	-0.38820800
H	7.36293400	-2.76674500	-1.34814900
C	8.03171500	0.16580600	0.21738100
H	6.55692700	1.63162700	0.78881300
H	9.25141100	-1.47180200	-0.47850200
H	8.87222600	0.73774200	0.60231500
C	4.45772100	-2.51457600	-3.35271500
C	3.16727400	-3.15037400	-3.91593900
H	3.27899800	-3.33119100	-4.99519000
H	2.29504400	-2.49902100	-3.78981800
H	2.94123400	-4.11380700	-3.44505300
C	5.64016600	-3.44875100	-3.69577400
H	5.67369700	-3.62492100	-4.78081600
H	5.55049700	-4.42593100	-3.20694500
H	6.60372500	-3.01095300	-3.41265400
C	4.68871500	-1.14910400	-4.03522300
H	5.61645300	-0.67633400	-3.69366000
H	3.86863300	-0.44881400	-3.83845600
H	4.76100000	-1.27569000	-5.12547500
N	-0.49806400	-1.60922600	-0.10037100
H	-1.12265500	-0.85157600	0.14588300
C	-1.01692000	-2.77554600	-0.57583800
O	-0.31832800	-3.75362400	-0.83975600
C	-2.53177400	-2.82301500	-0.86153700
H	-2.83304400	-3.84201800	-0.60653900
C	-2.83905400	-2.55710200	-2.36865600
C	-2.01776600	-3.44498600	-3.31660100
H	-2.29547700	-3.22846400	-4.35479900
H	-2.21747900	-4.50604800	-3.12741300
H	-0.94187400	-3.29444900	-3.21008800
C	-4.33843800	-2.74418400	-2.65305600
H	-4.55553700	-2.52353500	-3.70434300
H	-4.96495800	-2.09291500	-2.03631200
H	-4.64204100	-3.78069900	-2.45984200
N	-3.32713300	-1.93458100	-0.02249100
H	-3.33588200	-0.94060700	-0.23171700
C	-4.06149300	-2.39991100	1.03096900
O	-4.00971700	-3.55343000	1.44659300
C	-2.76203600	1.91368000	2.30270600
C	-2.78803800	1.17481300	3.37362500
H	-1.87865900	0.70579600	3.74622600
H	-3.71191400	0.97787800	3.91333400
C	-2.73154300	2.69356900	1.24178700
H	-2.61067900	3.76897900	1.34872500
C	-2.92181600	2.18718300	-0.13956900
O	-3.09201800	3.22121400	-0.97015100
O	-2.91519600	1.00663000	-0.47467800
C	-3.34327700	3.06522600	-2.42021500
H	-2.57859200	-1.50563700	-2.56947500
C	-3.54618200	4.51407800	-2.87129900
H	-4.40975800	4.95889500	-2.36675700
H	-3.71894100	4.54889700	-3.95184900
H	-2.66235700	5.11765800	-2.64152600
C	-4.61400800	2.24166700	-2.65419500
H	-4.91181300	2.32867700	-3.70500000
H	-5.43396800	2.61481200	-2.03343100
H	-4.45470100	1.18606100	-2.42809100
C	-2.11183400	2.44961400	-3.09003400
H	-1.94492700	1.42680300	-2.74666100
H	-1.21839100	3.04600000	-2.87625200
H	-2.25652700	2.43185100	-4.17593400
C	-4.98151800	-1.39217400	1.67705100
C	-5.30283200	-1.57692700	3.02647200
C	-5.55243500	-0.32588900	0.97948800
C	-6.14525900	-0.67599400	3.67462000
H	-4.88219200	-2.42829900	3.54981600
C	-6.41256300	0.56398400	1.62928700
H	-5.34690700	-0.18435700	-0.07474600
C	-6.70724500	0.40039900	2.98037500

H	-7.37241700	1.09094500	3.48540500
C	-6.37193600	-0.79294300	5.15830200
C	-6.96560000	1.72941900	0.85329600
F	-7.58754900	-0.33306900	5.52211100
F	-6.26249700	-2.06218100	5.59292400
F	-5.45408500	-0.05770800	5.83841800
F	-7.40433400	1.35183900	-0.36889100
F	-6.01298300	2.67446900	0.64072900
F	-7.98799600	2.33324600	1.48896600

23-ts

P	0.43045200	0.02144700	2.46124600
C	0.95255500	0.00787200	0.68036000
H	2.02203100	0.22310900	0.60406600
H	0.42959500	0.86387300	0.23532800
C	0.67211400	-1.27768300	-0.11673600
H	1.04559800	-2.14683400	0.42992000
C	1.42990500	-1.27713000	-1.47893100
H	1.15440200	-2.21682600	-1.96687300
C	1.06120100	-0.11062600	-2.39386500
H	1.42461000	0.83865000	-1.98642300
H	-0.02408300	-0.04499000	-2.52494500
H	1.51464800	-0.24380400	-3.38082500
C	1.33309800	-1.34634700	3.30341400
C	0.86473200	-1.74016900	4.56912400
C	2.45121100	-1.99925500	2.76029000
C	1.50141500	-2.75902800	5.27668800
H	0.00219400	-1.24401200	5.00743800
C	3.07895400	-3.02769700	3.46530600
H	2.84187100	-1.71678600	1.78852200
C	2.60669100	-3.40814200	4.72245900
H	1.12929400	-3.05017300	6.25519200
H	3.93072100	-3.53349300	3.02111400
H	3.09618300	-4.21077400	5.26729900
C	1.24035100	1.54156200	3.12585100
C	0.51632800	2.37512400	3.99165700
C	2.57877100	1.86020700	2.84341900
C	1.10994000	3.50870300	4.55088200
H	-0.51474500	2.13425300	4.23338100
C	3.17058300	2.99334300	3.40110300
H	3.17445500	1.21896900	2.19986300
C	2.43639700	3.82194500	4.25321200
H	0.53434900	4.14451900	5.21798400
H	4.20750800	3.22591000	3.17402300
H	2.89917000	4.70450400	4.68640200
O	2.83355600	-1.24251700	-1.21842700
Si	4.11043500	-2.29237400	-1.52337100
C	3.95325000	-3.84180500	-0.44259400
C	2.72548800	-4.53003100	-0.34419200
C	5.04740500	-4.36884900	0.27232200
C	2.60157800	-5.69057200	0.42290500
H	1.83191900	-4.16228400	-0.84009000
C	4.93070500	-5.53642800	1.03102600
H	6.00585400	-3.85875900	0.25133200
C	3.70573300	-6.20260800	1.10602100
H	1.63621400	-6.18519100	0.48561100
H	5.79600100	-5.92203900	1.56517400
H	3.61154100	-7.10963600	1.69791900
C	5.60266100	-1.24090600	-1.01392000
C	5.41078200	0.09768300	-0.62028200
C	6.92823500	-1.72004000	-1.03019600
C	6.48304400	0.91420400	-0.25482300
H	4.40422800	0.50466200	-0.61059000
C	8.00492000	-0.90955700	-0.66377100
H	7.13737300	-2.74248900	-1.33271000
C	7.78510700	0.41205600	-0.27319000
H	6.30168600	1.94503400	0.04036800
H	9.01489400	-1.31112400	-0.68602500
H	8.62169400	1.04536400	0.01066900
C	4.22591000	-2.77888900	-3.38709000
C	2.94161000	-3.49326200	-3.86361500
H	3.06185400	-3.82008600	-4.90676500
H	2.06483400	-2.83678000	-3.83481300
H	2.71767600	-4.38468500	-3.26657100
C	5.41383800	-3.74619600	-3.59589600
H	5.45878900	-4.05696900	-4.64965500
H	5.31819000	-4.65461100	-2.98969900
H	6.37484000	-3.27593100	-3.35981500
C	4.45515600	-1.51844000	-4.24899800
H	5.38044800	-0.99967800	-3.97384600
H	3.63225600	-0.80057000	-4.15419300
H	4.53097000	-1.79396000	-5.31093100
N	-0.75681100	-1.49293700	-0.32194400
H	-1.38511500	-0.68895200	-0.32662600
C	-1.24311500	-2.73671800	-0.60223100
O	-0.52098100	-3.73217900	-0.66539700
C	-2.74562200	-2.84886800	-0.92370700
H	-2.99557600	-3.90046100	-0.77250200
C	-3.03195200	-2.46720500	-2.40938900
C	-2.17236900	-3.29124500	-3.38102800
H	-2.39827700	-3.00812400	-4.41545500
H	-2.38059800	-4.36234900	-3.27166100
H	-1.10018800	-3.15024100	-3.21806400
C	-4.52194900	-2.64087000	-2.74001800
H	-4.71030400	-2.38043400	-3.78798500
H	-5.15569800	-2.00252300	-2.11739700
H	-4.83815800	-3.67977200	-2.58728400
N	-3.58915000	-2.09390900	-0.00221300
H	-3.45224600	-1.08760600	0.05196600
C	-4.57440700	-2.70144400	0.71616100

O	-4.75710500	-3.91765600	0.73147600
C	-1.85239400	0.63381300	2.43403100
C	-2.47568400	-0.26090000	3.18358000
H	-1.99075400	-1.13039800	3.61218200
H	-3.53971300	-0.13295400	3.37427000
C	-2.01660800	1.76799400	1.67817700
H	-1.76321700	2.74108100	2.08701100
C	-2.47379700	1.73351500	0.31561100
O	-2.62700500	2.98096700	-0.17557800
O	-2.71999200	0.70802400	-0.35727400
C	-3.11965000	3.26334600	-1.53177300
H	-2.77569100	-1.40388500	-2.52952100
C	-3.18144000	4.79447700	-1.54157200
H	-3.88268000	5.15555200	-0.78245000
H	-3.51378800	5.15326500	-2.52138600
H	-2.19569100	5.22152600	-1.33027300
C	-4.51684800	2.66974400	-1.74460200
H	-4.94436000	3.07008400	-2.67116600
H	-5.17991500	2.94146600	-0.91809300
H	-4.47868100	1.58218700	-1.82254900
C	-2.11126100	2.75566300	-2.56840500
H	-2.05229600	1.66576300	-2.55597200
H	-1.11608900	3.16852100	-2.36933500
H	-2.41775300	3.07643700	-3.57053700
C	-5.47945900	-1.79694900	1.52399600
C	-6.26389700	-2.41173700	2.50872200
C	-5.60503900	-0.42104400	1.31459600
C	-7.13798300	-1.65741400	3.28703800
H	-6.17740600	-3.48472500	2.63620500
C	-6.49141900	0.33059800	2.09420500
H	-5.02533700	0.08206900	0.54803900
C	-7.25789600	-0.27920700	3.08406700
H	-7.94507000	0.30596700	3.68331100
C	-7.92148300	-2.31212600	4.39552600
C	-6.61560300	1.80633400	1.82114700
F	-9.11214100	-1.70281800	4.59125500
F	-8.16307900	-3.61349700	4.14265400
F	-7.25316200	-2.25200000	5.57118500
F	-7.19045400	2.04211800	0.61742100
F	-5.40199500	2.40551500	1.78956500
F	-7.35779800	2.44070900	2.75119000

24-int

P	0.68728500	0.23760900	2.29669100
C	1.20664700	0.04595300	0.54375000
H	2.29536400	0.12861900	0.49073200
H	0.78505800	0.94092700	0.07206700
C	0.81058400	-1.22660700	-0.22620400
H	1.12765000	-2.11311300	0.32875500
C	1.58890000	-1.28050300	-1.57872000
H	1.28653500	-2.22040200	-2.04728800
C	1.27796700	-0.12106700	-2.52300400
H	1.67101100	0.82395700	-2.13311100
H	0.19853000	-0.01814800	-2.67371700
H	1.74288700	-0.29447600	-3.49841900
C	1.24566900	-1.15829600	3.32125700
C	0.72411900	-1.28767600	4.62192600
C	2.22500600	-2.06032000	2.87646000
C	1.16387800	-2.31762100	5.45101500
H	-0.02814600	-0.59265600	4.98174900
C	2.66110500	-3.08865800	3.71333500
H	2.65625000	-1.97497500	1.88550700
C	2.12928500	-3.21986100	4.99676100
H	0.75126100	-2.41524200	6.45101800
H	3.40957500	-3.78591100	3.35029800
H	2.46728300	-4.02365500	5.64483500
C	1.55212500	1.71177100	2.94041800
C	0.87571300	2.61074000	3.77957100
C	2.91790500	1.90590200	2.67848200
C	1.55417800	3.69673800	4.33359700
H	-0.18024000	2.47075900	3.98815300
C	3.59016500	2.99308500	3.23566700
H	3.46884500	1.21224200	2.04977300
C	2.90940000	3.89046100	4.06145000
H	1.02010200	4.39174800	4.97494400
H	4.64602700	3.13672900	3.02514400
H	3.43526100	4.73751900	4.49290100
O	2.98785500	-1.28881700	-1.29191500
Si	4.22779500	-2.40422600	-1.50285500
C	3.98077800	-3.87859600	-0.33537800
C	2.73739200	-4.54409600	-0.27229700
C	5.01815600	-4.37091900	0.48126800
C	2.54775000	-5.65459600	0.55335900
H	1.88200400	-4.19393800	-0.84285300
C	4.83613100	-5.48906800	1.29978100
H	5.98374300	-3.87415500	0.49057200
C	3.59972400	-6.13794900	1.33374800
H	1.57360100	-6.13488500	0.58292200
H	5.66040100	-5.85133800	1.90973600
H	3.45592800	-7.00905500	1.96816800
C	5.74681600	-1.38464700	-1.00860300
C	5.60447800	-0.01549800	-0.71016700
C	7.04867500	-1.92158400	-0.94871800
C	6.70122800	0.77557800	-0.36158600
H	4.61858300	0.43598700	-0.76627300
C	8.14963400	-1.13673700	-0.59872100
H	7.21891000	-2.97003300	-1.17817100
C	7.97867800	0.21621800	-0.30218400
H	6.55922300	1.83146300	-0.14319100
H	9.14038000	-1.58246900	-0.56105000

H	8.83432100	0.82976400	-0.03215900
C	4.36897500	-2.99656000	-3.33238600
C	3.07019100	-3.68407500	-3.80941500
H	3.20723600	-4.06956000	-4.83008300
H	2.22069100	-2.99302100	-3.84192200
H	2.79219800	-4.53365200	-3.17544500
C	5.52348700	-4.01748500	-3.45709200
H	5.58957500	-4.37737900	-4.49386800
H	5.37173200	-4.89299400	-2.81499700
H	6.49457700	-3.57452100	-3.20926400
C	4.66980200	-1.79081800	-4.24927500
H	5.61069600	-1.29826100	-3.97916300
H	3.87600500	-1.03593400	-4.20837500
H	4.75564800	-2.12169300	-5.29437900
N	-0.62582900	-1.36546200	-0.45113000
H	-1.25799200	-0.56380400	-0.36787900
C	-1.13525400	-2.60074100	-0.74257600
O	-0.41887700	-3.60073300	-0.82100900
C	-2.63744400	-2.70137900	-1.06284600
H	-2.86715400	-3.76697400	-1.00901000
C	-2.94145900	-2.19005900	-2.50476600
C	-2.06773500	-2.90196500	-3.54967200
H	-2.30854500	-2.53702600	-4.55470400
H	-2.24359500	-3.98435000	-3.53314500
H	-0.99804000	-2.74230700	-3.38248000
C	-4.42897300	-2.36597000	-2.84367300
H	-4.63048300	-2.00703200	-3.85967200
H	-5.06986400	-1.80481200	-2.15757700
H	-4.72220500	-3.42133100	-2.79187200
N	-3.47925600	-2.05100800	-0.06572100
H	-3.35962500	-1.04557500	0.06385400
C	-4.47681700	-2.73381000	0.56079300
O	-4.66067800	-3.94422700	0.43004300
C	-1.11028600	0.54896900	2.28613500
C	-1.92438100	-0.42936600	2.74348000
H	-1.55932300	-1.37131200	3.13810600
H	-2.99981000	-0.30100900	2.68554800
C	-1.43938300	1.73526100	1.53013100
H	-0.96119600	2.67890800	1.76220700
C	-2.29424000	1.71946400	0.41517400
O	-2.52438800	2.98122000	-0.06509300
O	-2.78258800	0.70373800	-0.16527000
C	-3.19053700	3.24445700	-1.33392000
H	-2.71146700	-1.11492100	-2.52745500
C	-3.17551500	4.77710000	-1.40252500
H	-3.72911600	5.20229000	-0.55900000
H	-3.63906500	5.12261100	-2.33303900
H	-2.14795100	5.15369100	-1.36291200
C	-4.63825700	2.73449300	-1.32608500
H	-5.17740300	3.13812500	-2.19173800
H	-5.15305000	3.06459100	-0.41875200
H	-4.67313600	1.64535400	-1.36829600
C	-2.38078300	2.65369200	-2.49616700
H	-2.36316900	1.56345100	-2.44066200
H	-1.35064200	3.02791600	-2.47091200
H	-2.82602300	2.94807300	-3.45377800
C	-5.39361500	-1.92911000	1.45748500
C	-6.27236500	-2.66043900	2.26670700
C	-5.43245200	-0.53126100	1.49730200
C	-7.16100800	-2.00572900	3.11596800
H	-6.24435300	-3.74219100	2.20304800
C	-6.32735400	0.11913400	2.35398800
H	-4.77495800	0.06868700	0.87427200
C	-7.19337300	-0.60950000	3.16597600
H	-7.88612700	-0.10016900	3.82527500
C	-8.05223000	-2.80305200	4.03211500
C	-6.36173500	1.62493000	2.35487100
F	-9.20554700	-2.15040200	4.30143300
F	-8.37405200	-4.00156500	3.50467000
F	-7.45157800	-3.04255900	5.22178100
F	-6.99383900	2.11029500	1.25941300
F	-5.11555600	2.14705600	2.34723300
F	-7.00477300	2.11956900	3.43485300

25-int

P	0.37841000	0.80985600	0.67344000
C	0.74102900	0.81608100	-1.13956200
H	1.83168000	0.76888100	-1.22211400
H	0.39156300	1.79389000	-1.47663400
C	0.18021800	-0.30313300	-2.03405000
H	0.47457400	-1.28571700	-1.64883500
C	0.82267500	-0.20638900	-3.45394500
H	0.33148700	-0.97433800	-4.05310200
C	0.64175100	1.14734500	-4.13587100
H	1.23521200	1.92360300	-3.64265800
H	-0.40963000	1.45311700	-4.12642200
H	0.97369600	1.08668200	-5.17719400
C	1.33431800	-0.60004100	1.35085300
C	0.97726500	-1.91734700	1.00956700
C	2.47107200	-0.38463600	2.14430700
C	1.75148600	-2.99193000	1.44310700
H	0.08202400	-2.10856000	0.42754600
C	3.23847000	-1.46550800	2.58281700
H	2.75902600	0.62290400	2.42299700
C	2.88384300	-2.76718900	2.22951500
H	1.47349700	-4.00079400	1.15560700
H	4.11399500	-1.28516300	3.20011900
H	3.48661500	-3.60607000	2.56629900
C	1.09601800	2.31339700	1.40568400
C	0.52384700	2.83239300	2.57547300

C	2.22663500	2.92925600	0.85075500
C	1.07957400	3.95938900	3.18047200
H	-0.35801600	2.36264500	2.99992500
C	2.77589700	4.05692800	1.46084700
H	2.67921700	2.54371600	-0.05724900
C	2.20374400	4.57336000	2.62505300
H	0.62715000	4.36162300	4.08239800
H	3.64737900	4.53418000	1.02170100
H	2.63117200	5.45431000	3.09583700
O	2.21843200	-0.48249000	-3.32600400
Si	3.12889200	-1.83383500	-3.74618600
C	2.51119500	-3.38186300	-2.83497400
C	1.17007400	-3.81088400	-2.93911000
C	3.37253100	-4.15507500	-2.03217100
C	0.72047600	-4.95980500	-2.28411400
H	0.44302600	-3.24178400	-3.51055000
C	2.93057100	-5.31284800	-1.38715100
H	4.40497100	-3.84835500	-1.89599100
C	1.60199400	-5.72186900	-1.51428900
H	-0.32025000	-5.25769700	-2.38253400
H	3.62507900	-5.89279300	-0.78383700
H	1.25609000	-6.62495000	-1.01708800
C	4.85678800	-1.34063300	-3.15069200
C	5.07781700	-0.04399000	-2.64862400
C	5.96891300	-2.20448400	-3.21274000
C	6.34213600	0.36949200	-2.22400000
H	4.24416500	0.64973700	-2.59816900
C	7.23561600	-1.79781300	-2.78797200
H	5.85441200	-3.21584900	-3.59405300
C	7.42579000	-0.50750300	-2.29086700
H	6.48180100	1.37838300	-1.84302500
H	8.07290600	-2.48867600	-2.84714000
H	8.41108300	-0.18792500	-1.96118500
C	3.13093800	-2.09773200	-5.65681900
C	1.71360500	-2.36866000	-6.20846100
H	1.76578500	-2.54643800	-7.29262000
H	1.03599100	-1.52099600	-6.05690100
H	1.25453900	-3.25500400	-5.75669500
C	4.02247100	-3.30865000	-6.01500700
H	4.00570300	-3.47495000	-7.10177100
H	3.67498900	-4.23244500	-5.53684600
H	5.06828600	-3.14859500	-5.73072500
C	3.70165300	-0.83566800	-6.34062900
H	4.72727400	-0.62488700	-6.01709500
H	3.09666900	0.05304300	-6.12664300
H	3.71796900	-0.97061300	-7.43184900
N	-1.28337600	-0.22616500	-2.09965300
H	-1.75832000	0.40651900	-1.44450600
C	-2.05999000	-1.09702200	-2.78490500
O	-1.63675400	-2.01642700	-3.49610400
C	-3.57792400	-0.86385600	-2.68495700
H	-3.80393500	0.09462300	-2.20982300
C	-4.27458600	-2.02022300	-1.91825600
C	-3.64017100	-2.25175500	-0.53887700
H	-4.17593300	-3.04463800	-0.00468500
H	-3.67721300	-1.34625400	0.07701000
H	-2.59177400	-2.55950200	-0.61920300
C	-5.78157300	-1.75867900	-1.79478600
H	-6.27984200	-2.61149500	-1.31939700
H	-6.24568000	-1.60033100	-2.77306400
H	-5.97695000	-0.87032800	-1.18195000
N	-4.06018300	-0.78610200	-4.06352800
H	-3.63110400	-1.45662900	-4.68961000
C	-4.69924300	0.30682500	-4.57070400
O	-5.00369500	1.28791700	-3.89557200
C	-1.38349300	0.63333500	1.13517100
C	-1.62825000	-0.25641900	2.14443800
H	-0.86990400	-0.87894300	2.59761900
H	-2.64396200	-0.37453600	2.51323200
C	-2.37354000	1.38634600	0.42400000
H	-3.40892600	1.16210000	0.66342900
C	-2.11780700	2.58877600	-0.28201900
O	-3.27497000	3.17248600	-0.71856800
O	-0.98556700	3.06668400	-0.51805000
C	-3.28516600	4.34886600	-1.58102900
H	-4.12070400	-2.92726100	-2.51984900
C	-2.68155700	5.55401800	-0.84734500
H	-3.20248800	5.71642100	0.10324800
H	-2.79566800	6.45879400	-1.45665200
H	-1.62171500	5.39284500	-0.64332700
C	-4.78171600	4.56133100	-1.83850300
H	-4.93637700	5.43964100	-2.47550600
H	-5.31441300	4.71944400	-0.89453800
H	-5.20814700	3.68542700	-2.33746500
C	-2.56377600	4.05677400	-2.90442200
H	-2.99531600	3.16447200	-3.37123600
H	-1.49557800	3.90235500	-2.74194500
H	-2.69705500	4.89964900	-3.59329300
C	-5.04370800	0.25112300	-6.04033200
C	-5.37137800	1.46266800	-6.65971200
C	-5.07642700	-0.92928200	-6.78966100
C	-5.69875200	1.49487400	-8.01366800
H	-5.35905500	2.36714600	-6.06234300
C	-5.41114500	-0.89298400	-8.14534000
H	-4.86108000	-1.88830100	-6.33076200
C	-5.71800700	0.31771400	-8.76486100
H	-5.96127500	0.34519900	-9.82053500
C	-6.09173800	2.79946600	-8.65984400
C	-5.49864000	-2.18085000	-8.92317900
F	-7.41882400	3.02579100	-8.54103300

F	-5.45789900	3.84808200	-8.09638600
F	-5.80599700	2.80795500	-9.98057700
F	-6.71703200	-2.75273400	-8.79711400
F	-4.59346000	-3.08581400	-8.48983600
F	-5.28544000	-1.98652200	-10.24147200

26-int

P	0.40924600	-3.56922200	-4.56409000
C	-0.52796600	-2.24529400	-5.47164900
H	-0.38113000	-2.47560100	-6.52554300
H	0.03193300	-1.32469500	-5.29083500
C	-2.02651800	-2.05715300	-5.18258600
H	-2.50335100	-3.00726800	-4.92376000
C	-2.77481500	-1.53268100	-6.44569100
H	-3.81009400	-1.38017600	-6.12838500
C	-2.21871500	-0.21998500	-6.99492600
H	-1.20142700	-0.35054600	-7.37871500
H	-2.20704500	0.55084900	-6.21644600
H	-2.84397400	0.13882600	-7.81832300
C	-0.71245100	-4.26136300	-3.28846400
C	-1.24067300	-5.55379800	-3.42529700
C	-1.10930300	-3.46817800	-2.19578400
C	-2.14894200	-6.04516900	-2.48562800
H	-0.95269500	-6.17705800	-4.26416000
C	-2.02555200	-3.96014100	-1.26709600
H	-0.70305500	-2.46993100	-2.07561000
C	-2.54411400	-5.24993500	-1.41095000
H	-2.55284000	-7.04601500	-2.60609100
H	-2.35178500	-3.33054100	-0.44447800
H	-3.26036100	-5.62831100	-0.68673500
C	0.80215500	-4.95489400	-5.67846600
C	1.88202700	-5.79104100	-5.36085100
C	0.01962200	-5.22414200	-6.80932100
C	2.17475300	-6.88607600	-6.17367400
H	2.49397200	-5.58032400	-4.48896100
C	0.32073000	-6.31969100	-7.61931700
H	-0.81480500	-4.58369600	-7.07319700
C	1.39677600	-7.15111600	-7.30265500
H	3.01684300	-7.52688300	-5.92722800
H	-0.28357200	-6.51095000	-8.50095400
H	1.63105400	-8.00194700	-7.93679800
O	-2.70736100	-2.52657200	-7.46764800
Si	-3.86048500	-3.54811400	-8.14663800
C	-4.39385600	-4.85540700	-6.88244900
C	-4.91470200	-4.46850400	-5.62905300
C	-4.28547300	-6.23644700	-7.13718500
C	-5.32269300	-5.41550900	-4.68765200
H	-4.98202100	-3.42122500	-5.34991500
C	-4.70109400	-7.18698500	-6.20187200
H	-3.86173400	-6.58339900	-8.07468100
C	-5.22679100	-6.77829900	-4.97532000
H	-5.70815800	-5.08063700	-3.72857000
H	-4.60908700	-8.24594500	-6.43124800
H	-5.55294900	-7.51653700	-4.24659400
C	-2.89134900	-4.32374300	-9.57726700
C	-1.62406900	-3.81484600	-9.92261400
C	-3.39574900	-5.38036000	-10.36346100
C	-0.89151700	-4.34033700	-10.98986400
H	-1.20675100	-2.99552100	-9.34581600
C	-2.66823000	-5.91025400	-11.43140500
H	-4.37247300	-5.80461500	-10.14623200
C	-1.41089400	-5.39162100	-11.74704000
H	0.08524600	-3.92696900	-11.22810300
H	-3.08473800	-6.72640700	-12.01649400
H	-0.84275400	-5.80163000	-12.57816800
C	-5.37824600	-2.57973400	-8.84277500
C	-6.16805100	-1.83241100	-7.74535200
H	-7.04459000	-1.34008700	-8.19150200
H	-5.57532200	-1.04783200	-7.26191200
H	-6.53703200	-2.50727600	-6.96545200
C	-6.34263200	-3.58081100	-9.52132000
H	-7.22871600	-3.04769100	-9.89499700
H	-6.69330100	-4.35109200	-8.82384800
H	-5.88163400	-4.08063700	-10.38027700
C	-4.89879700	-1.55878000	-9.89816400
H	-4.36297800	-2.04290600	-10.72234500
H	-4.23043900	-0.80522500	-9.46505800
H	-5.75946900	-1.02640200	-10.32830300
N	-2.22478900	-1.13604100	-4.06251400
H	-1.41938300	-0.60321500	-3.74032000
C	-3.40512400	-1.08109800	-3.38432300
O	-4.38249800	-1.76341700	-3.69018200
C	-3.52197400	-0.04136900	-2.24997000
H	-4.33550000	-0.40403300	-1.61859400
C	-3.89427000	1.36428100	-2.81411000
C	-5.11631700	1.30689300	-3.74423200
H	-5.36501600	2.31456900	-4.09612000
H	-5.98986500	0.90755600	-3.21578700
H	-4.94819600	0.67455600	-4.61992400
C	-4.14231000	2.36186600	-1.67287800
H	-4.36755100	3.35487600	-2.07805800
H	-3.27379600	2.45604200	-1.01446900
H	-4.99648400	2.04823900	-1.05997800
N	-2.34644400	0.04210500	-1.38746600
H	-1.45749300	0.32809100	-1.78912300
C	-2.36719200	-0.37580000	-0.08833700
O	-3.30904800	-0.97919500	0.42428100
C	1.94265000	-2.98585800	-3.73651900
C	2.16465000	-3.47100100	-2.47242500
H	1.44794500	-4.06890600	-1.92660900

H	3.11704600	-3.27938800	-1.98918000
C	2.82755400	-2.11952400	-4.43549800
H	3.69639300	-1.75788900	-3.89770600
C	2.80313700	-1.94246700	-5.84338000
O	3.83092300	-1.15609100	-6.28557200
O	1.95281400	-2.43498900	-6.61209900
C	4.06510300	-0.92384200	-7.70665100
H	-3.03136900	1.71892700	-3.39991500
C	4.34815600	-2.24921700	-8.42792700
H	5.17287700	-2.77767900	-7.93639700
H	4.64068700	-2.05270200	-9.46637300
H	3.46696300	-2.89266100	-8.42358600
C	5.31952800	-0.04224700	-7.70096700
H	5.60891600	0.21684400	-8.72549400
H	6.15498100	-0.56677000	-7.22537000
H	5.13326900	0.88214400	-7.14466200
C	2.88707200	-0.16829400	-8.33804300
H	2.68037800	0.74809600	-7.77449100
H	1.98936800	-0.78803000	-8.34448000
H	3.13451300	0.11169600	-9.36928400
C	-1.14600400	-0.03474700	0.73184900
C	-0.89698500	-0.80537400	1.87315600
C	-0.30363400	1.04262100	0.43895000
C	0.20223300	-0.52507600	2.68341600
H	-1.57837500	-1.61377700	2.11337600
C	0.78432700	1.33162900	1.26506700
H	-0.48741900	1.66961100	-0.42557600
C	1.04813000	0.54615600	2.38598100
H	1.90390100	0.75938600	3.01519100
C	0.52572800	-1.43469400	3.83958900
C	1.65031000	2.52763200	0.96272000
F	-0.58536200	-1.94270300	4.40798300
F	1.27634000	-2.49348700	3.43375400
F	1.23053100	-0.80232600	4.80005000
F	1.15961100	3.65051300	1.53557500
F	1.72238200	2.76652500	-0.36659000
F	2.90763600	2.36438100	1.41997000
C	3.19770500	1.58312300	-3.73842800
C	1.84121400	1.36754000	-4.01332600
C	1.21322700	2.07497600	-5.04457800
C	1.97228600	2.98081100	-5.78065600
C	3.33590300	3.17544600	-5.48840100
C	3.97576000	2.47861100	-4.46035600
C	2.54730100	0.12508500	-2.19146400
C	1.36671100	0.40856100	-3.02019400
H	0.15912500	1.91844200	-5.25529500
H	1.51260500	3.54833800	-6.58425200
H	3.90656800	3.89123500	-6.07356000
H	5.02405400	2.63010900	-4.22677400
O	3.63964300	0.83728400	-2.67733400
O	0.20787800	-0.02519400	-2.82966100
C	2.58075100	-0.66251700	-1.08754000
H	1.61888100	-1.10636500	-0.85280000
C	3.67070800	-0.95572600	-0.17019300
C	4.98923800	-0.47565500	-0.33183600
C	3.38712500	-1.75377100	0.95948200
C	5.97144100	-0.78418400	0.60538600
H	5.23382900	0.13868300	-1.18924500
C	4.37014200	-2.05182100	1.89731200
H	2.38044700	-2.13426600	1.10178900
C	5.66986400	-1.56864200	1.72291200
H	6.98080100	-0.40677000	0.46466200
H	4.11641100	-2.65487100	2.76447900
H	6.44169900	-1.80054200	2.45177300

27-ts-*re*

P	-0.48259200	0.87299700	0.46448800
C	-0.45506400	1.10982800	-1.38049400
H	0.46666300	1.65619800	-1.57396100
H	-1.26370500	1.80846600	-1.60575100
C	-0.52731200	-0.12386900	-2.29616000
H	-0.02510200	-0.98250400	-1.83571000
C	0.22624500	0.14552900	-3.63541500
H	0.08457100	-0.75482700	-4.23515500
C	-0.29292100	1.34655200	-4.42048800
H	-0.16649600	2.28131100	-3.86285700
H	-1.34966000	1.21005900	-4.66969500
H	0.26470900	1.44119000	-5.35745500
C	-0.78657500	-0.89868800	0.82016800
C	-2.03441800	-1.48369000	0.53050700
C	0.24488500	-1.69760800	1.33948600
C	-2.23531400	-2.84226800	0.77282600
H	-2.84608600	-0.88667000	0.12301000
C	0.03381700	-3.05779300	1.57001500
H	1.21511500	-1.26764200	1.55861900
C	-1.20592500	-3.63141900	1.29086600
H	-3.20327100	-3.28473300	0.55410600
H	0.84407200	-3.66444600	1.96297500
H	-1.37044600	-4.68991200	1.47340900
C	1.16102300	1.23562500	1.16386700
C	1.25553800	1.58609200	2.51890400
C	2.32465800	1.11506400	0.39215000
C	2.50578800	1.81761200	3.09235800
H	0.35648300	1.68541100	3.11951900
C	3.57195300	1.35063300	0.97209000
H	2.27276700	0.84876800	-0.65779400
C	3.66389100	1.70129200	2.32042900
H	2.57240200	2.09498800	4.14065100
H	4.46554800	1.26758500	0.36107600
H	4.63626200	1.88680400	2.76850900

O	1.61500000	0.33682100	-3.33812300
Si	2.94678000	-0.64917700	-3.63217200
C	2.83469600	-2.21763400	-2.56876800
C	1.73688200	-3.09731900	-2.69012200
C	3.82403400	-2.55052700	-1.62293600
C	1.64664400	-4.25726700	-1.91804600
H	0.91622500	-2.87691100	-3.36670700
C	3.74172800	-3.71460400	-0.85506600
H	4.67276700	-1.89045200	-1.47240100
C	2.65384700	-4.57551200	-1.00503300
H	0.78399800	-4.90823800	-2.03275500
H	4.52702700	-3.94575300	-0.13915400
H	2.58899800	-5.48469600	-0.41204600
C	4.37770600	0.46060700	-3.07004000
C	4.13987600	1.82375600	-2.80540300
C	5.70694400	0.00511200	-2.95149700
C	5.17259900	2.68652700	-2.42935300
H	3.13045100	2.21259000	-2.89745700
C	6.74349200	0.86191500	-2.57409000
H	5.94734700	-1.03510900	-3.15509200
C	6.47832800	2.20720600	-2.31005400
H	4.95575600	3.73335900	-2.23174200
H	7.75747100	0.47891200	-2.48991500
H	7.28386500	2.87670900	-2.01915700
C	3.16006100	-1.07091100	-5.50316000
C	1.97217200	-1.87244800	-6.07933300
H	2.17381000	-2.12538300	-7.13048600
H	1.03491500	-1.30639200	-6.06244100
H	1.80860800	-2.81470100	-5.54547500
C	4.43945800	-1.92153300	-5.67987500
H	4.56901100	-2.18147300	-6.74035000
H	4.38973900	-2.86107000	-5.11639600
H	5.34059000	-1.38205200	-5.36784800
C	3.31690900	0.23817600	-6.30828200
H	4.17578200	0.82800700	-5.96891000
H	2.42660700	0.87317900	-6.23361300
H	3.46981400	0.00922900	-7.37276200
N	-1.92401700	-0.47314000	-2.54474500
H	-2.64765500	0.09359100	-2.10767700
C	-2.28671400	-1.60920500	-3.20330700
O	-1.46596000	-2.41217300	-3.64541700
C	-3.81990600	-1.85217700	-3.30875900
H	-4.11449800	-2.29435300	-2.34649900
C	-4.17882600	-2.87709900	-4.42010500
C	-3.82895400	-4.30364700	-3.96174800
H	-4.03299600	-5.02031300	-4.76602300
H	-4.44228100	-4.59734600	-3.09762900
H	-2.77531500	-4.38448800	-3.68896600
C	-5.66144400	-2.80215400	-4.81216100
H	-5.89074700	-3.59260200	-5.53643400
H	-5.91746300	-1.84465600	-5.27249800
H	-6.31643700	-2.94860300	-3.94318600
N	-4.56732700	-0.59217800	-3.36658000
H	-4.80140500	-0.20484000	-2.44958500
C	-4.39097400	0.29682900	-4.38590200
O	-3.72268500	0.05737600	-5.39191100
C	-1.73350700	1.88405300	1.34757500
C	-2.57950200	1.23810700	2.19901800
H	-2.55907600	0.17191800	2.37618500
H	-3.28733500	1.81544400	2.78622100
C	-1.85724400	3.30697200	1.12200500
H	-2.39264100	3.83856100	1.90162100
C	-0.78806300	4.06885300	0.50292900
O	-0.86932100	5.38295900	0.79575900
O	0.08768100	3.57393500	-0.21649200
C	0.13784100	6.35385400	0.33992000
H	-3.57395300	-2.63436200	-5.29893900
C	1.50375800	6.00222400	0.94257100
H	1.42978300	5.92431400	2.03307600
H	2.22394800	6.79427900	0.70680000
H	1.87965500	5.05697500	0.54697800
C	-0.38858400	7.67043000	0.91927300
H	0.27802100	8.49575500	0.64640500
H	-0.44410500	7.61569600	2.01159600
H	-1.38999200	7.88448600	0.53358300
C	0.18220600	6.41947900	-1.19109700
H	-0.81496800	6.62996400	-1.58941700
H	0.54429200	5.48240500	-1.61642400
H	0.85262600	7.22982900	-1.50077000
C	-5.06184700	1.63755100	-4.20403700
C	-4.57158700	2.70749000	-4.96093100
C	-6.13423900	1.85197200	-3.33145600
C	-5.12026100	3.98086600	-4.81504200
H	-3.76464700	2.52021000	-5.66055800
C	-6.69051700	3.12615500	-3.20098500
H	-6.55732800	1.02993000	-2.76576000
C	-6.18191900	4.19775800	-3.93385600
H	-6.61472600	5.18548700	-3.82930700
C	-4.51874500	5.14467900	-5.55693000
C	-7.81112500	3.35338600	-2.21851700
F	-3.50191200	5.70752200	-4.84868600
F	-4.00326700	4.77649100	-6.74609700
F	-5.42081700	6.12258500	-5.78201500
F	-7.33953500	3.61913000	-0.98026300
F	-8.60872000	2.26845600	-2.11429600
F	-8.58737700	4.39884200	-2.57874100
C	-6.24260000	1.86326400	1.66214500
C	-5.86961300	0.75309600	0.89029700
C	-6.55208100	-0.45978500	1.03997400
C	-7.58732100	-0.52715700	1.96862000

C	-7.93838500	0.59992200	2.73675800
C	-7.27044200	1.81774100	2.59796400
C	-4.58364300	2.57059500	0.37318300
C	-4.76311000	1.19408900	0.03999100
H	-6.27303100	-1.32281400	0.44142500
H	-8.13520200	-1.45532900	2.10434600
H	-8.75165600	0.52236500	3.45319700
H	-7.53971400	2.69202400	3.18163800
O	-5.47400800	2.95628400	1.38194700
O	-4.14784100	0.51991200	-0.85001400
C	-3.68943300	3.47111900	-0.20572900
H	-3.20014800	3.03437400	-1.07166900
C	-3.90718200	4.92775500	-0.30012100
C	-4.45546400	5.69949300	0.74059300
C	-3.60013900	5.56856500	-1.51392400
C	-4.68651800	7.06254700	0.56525900
H	-4.70825600	5.22675000	1.68249000
C	-3.84670100	6.92911900	-1.69390700
H	-3.18908400	4.99016000	-2.33625500
C	-4.38738900	7.68440900	-0.65070100
H	-5.11068100	7.64209900	1.38115300
H	-3.62562500	7.38836400	-2.65338200
H	-4.58156800	8.74519300	-0.78536200

28-int-*re*

P	-0.83626300	0.86446200	0.09418300
C	-0.86403100	1.14420000	-1.74036400
H	0.03937000	1.71414900	-1.95462200
H	-1.69854800	1.81513400	-1.94481500
C	-0.93851100	-0.09310900	-2.65321300
H	-0.39465000	-0.93736500	-2.21216900
C	-0.24145300	0.19181800	-4.01795100
H	-0.38501900	-0.71365100	-4.60956500
C	-0.81611500	1.37747300	-4.78629300
H	-0.69224600	2.31775600	-4.23692800
H	-1.87775400	1.21435800	-4.99449600
H	-0.29664400	1.48226200	-5.74396600
C	-1.13300500	-0.89959900	0.46168300
C	-2.40885600	-1.45915300	0.25157000
C	-0.07822600	-1.70842400	0.91433500
C	-2.61046900	-2.81455300	0.51131300
H	-3.23297400	-0.84589000	-0.11686200
C	-0.29495200	-3.06470300	1.16021700
H	0.91048100	-1.29135600	1.06816200
C	-1.56032800	-3.61749900	0.96349600
H	-3.59635800	-3.24343000	0.35552300
H	0.52924600	-3.68421000	1.50030100
H	-1.72864500	-4.67307200	1.15908800
C	0.83318600	1.25423000	0.72118600
C	0.99625100	1.59058000	2.07416800
C	1.95686000	1.16998800	-0.11385300
C	2.27020300	1.84281400	2.58231700
H	0.13154900	1.66266600	2.72662000
C	3.22882100	1.42658600	0.40094300
H	1.85650900	0.91236700	-1.16229700
C	3.38665200	1.76238300	1.74664000
H	2.38813100	2.10649200	3.62956600
H	4.08879900	1.36991200	-0.25911800
H	4.37771800	1.96392100	2.14369500
O	1.15402100	0.41474100	-3.76953100
Si	2.48980100	-0.55345900	-4.10546800
C	2.44133800	-2.11529700	-3.02638900
C	1.35972100	-3.01859500	-3.11490200
C	3.45988800	-2.42000900	-2.10221500
C	1.31327600	-4.17510000	-2.33373100
H	0.51711900	-2.81897600	-3.77047100
C	3.42103000	-3.58002700	-1.32475300
H	4.29820300	-1.74148600	-1.97679800
C	2.34903800	-4.46560900	-1.44367200
H	0.46208800	-4.84469500	-2.42349000
H	4.22835100	-3.78962900	-0.62689500
H	2.31876300	-5.37255000	-0.84446600
C	3.92122700	0.58507500	-3.60313400
C	3.67273700	1.95049500	-3.36021200
C	5.25937300	0.14957300	-3.51329100
C	4.70386300	2.83431300	-3.03157600
H	2.65588800	2.32398200	-3.43368500
C	6.29441800	1.02732700	-3.18239500
H	5.50816400	-0.89116800	-3.70383600
C	6.01873800	2.37433100	-2.93859000
H	4.47932600	3.88286200	-2.85198100
H	7.31549300	0.65942200	-3.11965100
H	6.82314100	3.06018900	-2.68507700
C	2.64594900	-0.98514000	-5.97895200
C	1.44749800	-1.79987800	-6.51333900
H	1.61962200	-2.05830800	-7.56828700
H	0.50714600	-1.24043400	-6.47227900
H	1.30654500	-2.73977200	-5.96905000
C	3.92738600	-1.82490100	-6.19032600
H	4.02816600	-2.08687900	-7.25335100
H	3.90303900	-2.76297000	-5.62290000
H	4.83233100	-1.27617300	-5.90646400
C	2.76506700	0.32074500	-6.79606200
H	3.63149900	0.91855400	-6.49155500
H	1.87335700	0.94978200	-6.69288500
H	2.88105200	0.08664000	-7.86400900
N	-2.33437500	-0.46785000	-2.83927000
H	-3.05028200	0.07913100	-2.35296400
C	-2.69835000	-1.61839500	-3.47250000
O	-1.87780200	-2.40490600	-3.94616400

C	-4.23007200	-1.88851500	-3.50243300
H	-4.47525100	-2.29559700	-2.51100100
C	-4.62041300	-2.96027600	-4.55558600
C	-4.21912000	-4.36141400	-4.06265000
H	-4.44924900	-5.11323600	-4.82695800
H	-4.78058900	-4.63225200	-3.15677000
H	-3.15116600	-4.41208400	-3.84245700
C	-6.12128500	-2.92673500	-4.87690300
H	-6.36977700	-3.74474600	-5.56354000
H	-6.41727300	-1.98904900	-5.35356200
H	-6.73040000	-3.05541900	-3.97254900
N	-4.99396800	-0.63898400	-3.57212800
H	-5.13742100	-0.19987800	-2.64990400
C	-4.86126800	0.20500000	-4.63377000
O	-4.24580500	-0.07737200	-5.66393600
C	-2.04720400	1.86103600	1.04220500
C	-2.71774800	1.24334300	2.03051500
H	-2.59698900	0.19415100	2.27116700
H	-3.43338800	1.79794200	2.63168000
C	-2.39075600	3.30780000	0.73283100
H	-2.77250000	3.75252200	1.65462100
C	-1.19716000	4.12956400	0.28022100
O	-1.13483600	5.29366800	0.92450100
O	-0.41637900	3.74676800	-0.57924000
C	-0.11219100	6.32472400	0.61595700
H	-4.06377600	-2.73966400	-5.47124200
C	1.27757600	5.77914700	0.95797100
H	1.30522800	5.42123200	1.99298200
H	2.01739700	6.58120400	0.85640900
H	1.55772600	4.95916800	0.29439700
C	-0.50763200	7.46299500	1.55911500
H	0.17579700	8.30909800	1.43184000
H	-0.46329800	7.13578100	2.60319100
H	-1.52632000	7.80029600	1.34442000
C	-0.22844300	6.76253100	-0.84661300
H	-1.25202200	7.07538900	-1.07464600
H	0.05433700	5.95800300	-1.52746400
H	0.43827900	7.61527800	-1.01849200
C	-5.50967200	1.55985800	-4.47275100
C	-5.06093000	2.58541500	-5.31033000
C	-6.51574700	1.83061900	-3.53730100
C	-5.58190500	3.87361400	-5.18654100
H	-4.30545200	2.35313000	-6.05285700
C	-7.04681600	3.11669800	-3.43132700
H	-6.90019800	1.04550500	-2.89750300
C	-6.57743800	4.14627200	-4.24765700
H	-6.98871300	5.14476400	-4.15852200
C	-5.00639000	4.98739900	-6.01750500
C	-8.09250500	3.42152300	-2.38879500
F	-3.87288600	5.48769400	-5.45054900
F	-4.66525500	4.57501600	-7.25477200
F	-5.86208600	6.02256700	-6.14933500
F	-7.53757900	3.91270200	-1.26046700
F	-8.79793000	2.32456300	-2.04754500
F	-8.97223700	4.35209700	-2.82948200
C	-6.56579200	2.08995800	1.23327600
C	-6.28093500	0.96600000	0.43905200
C	-7.11666200	-0.15519500	0.51068000
C	-8.21109500	-0.11676700	1.37240100
C	-8.47447900	1.02271000	2.15654200
C	-7.65193500	2.14941200	2.09987800
C	-4.75360100	2.58110900	0.07149500
C	-5.07020600	1.28997800	-0.31697200
H	-6.90847200	-1.03275800	-0.09621200
H	-8.87502200	-0.97449100	1.44185500
H	-9.33673400	1.02799900	2.81800200
H	-7.84842300	3.03535500	2.69591700
O	-5.64090700	3.07411000	1.03937500
O	-4.43100200	0.51292000	-1.14969500
C	-3.59528500	3.40818200	-0.33880600
H	-3.22774000	2.94076300	-1.25651900
C	-3.92946100	4.85990800	-0.66345600
C	-4.41584300	5.74709800	0.30891400
C	-3.76396500	5.33114300	-1.97260400
C	-4.71915800	7.06792700	-0.02158600
H	-4.57724100	5.39882000	1.32372600
C	-4.07373800	6.65160600	-2.30890400
H	-3.40162100	4.65978600	-2.74696000
C	-4.54775200	7.52670200	-1.33072300
H	-5.10190100	7.73815100	0.74398000
H	-3.95131800	6.98207600	-3.33606100
H	-4.79172400	8.55447700	-1.58659200

29-ts-*re*

P	-0.78493100	0.90802500	0.23576700
C	-0.77024700	1.23549100	-1.61115900
H	0.15469900	1.77796500	-1.80672400
H	-1.57734600	1.94953300	-1.78962000
C	-0.88629000	0.04105700	-2.57195800
H	-0.36289400	-0.83481600	-2.17272800
C	-0.22023900	0.34249300	-3.94674200
H	-0.41358600	-0.54040900	-4.56179400
C	-0.77596900	1.57327200	-4.65750800
H	-0.55690200	2.48982400	-4.09895000
H	-1.85881400	1.48521200	-4.78998000
H	-0.32143800	1.66877300	-5.64846900
C	-1.01575300	-0.88936800	0.52112600
C	-2.22344700	-1.52502200	0.17742000
C	0.01837100	-1.65349400	1.08353700
C	-2.38883300	-2.89037800	0.40552200

H	-3.03205200	-0.95829800	-0.26976700
C	-0.15188800	-3.02158600	1.30253400
H	0.96086500	-1.18843700	1.34715800
C	-1.35457800	-3.64159600	0.96790200
H	-3.32602100	-3.36858200	0.13620100
H	0.66213400	-3.59871500	1.73107600
H	-1.48683500	-4.70631600	1.13919300
C	0.90589700	1.28526500	0.83455100
C	1.07875100	1.65237900	2.17754100
C	2.02919800	1.16405900	0.00301600
C	2.35672500	1.90396300	2.67811700
H	0.20951100	1.74512600	2.82176200
C	3.30539900	1.42055300	0.50707700
H	1.92495800	0.87516300	-1.03707900
C	3.47066300	1.79111700	1.84316700
H	2.48011600	2.19047100	3.71901200
H	4.16400500	1.33661500	-0.15224900
H	4.46511800	1.99203000	2.23250500
O	1.18585100	0.51762700	-3.74329600
Si	2.50421800	-0.45771100	-4.11766500
C	2.46795800	-2.03258000	-3.05963200
C	1.37100400	-2.91835900	-3.12452100
C	3.50836800	-2.36025500	-2.16832900
C	1.32730700	-4.07922300	-2.35004700
H	0.51393100	-2.70080800	-3.75448100
C	3.47314300	-3.52565800	-1.39909200
H	4.35994700	-1.69503600	-2.06172600
C	2.38320900	-4.39218800	-1.49214000
H	0.46180500	-4.73268500	-2.41843500
H	4.29606100	-3.75295200	-0.72542200
H	2.35418200	-5.30128000	-0.89618100
C	3.95816600	0.65578100	-3.62678600
C	3.72851700	2.00934300	-3.31199700
C	5.29513600	0.20833000	-3.60511100
C	4.77773800	2.87127800	-2.98263700
H	2.71216600	2.39067600	-3.32898800
C	6.34835500	1.06435100	-3.27528200
H	5.52817800	-0.82542500	-3.84664100
C	6.09185900	2.40048500	-2.96148500
H	4.56762900	3.91135100	-2.74545400
H	7.36822000	0.68787500	-3.26643800
H	6.91011100	3.06961300	-2.70749800
C	2.60697800	-0.86281700	-6.00106600
C	1.38259600	-1.65510200	-6.51017800
H	1.51794700	-1.90004700	-7.57370800
H	0.45122400	-1.08434200	-6.43200100
H	1.24644700	-2.60116900	-5.97531200
C	3.87080300	-1.71389500	-6.26517400
H	3.93227700	-1.96549900	-7.33374900
H	3.85626100	-2.65748900	-5.70661500
H	4.79061900	-1.17745400	-6.00715500
C	2.71636200	0.45408200	-6.80157700
H	3.59851600	1.03721200	-6.51414900
H	1.83573100	1.09154200	-6.66111100
H	2.79703400	0.23614500	-7.87629100
N	-2.29237200	-0.30711800	-2.74812300
H	-2.99644800	0.31658900	-2.35718700
C	-2.69053400	-1.50902400	-3.25510800
O	-1.89952200	-2.35977700	-3.65487500
C	-4.23077000	-1.72460300	-3.22542900
H	-4.50603300	-1.79780400	-2.16429400
C	-4.66042400	-3.04537900	-3.91150900
C	-4.25866100	-4.24564800	-3.03686600
H	-4.50701300	-5.18521000	-3.54419200
H	-4.80580100	-4.23560100	-2.08253000
H	-3.18644800	-4.24503100	-2.82883000
C	-6.16892800	-3.08006200	-4.19282300
H	-6.44494800	-4.05360000	-4.61475100
H	-6.47009400	-2.30971500	-4.90703500
H	-6.75447000	-2.94101600	-3.27374000
N	-4.92886600	-0.51902000	-3.68621600
H	-5.20039900	0.11931500	-2.94025800
C	-4.81436300	-0.07094400	-4.96664100
O	-4.27316200	-0.72351900	-5.85895100
C	-1.94944200	1.78675100	1.23860400
C	-3.10468300	1.18495600	1.74614500
H	-3.28735600	0.11930800	1.66326600
H	-3.58081600	1.63170100	2.61800500
C	-2.17268800	3.28072200	1.19863100
H	-2.40369500	3.63864800	2.20738500
C	-1.05120000	4.13523400	0.64739900
O	-0.88595100	5.24681400	1.36795500
O	-0.41419600	3.83583800	-0.35132800
C	0.07177600	6.30910500	0.98294800
H	-4.12382100	-3.11125300	-4.86150100
C	1.50044800	5.76126100	1.05222400
H	1.69915500	5.33149000	2.03991400
H	2.21188100	6.57838700	0.88678400
H	1.66743400	4.99197600	0.29637900
C	-0.16783000	7.36749300	2.06237200
H	0.48444800	8.23067200	1.89292000
H	0.04527300	6.96151300	3.05677500
H	-1.20876200	7.70518500	2.04259200
C	-0.28103500	6.85716600	-0.40361500
H	-1.33337700	7.15712800	-0.44007200
H	-0.09802300	6.11621200	-1.18361100
H	0.33471700	7.74007800	-0.60987500
C	-5.43810300	1.26932800	-5.28301700
C	-5.73281700	1.51242500	-6.62913800
C	-5.70021500	2.26249700	-4.33105200

C	-6.31349200	2.71802400	-7.01890200
H	-5.50247800	0.73933000	-7.35330800
C	-6.27033900	3.47313600	-4.73244900
H	-5.44950800	2.11650400	-3.28437100
C	-6.58801500	3.70440400	-6.07132900
H	-7.05127700	4.63788300	-6.37036500
C	-6.59483100	2.98042000	-8.47594300
C	-6.50203100	4.57296200	-3.73042600
F	-5.52627900	3.53354900	-9.09361800
F	-6.89264000	1.84555500	-9.14146100
F	-7.62939700	3.83512200	-8.63929200
F	-5.50291400	5.49444100	-3.77664000
F	-6.55527400	4.10873800	-2.46746800
F	-7.65021400	5.24017600	-3.97521200
C	-6.52000100	1.87573900	1.46963700
C	-6.34418100	1.07839600	0.32873200
C	-7.33829200	0.16598700	-0.04521000
C	-8.47937800	0.06940400	0.74616300
C	-8.62866400	0.87305200	1.89345800
C	-7.64962400	1.78925700	2.27886400
C	-4.50884400	2.41315000	0.67724500
C	-5.05773800	1.46670600	-0.25020500
H	-7.21615500	-0.44898300	-0.93295300
H	-9.26626600	-0.63066700	0.48040600
H	-9.53052300	0.78042700	2.49256300
H	-7.76052900	2.41522300	3.15831400
O	-5.46518800	2.71469600	1.67124500
O	-4.52132200	1.03744700	-1.32104500
C	-3.51282500	3.48344600	0.32326800
H	-3.22136500	3.26232600	-0.70971800
C	-4.04412600	4.90561200	0.36994900
C	-4.39332900	5.53106000	1.57692600
C	-4.18355400	5.63103400	-0.81982700
C	-4.86046400	6.84511700	1.58885500
H	-4.31264700	4.98561100	2.51198500
C	-4.66020400	6.94354700	-0.81288100
H	-3.93382400	5.16086500	-1.76580800
C	-4.99515700	7.55792100	0.39426600
H	-5.12798600	7.31132200	2.53379200
H	-4.77911800	7.47349200	-1.75381500
H	-5.36607400	8.57945900	0.40553200

30-int-*re*

P	-0.59576800	0.97964700	0.21204300
C	-0.48434700	1.17018100	-1.67915600
H	0.51241200	1.55454400	-1.90086800
H	-1.16828600	1.99551500	-1.89229300
C	-0.74230800	-0.02023100	-2.61134500
H	-0.29149900	-0.93199800	-2.20697300
C	-0.08783800	0.19354800	-4.01126800
H	-0.35250300	-0.68785000	-4.60132300
C	-0.56749400	1.44182600	-4.74727200
H	-0.30316400	2.35444500	-4.20259400
H	-1.65148700	1.41075200	-4.89386700
H	-0.09807100	1.49145000	-5.73469800
C	-0.58024100	-0.81624700	0.60911600
C	-1.59434400	-1.67080000	0.14563400
C	0.42775200	-1.35175500	1.42604500
C	-1.60079800	-3.02228800	0.49057900
H	-2.37625700	-1.28363300	-0.49677100
C	0.41918500	-2.70436200	1.76958800
H	1.22269100	-0.71516900	1.79712900
C	-0.59351300	-3.54306200	1.30398700
H	-2.39012700	-3.66707000	0.11430300
H	1.21020300	-3.10078400	2.40015200
H	-0.59620500	-4.59673300	1.56952700
C	1.04640900	1.61530000	0.77441600
C	1.12872900	2.21627500	2.03915400
C	2.22241100	1.45087500	0.02462700
C	2.35482900	2.66520200	2.53437300
H	0.22361700	2.32039700	2.62972800
C	3.44782800	1.89705700	0.52163300
H	2.20253100	0.96665900	-0.94605200
C	3.51597100	2.50983300	1.77492300
H	2.40118100	3.13214900	3.51482500
H	4.34389900	1.76808500	-0.07870600
H	4.47076900	2.85874300	2.15960800
O	1.33040900	0.27516600	-3.84121500
Si	2.58408100	-0.78214200	-4.20185700
C	2.47199400	-2.33329300	-3.11705200
C	1.31864600	-3.14654700	-3.13559100
C	3.51663200	-2.71673700	-2.25377900
C	1.22429900	-4.29157800	-2.34235200
H	0.45946600	-2.88269200	-3.74495800
C	3.42966000	-3.86638800	-1.46550300
H	4.41120900	-2.10558100	-2.18194400
C	2.28334400	-4.66047900	-1.51125500
H	0.31710400	-4.88905500	-2.37382400
H	4.25575200	-4.13666700	-0.81192400
H	2.21257200	-5.55544800	-0.89783300
C	4.10620600	0.25325300	-3.74795800
C	3.95486500	1.61550000	-3.42431200
C	5.41780800	-0.26353300	-3.75438100
C	5.05469600	2.42022200	-3.11504400
H	2.95977700	2.04951700	-3.41983000
C	6.52135400	0.53467200	-3.44533000
H	5.59121800	-1.30796300	-3.99995200
C	6.34261500	1.88125800	-3.12320600
H	4.90421900	3.46947800	-2.87256300
H	7.51978400	0.10458500	-3.45790300

H	7.20014500	2.50568900	-2.88531200
C	2.64064400	-1.23215100	-6.07783800
C	1.37102100	-1.97812300	-6.54456400
H	1.46840000	-2.24502300	-7.60712400
H	0.46689700	-1.36695000	-6.45176800
H	1.20863300	-2.90899600	-5.99038800
C	3.85808300	-2.14660300	-6.34655100
H	3.88189000	-2.43185900	-7.40827600
H	3.81627800	-3.07201600	-5.75949900
H	4.80670100	-1.64449900	-6.12687900
C	2.79541600	0.05948600	-6.91106900
H	3.70555300	0.61053300	-6.64809800
H	1.94519800	0.73774100	-6.77509400
H	2.85276200	-0.18650200	-7.98135300
N	-2.17982100	-0.26663300	-2.75528200
H	-2.83322400	0.43895700	-2.43356900
C	-2.67180100	-1.43157100	-3.26630800
O	-1.94815100	-2.35162900	-3.63742500
C	-4.22369500	-1.53305900	-3.26716900
H	-4.52676900	-1.60439300	-2.21356500
C	-4.73154800	-2.80784300	-3.98959800
C	-4.43717200	-4.05147800	-3.13307400
H	-4.75502100	-4.95888000	-3.65960800
H	-4.99005300	-4.01337500	-2.18310000
H	-3.37031000	-4.13995600	-2.91803100
C	-6.23236300	-2.72732100	-4.30232800
H	-6.56744000	-3.66727200	-4.75587200
H	-6.46574100	-1.91918200	-5.00074000
H	-6.82655900	-2.57159300	-3.39130300
N	-4.82989400	-0.27927100	-3.72472200
H	-5.22136300	0.30338000	-2.99410900
C	-4.62000100	0.21524600	-4.97397800
O	-3.99037000	-0.39959700	-5.83408200
C	-1.84194000	1.71553800	1.12070800
C	-3.11145300	1.04205700	1.55937500
H	-3.22815000	0.00010800	1.25440100
H	-3.24152300	1.06587700	2.65330600
C	-2.16248500	3.19794300	1.20412100
H	-2.23102300	3.52350500	2.25234300
C	-1.25341100	4.17929900	0.48476500
O	-1.01370900	5.25650900	1.24437200
O	-0.84312500	4.01663100	-0.65266900
C	-0.27195700	6.43073700	0.73575400
H	-4.17946800	-2.89267200	-4.92985500
C	1.16860900	6.03436400	0.39797700
H	1.64220100	5.54354300	1.25460600
H	1.74594300	6.93419200	0.15579700
H	1.20190400	5.35515100	-0.45510700
C	-0.31183200	7.38431100	1.93287800
H	0.20544900	8.31782700	1.68735300

H	0.17887300	6.93423100	2.80223100
H	-1.34634800	7.61883700	2.20310000
C	-1.01041700	7.03370300	-0.46414100
H	-2.05802900	7.22747900	-0.21072900
H	-0.97446600	6.36770400	-1.32788900
H	-0.54144500	7.98592900	-0.73717200
C	-5.23277400	1.56094900	-5.29454900
C	-5.28675700	1.90748200	-6.64914500
C	-5.72903500	2.45720000	-4.33972100
C	-5.84911000	3.11895100	-7.04807800
H	-4.88742000	1.20598200	-7.37291200
C	-6.28769400	3.67117000	-4.74602200
H	-5.68051000	2.23699900	-3.27851400
C	-6.35664800	4.00607000	-6.09887500
H	-6.81043600	4.94024800	-6.40925600
C	-5.86307600	3.49319900	-8.50864500
C	-6.78628200	4.65608800	-3.72139500
F	-4.70947100	4.09593000	-8.87302100
F	-6.01266100	2.41148900	-9.29990300
F	-6.86601900	4.35286300	-8.79425300
F	-5.84651000	5.59659500	-3.44016500
F	-7.10287700	4.05807600	-2.55454700
F	-7.87847400	5.31973000	-4.15437500
C	-6.43554600	1.65294100	1.76251200
C	-6.45015700	1.12132100	0.46451500
C	-7.61833100	0.55789700	-0.06783000
C	-8.75758200	0.53505000	0.72594000
C	-8.72502600	1.07149600	2.03035800
C	-7.57259700	1.63597900	2.57285900
C	-4.29309700	1.95956300	1.03521600
C	-5.11862900	1.32373100	-0.07530200
H	-7.62130200	0.15190400	-1.07551000
H	-9.68014000	0.10644300	0.34692100
H	-9.62939800	1.04527600	2.63246400
H	-7.55043400	2.05057600	3.57478200
O	-5.23737700	2.15955200	2.12617600
O	-4.68784900	1.05717200	-1.20601100
C	-3.61046800	3.28488400	0.58135400
H	-3.46009700	3.16422100	-0.49725500
C	-4.38009100	4.56693300	0.81135000
C	-4.61420400	5.08390400	2.09589300
C	-4.85543400	5.28913000	-0.29161400
C	-5.30213400	6.28527600	2.26507600
H	-4.26337400	4.54132000	2.96798400
C	-5.55112300	6.48819900	-0.12622400
H	-4.68104500	4.91101700	-1.29408200
C	-5.77492700	6.99181200	1.15550500
H	-5.47302600	6.66968200	3.26752900
H	-5.91710200	7.01657400	-1.00205300
H	-6.31456500	7.92545700	1.29139300

31-ts-*re*

P	1.95727600	3.53678000	0.46424500
C	2.27907500	3.04079400	-1.29501600
H	3.30226700	3.35081900	-1.52149900
H	1.60678300	3.65173700	-1.90299000
C	2.11403600	1.55275800	-1.64895100
H	2.48909700	0.91953800	-0.83670700
C	2.94900400	1.17086000	-2.90647300
H	2.73075300	0.11539800	-3.09033300
C	2.59649000	1.97376000	-4.15564200
H	2.85546000	3.03189200	-4.03605600
H	1.52877700	1.88789700	-4.38018100
H	3.15342400	1.59126100	-5.01614000
C	1.15086100	2.20819300	1.41322600
C	1.89398300	1.44390700	2.32690100
C	-0.22256700	1.95502700	1.24364700
C	1.27139800	0.43473100	3.06217400
H	2.95260200	1.63127300	2.46830400
C	-0.83586200	0.95448000	1.99650900
H	-0.80380400	2.52612800	0.52608800
C	-0.09318500	0.19346100	2.90289300
H	1.85559300	-0.15643500	3.76108900
H	-1.89966600	0.77133600	1.87535200
H	-0.57905300	-0.58574700	3.48355700
C	3.58268700	3.84868800	1.22895800
C	3.69524600	4.82672100	2.23015800
C	4.71346200	3.11227500	0.84171700
C	4.92897200	5.06101100	2.83548500
H	2.82481000	5.40583300	2.52212600
C	5.94554500	3.35607200	1.45061700
H	4.64821900	2.35480400	0.06759900
C	6.05334500	4.32805000	2.44666000
H	5.01251600	5.82309500	3.60493700
H	6.81639100	2.79050800	1.13407900
H	7.01446400	4.51921900	2.91594400
O	4.33298000	1.35813300	-2.59957900
Si	5.59794300	0.27143300	-2.35857100
C	5.29227200	-0.71313200	-0.76506300
C	4.09478400	-1.43818100	-0.58420200
C	6.22291700	-0.73474100	0.29280600
C	3.84580200	-2.15095300	0.59068300
H	3.31758900	-1.43771300	-1.34218900
C	5.98229400	-1.45475700	1.46540900
H	7.15052300	-0.17611000	0.21030300
C	4.79215300	-2.16790100	1.61676200
H	2.90761300	-2.68872000	0.69723400
H	6.72383000	-1.45458000	2.26076400
H	4.60277700	-2.72926100	2.52849900
C	7.07494800	1.44113900	-2.15287800
C	6.91555600	2.82016500	-2.39052800
C	8.36473900	0.99543300	-1.79670100
C	7.98427100	3.71149700	-2.27145700
H	5.93880100	3.19658400	-2.67806100
C	9.43727300	1.88179000	-1.67410800
H	8.54631100	-0.05987100	-1.60986300
C	9.24913200	3.24489500	-1.91039000
H	7.82859400	4.77033500	-2.46283700
H	10.41924700	1.50630400	-1.39739800
H	10.08269700	3.93636600	-1.81779700
C	5.86380400	-0.88350800	-3.87643200
C	4.64233900	-1.79004800	-4.14624500
H	4.85822200	-2.45879400	-4.99189500
H	3.74668600	-1.21909100	-4.41357800
H	4.39702100	-2.42286900	-3.28628500
C	7.08620200	-1.79295200	-3.61308000
H	7.22818500	-2.47874300	-4.46054100
H	6.95744100	-2.40692300	-2.71343900
H	8.01225000	-1.21755900	-3.50620800
C	6.14093900	-0.02620800	-5.13118200
H	7.02252900	0.61229500	-5.00387600
H	5.29266500	0.62221000	-5.37872400
H	6.32274300	-0.67586100	-5.99937300
N	0.70283300	1.25231900	-1.84042300
H	0.04291200	2.02506500	-1.90595100
C	0.22619700	-0.02490100	-1.77144300
O	0.96094200	-0.99608200	-1.61182400
C	-1.32094900	-0.12772900	-1.85052900
H	-1.69408100	0.29508800	-0.90839500
C	-1.81959900	-1.59294100	-1.93641400
C	-1.63076100	-2.29521200	-0.58043700
H	-1.93281200	-3.34639600	-0.65435400
H	-2.25568600	-1.82833600	0.19444500
H	-0.58879000	-2.26279900	-0.25564700
C	-3.29105200	-1.67043700	-2.36719000
H	-3.62768600	-2.71351000	-2.34808500
H	-3.44384500	-1.29044600	-3.38042700
H	-3.94041200	-1.10129900	-1.68840900
N	-1.84577300	0.77447300	-2.88129700
H	-2.14320400	1.68605600	-2.54381000
C	-1.57415000	0.59150400	-4.20410400
O	-1.00720200	-0.41125000	-4.63680800
C	1.02061300	5.09084200	0.49864500
C	1.38687300	6.02658600	-0.47732500
H	1.16867500	7.06609400	-0.27711900
H	2.21240500	5.82691300	-1.15469400
C	0.08933400	5.24113000	1.52912400
H	-0.13050300	4.41706600	2.19415500
C	-0.51202800	6.51419200	1.89252600
O	-1.25018800	6.36174800	3.03275900

O	-0.37343900	7.58539800	1.30600900
C	-1.86735900	7.50187100	3.71173500
H	-1.20753900	-2.10526900	-2.68338600
C	-0.78823900	8.49636700	4.16073200
H	-0.03569700	7.98830900	4.77477900
H	-1.24295800	9.28828300	4.76742700
H	-0.29397600	8.95112900	3.30079800
C	-2.53453000	6.84839000	4.92703900
H	-3.04108200	7.60549200	5.53547100
H	-1.79011600	6.34205000	5.55057700
H	-3.27470800	6.10780600	4.60653000
C	-2.92105500	8.16350100	2.81539200
H	-3.64700800	7.41900200	2.47277100
H	-2.45718300	8.62703800	1.94376600
H	-3.45910900	8.93168400	3.38389800
C	-2.05367100	1.66496600	-5.15252500
C	-2.22960600	1.29115500	-6.49032900
C	-2.29070200	2.99005400	-4.77464200
C	-2.67002800	2.22226200	-7.42853800
H	-2.00753500	0.26858600	-6.77442000
C	-2.71847300	3.92213200	-5.72418200
H	-2.11906800	3.31609800	-3.75382800
C	-2.91836400	3.54408400	-7.05036700
H	-3.25402200	4.26971900	-7.78129400
C	-2.92784200	1.78616300	-8.84788900
C	-2.90109800	5.35756100	-5.30327900
F	-2.04466000	0.84889300	-9.25074600
F	-4.16209000	1.25012900	-8.98408000
F	-2.84914800	2.82152100	-9.71268600
F	-1.70848600	5.98543300	-5.17987800
F	-3.51870400	5.44894500	-4.10595500
F	-3.63125200	6.05881200	-6.19578700
C	-3.52972000	5.97680100	-0.57270500
C	-3.32033900	4.59077300	-0.63414400
C	-4.32616200	3.71269000	-0.21170200
C	-5.51730300	4.25125400	0.26518200
C	-5.70516800	5.64671500	0.31518900
C	-4.71444100	6.53599400	-0.10477900
C	-1.51679300	5.73586300	-1.45864000
C	-1.99464800	4.40490500	-1.21306100
H	-4.17484600	2.63774400	-0.26284900
H	-6.31583400	3.59425100	0.59778900
H	-6.64701100	6.04258300	0.68529900
H	-4.85576800	7.61118600	-0.07809600
O	-2.45587400	6.67596700	-1.03749300
O	-1.41212700	3.30820500	-1.50039900
C	-0.33642900	6.08959200	-2.09311700
H	0.12943000	5.24557200	-2.59129800
C	-0.00450000	7.41117600	-2.64461000
C	-0.40442400	8.61585400	-2.03385200

C	0.75152600	7.47761500	-3.82971000
C	-0.07237800	9.83957500	-2.61060700
H	-0.94573700	8.58129300	-1.09597500
C	1.07861100	8.70384200	-4.40435600
H	1.06633000	6.55510500	-4.31189100
C	0.66432000	9.89147200	-3.79758200
H	-0.38535400	10.76057300	-2.12570500
H	1.65411800	8.73198600	-5.32592400
H	0.91948400	10.84997600	-4.24179300

31-ts-*si*

P	0.49834400	-3.54004300	-4.70071300
C	-0.46317300	-2.28486400	-5.68319900
H	-0.34264700	-2.60157700	-6.71803100
H	0.09132700	-1.34979400	-5.58815500
C	-1.95593000	-2.07369100	-5.37156300
H	-2.41838900	-2.99195000	-4.99541400
C	-2.74601400	-1.69184800	-6.65918600
H	-3.76471600	-1.48833500	-6.31996800
C	-2.20115800	-0.46233300	-7.38336000
H	-1.20968700	-0.65742900	-7.80584400
H	-2.13041800	0.39066400	-6.70025500
H	-2.86640000	-0.18589100	-8.20734400
C	-0.55566900	-4.15408000	-3.33886700
C	-0.97290500	-5.49311000	-3.30155200
C	-0.99391400	-3.26304600	-2.34200900
C	-1.81806700	-5.93395500	-2.28193700
H	-0.64978100	-6.19071800	-4.06571300
C	-1.84921300	-3.70874100	-1.33490800
H	-0.67712000	-2.22582900	-2.36575900
C	-2.25917000	-5.04428100	-1.30289600
H	-2.13928600	-6.97118200	-2.26541400
H	-2.21474400	-3.00903600	-0.58925400
H	-2.92804600	-5.38616400	-0.51790500
C	0.85628100	-4.97845900	-5.76157100
C	1.98367100	-5.76752600	-5.48893400
C	-0.00643000	-5.33525100	-6.80805800
C	2.24398900	-6.90048500	-6.25929100
H	2.65857700	-5.49194500	-4.68476400
C	0.26274200	-6.46814700	-7.57725200
H	-0.88042600	-4.73570100	-7.03815700
C	1.38566700	-7.25111500	-7.30354600
H	3.12220300	-7.50371100	-6.04684600
H	-0.40448600	-6.72639500	-8.39378000
H	1.59383900	-8.13141300	-7.90564400
O	-2.73175600	-2.80223700	-7.55844900
Si	-3.93371600	-3.87231500	-8.05374800
C	-4.37808100	-5.03720100	-6.62527500
C	-4.83536100	-4.51711400	-5.39513600
C	-4.25412600	-6.43653800	-6.72799800

C	-5.16737300	-5.35663400	-4.32997000
H	-4.91002100	-3.44554300	-5.23308400
C	-4.59383000	-7.27998900	-5.66764000
H	-3.87693000	-6.88190900	-7.64361300
C	-5.05717600	-6.74169900	-4.46633700
H	-5.50532000	-4.92083500	-3.39376200
H	-4.49215300	-8.35679400	-5.78049300
H	-5.32432700	-7.39658800	-3.64042000
C	-3.06630700	-4.80357000	-9.45794000
C	-1.83546800	-4.33257900	-9.95531500
C	-3.61951000	-5.94048400	-10.08189900
C	-1.18431300	-4.96793800	-11.01566100
H	-1.38241100	-3.45598500	-9.50247200
C	-2.97286000	-6.58146200	-11.14100000
H	-4.57117200	-6.34023700	-9.74173800
C	-1.75073900	-6.09644200	-11.61097000
H	-0.23469400	-4.58001100	-11.37570600
H	-3.42509300	-7.45726300	-11.59964500
H	-1.24655000	-6.59222800	-12.43661100
C	-5.49489300	-2.97557300	-8.75015900
C	-6.19723000	-2.08380700	-7.70230100
H	-7.11356900	-1.65696900	-8.13564000
H	-5.57471600	-1.24088300	-7.38305000
H	-6.49184400	-2.64349300	-6.80826900
C	-6.51048500	-4.04400300	-9.21955800
H	-7.41641100	-3.55160600	-9.60140000
H	-6.81766600	-4.70744100	-8.40241600
H	-6.11321300	-4.66223500	-10.03202500
C	-5.09589200	-2.10469600	-9.96218400
H	-4.62864100	-2.69754100	-10.75641700
H	-4.39337900	-1.31043900	-9.68368600
H	-5.98668400	-1.62097300	-10.38834900
N	-2.11523400	-1.03213900	-4.35767200
H	-1.27837300	-0.51968600	-4.07684000
C	-3.27901000	-0.87520500	-3.66794200
O	-4.28756600	-1.54392200	-3.89496000
C	-3.31835100	0.25886200	-2.62116500
H	-4.19431100	0.04224800	-2.00689600
C	-3.49320600	1.65063900	-3.29749800
C	-4.67088100	1.65663400	-4.28493500
H	-4.79140800	2.65487900	-4.72080000
H	-5.60666900	1.39399900	-3.77746300
H	-4.53291400	0.94610600	-5.10512100
C	-3.67656100	2.74989300	-2.24084100
H	-3.75963600	3.73140500	-2.72141500
H	-2.83617700	2.78618800	-1.54152200
H	-4.59171200	2.58185200	-1.65958100
N	-2.17334800	0.25784300	-1.71297700
H	-1.23697100	0.37233800	-2.10394100
C	-2.30563500	-0.07980000	-0.39771000

O	-3.34813200	-0.50403300	0.10080800
C	2.08493900	-2.96296200	-3.98673400
C	2.38329800	-3.31021200	-2.65280300
H	1.71145100	-3.92780400	-2.06917400
H	3.43292300	-3.40948700	-2.40059000
C	2.96166300	-2.16764500	-4.71826700
H	3.86672100	-1.83795400	-4.22031900
C	2.88212900	-1.89818200	-6.13614600
O	3.89649800	-1.09643300	-6.53504500
O	2.01791100	-2.35236300	-6.89343000
C	4.06197000	-0.67924900	-7.93220700
H	-2.56945600	1.86209900	-3.85824400
C	4.31124300	-1.90387200	-8.82136200
H	5.16215900	-2.48100700	-8.44247500
H	4.54927100	-1.57736000	-9.84045000
H	3.43350400	-2.55147000	-8.85476100
C	5.30909300	0.20810000	-7.86733600
H	5.54819700	0.59793100	-8.86273900
H	6.16919700	-0.36279900	-7.50198900
H	5.14292500	1.05159400	-7.18974100
C	2.84711800	0.13643200	-8.39209300
H	2.66071500	0.95944300	-7.69389200
H	1.95409600	-0.48781100	-8.45253700
H	3.04519100	0.56407800	-9.38189200
C	-1.07835400	0.10515500	0.46319200
C	-1.02346800	-0.62018700	1.65759600
C	-0.04175700	0.99398400	0.15514800
C	0.07343800	-0.49296400	2.50907900
H	-1.85338100	-1.27258200	1.90589800
C	1.04352000	1.13356400	1.02195300
H	-0.07223900	1.58615700	-0.75178800
C	1.11269400	0.38436800	2.19683600
H	1.96738000	0.47927600	2.85587900
C	0.16142800	-1.37040000	3.72927400
C	2.12898500	2.13456000	0.71776400
F	-1.03831500	-1.52129100	4.32655900
F	0.59178200	-2.61857300	3.40443200
F	1.02108500	-0.88455900	4.64744100
F	1.89097700	3.31812000	1.33013500
F	2.22318900	2.38872700	-0.60531700
F	3.33496800	1.70646800	1.14262500
C	3.54694100	1.25746100	-3.43174400
C	2.21349700	1.27734600	-3.86809600
C	1.80101400	2.23491600	-4.80214300
C	2.74289900	3.14359600	-5.27914800
C	4.07709800	3.09983600	-4.82866300
C	4.50356600	2.15319600	-3.89471800
C	2.54196000	-0.37065200	-2.31260600
C	1.53341200	0.20528800	-3.14633600
H	0.76790500	2.26336400	-5.13753600

H	2.44896700	3.90130300	-6.00004200
H	4.79141400	3.82410700	-5.21067300
H	5.52790800	2.11696300	-3.53863600
O	3.76889500	0.26555700	-2.51734700
O	0.30622100	-0.13690500	-3.18229000
C	2.35705700	-1.42835400	-1.42223500
H	1.30588200	-1.59757800	-1.21709700
C	3.26728400	-1.83085700	-0.33412100
C	4.65630400	-1.59581400	-0.35493500
C	2.72443400	-2.49453000	0.78293400
C	5.46046700	-2.00580300	0.70741200
H	5.10034500	-1.08211000	-1.19889900
C	3.52896600	-2.89544100	1.84735500
H	1.65527300	-2.68461300	0.82223000
C	4.90433800	-2.65466600	1.81266100
H	6.52944600	-1.81170700	0.67300700
H	3.07564900	-3.38321500	2.70523300
H	5.53619300	-2.96674300	2.63987700

32-int-*re*

P	2.42488700	3.77662300	0.35702700
C	2.53099400	3.07543900	-1.35532300
H	3.48900700	3.40124500	-1.76641800
H	1.74246800	3.55644900	-1.93829700
C	2.42122300	1.54474000	-1.48779200
H	2.94707300	1.05151800	-0.66116000
C	3.12081800	1.04680300	-2.78615000
H	2.96326100	-0.03381900	-2.80292300
C	2.56319500	1.64619100	-4.07286500
H	2.71569900	2.73097100	-4.11393600
H	1.49512600	1.42726000	-4.16269600
H	3.07263700	1.20812700	-4.93655900
C	2.02208400	2.52273700	1.61207800
C	3.03016100	2.03307100	2.45886500
C	0.70640200	2.03277500	1.71844200
C	2.72617100	1.05620700	3.40676000
H	4.04651800	2.40269000	2.38003600
C	0.41811400	1.06515000	2.68071000
H	-0.07197000	2.39581800	1.04493100
C	1.42120500	0.57567900	3.52114600
H	3.51161600	0.67385900	4.05148500
H	-0.59765200	0.69064300	2.77031400
H	1.18507900	-0.18158500	4.26378500
C	4.08347200	4.44234000	0.74089800
C	4.20887000	5.54999400	1.59484000
C	5.23701100	3.83957700	0.21181800
C	5.47245200	6.04723600	1.91147800
H	3.32302700	6.02838800	1.99973700
C	6.49798200	4.34489800	0.53108200
H	5.16700200	2.98174800	-0.44850600
C	6.61622800	5.44735800	1.37939400
H	5.56110300	6.90890400	2.56674400
H	7.38053500	3.87885800	0.10408300
H	7.59875400	5.84255400	1.62176800
O	4.51679100	1.35381600	-2.68437800
Si	5.85492400	0.36816700	-2.40633900
C	5.77240800	-0.34796600	-0.64937700
C	4.67497100	-1.13936100	-0.24580300
C	6.77708800	-0.10608800	0.30853500
C	4.59724300	-1.67288900	1.04243000
H	3.84286800	-1.32704300	-0.91765200
C	6.70746400	-0.64549800	1.59541400
H	7.62952200	0.51712300	0.05521900
C	5.61839000	-1.43631900	1.96456200
H	3.73281100	-2.27003500	1.31972300
H	7.50425800	-0.44592700	2.30809800
H	5.56423400	-1.86136600	2.96397800
C	7.27820000	1.61059700	-2.56007100
C	7.02808800	2.90576800	-3.05423000
C	8.61367500	1.29244500	-2.23804700
C	8.05465900	3.84056700	-3.20923800
H	6.01313200	3.17976700	-3.32556900
C	9.64447100	2.22305600	-2.38818600
H	8.86309100	0.30330400	-1.86308900
C	9.36689500	3.50257500	-2.87360600
H	7.82938900	4.83147300	-3.59582100
H	10.66368500	1.94669200	-2.13000800
H	10.16787400	4.22734600	-2.99493400
C	6.04285600	-1.01314300	-3.73717700
C	4.86457400	-2.01207800	-3.72832900
H	5.03874600	-2.79491200	-4.48071100
H	3.90895600	-1.53875600	-3.97775000
H	4.75167200	-2.51306700	-2.76088100
C	7.34339800	-1.80289700	-3.46135900
H	7.44926500	-2.61222200	-4.19783500
H	7.34296600	-2.26441000	-2.46644600
H	8.23469900	-1.17140700	-3.54586800
C	6.14091600	-0.36363300	-5.13551300
H	6.98712400	0.32918200	-5.20655100
H	5.23222600	0.19234700	-5.39337900
H	6.28084400	-1.13910400	-5.90212100
N	1.01981000	1.16421400	-1.45166500
H	0.31672200	1.90750300	-1.39369100
C	0.63257700	-0.13053200	-1.26886700
O	1.43731100	-1.05702900	-1.18073300
C	-0.90330500	-0.31674700	-1.12197500
H	-1.14731500	0.03672700	-0.11045700
C	-1.33545400	-1.80311200	-1.21497700
C	-0.93486800	-2.55188900	0.06807300
H	-1.19654500	-3.61340100	-0.01534700

H	-1.47075400	-2.14995800	0.94013900
H	0.13901200	-2.47775100	0.25064400
C	-2.84438300	-1.94598800	-1.45852500
H	-3.12199400	-3.00664500	-1.44551800
H	-3.14437000	-1.53338100	-2.42491900
H	-3.42846600	-1.44041100	-0.67815800
N	-1.63002900	0.60329300	-2.00427000
H	-1.76579300	1.53326100	-1.58846400
C	-1.48592300	0.51864500	-3.35751600
O	-0.87348900	-0.39540000	-3.91331600
C	1.27002200	5.17770000	0.40321200
C	1.18181500	6.06346200	-0.80420100
H	1.07055200	7.09257800	-0.46323000
H	2.08796300	6.00756000	-1.41898500
C	0.54197400	5.35540900	1.53302800
H	0.57613900	4.62228000	2.33120100
C	-0.25657500	6.56727700	1.87396000
O	-0.83823700	6.36538300	3.07222600
O	-0.31850000	7.59715900	1.22345100
C	-1.61305000	7.42083900	3.75792100
H	-0.80133300	-2.24909300	-2.05875400
C	-0.69340600	8.60655400	4.07186300
H	0.18487800	8.27188300	4.63540900
H	-1.23383000	9.33412500	4.68771600
H	-0.36196800	9.10253700	3.15786500
C	-2.05778200	6.71916500	5.04375200
H	-2.65054400	7.40444500	5.65875400
H	-1.19146200	6.39029100	5.62742500
H	-2.67135800	5.84294700	4.81158800
C	-2.82325300	7.82990000	2.91408900
H	-3.43338200	6.95715200	2.66307300
H	-2.51507600	8.31648200	1.98767000
H	-3.44274600	8.52742600	3.48981500
C	-2.12776400	1.61262500	-4.17837400
C	-1.79879000	1.65016000	-5.53802700
C	-3.01908400	2.56182300	-3.66377500
C	-2.33410900	2.63421600	-6.36749700
H	-1.12080400	0.89639000	-5.92237700
C	-3.55212000	3.54562400	-4.49952900
H	-3.31933400	2.54097600	-2.62291800
C	-3.21124900	3.58938800	-5.85144500
H	-3.62790000	4.35603900	-6.49402600
C	-2.00660600	2.62978500	-7.83766700
C	-4.45859500	4.60734400	-3.92899800
F	-0.73899500	2.22236200	-8.06616700
F	-2.81792800	1.79611200	-8.52691100
F	-2.14448600	3.85744600	-8.38685000
F	-3.75576800	5.68176400	-3.51047300
F	-5.16299900	4.15149800	-2.87448000
F	-5.34402000	5.05125000	-4.85215900
C	-3.36211900	5.63927100	-0.29380200
C	-3.03947900	4.28420000	-0.10157900
C	-3.98315300	3.42882300	0.48239300
C	-5.21889800	3.95482500	0.85384100
C	-5.51861300	5.31502200	0.64544100
C	-4.59090700	6.18274200	0.06507000
C	-1.32175300	5.37306900	-1.08015600
C	-1.69059200	4.11093100	-0.63562700
H	-3.74997300	2.37851800	0.63919500
H	-5.96694600	3.30920300	1.30651100
H	-6.49363700	5.69734300	0.93552700
H	-4.81337300	7.23075600	-0.10943200
O	-2.32719200	6.31431600	-0.86938500
O	-0.99461100	3.00503500	-0.69424600
C	-0.06073100	5.74043600	-1.77201000
H	0.22121400	4.83151000	-2.31657100
C	-0.18365300	6.86556800	-2.78968000
C	-0.51862800	8.17092700	-2.39897200
C	0.06087500	6.61215600	-4.14494500
C	-0.60851700	9.19090000	-3.34603800
H	-0.72021200	8.38121100	-1.35309400
C	-0.02810500	7.63284300	-5.09404400
H	0.31713900	5.60391100	-4.46392700
C	-0.36291700	8.92728900	-4.69603900
H	-0.87489100	10.19578900	-3.02825100
H	0.16300800	7.41396400	-6.14148900
H	-0.43398900	9.72465100	-5.43121800

32-int-*si*

P	0.50840600	-3.64554400	-4.82866300
C	-0.44127700	-2.38344400	-5.81367600
H	-0.35689300	-2.72914400	-6.84344000
H	0.13122500	-1.45765500	-5.74008700
C	-1.92021300	-2.12993500	-5.46725500
H	-2.39382600	-3.02876300	-5.05808100
C	-2.73211400	-1.76574200	-6.74629700
H	-3.73825400	-1.53594500	-6.38749600
C	-2.18309600	-0.56431900	-7.51274200
H	-1.20711200	-0.78857400	-7.95703300
H	-2.07764600	0.30153800	-6.85115300
H	-2.86403800	-0.29402400	-8.32577600
C	-0.49212700	-4.22968000	-3.42055100
C	-0.84579300	-5.58413700	-3.30935000
C	-0.94762000	-3.30744400	-2.46001700
C	-1.64751700	-6.00854300	-2.24934300
H	-0.50928500	-6.30456000	-4.04626500
C	-1.76112600	-3.74053200	-1.41305100
H	-0.67282800	-2.26052100	-2.54049700
C	-2.10879900	-5.08948800	-1.30642400
H	-1.91951700	-7.05703600	-2.17158900

H	-2.13975200	-3.01971500	-0.69427800
H	-2.74467200	-5.42131800	-0.49035400
C	0.81467800	-5.09279000	-5.89356500
C	1.99087300	-5.84208600	-5.74372900
C	-0.15110900	-5.49215300	-6.82984400
C	2.19733400	-6.97906000	-6.52460300
H	2.74949600	-5.53372600	-5.03117500
C	0.06426700	-6.62817000	-7.61074600
H	-1.06337400	-4.92100800	-6.96513200
C	1.23568700	-7.37229800	-7.45773300
H	3.11283100	-7.55189400	-6.40735300
H	-0.68245900	-6.91897400	-8.34285900
H	1.40149100	-8.25529300	-8.06871400
O	-2.75754600	-2.89958600	-7.61688400
Si	-3.98970900	-3.95730200	-8.06231800
C	-4.41575100	-5.08043400	-6.59539500
C	-4.81800100	-4.52089300	-5.36300300
C	-4.33232500	-6.48464700	-6.66973500
C	-5.13243000	-5.32749600	-4.26741100
H	-4.86388900	-3.44425200	-5.22352100
C	-4.65500200	-7.29485300	-5.57850300
H	-4.00190000	-6.96106100	-7.58793200
C	-5.06026700	-6.71760500	-4.37431800
H	-5.42779300	-4.86172300	-3.33125200
H	-4.58556400	-8.37627300	-5.66974900
H	-5.31314500	-7.34642300	-3.52407100
C	-3.16517100	-4.93398700	-9.46237400
C	-1.93872800	-4.49155200	-9.99584300
C	-3.74564600	-6.07744200	-10.04899700
C	-1.31833900	-5.15914000	-11.05463500
H	-1.46464500	-3.61117800	-9.57307900
C	-3.12908700	-6.75143000	-11.10571700
H	-4.69632400	-6.45535700	-9.68225600
C	-1.91102300	-6.29341200	-11.61198600
H	-0.37187200	-4.79181900	-11.44358500
H	-3.60236400	-7.63102400	-11.53488400
H	-1.43081200	-6.81428600	-12.43647900
C	-5.55204000	-3.05344800	-8.74243600
C	-6.22886600	-2.14648700	-7.69064100
H	-7.14738500	-1.71376600	-8.11318800
H	-5.59372700	-1.30755300	-7.38620800
H	-6.51501800	-2.69728600	-6.78839900
C	-6.58453000	-4.11801900	-9.18341600
H	-7.49452800	-3.62166700	-9.54992200
H	-6.88021300	-4.77322900	-8.35532400
H	-6.20897900	-4.74510600	-9.99946300
C	-5.16601500	-2.19851400	-9.96974300
H	-4.71340900	-2.80256100	-10.76414700
H	-4.45565500	-1.40494900	-9.71009000
H	-6.06025900	-1.71473300	-10.38830900

N	-2.02403000	-1.05993500	-4.47967300
H	-1.15446100	-0.59349000	-4.19708300
C	-3.16697300	-0.87090000	-3.76290200
O	-4.19732800	-1.51581600	-3.96558900
C	-3.14709100	0.26274800	-2.71732500
H	-4.02582700	0.08570200	-2.09428800
C	-3.26753300	1.66033500	-3.39477600
C	-4.45396500	1.71654600	-4.36990300
H	-4.53252700	2.71661700	-4.81132300
H	-5.39608700	1.50140600	-3.85162300
H	-4.35723300	0.99519600	-5.18686100
C	-3.39020500	2.76791100	-2.33818700
H	-3.43483400	3.75169200	-2.81962900
H	-2.54021600	2.76720700	-1.64984500
H	-4.30495500	2.64200600	-1.74560100
N	-1.99084500	0.21037800	-1.82558000
H	-1.04930000	0.24270200	-2.23969400
C	-2.13503600	-0.07284900	-0.50058900
O	-3.19726500	-0.42626600	0.01569700
C	2.12009800	-3.05845100	-4.18754800
C	2.50184900	-3.29868500	-2.74998900
H	1.93715500	-4.11682400	-2.29619800
H	3.55765300	-3.58657500	-2.73258300
C	2.99032800	-2.33351400	-4.93195600
H	3.90616200	-1.99810100	-4.45478500
C	2.88235200	-1.97956000	-6.36324400
O	3.85458800	-1.12111300	-6.68832400
O	2.03914000	-2.43458100	-7.12591900
C	3.97032400	-0.51459100	-8.03272000
H	-2.34091100	1.82958100	-3.96408500
C	4.23628000	-1.60720300	-9.07295800
H	5.11634100	-2.19650900	-8.79274400
H	4.43603000	-1.14189800	-10.04475800
H	3.38001500	-2.27625800	-9.17431100
C	5.18313200	0.40528100	-7.87307900
H	5.37561000	0.93489700	-8.81218900
H	6.07548000	-0.17369500	-7.61322300
H	5.00617100	1.14202400	-7.08340900
C	2.70778500	0.29756400	-8.33859000
H	2.51564300	1.01503500	-7.53385300
H	1.83631700	-0.34947100	-8.45798100
H	2.85393300	0.85694900	-9.26942200
C	-0.90335200	0.09510800	0.35971600
C	-0.93167900	-0.49475700	1.62665000
C	0.20648100	0.86591500	-0.01141000
C	0.14526900	-0.34199700	2.50061600
H	-1.81531600	-1.05317600	1.91576300
C	1.27092300	1.03193100	0.87453900
H	0.24355000	1.35577000	-0.97605900
C	1.24966500	0.42485100	2.13149500

H	2.07991500	0.55571800	2.81564400
C	0.12375300	-1.06332600	3.82090100
C	2.48016100	1.84301500	0.48247500
F	-1.09088400	-1.00668300	4.40529500
F	0.41419000	-2.38277900	3.66362800
F	1.02339200	-0.56449900	4.69341700
F	2.85886600	2.67015100	1.48664600
F	2.25192500	2.60360800	-0.60655400
F	3.53937900	1.05228300	0.21088800
C	3.70154900	0.97138000	-3.32050000
C	2.36543500	1.12675600	-3.73251400
C	2.01909000	2.21883000	-4.53879300
C	3.01797400	3.11915500	-4.90690400
C	4.34792700	2.94164600	-4.47661800
C	4.71156000	1.85967400	-3.67133400
C	2.58397800	-0.72287400	-2.45508900
C	1.63319500	-0.00368700	-3.16557700
H	0.98988700	2.35739600	-4.86004300
H	2.77018700	3.97780600	-5.52566900
H	5.10502100	3.66439300	-4.76897400
H	5.73127100	1.71540400	-3.32797200
O	3.85575800	-0.15240800	-2.55872300
O	0.37018100	-0.31094800	-3.29430500
C	2.35608500	-2.02193900	-1.77765600
H	1.29866600	-1.99570200	-1.50238800
C	3.16038200	-2.29872700	-0.51472100
C	4.56324200	-2.25461700	-0.50314400
C	2.49690400	-2.65784700	0.66572700
C	5.27578100	-2.55141600	0.65879200
H	5.09944300	-1.96487500	-1.40109300
C	3.20708800	-2.95331800	1.83180500
H	1.41049000	-2.70052800	0.67892600
C	4.60118400	-2.90202100	1.83075300
H	6.36176100	-2.50258300	0.64955900
H	2.66506200	-3.20977300	2.73710800
H	5.15831000	-3.12977800	2.73577500

33-ts-*re*

P	2.61356600	3.85076400	0.34783000
C	2.77723000	3.11856700	-1.36953000
H	3.77210300	3.38371800	-1.73570200
H	2.04721900	3.65339500	-1.98633100
C	2.59360300	1.60060800	-1.52684900
H	3.10264200	1.07281500	-0.71262000
C	3.24206100	1.07814200	-2.84360000
H	3.03732500	0.00413800	-2.86298800
C	2.67946300	1.71175800	-4.11289800
H	2.89697900	2.78489700	-4.15384100
H	1.59697800	1.56199300	-4.17178600
H	3.13373000	1.24655700	-4.99299300
C	2.19638100	2.58314600	1.59814800
C	3.16215000	2.15307200	2.52150900
C	0.89947700	2.04039700	1.65749200
C	2.83957600	1.19557700	3.48454400
H	4.16656500	2.55915700	2.49293300
C	0.58317300	1.09337100	2.63067200
H	0.14272100	2.35585300	0.94698000
C	1.55092900	0.66665500	3.54294400
H	3.59974000	0.86768300	4.18746000
H	-0.42253000	0.68524000	2.67353600
H	1.29910900	-0.07574100	4.29520400
C	4.31518800	4.42778900	0.73371900
C	4.47844300	5.65025400	1.40154000
C	5.44699300	3.67534100	0.37731000
C	5.75917500	6.11312700	1.70853400
H	3.60244700	6.23755900	1.66234900
C	6.72418100	4.14362800	0.68598200
H	5.34420200	2.72349700	-0.13496700
C	6.88067400	5.36263500	1.35070300
H	5.87862800	7.06394700	2.22041000
H	7.59222700	3.55928400	0.39594300
H	7.87683700	5.72871700	1.58417800
O	4.64820100	1.32495900	-2.78591700
Si	5.97779700	0.32827500	-2.52353600
C	5.92199100	-0.37287700	-0.75995400
C	4.81021400	-1.12103400	-0.31602400
C	6.96302500	-0.15787700	0.16511900
C	4.74911400	-1.63421700	0.98122100
H	3.95526600	-1.29050700	-0.96354500
C	6.91005900	-0.67758600	1.46088600
H	7.83015100	0.42971300	-0.12191300
C	5.80288000	-1.42109700	1.87180100
H	3.87186700	-2.19582200	1.29070300
H	7.73308600	-0.49794100	2.14858400
H	5.75988000	-1.82746000	2.87942300
C	7.41536600	1.54733800	-2.71059100
C	7.16843200	2.85834300	-3.16153300
C	8.75593000	1.20022800	-2.44432700
C	8.20391000	3.78010300	-3.33115300
H	6.14863700	3.15499000	-3.38612500
C	9.79579400	2.11787800	-2.61011700
H	9.00240300	0.19915500	-2.09984400
C	9.52165000	3.41300700	-3.05379100
H	7.98073800	4.78489100	-3.68134900
H	10.81901100	1.81960900	-2.39560000
H	10.32935200	4.12845300	-3.18574700
C	6.10990600	-1.06767600	-3.84526600
C	4.91337900	-2.04315500	-3.79184400
H	5.04832000	-2.83581800	-4.54202500
H	3.96029600	-1.55182600	-4.01595600

H	4.82089800	-2.53242800	-2.81616500
C	7.40249500	-1.87938800	-3.60048600
H	7.47053000	-2.70005700	-4.32906600
H	7.42473800	-2.32799500	-2.59988100
H	8.30214600	-1.26604500	-3.72099700
C	6.17753300	-0.43236000	-5.25170200
H	7.03527700	0.24197200	-5.35512300
H	5.27290300	0.14099900	-5.48482200
H	6.27654700	-1.21666800	-6.01590800
N	1.17807500	1.26032200	-1.47068800
H	0.49560100	2.01388400	-1.50331300
C	0.75362800	-0.00786900	-1.19968300
O	1.52887000	-0.94948100	-1.05490300
C	-0.78527200	-0.13859700	-1.03101800
H	-1.02851000	0.37355500	-0.09049900
C	-1.24933100	-1.61152000	-0.89256100
C	-0.83987600	-2.16700900	0.48246900
H	-1.11410000	-3.22564800	0.55940600
H	-1.36016900	-1.63497300	1.29222900
H	0.23690600	-2.08174500	0.64169600
C	-2.76555000	-1.75258600	-1.08598700
H	-3.06685000	-2.79118300	-0.90606500
H	-3.07724700	-1.48673600	-2.09914700
H	-3.32245100	-1.12065900	-0.38084900
N	-1.48800600	0.64452600	-2.05323900
H	-1.75185800	1.58390200	-1.76715300
C	-1.41988800	0.32301200	-3.37548800
O	-0.88440500	-0.70628600	-3.78525000
C	1.53431000	5.21896400	0.40556200
C	1.54371500	6.22665700	-0.70798800
H	1.66011800	7.23923500	-0.31580700
H	2.33638500	6.07468400	-1.45058600
C	0.38556400	5.30982500	1.21437500
H	0.07451800	4.45013200	1.80097300
C	0.03868800	6.58686600	1.92727700
O	-0.90785800	6.33786600	2.85364300
O	0.56759300	7.67066900	1.74353000
C	-1.34558700	7.36291800	3.82092800
H	-0.74044000	-2.19055800	-1.66761900
C	-0.16742000	7.73142300	4.72999600
H	0.24469700	6.83452700	5.20550900
H	-0.51013400	8.40875600	5.52032900
H	0.62282900	8.22920500	4.16433000
C	-2.43699400	6.63018200	4.60618300
H	-2.84439600	7.28463200	5.38425600
H	-2.03263100	5.73207200	5.08459700
H	-3.25209200	6.32865300	3.94061800
C	-1.92358200	8.58929000	3.10634200
H	-2.70494100	8.28762600	2.40304300
H	-1.15183300	9.13225500	2.56042700
H	-2.37209800	9.25927100	3.84918500
C	-2.09824700	1.26386200	-4.34359900
C	-2.44914400	0.72939600	-5.58710400
C	-2.36259800	2.61303500	-4.07494900
C	-3.08965400	1.52106600	-6.54040500
H	-2.20417700	-0.30718200	-5.78963800
C	-2.99031400	3.40204100	-5.03937600
H	-2.06185300	3.06560700	-3.13581300
C	-3.36611100	2.86048900	-6.27029200
H	-3.85940900	3.47837300	-7.01227600
C	-3.52965700	0.90786000	-7.84502600
C	-3.23575700	4.86771400	-4.79530600
F	-2.65278200	-0.02020600	-8.28052000
F	-4.72766000	0.29267400	-7.72183000
F	-3.66026200	1.83727500	-8.81708200
F	-2.40763000	5.63132100	-5.55275600
F	-3.03540200	5.21299600	-3.50817000
F	-4.49580100	5.22247400	-5.12676000
C	-3.05600800	5.85037600	0.25177000
C	-2.87162800	4.47875900	0.02598100
C	-3.91869000	3.58021700	0.26828600
C	-5.12583900	4.08412300	0.74134500
C	-5.28989300	5.46676700	0.95923000
C	-4.25864800	6.37531900	0.71881500
C	-0.94398400	5.66126200	-0.44375800
C	-1.52433300	4.33742600	-0.51311400
H	-3.78517700	2.51670700	0.08859100
H	-5.95491600	3.41134200	0.94049800
H	-6.24753200	5.83763200	1.31443500
H	-4.38824700	7.44254600	0.86437800
O	-1.94409900	6.57433100	-0.05709400
O	-0.96126800	3.28872800	-0.95171400
C	0.11858300	6.13348200	-1.41751100
H	0.20071800	5.31984900	-2.14874000
C	-0.22764200	7.40610200	-2.17335500
C	-0.26648800	8.65338100	-1.53091100
C	-0.49669900	7.35119600	-3.54569200
C	-0.56637900	9.81060400	-2.24957600
H	-0.07098000	8.71508300	-0.46403200
C	-0.80130400	8.50795400	-4.26578100
H	-0.48105800	6.39411300	-4.05793000
C	-0.83421600	9.74362500	-3.61944300
H	-0.59352600	10.76865400	-1.73631600
H	-1.01740700	8.43689100	-5.32848200
H	-1.06909300	10.64720800	-4.17613800

33-ts-*si*

P	0.84449700	-4.40683700	-3.91234700
C	0.04080600	-3.72727700	-5.46737300
H	0.29131400	-4.44579900	-6.24900900

H	0.58594000	-2.81606800	-5.70743700
C	-1.47216300	-3.46281300	-5.48371100
H	-2.01513400	-4.18625600	-4.86768800
C	-2.05345400	-3.59683100	-6.92451600
H	-3.10230300	-3.30014100	-6.84168500
C	-1.35944700	-2.71153000	-7.95811700
H	-0.32183400	-3.02450900	-8.11450200
H	-1.36513500	-1.66387200	-7.63928600
H	-1.87816900	-2.77897300	-8.91938300
C	-0.46372200	-4.60846500	-2.64582100
C	-0.86691600	-5.87006200	-2.18733100
C	-1.08793800	-3.45736300	-2.13188100
C	-1.88250800	-5.97724300	-1.23437500
H	-0.39687200	-6.76829400	-2.57215300
C	-2.10927500	-3.56992600	-1.19008100
H	-0.78315100	-2.47588700	-2.47788700
C	-2.50577500	-4.83196500	-0.73915600
H	-2.18929500	-6.95998800	-0.88826600
H	-2.60167900	-2.67409600	-0.82192000
H	-3.30206800	-4.91965700	-0.00505400
C	1.36839500	-6.10622300	-4.38373800
C	2.57244500	-6.61225300	-3.87727500
C	0.57464200	-6.91579400	-5.21232200
C	2.98063000	-7.90827200	-4.19601500
H	3.18701600	-5.97928100	-3.24410500
C	0.98608300	-8.21053300	-5.53036500
H	-0.36125400	-6.54457900	-5.61755200
C	2.18905100	-8.70750900	-5.02268400
H	3.91890600	-8.29000300	-3.80251300
H	0.37007000	-8.82133700	-6.18331500
H	2.50950300	-9.71498600	-5.27452100
O	-1.95318800	-4.95877800	-7.34238500
Si	-3.09427600	-6.16720100	-7.60064900
C	-3.80023100	-6.74707700	-5.94023800
C	-4.36724900	-5.81609800	-5.04348600
C	-3.77901200	-8.09754100	-5.54094500
C	-4.89689800	-6.21973500	-3.81629800
H	-4.38248300	-4.75437700	-5.27358100
C	-4.31711000	-8.50641200	-4.31841000
H	-3.32725500	-8.84648700	-6.18461100
C	-4.88131000	-7.56776600	-3.45348900
H	-5.31434500	-5.47379100	-3.14554200
H	-4.28987800	-9.55759200	-4.04114800
H	-5.30073000	-7.88392400	-2.50141400
C	-2.04612300	-7.51927400	-8.41747800
C	-0.72189800	-7.23641800	-8.80518700
C	-2.53720300	-8.81036300	-8.70121000
C	0.07489600	-8.19396500	-9.43747200
H	-0.31268700	-6.25094300	-8.60660700
C	-1.74514000	-9.77274700	-9.33136000
H	-3.55448000	-9.07949900	-8.42983900
C	-0.43405700	-9.46660500	-9.70116800
H	1.09367400	-7.94441800	-9.72362500
H	-2.15336600	-10.75959200	-9.53480400
H	0.18396200	-10.21318700	-10.19354900
C	-4.50249600	-5.61583200	-8.80175200
C	-5.34052200	-4.44934100	-8.23387600
H	-6.16288000	-4.21359500	-8.92515400
H	-4.75355800	-3.53254400	-8.11274800
H	-5.78904900	-4.69300400	-7.26479700
C	-5.45392600	-6.81134100	-9.04060700
H	-6.27882900	-6.50333300	-9.69906800
H	-5.89870600	-7.17596500	-8.10688600
H	-4.94663400	-7.65005500	-9.52956800
C	-3.89478200	-5.18689500	-10.15514500
H	-3.31432700	-5.99346800	-10.61704100
H	-3.23345200	-4.31882000	-10.05068700
H	-4.69371200	-4.90801900	-10.85763800
N	-1.74224100	-2.12273000	-4.96166800
H	-0.94354800	-1.51420800	-4.79653600
C	-2.98996400	-1.73095300	-4.58591700
O	-3.97920700	-2.45721300	-4.68413200
C	-3.14256200	-0.27482900	-4.10445500
H	-4.09816500	-0.25131000	-3.57768500
C	-3.18901300	0.71730500	-5.30556000
C	-4.20756100	0.27861400	-6.36947000
H	-4.24352100	1.01452000	-7.18076100
H	-5.21269200	0.20026500	-5.93879400
H	-3.96267000	-0.69298300	-6.80777500
C	-3.50323900	2.14172700	-4.82407600
H	-3.49429400	2.84015800	-5.66869600
H	-2.77562000	2.49566200	-4.08771300
H	-4.49771600	2.18607300	-4.36308500
N	-2.13309300	0.13871700	-3.13140100
H	-1.16103600	0.19319600	-3.42897700
C	-2.47028600	0.44625700	-1.84511100
O	-3.57528100	0.20570800	-1.35955400
C	2.17835800	-3.53985700	-3.17723600
C	2.21316400	-3.17188300	-1.72022100
H	1.41886500	-3.60102200	-1.10718700
H	3.16662300	-3.48526200	-1.27249900
C	3.16466300	-2.73891200	-3.81048600
H	4.11182700	-2.65723400	-3.27362100
C	3.43178700	-2.74555600	-5.28027200
O	4.53283600	-2.03211500	-5.56417700
O	2.76505400	-3.35877900	-6.10082900
C	5.06054900	-1.91557400	-6.94095500
H	-2.18814900	0.72184800	-5.76498800
C	5.45923800	-3.30113800	-7.46158300
H	6.15356700	-3.78487100	-6.76558100

H	5.96741600	-3.19367800	-8.42652000
H	4.58730900	-3.94331700	-7.59486900
C	6.29567800	-1.03217900	-6.74882300
H	6.79330000	-0.87142300	-7.71124000
H	7.00764800	-1.50777900	-6.06615300
H	6.01417300	-0.06007800	-6.33323000
C	4.03207200	-1.22568100	-7.84318600
H	3.72777400	-0.26670000	-7.41147400
H	3.14786600	-1.84951700	-7.98418800
H	4.48263200	-1.03178100	-8.82337100
C	-1.42516100	1.15866300	-1.01634800
C	-1.67531800	1.26070700	0.35727800
C	-0.29683800	1.79041500	-1.55221800
C	-0.80327700	1.96893900	1.18242700
H	-2.57350400	0.79850100	0.75102900
C	0.57052600	2.50272500	-0.72144600
H	-0.09049800	1.75983900	-2.61534300
C	0.32392800	2.59526400	0.64742900
H	0.99453200	3.15766700	1.28607900
C	-1.05097300	2.01394700	2.66757900
C	1.81384600	3.12914200	-1.29960900
F	-2.36654300	2.02222200	2.95989500
F	-0.51992100	0.92862800	3.28840300
F	-0.49393600	3.10252000	3.23863700
F	2.18500300	4.22996900	-0.60965800
F	1.63722100	3.49392000	-2.58811700
F	2.85676800	2.27096600	-1.26797300
C	3.83714600	0.73044500	-3.82183000
C	2.59641100	0.74491700	-4.47612400
C	2.33124800	1.70565500	-5.46119500
C	3.32986400	2.62208300	-5.77508200
C	4.57213400	2.58451000	-5.10891900
C	4.85013300	1.63710100	-4.12292700
C	2.70202200	-0.97229200	-2.92484500
C	1.79882500	-0.30241500	-3.85101300
H	1.36605700	1.72567000	-5.95976900
H	3.15478400	3.37948400	-6.53359500
H	5.33165600	3.31878000	-5.36369500
H	5.79964700	1.61222000	-3.59872800
O	3.90851200	-0.24127000	-2.87285000
O	0.58633700	-0.59625100	-4.03990400
C	2.16541900	-1.57638400	-1.63295400
H	1.10266800	-1.31335800	-1.62163200
C	2.77988300	-1.08721700	-0.33420600
C	4.15993700	-1.15688600	-0.08422700
C	1.94443000	-0.64093700	0.69787400
C	4.68216700	-0.77552400	1.15157200
H	4.83355000	-1.49886700	-0.86360100
C	2.46167500	-0.26230000	1.93866300
H	0.87120400	-0.59326500	0.53064700
C	3.83601900	-0.32630800	2.16837900
H	5.75472700	-0.83001900	1.32057800
H	1.78637800	0.08308300	2.71576800
H	4.24541800	-0.03140400	3.13105100

34-int-*re*

P	2.49835400	3.82627200	0.00419300
C	3.00342000	2.93040100	-1.55737700
H	4.07149700	3.08338500	-1.72097100
H	2.47814800	3.44879000	-2.36722600
C	2.73764300	1.42065900	-1.62444500
H	3.13746800	0.93880700	-0.72527700
C	3.47545000	0.76059900	-2.82892000
H	3.24846000	-0.30682300	-2.76605100
C	3.03362700	1.27426300	-4.19629300
H	3.25682000	2.34021700	-4.31732200
H	1.96156200	1.10753400	-4.33972000
H	3.56750100	0.73261300	-4.98340600
C	2.22830200	2.64880600	1.38772800
C	3.21821900	2.39330100	2.34799100
C	0.99733500	1.97567800	1.48524900
C	2.98721800	1.47666300	3.37579600
H	4.17058200	2.90827200	2.30006400
C	0.76719600	1.06950200	2.51925000
H	0.22085600	2.17295900	0.75450200
C	1.76342000	0.81430100	3.46452700
H	3.76798700	1.28394900	4.10593200
H	-0.19047700	0.56027300	2.58516100
H	1.58444000	0.10284600	4.26623700
C	4.07913000	4.72163500	0.42643800
C	3.98801800	6.07414200	0.78419900
C	5.34921700	4.12289400	0.33861100
C	5.14342700	6.81001700	1.06206900
H	3.01537600	6.55538800	0.83133600
C	6.50070700	4.86029900	0.61525300
H	5.45164500	3.07717600	0.05984900
C	6.39834800	6.20629300	0.97842800
H	5.05951200	7.85871000	1.33493100
H	7.47476600	4.38582600	0.53435000
H	7.29615300	6.78223500	1.18738000
O	4.87751800	0.98805600	-2.67398100
Si	6.15476400	0.01159200	-2.19279900
C	5.88746300	-0.60407400	-0.41732200
C	4.72808700	-1.32768500	-0.06372400
C	6.82180900	-0.34774300	0.60561400
C	4.52364100	-1.78279100	1.24028100
H	3.94673000	-1.52104800	-0.79257900
C	6.62546200	-0.80925100	1.90945000
H	7.71677500	0.22804000	0.38935800
C	5.47641200	-1.53314300	2.22965900

H	3.61419000	-2.32782900	1.47857700
H	7.36964200	-0.59900900	2.67409600
H	5.32134900	-1.89450400	3.24340100
C	7.61507000	1.21667600	-2.26386400
C	7.42627400	2.51727200	-2.76972500
C	8.91690100	0.87088900	-1.84741400
C	8.48017800	3.43074600	-2.84832000
H	6.43889400	2.81275600	-3.11063800
C	9.97485100	1.77944500	-1.92312900
H	9.11797800	-0.12119700	-1.45141400
C	9.75843400	3.06475100	-2.42343200
H	8.30202400	4.42784000	-3.24346000
H	10.96704100	1.48252100	-1.59233300
H	10.58043700	3.77354200	-2.48465500
C	6.42587500	-1.44603600	-3.42483300
C	5.20920700	-2.39659100	-3.47208000
H	5.41062300	-3.22328000	-4.16910000
H	4.30029200	-1.89719600	-3.82522900
H	4.99339600	-2.84254200	-2.49495400
C	7.66253600	-2.26962600	-2.99890100
H	7.79574300	-3.12124400	-3.68160400
H	7.55848400	-2.67562900	-1.98532500
H	8.58368000	-1.67785900	-3.03824000
C	6.66916700	-0.87325300	-4.83874500
H	7.54108300	-0.20981900	-4.86622600
H	5.80620100	-0.30328900	-5.20198300
H	6.84947300	-1.69048500	-5.55212300
N	1.30338100	1.14617500	-1.67295000
H	0.66266800	1.91674100	-1.81420400
C	0.79468800	-0.09365000	-1.43067600
O	1.50894200	-1.06932700	-1.21406500
C	-0.75428500	-0.16892600	-1.35821000
H	-1.02329500	0.20900100	-0.36248300
C	-1.28055000	-1.62906100	-1.45821400
C	-1.01178700	-2.38081700	-0.14263100
H	-1.33559700	-3.42444000	-0.23072700
H	-1.57735700	-1.93110000	0.68631600
H	0.04985500	-2.37077500	0.10918200
C	-2.77946500	-1.67793000	-1.78868500
H	-3.12655200	-2.71741400	-1.77251800
H	-3.00189400	-1.27156300	-2.77913300
H	-3.37270000	-1.12063000	-1.05121300
N	-1.38288900	0.77687800	-2.28252500
H	-1.83465900	1.58288700	-1.86586200
C	-1.24170100	0.67543100	-3.63156700
O	-0.54077900	-0.18664200	-4.16237900
C	1.08759500	4.73018900	-0.28394000
C	0.91530300	5.65476800	-1.47468800
H	1.31944200	6.66196800	-1.30597100
H	1.37286400	5.28917000	-2.40319800
C	0.01983100	5.08846800	0.71767600
H	-0.23323100	4.27754900	1.40523700
C	0.29952500	6.31945800	1.60247300
O	-0.14676100	6.09602500	2.85103700
O	0.86440800	7.33416800	1.23297400
C	-0.02587500	7.10254700	3.92467300
H	-0.72713200	-2.12429700	-2.26180700
C	1.45247700	7.39313800	4.20480500
H	1.99533600	6.45995000	4.39083300
H	1.53928900	8.02108800	5.09900800
H	1.91957000	7.91157000	3.36620000
C	-0.67526100	6.39412000	5.11668600
H	-0.65344400	7.04279700	5.99893800
H	-0.14139000	5.46795100	5.35322400
H	-1.71780900	6.14347000	4.89533500
C	-0.80772700	8.36510900	3.54801200
H	-1.84620800	8.11136000	3.30956200
H	-0.36102900	8.86423200	2.68679300
H	-0.81115300	9.05912400	4.39634300
C	-1.99611900	1.67669300	-4.47548100
C	-1.68947800	1.70311600	-5.84046900
C	-2.97914600	2.54111700	-3.97901700
C	-2.34079900	2.59248400	-6.69351400
H	-0.93380300	1.01938600	-6.21016900
C	-3.62989800	3.42857000	-4.83872300
H	-3.24978500	2.54213500	-2.92966700
C	-3.31314300	3.46185600	-6.19730300
H	-3.80872100	4.16404800	-6.85792000
C	-2.03118900	2.58150600	-8.16935600
C	-4.71905700	4.32582100	-4.30985300
F	-0.74965100	2.23718100	-8.40882200
F	-2.81138600	1.69664200	-8.82911700
F	-2.24383900	3.79175600	-8.73122800
F	-4.72635700	5.52112700	-4.94385700
F	-4.57448000	4.56604200	-2.98808500
F	-5.94344200	3.78144300	-4.48290600
C	-3.23463900	6.11473600	0.60731600
C	-3.46258600	4.77266200	0.27947800
C	-4.71915700	4.18564400	0.47853700
C	-5.73116900	4.96984900	1.01718800
C	-5.48479300	6.31990400	1.34295400
C	-4.24142900	6.91780000	1.14604500
C	-1.21384200	5.42490400	-0.21194700
C	-2.21618600	4.26059000	-0.26437200
H	-4.88272300	3.14499000	0.21439400
H	-6.71822200	4.55061900	1.18574400
H	-6.29306100	6.91680600	1.75722000
H	-4.05749000	7.95935200	1.38606400
O	-1.97425300	6.53083900	0.34839000
O	-1.97226200	3.13838500	-0.70845000

C	-0.63077300	5.74861600	-1.64035700
H	-0.93155400	4.90619500	-2.27183000
C	-1.14872500	7.02009300	-2.28149300
C	-0.80298500	8.29050500	-1.79276300
C	-1.98599300	6.94165000	-3.40058700
C	-1.27995800	9.44256400	-2.41725500
H	-0.17241500	8.37321200	-0.91219100
C	-2.47034400	8.09316400	-4.02429600
H	-2.26279400	5.96653700	-3.79039900
C	-2.11457300	9.34971000	-3.53500500
H	-1.00178400	10.41844700	-2.02695200
H	-3.12603900	8.00092600	-4.88592700
H	-2.48522800	10.25057500	-4.01729500

34-int-*si*

P	0.85686900	-4.55924100	-3.93839500
C	0.08164700	-4.08331600	-5.57345400
H	0.15878400	-4.95788000	-6.22250000
H	0.73568200	-3.32621000	-6.00547300
C	-1.37549400	-3.61141900	-5.60247600
H	-1.99952500	-4.24694100	-4.96627700
C	-1.96199100	-3.73792100	-7.04381500
H	-2.97910200	-3.34381800	-6.98743700
C	-1.18022700	-2.96628300	-8.10539700
H	-0.19167300	-3.40924400	-8.26497200
H	-1.05291700	-1.91891000	-7.81115800
H	-1.71607600	-2.99173200	-9.05973900
C	-0.49099200	-4.83721000	-2.71920200
C	-0.86911600	-6.11691700	-2.28971200
C	-1.18331500	-3.72078400	-2.21708000
C	-1.92127400	-6.27441400	-1.38437000
H	-0.34582900	-6.99269600	-2.65642600
C	-2.23963800	-3.87999300	-1.32184800
H	-0.89513100	-2.72467900	-2.53347800
C	-2.60970200	-5.16070700	-0.90359300
H	-2.20410900	-7.27246800	-1.06173500
H	-2.77534600	-3.00347300	-0.96754100
H	-3.43377300	-5.28844500	-0.20679900
C	1.52492800	-6.25936600	-4.30224500
C	2.78740100	-6.57408000	-3.78467600
C	0.84026900	-7.22610100	-5.05682000
C	3.35739900	-7.82869800	-4.00861200
H	3.32315900	-5.82188900	-3.21153700
C	1.41004100	-8.47999300	-5.28452500
H	-0.14274900	-7.01481500	-5.46880700
C	2.66937400	-8.78271600	-4.76017600
H	4.33921300	-8.05807800	-3.60252700
H	0.87156800	-9.21258900	-5.87932300
H	3.11333400	-9.75828800	-4.94084400
O	-1.98152100	-5.11822000	-7.40963700
Si	-3.21081700	-6.25240700	-7.56961000
C	-4.04247800	-6.58946300	-5.90033100
C	-4.62816300	-5.54298700	-5.15596100
C	-4.10024400	-7.88413000	-5.34795600
C	-5.25299800	-5.78427700	-3.93091000
H	-4.57638200	-4.51458300	-5.49998900
C	-4.73288700	-8.13071800	-4.12739500
H	-3.63629300	-8.71654900	-5.86843500
C	-5.31611400	-7.08066700	-3.41725200
H	-5.68220600	-4.95263700	-3.37874100
H	-4.76445600	-9.14279300	-3.73068700
H	-5.80868500	-7.26976300	-2.46652200
C	-2.25884500	-7.77811200	-8.16782400
C	-0.88848600	-7.67568700	-8.47478700
C	-2.86729000	-9.03588000	-8.35672400
C	-0.15898900	-8.77325000	-8.93913000
H	-0.38934200	-6.72007200	-8.34910700
C	-2.14423300	-10.13706200	-8.82020000
H	-3.92297300	-9.17042200	-8.13623100
C	-0.78505100	-10.00896100	-9.11207200
H	0.89813100	-8.66072500	-9.16712000
H	-2.64314000	-11.09382300	-8.95317200
H	-0.22012400	-10.86415700	-9.47445900
C	-4.49559900	-5.71755100	-8.90703400
C	-5.19656600	-4.38921500	-8.54559700
H	-5.94453200	-4.14394600	-9.31396000
H	-4.49725400	-3.54719900	-8.50058900
H	-5.72250000	-4.44390400	-7.58628500
C	-5.58382600	-6.80653300	-9.04802500
H	-6.33281300	-6.49019200	-9.78849800
H	-6.11077700	-6.98602000	-8.10336500
H	-5.16994500	-7.75923700	-9.39641300
C	-3.77951000	-5.54974800	-10.26542800
H	-3.30568400	-6.48124400	-10.59485800
H	-3.00180700	-4.77788800	-10.22516200
H	-4.50160500	-5.25159300	-11.03966900
N	-1.50117000	-2.22893000	-5.11884100
H	-0.65694200	-1.71715300	-4.87478900
C	-2.72289300	-1.69819800	-4.82723800
O	-3.77446600	-2.31445800	-4.99545100
C	-2.78206700	-0.22672000	-4.36952300
H	-3.78294700	-0.12505200	-3.94492300
C	-2.62469300	0.75200900	-5.57043200
C	-3.53003100	0.36477400	-6.75019200
H	-3.43232200	1.10299900	-7.55421100
H	-4.58190700	0.33422300	-6.44343800
H	-3.27940700	-0.61589000	-7.16398400
C	-2.91515400	2.19685100	-5.13472000
H	-2.75126300	2.88782600	-5.96944600
H	-2.27738300	2.51517900	-4.30433600

H	-3.95904300	2.30214200	-4.81367300
N	-1.84821500	0.13004900	-3.30459300
H	-0.88167100	0.31210600	-3.55775800
C	-2.26378800	0.27893400	-2.01300300
O	-3.35394300	-0.11818100	-1.60510600
C	1.92573300	-3.42444600	-3.27480800
C	2.08966800	-3.15196000	-1.80064500
H	1.25433600	-3.46552900	-1.17040600
H	2.99971500	-3.61721700	-1.37874300
C	3.03794700	-2.60989000	-3.88288300
H	4.02323700	-2.97729800	-3.52010700
C	3.23840900	-2.60949400	-5.39021800
O	3.80277000	-1.47259200	-5.82692600
O	2.99263900	-3.57691600	-6.08810400
C	4.23013800	-1.29774000	-7.23592900
H	-1.57705400	0.69396500	-5.90437000
C	5.30027800	-2.33748700	-7.58404900
H	6.12872900	-2.28510000	-6.86915400
H	5.70085400	-2.12590300	-8.58192800
H	4.89047900	-3.34857300	-7.57930800
C	4.82443500	0.11245900	-7.23572000
H	5.19330000	0.35979600	-8.23687200
H	5.65921900	0.18138800	-6.53131800
H	4.07342600	0.85432800	-6.94810300
C	3.01303100	-1.37577800	-8.16295200
H	2.23712500	-0.67680600	-7.83176500
H	2.59594000	-2.38364200	-8.18759700
H	3.31104400	-1.09482400	-9.17939700
C	-1.34156800	1.03018900	-1.07882000
C	-1.61538600	0.93631500	0.29242400
C	-0.33140800	1.89421700	-1.51397200
C	-0.88247900	1.68598000	1.20892700
H	-2.42597100	0.29237800	0.61304200
C	0.39366700	2.65325000	-0.59048800
H	-0.12176800	2.01563100	-2.57052700
C	0.12181700	2.55438800	0.77167800

H	0.67392600	3.15686500	1.48364400
C	-1.12819400	1.53951800	2.68841300
C	1.50324300	3.55327500	-1.07112400
F	-2.35724800	1.05983500	2.95539100
F	-0.23619700	0.68458000	3.24980100
F	-0.99410200	2.71850000	3.33350000
F	1.73096900	4.57122500	-0.21449100
F	1.21599400	4.09397400	-2.27835300
F	2.66399000	2.87817600	-1.20612600
C	4.18890000	0.64345200	-3.45067900
C	2.94406400	1.00454800	-3.98145500
C	2.73250300	2.28246600	-4.51491300
C	3.78676700	3.18697500	-4.49142400
C	5.03124700	2.81028100	-3.94568500
C	5.25846600	1.53970300	-3.41977700
C	2.92011600	-1.22860600	-3.13906100
C	2.08705800	-0.16114500	-3.86180000
H	1.76456500	2.54868200	-4.92971600
H	3.65665900	4.18993800	-4.88571900
H	5.84051500	3.53560500	-3.93143200
H	6.21590100	1.25334000	-2.99834300
O	4.23571800	-0.62859000	-2.99939500
O	0.91148200	-0.28706600	-4.22185400
C	2.27847500	-1.59959400	-1.73674400
H	1.27291000	-1.16701100	-1.74646400
C	2.98028400	-1.12426900	-0.48146900
C	4.32733000	-1.41512000	-0.20628300
C	2.23450100	-0.46952100	0.50774300
C	4.90504900	-1.04309900	1.00742300
H	4.93113900	-1.92870000	-0.94688800
C	2.80468800	-0.10530500	1.72897700
H	1.18347400	-0.26117000	0.32724700
C	4.14741900	-0.38629600	1.98038800
H	5.95063000	-1.27351600	1.19633500
H	2.19192100	0.38489300	2.47977200
H	4.59895600	-0.10411000	2.92789300

L. References

[1] a) X. Han, Y. Wang, F. Zhong, Y. Lu, *J. Am. Chem. Soc.* **2011**, *133*, 1726. b) X. Han, F. Zhong, Y. Wang, Y. Lu, *Angew. Chem. Int. Ed.* **2012**, *51*, 767 c) F. Zhong, X. Han, Y. Wang, Y. Lu, *Chem. Sci.* **2012**, *3*, 1231 d) F. Zhong, X. Han, Y. Wang, Y. Lu, *Angew. Chem. Int. Ed.* **2011**, *50*, 7837 e) F. Zhong, J. Luo, G.–Y. Chen, X. Dou, Y. Lu, *J. Am. Chem. Soc.* **2012**, *134*, 10222 f) F. Zhong, X. Dou, X. Han, W. Yao, Q. Zhu, Y. Meng, Y. Lu, *Angew. Chem. Int. Ed.* **2013**, *52*, 943 g) W. Yao, X. Dou, Y. Lu, *J. Am. Chem. Soc.* **2015**, *137*, 54.

[2] H. M. Sim, K. Y. Loh, W. K. Yeo, C. Y. Lee, M. L. Go *ChemMedChem* **2011**, *6*, 713.

[3] M. J. Frisch, G. W. Trucks, H. B. Schlegel, G. E. Scuseria, M. A. Robb, J. R. Cheeseman, G. Scalmani, V. Barone, B. Mennucci, G. A. Petersson, H. Nakatsuji, M. Caricato, X. Li, H. P. Hratchian, A. F. Izmaylov, J. Bloino, G. Zheng, J. L. Sonnenberg, M. Hada, M. Ehara, K. Toyota, R. Fukuda, J. Hasegawa, M. Ishida, T. Nakajima, Y. Honda, O. Kitao, H. Nakai, T. Vreven, J. J. A. Montgomery, J. E. Peralta, F. Ogliaro, M. Bearpark, J. J. Heyd, E. Brothers, K. N. Kudin, V. N. Staroverov, T. Keith, R. Kobayashi, J. Normand, K. Raghavachari, A. Rendell, J. C. Burant, S. S. Iyengar, J. Tomasi, M. Cossi, N. Rega, J. M. Millam, M. Klene, J. E. Knox, J. B. Cross, V. Bakken, C. Adamo, J. Jaramillo, R. Gomperts, R. E. Stratmann, O. Yazyev, A. J. Austin, R. Cammi, C. Pomelli, J. W. Ochterski, R. L. Martin, K. Morokuma, V. G. Zakrzewski, G. A. Voth, P. Salvador, J. J. Dannenberg, S. Dapprich, A. D. Daniels, O. Farkas, J. B. Foresman, J. V. Ortiz, J. Cioslowski, D. J. Fox, Gaussian 09, revision D.01, Gaussian, Inc.: Wallingford, CT, **2013**.

[4] (a) A. D. Becke, *J. Chem. Phys*. **1993**, *98*, 5648. (b) C. Lee, W. Yang, R. G. Parr, *Phys. Rev. B: Condens. Matter Mater. Phys.* **1988**, *37*, 785. (c) P. J. Stephens, F. J. Devlin, C. F. Chabalowski, M. J. Frisch, *J. Phys. Chem*. **1994**, *98*, 11623.

[5] (a) W. J. Hehre, R. Ditchfield, J. A. Pople, *J. Chem. Phys*. **1972**, *56*, 2257. (b) J. D. Dill, J. A. Pople, *J. Chem. Phys*. **1975**, *62*, 2921. (c) M. M. Francl, W. J. Pietro, W. J. Hehre, J. S. Binkley, M. S. Gordon, D. J. DeFrees, J. A. Pople, *J. Chem. Phys*. **1982**, *77*, 3654

[6] A. V. Marenich, C. J. Cramer, D. G. Truhlar, *J. Phys. Chem. B* **2009**, *113*, 6378–6396.

[7] R. Peverati, D. G. Truhlar. *J. Phys. Chem. Lett*. **2011**, *2*, 2810–2817.

[8] (a) R. Krishnan, J. S. Binkley, R. Seeger, J. A. Pople, *J. Chem. Phys*. **1980**, *72*, 650. (b) A. D. McLean, G. S. Chandler, *J. Chem. Phys*. **1980**, *72*, 5639.

M. NMR Spectra of the Products

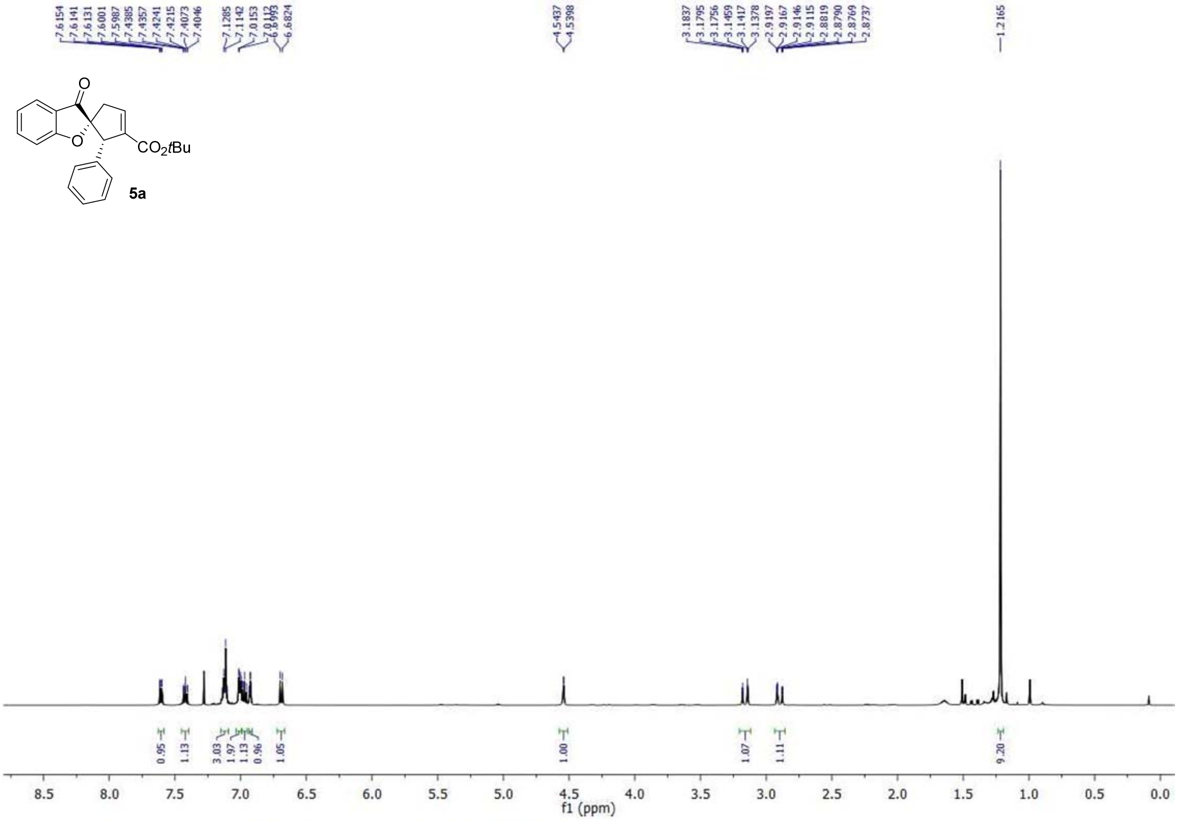

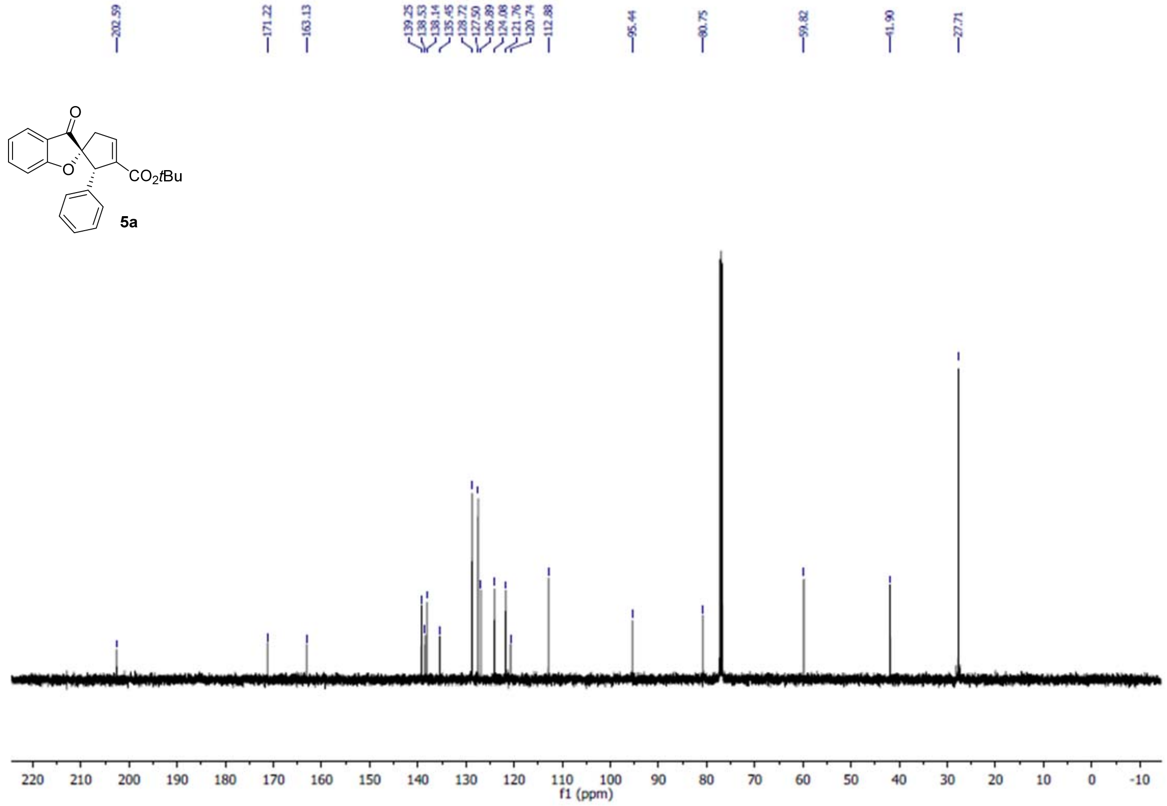

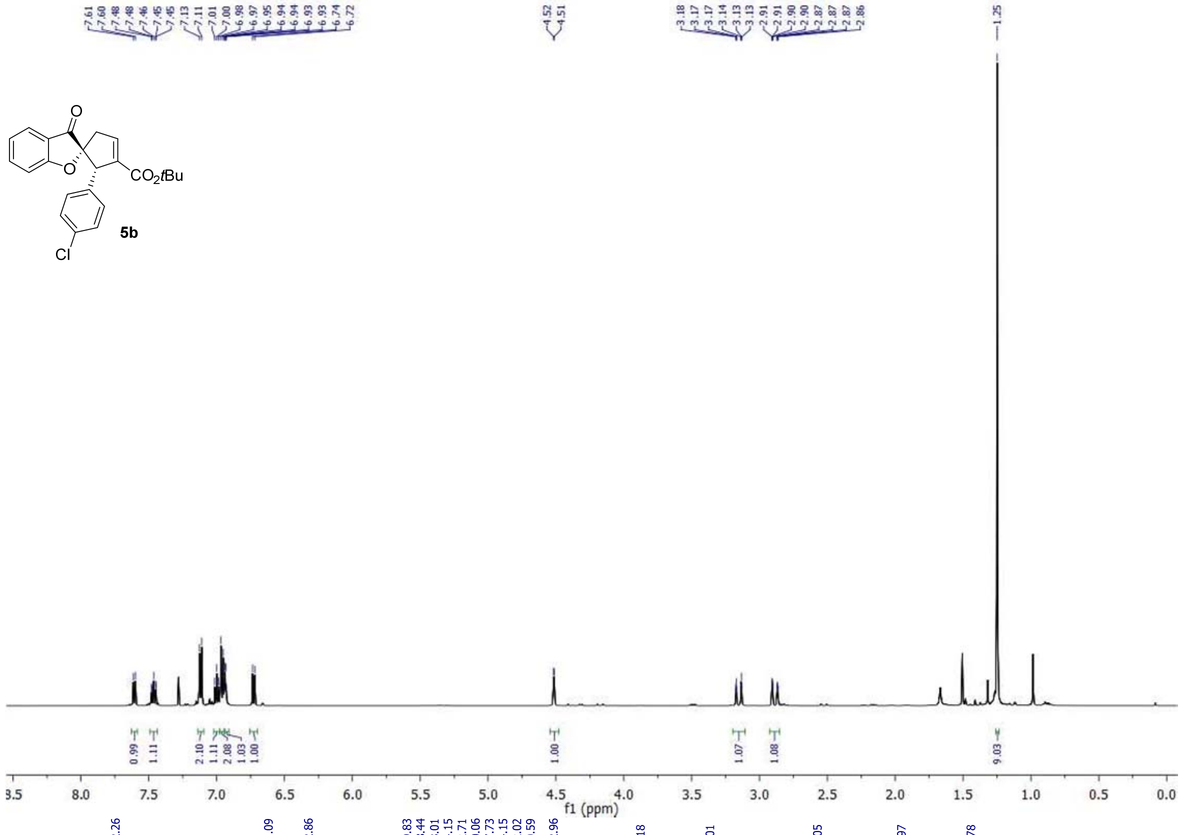
7.61 7.60 7.48 7.46 7.45 7.45 7.13 7.11 7.01 7.00 6.98 6.97 6.95 6.94 6.94 6.93 6.93 6.74 6.72
4.52 4.51
3.18 3.17 3.17 3.14 3.13 3.13 2.91 2.91 2.90 2.87 2.87 2.87 2.86
1.25
CO2tBu
Cl
5b
0.99 1.11 2.10 1.11 2.08 1.03 1.00 1.00 1.07 1.08 9.03
f1 (ppm)

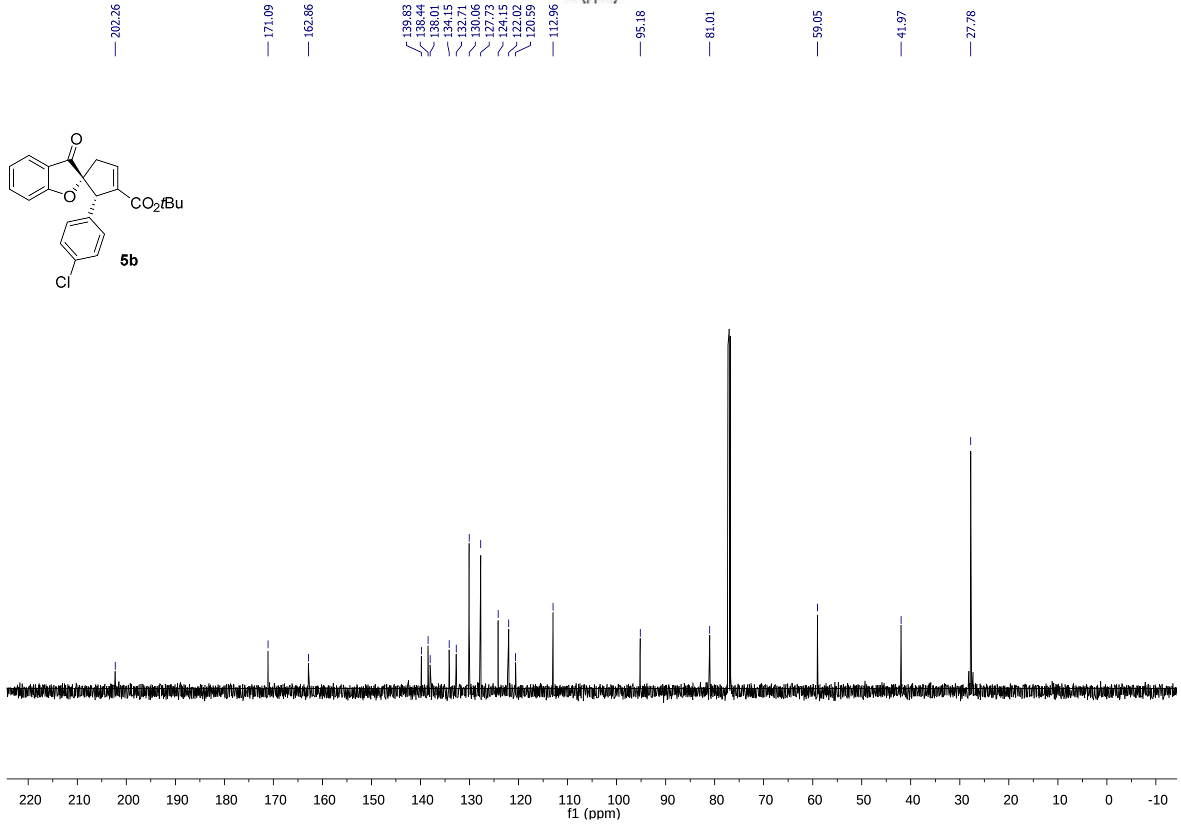
202.26
171.09
162.86
139.83 138.44 138.01 134.15 132.71 130.06 127.73 124.15 122.02 120.59
112.96
95.18
81.01
59.05
41.97
27.78
CO2tBu
Cl
5b
f1 (ppm)

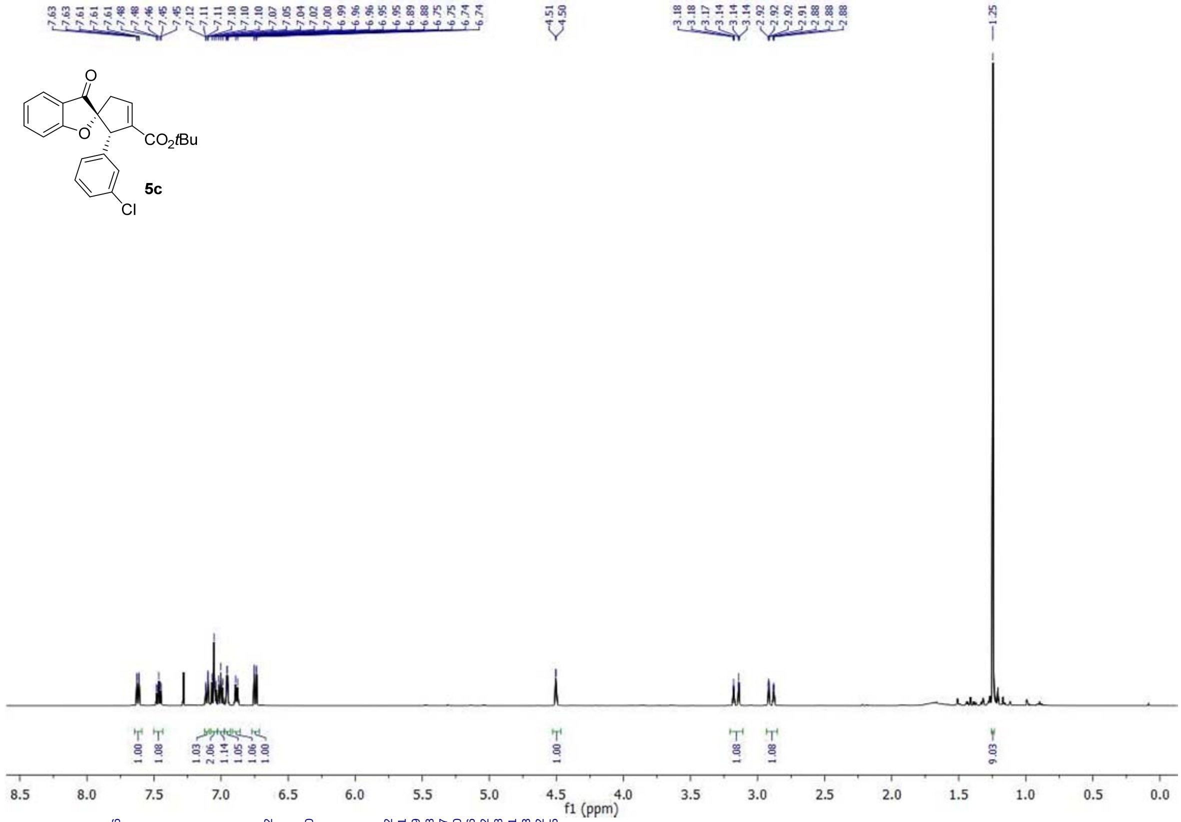
7.63 7.63 7.61 7.61 7.61 7.48 7.46 7.45 7.45 7.12 7.11 7.11 7.10 7.10 7.10 7.07 7.05 7.04 7.02 7.00 6.99 6.96 6.96 6.95 6.95 6.89 6.88 6.75 6.75 6.74 6.74
4.51 4.50
3.18 3.18 3.17 3.14 3.14 3.14 2.92 2.92 2.91 2.88 2.88 2.88
1.25
O
O
CO2tBu
5c
Cl
1.00 1.08 1.03 2.06 1.14 1.05 1.06 1.00 1.00 1.08 1.08 9.03
f1 (ppm)

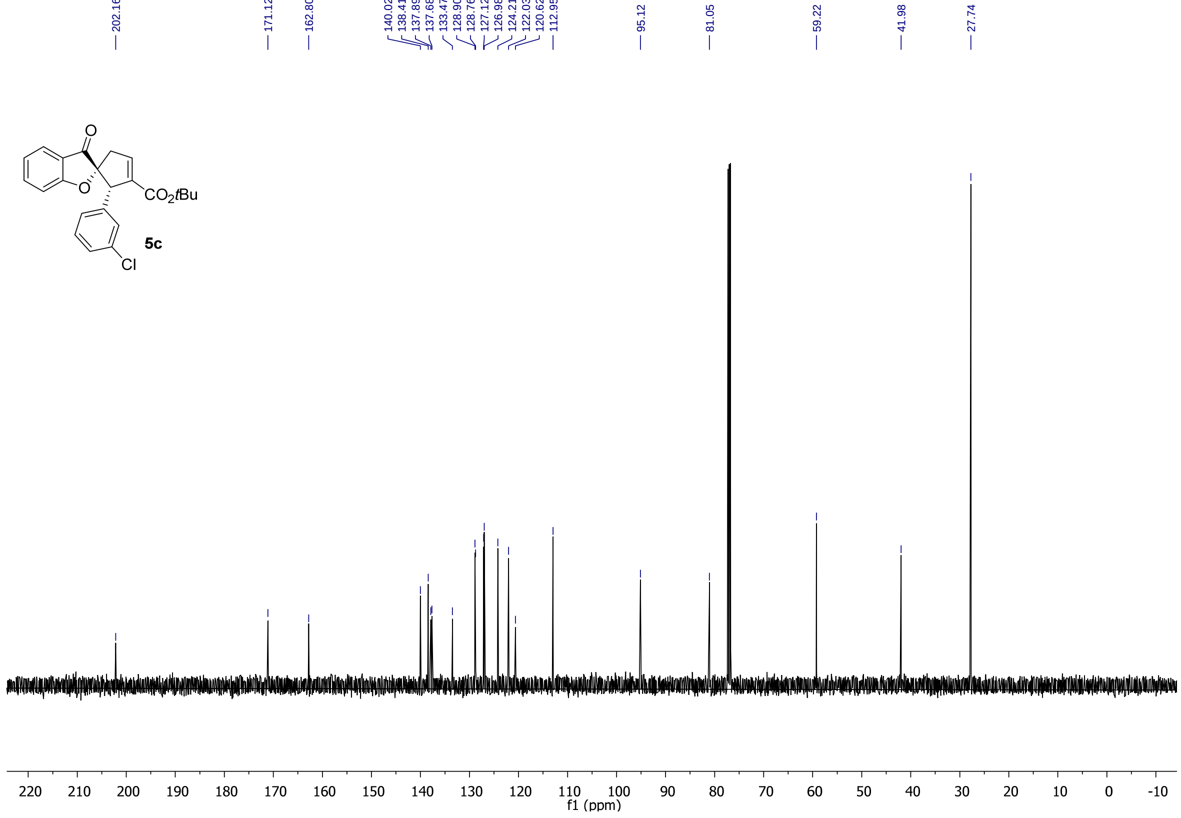
202.16 171.12 162.80 140.02 138.41 137.89 137.68 133.47 128.90 128.76 127.12 126.98 124.21 122.03 120.62 112.95 95.12 81.05 59.22 41.98 27.74
O
O
CO2tBu
5c
Cl
f1 (ppm)

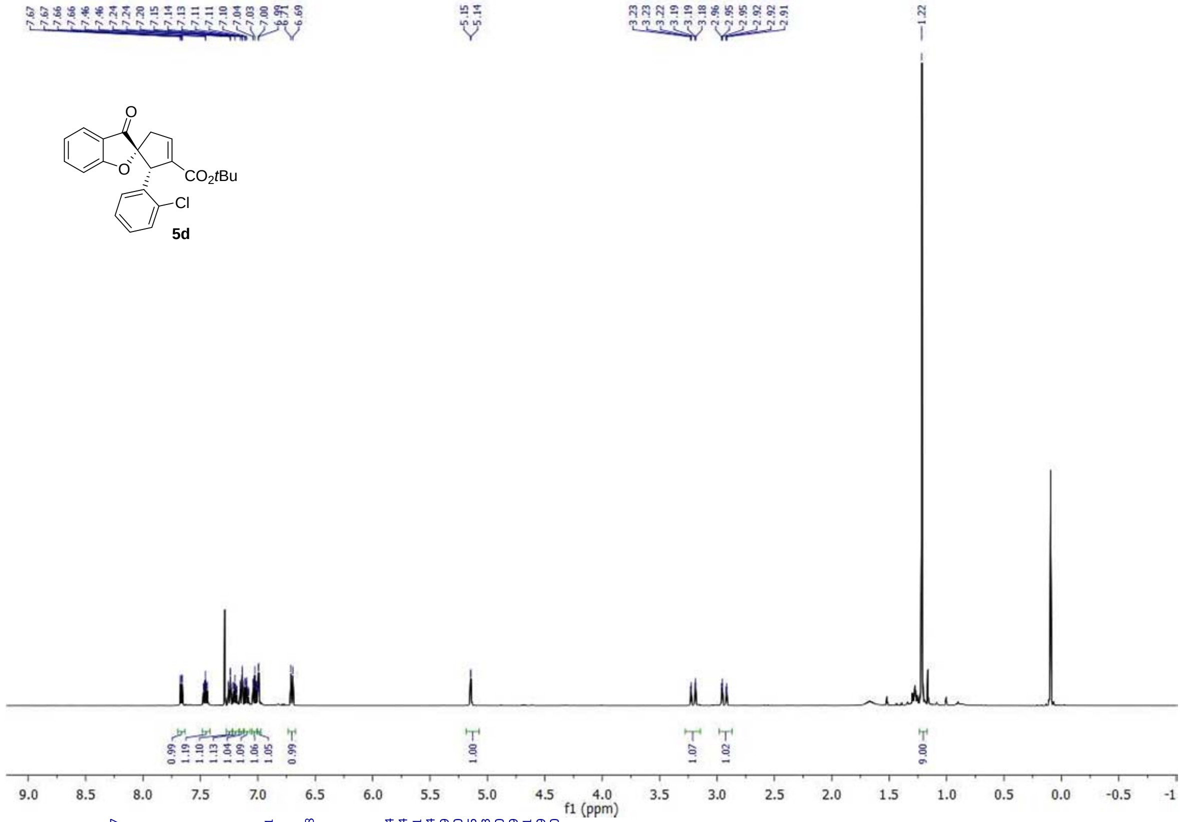
7.67
7.67
7.66
7.46
7.46
7.24
7.24
7.20
7.15
7.14
7.13
7.11
7.11
7.10
7.04
7.03
7.00
6.99
6.71
6.69
5.15
5.14
3.23
3.23
3.22
3.19
3.19
3.18
2.96
2.95
2.95
2.92
2.92
2.91
1.22
O
O
CO2tBu
Cl
5d
0.99
1.19
1.10
1.13
1.04
1.09
1.06
1.05
0.99
1.00
1.07
1.02
9.00
f1 (ppm)

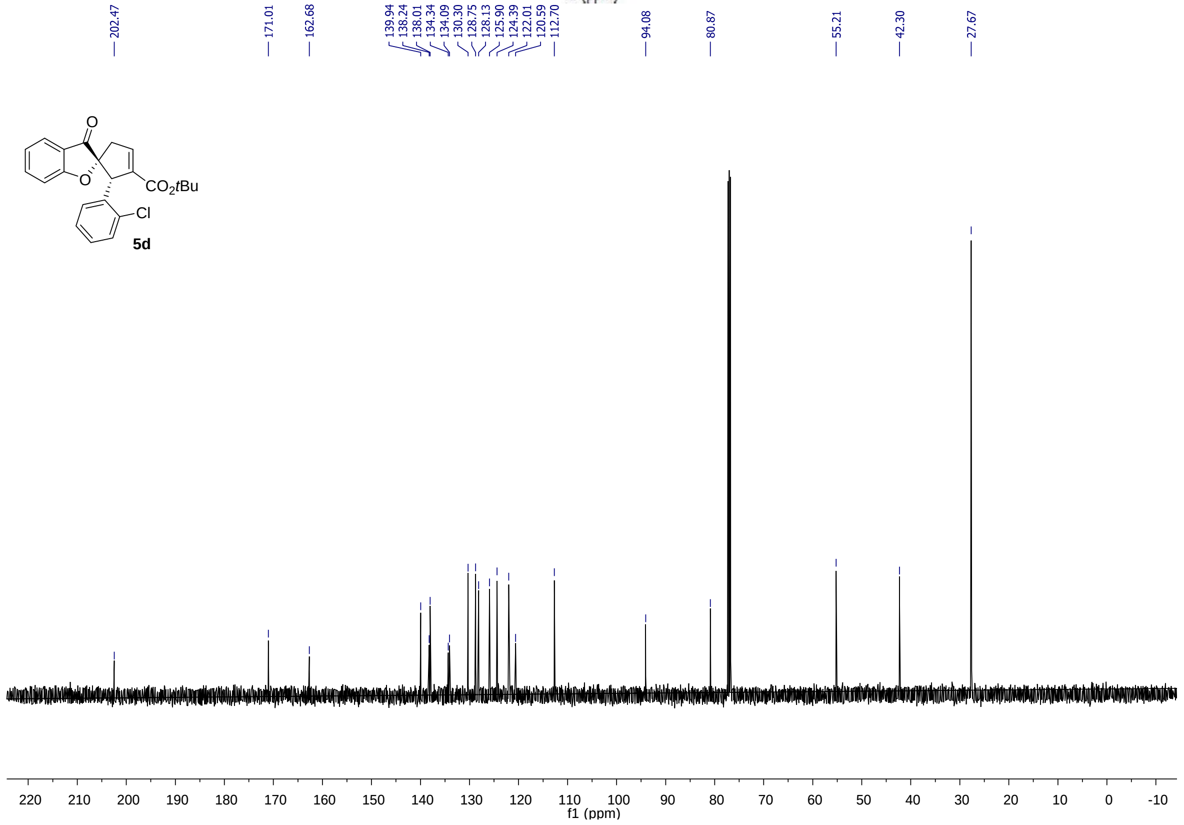
202.47
171.01
162.68
139.94
138.24
138.01
134.34
134.09
130.30
128.75
128.13
125.90
124.39
122.01
120.59
112.70
94.08
80.87
55.21
42.30
27.67
O
O
CO2tBu
Cl
5d
f1 (ppm)

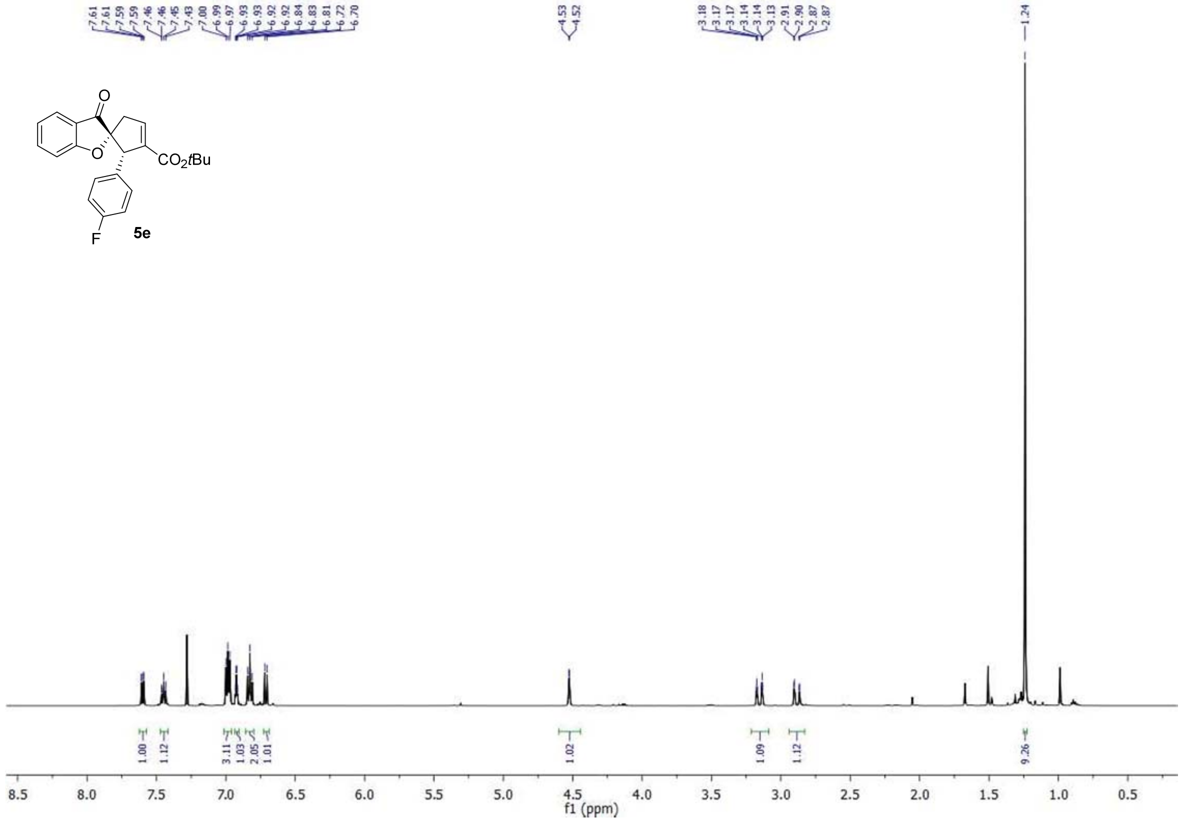
7.61 7.61 7.59 7.59 7.46 7.46 7.45 7.43 7.00 6.99 6.97 6.93 6.93 6.92 6.92 6.84 6.83 6.81 6.72 6.70
4.53 4.52
3.18 3.17 3.17 3.14 3.14 3.13 2.91 2.90 2.87 2.87
1.24
O
O
CO2tBu
F
5e
1.00 1.12 3.11 1.03 2.05 1.01 1.02 1.09 1.12 9.26
8.5 8.0 7.5 7.0 6.5 6.0 5.5 5.0 4.5 4.0 3.5 3.0 2.5 2.0 1.5 1.0 0.5
f1 (ppm)

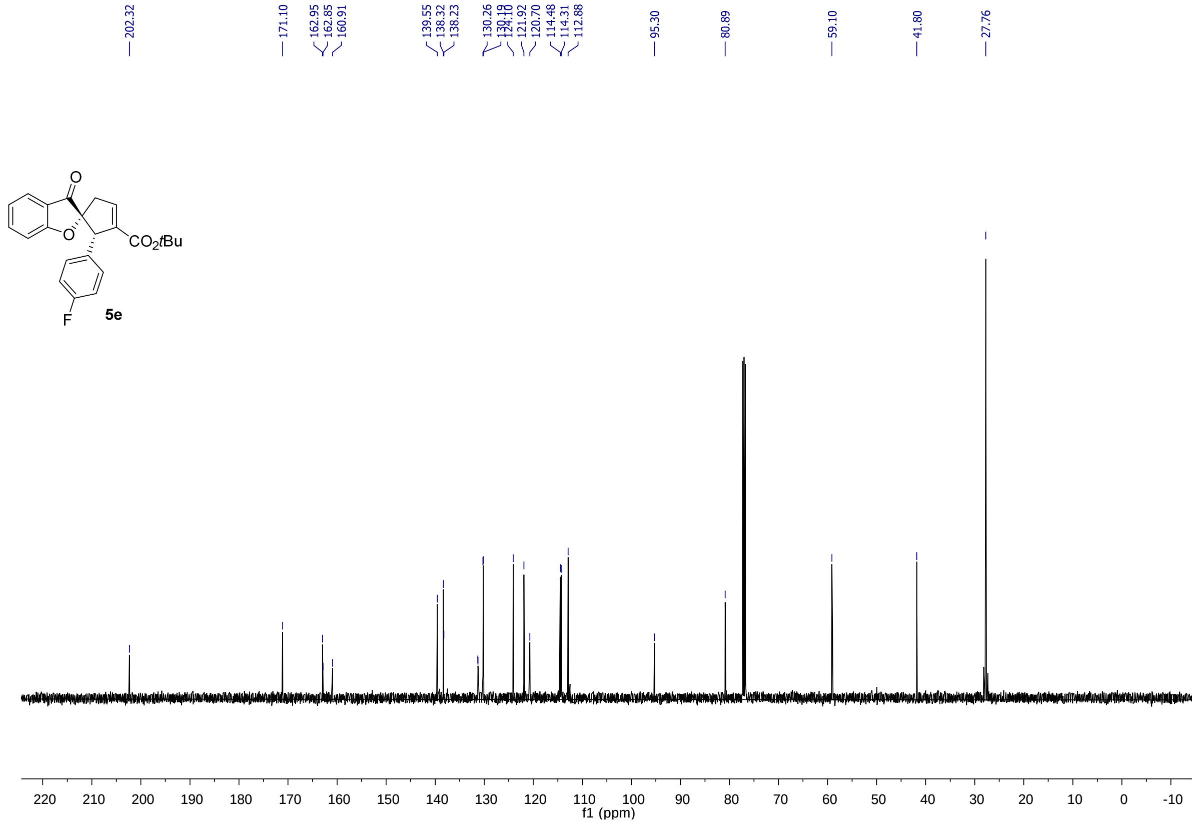
202.32
171.10
162.95 162.85 160.91
139.55 138.32 138.23
130.26 130.19 124.10 121.92 120.70 114.48 114.31 112.88
95.30
80.89
59.10
41.80
27.76
O
O
CO2tBu
F
5e
220 210 200 190 180 170 160 150 140 130 120 110 100 90 80 70 60 50 40 30 20 10 0 -10
f1 (ppm)

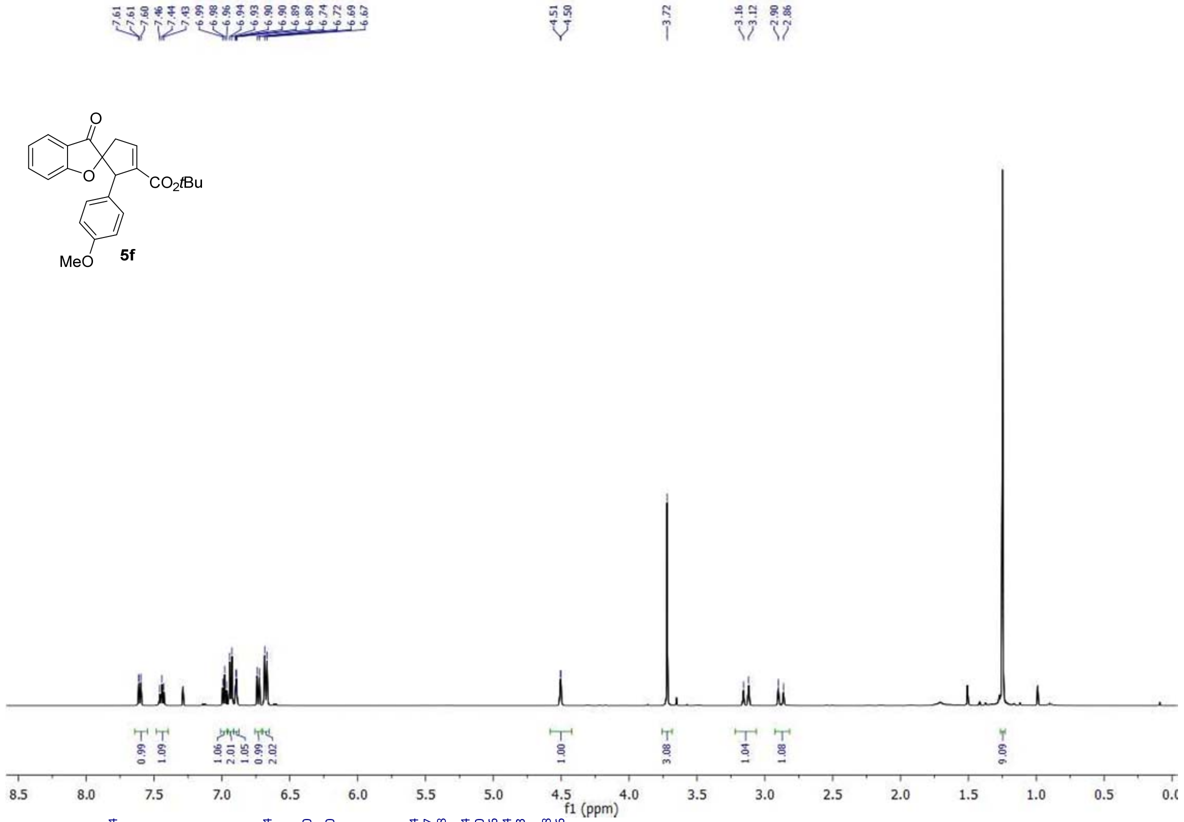
7.61 7.61 7.60 7.46 7.44 7.43 6.99 6.98 6.96 6.94 6.93 6.90 6.90 6.89 6.89 6.74 6.72 6.69 6.67
4.51 4.50
3.72
3.16 3.12 2.90 2.86
O
O
CO2tBu
MeO
5f
0.99 1.09 1.06 2.01 1.05 0.99 2.02 1.00 3.08 1.04 1.08 9.09
8.5 8.0 7.5 7.0 6.5 6.0 5.5 5.0 4.5 4.0 3.5 3.0 2.5 2.0 1.5 1.0 0.5 0.0
f1 (ppm)

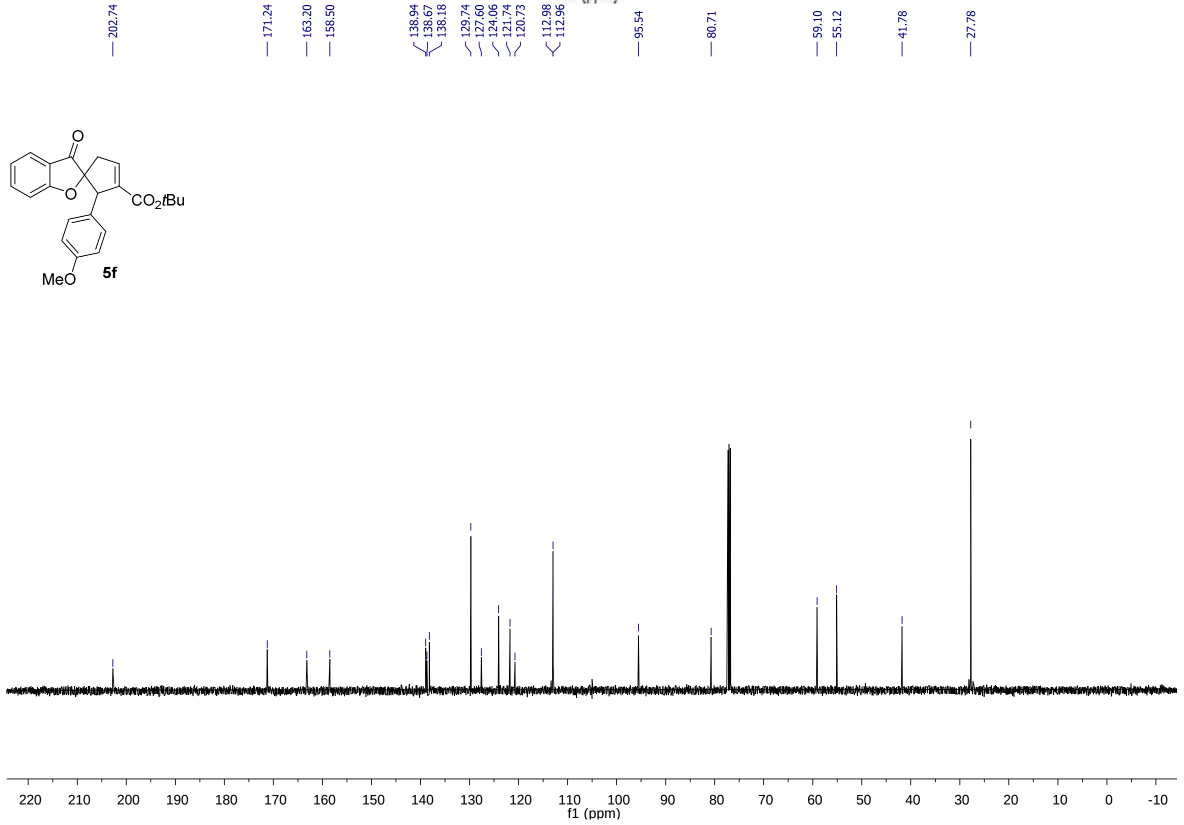
202.74
171.24
163.20
158.50
138.94 138.67 138.18
129.74 127.60 124.06 121.74 120.73
112.98 112.96
95.54
80.71
59.10
55.12
41.78
27.78
O
O
CO2tBu
MeO
5f
220 210 200 190 180 170 160 150 140 130 120 110 100 90 80 70 60 50 40 30 20 10 0 -10
f1 (ppm)

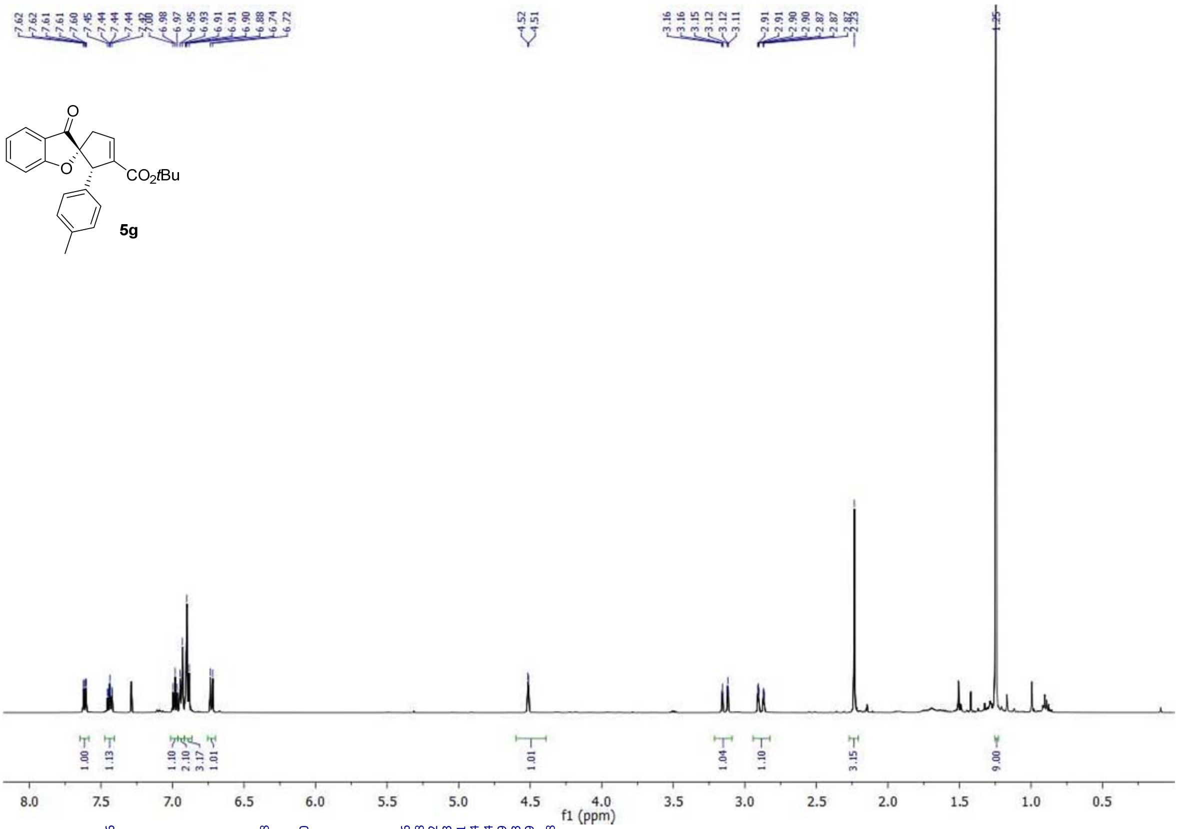
7.62 7.62 7.61 7.61 7.60 7.45 7.44 7.44 7.44 7.42 7.00 6.98 6.97 6.95 6.93 6.91 6.91 6.90 6.88 6.74 6.72
4.52 4.51
3.16 3.16 3.15 3.12 3.12 3.11
2.91 2.91 2.90 2.90 2.87 2.87 2.23 2.23
1.25
CO2tBu
5g
1.00 1.13 1.10 2.10 3.17 1.01 1.01 1.04 1.10 3.15 9.00
f1 (ppm)

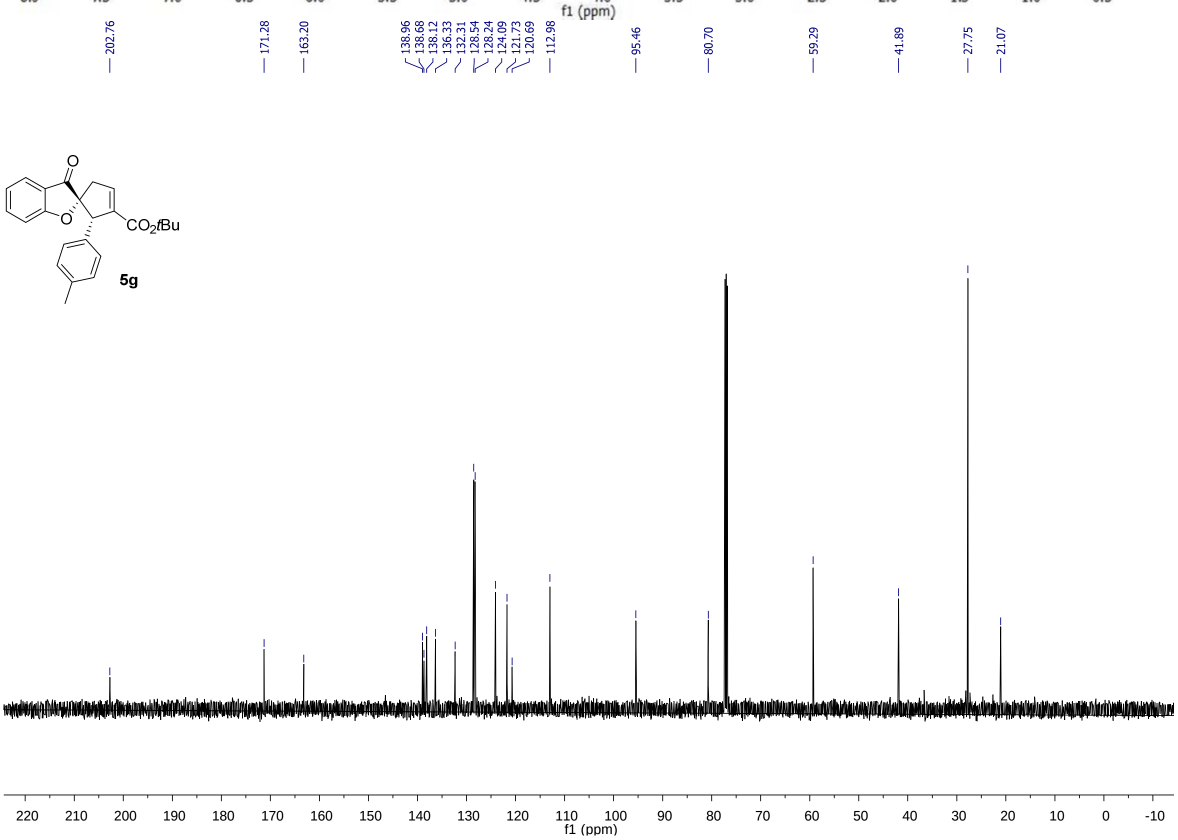
202.76 171.28 163.20 138.96 138.68 138.12 136.33 132.31 128.54 128.24 124.09 121.73 120.69 112.98 95.46 80.70 59.29 41.89 27.75 21.07
CO2tBu
5g
f1 (ppm)

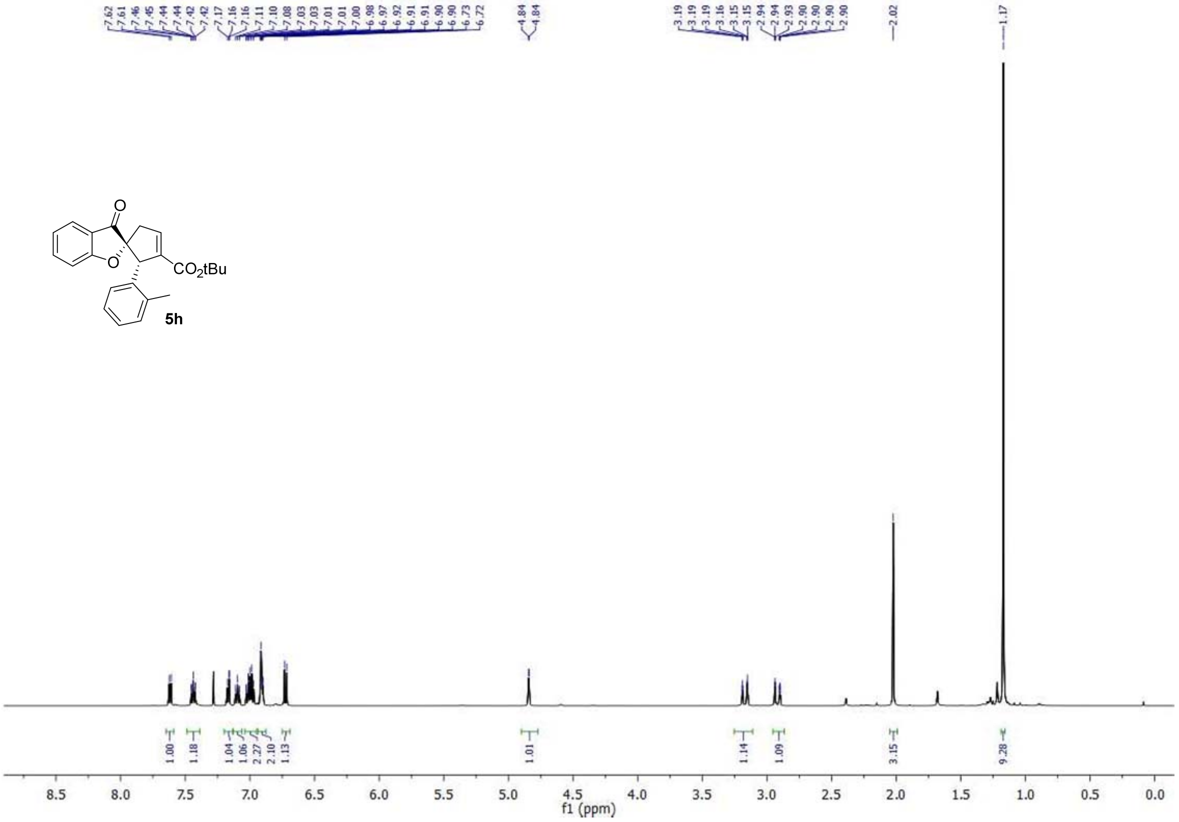

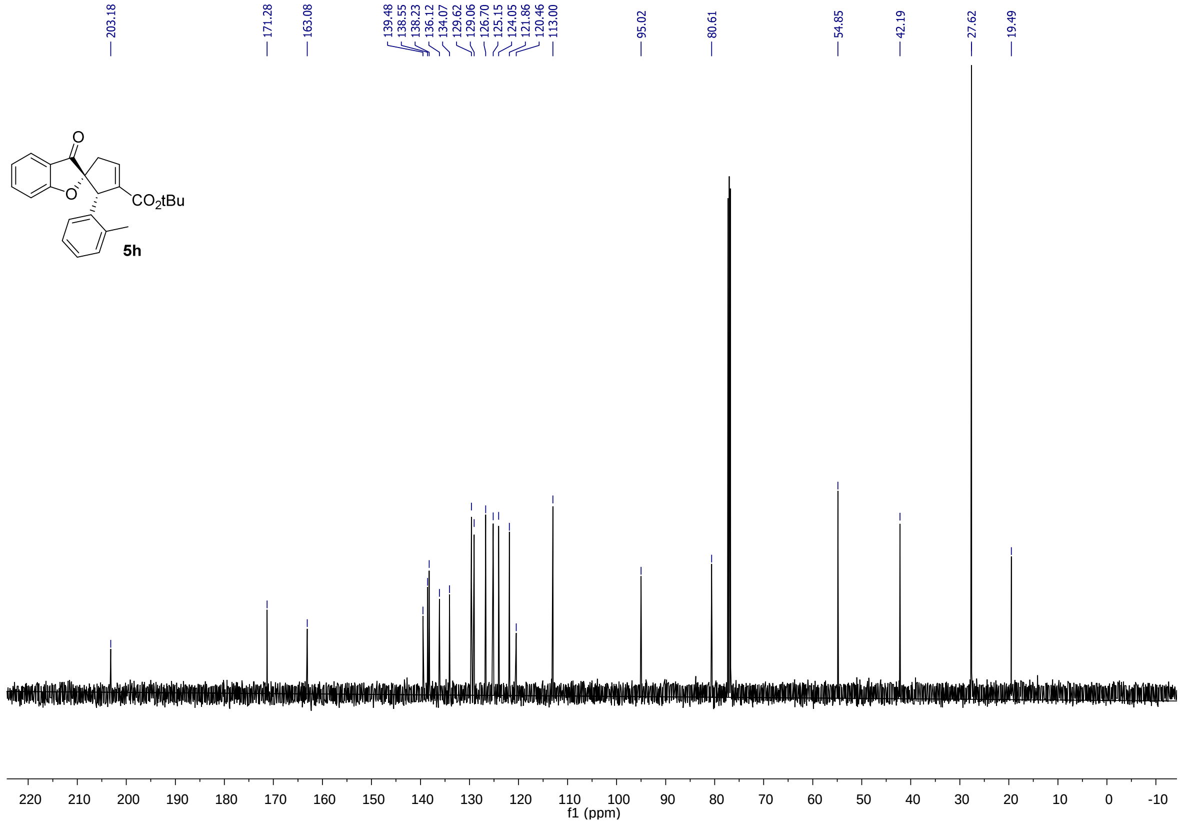

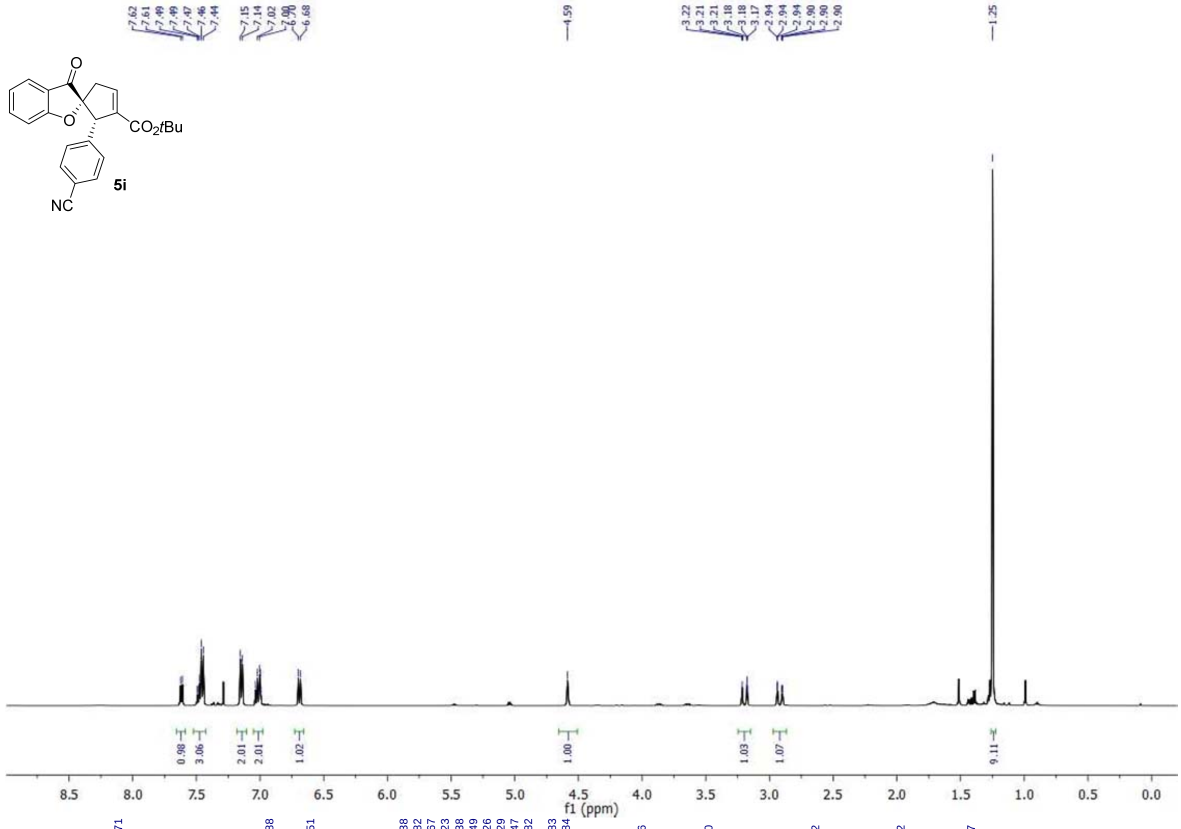
7.62
7.61
7.49
7.49
7.47
7.46
7.44
7.15
7.14
7.02
7.00
6.70
6.68
4.59
3.22
3.21
3.21
3.18
3.18
3.17
2.94
2.94
2.94
2.90
2.90
2.90
1.25
O
O
CO_2tBu
NC
5i
0.98
3.06
2.01
2.01
1.02
1.00
1.03
1.07
9.11
f1 (ppm)

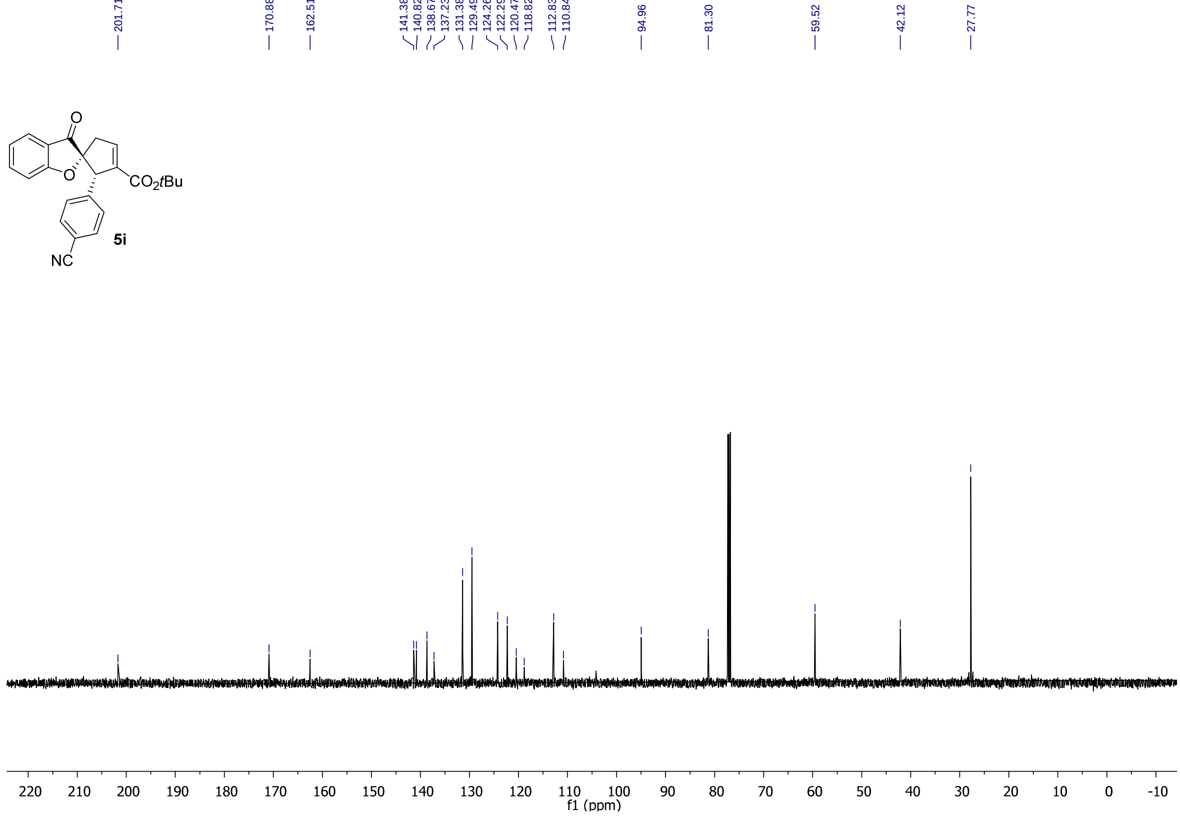
201.71
170.88
162.51
141.38
140.82
138.67
137.23
131.38
129.49
124.26
122.29
120.47
118.82
112.83
110.84
94.96
81.30
59.52
42.12
27.77
O
O
CO_2tBu
NC
5i
f1 (ppm)

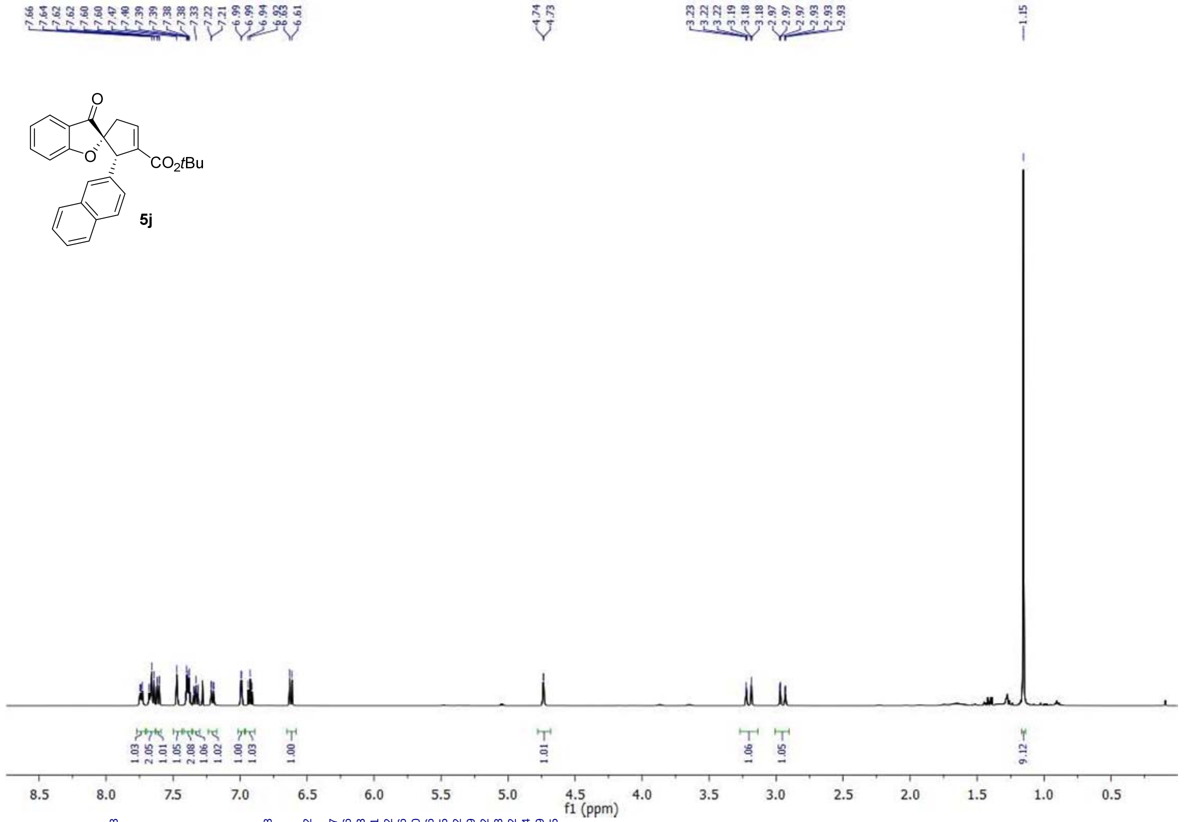
7.66 7.64 7.62 7.62 7.60 7.60 7.47 7.40 7.39 7.39 7.38 7.38 7.33 7.22 7.21 6.99 6.99 6.94 6.93 6.93 6.61
4.74 4.73
3.23 3.22 3.22 3.19 3.18 2.97 2.97 2.97 2.93 2.93 2.93
1.15
O
O
CO2tBu
5j
1.03 2.05 1.01 1.05 2.08 1.06 1.02 1.00 1.03 1.00 1.01 1.06 1.05 9.12
f1 (ppm)

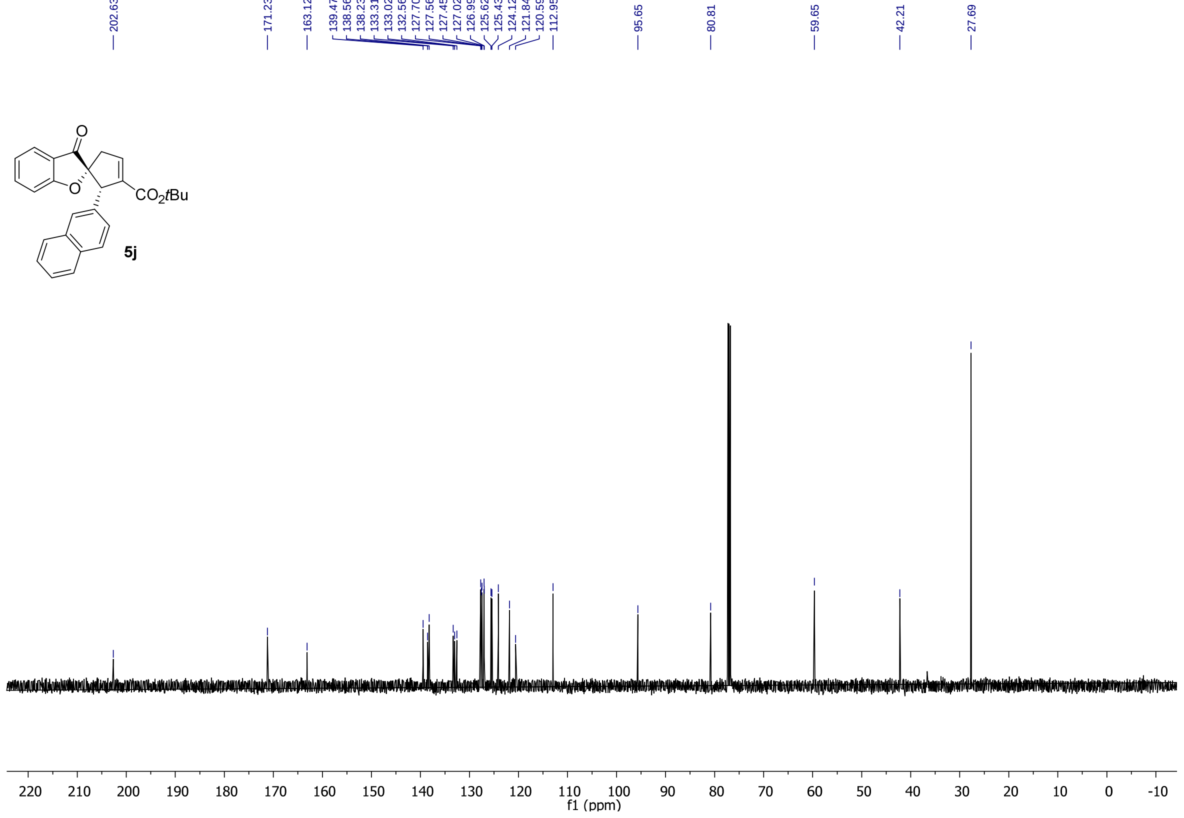
202.63
171.23
163.12
139.47 138.56 138.23 133.31 133.02 132.56 127.70 127.56 127.45 127.02 126.99 125.62 125.43 124.12 121.84 120.59 112.95
95.65
80.81
59.65
42.21
27.69
O
O
CO2tBu
5j
f1 (ppm)

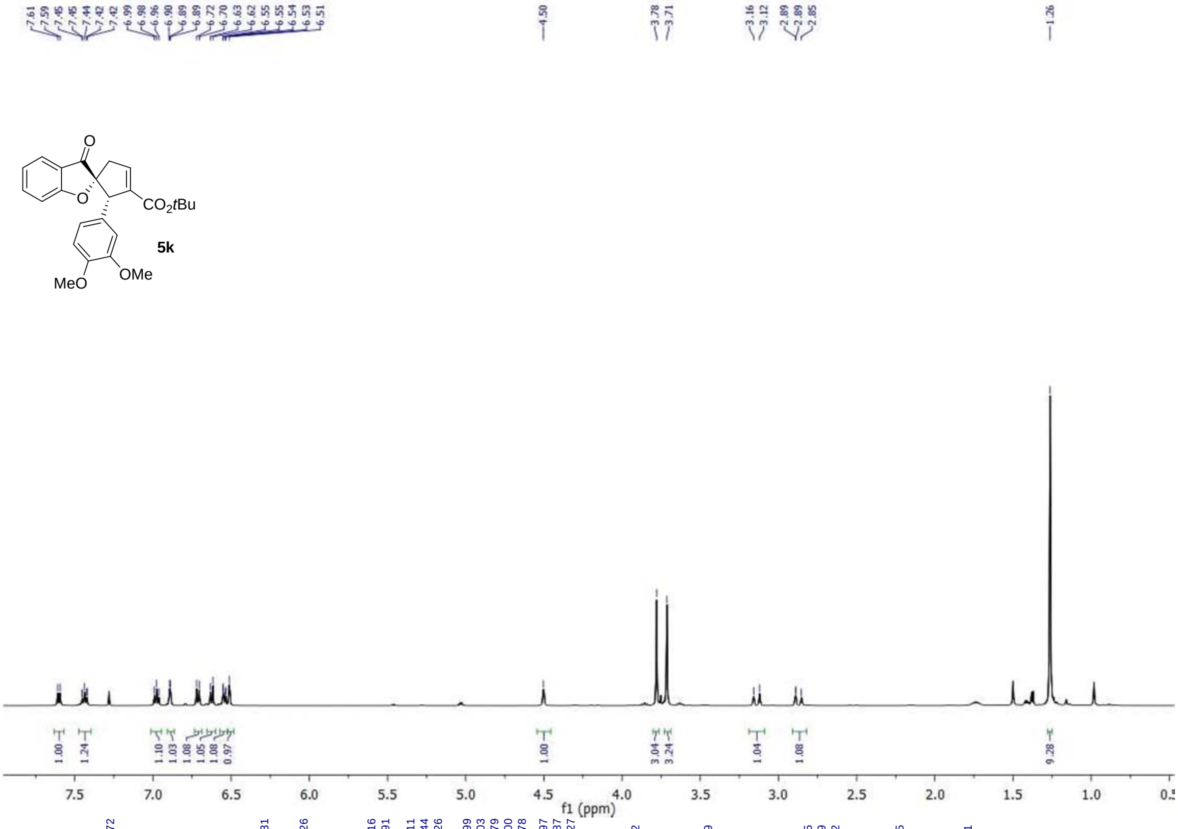
7.61
7.59
7.45
7.44
7.42
6.99
6.98
6.96
6.90
6.89
6.72
6.70
6.63
6.62
6.55
6.54
6.53
6.51
4.50
3.78
3.71
3.16
3.12
2.89
2.85
1.26
O
O
CO2tBu
5k
MeO
OMe
1.00
1.24
1.10
1.03
1.08
1.05
1.08
0.97
1.00
3.04
3.24
1.04
1.08
9.28
f1 (ppm)

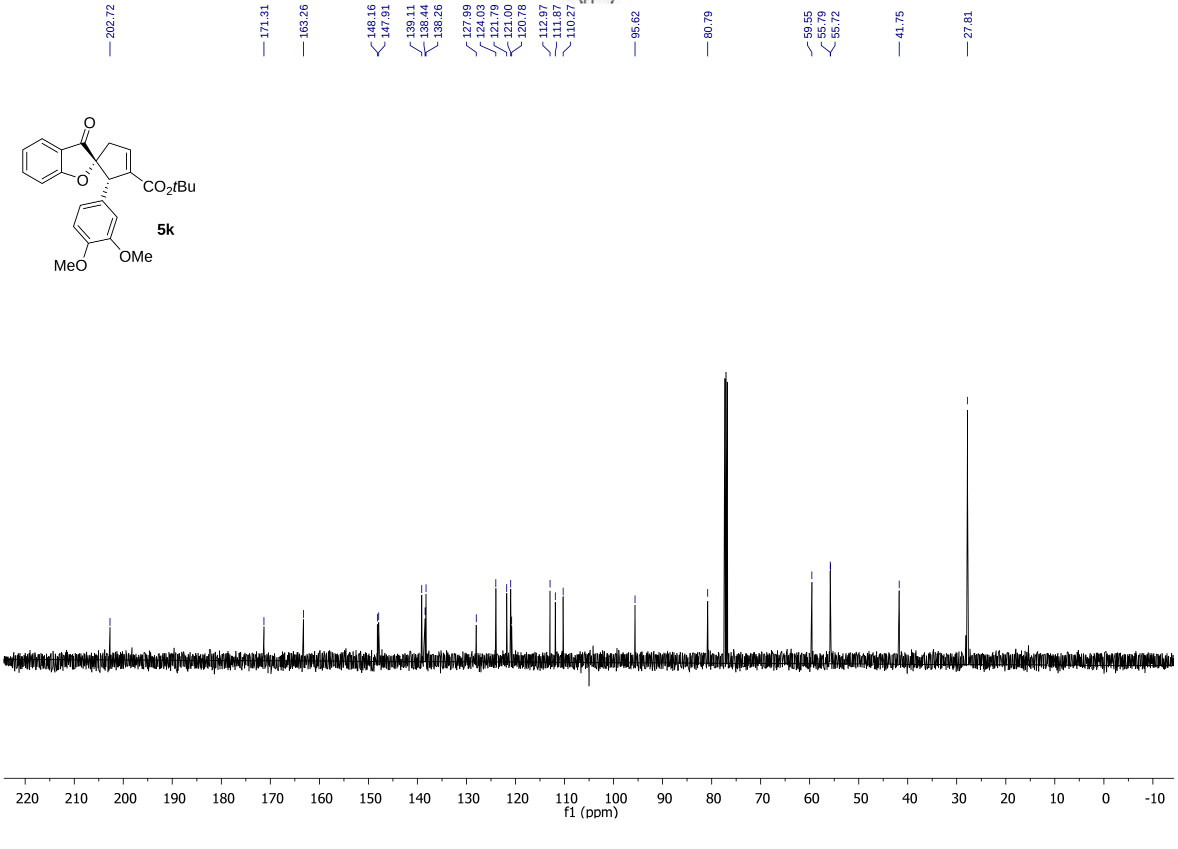
202.72
171.31
163.26
148.16
147.91
139.11
138.44
138.26
127.99
124.03
121.79
121.00
120.78
112.97
111.87
110.27
95.62
80.79
59.55
55.79
55.72
41.75
27.81
O
O
CO2tBu
5k
MeO
OMe
f1 (ppm)

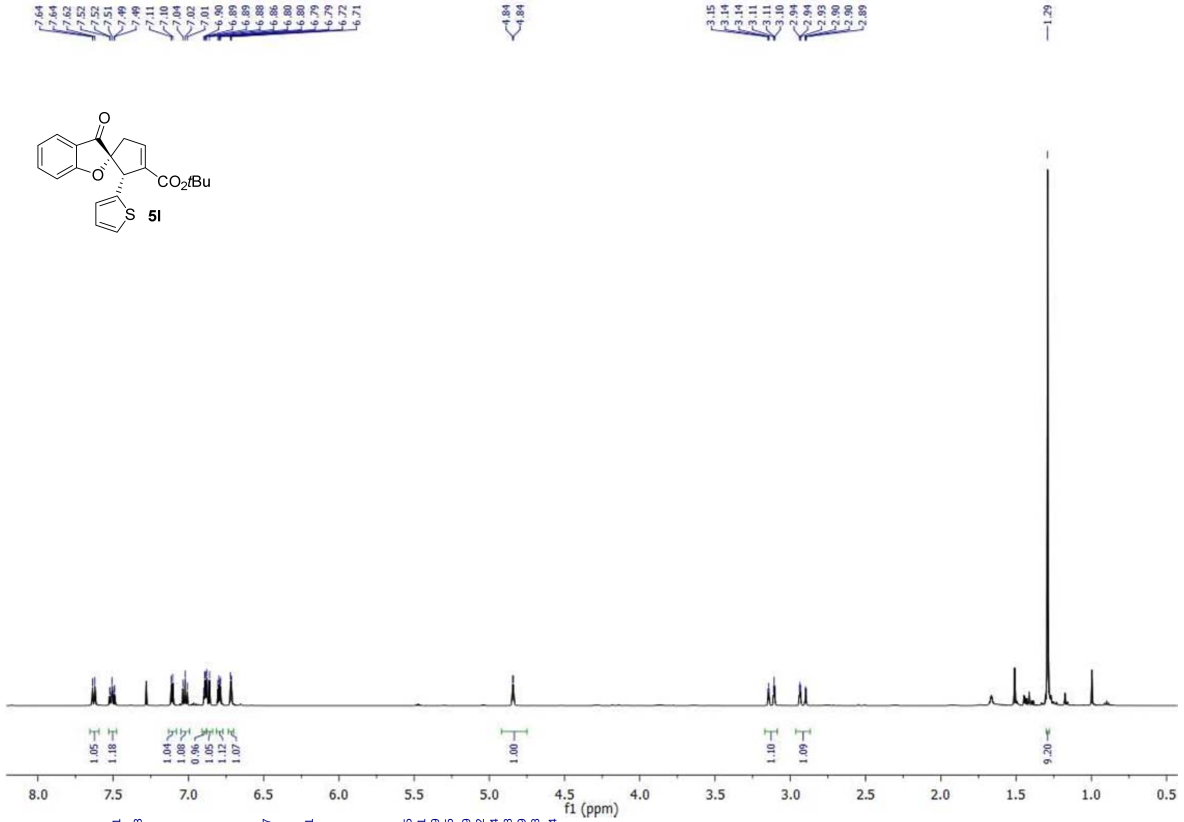
CO2tBu
S
5l
1.05
1.18
1.04
1.08
0.96
1.05
1.12
1.07
1.00
1.10
1.09
9.20
f1 (ppm)

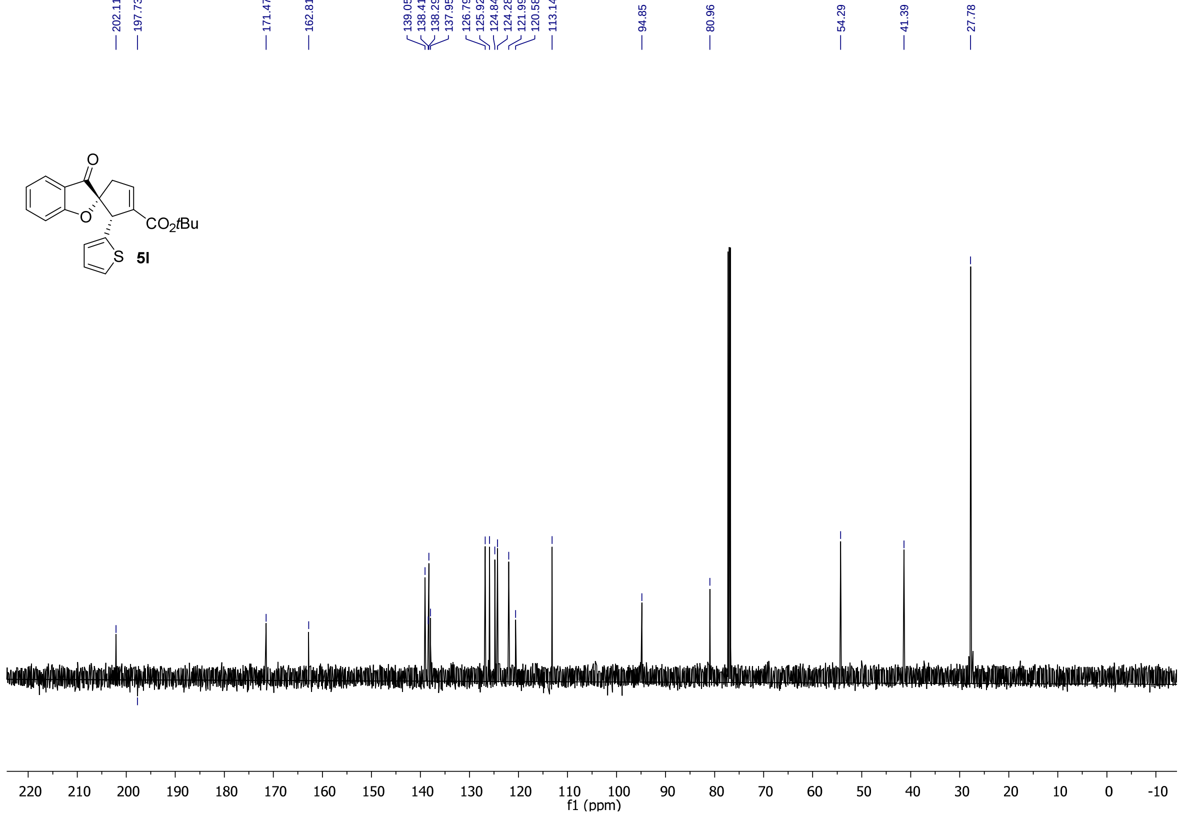
202.11
197.73
171.47
162.81
139.05
138.41
138.29
137.95
126.79
125.92
124.84
124.28
121.99
120.58
113.14
94.85
80.96
54.29
41.39
27.78
CO2tBu
S
5l
f1 (ppm)

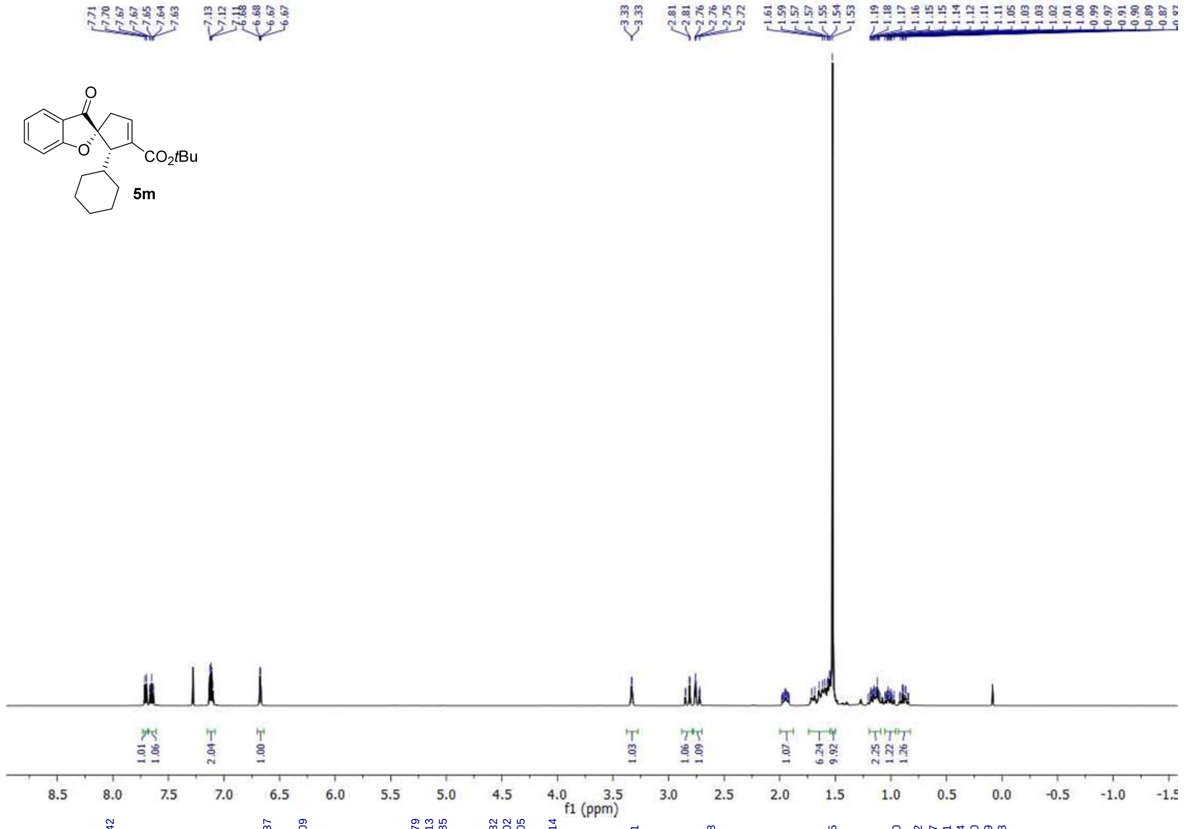
O
O
CO2tBu
5m
1.01
1.06
2.04
1.00
1.03
1.06
1.09
1.07
6.24
9.92
2.25
1.22
1.26
f1 (ppm)

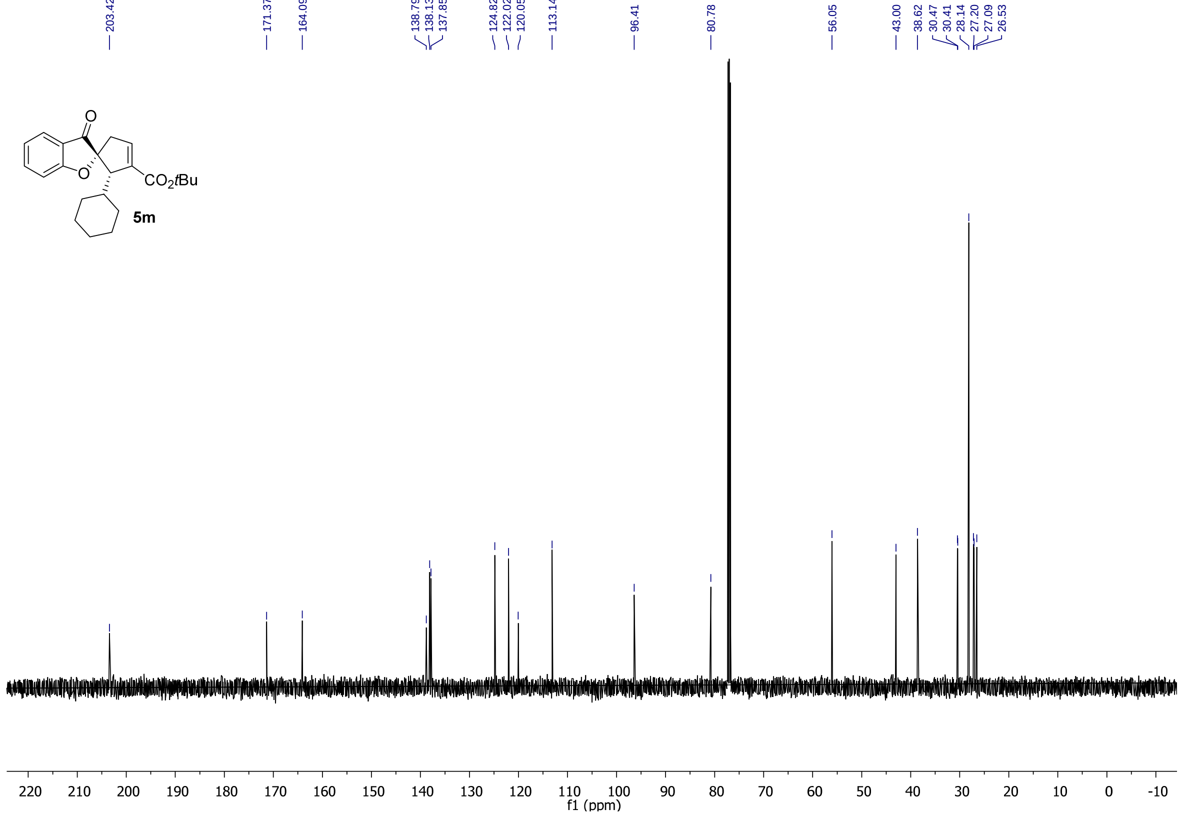
203.42
171.37
164.09
138.79
138.13
137.85
124.82
122.02
120.05
113.14
96.41
80.78
56.05
43.00
38.62
30.47
30.41
28.14
27.20
27.09
26.53
O
O
CO2tBu
5m
f1 (ppm)

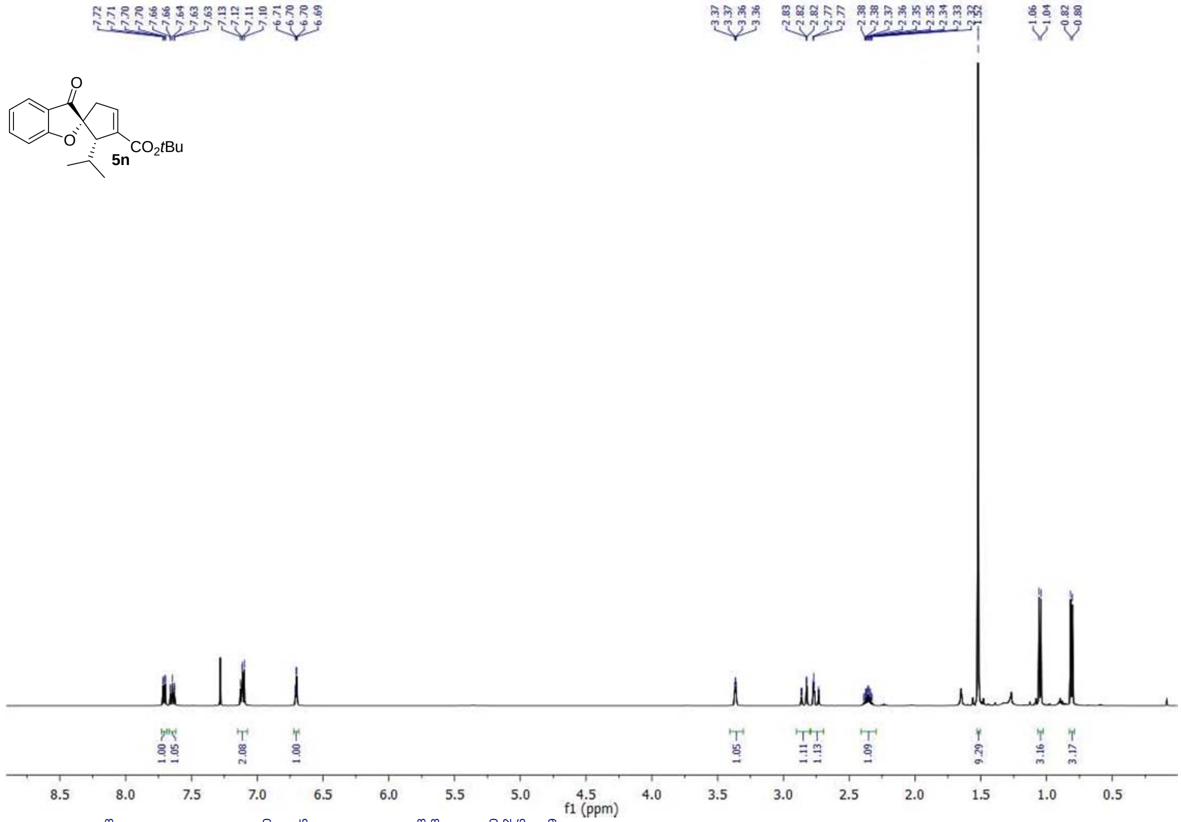

7.72
7.71
7.70
7.70
7.66
7.66
7.64
7.63
7.63
7.13
7.12
7.11
7.10
6.71
6.70
6.70
6.69
3.37
3.37
3.36
3.36
2.83
2.82
2.82
2.77
2.77
2.38
2.38
2.37
2.36
2.35
2.35
2.34
2.33
2.32
1.52
1.06
1.04
0.82
0.80
O
O
CO2tBu
5n
1.00
1.05
2.08
1.00
1.05
1.11
1.13
1.09
9.29
3.16
3.17
8.5
8.0
7.5
7.0
6.5
6.0
5.5
5.0
4.5
4.0
3.5
3.0
2.5
2.0
1.5
1.0
0.5
f1 (ppm)

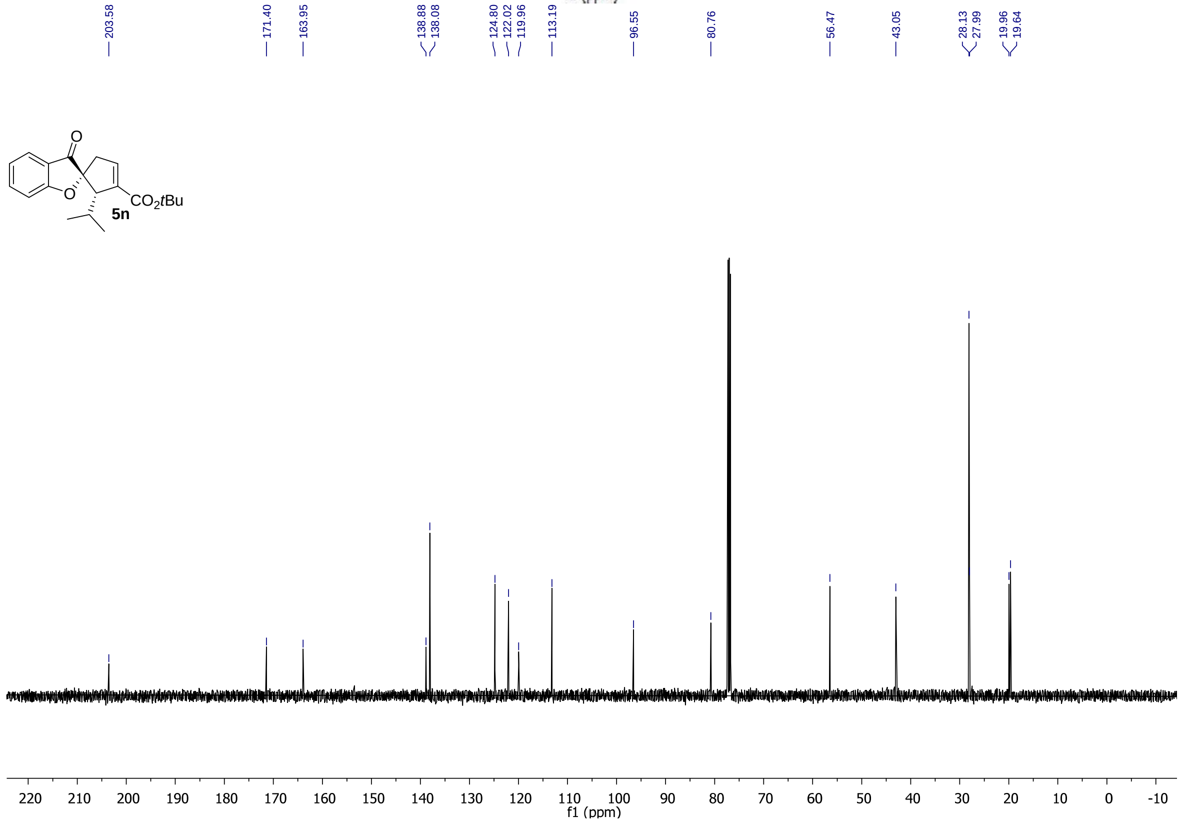

203.58
171.40
163.95
138.88
138.08
124.80
122.02
119.96
113.19
96.55
80.76
56.47
43.05
28.13
27.99
19.96
19.64
O
O
CO2tBu
5n
220
210
200
190
180
170
160
150
140
130
120
110
100
90
80
70
60
50
40
30
20
10
0
-10
f1 (ppm)

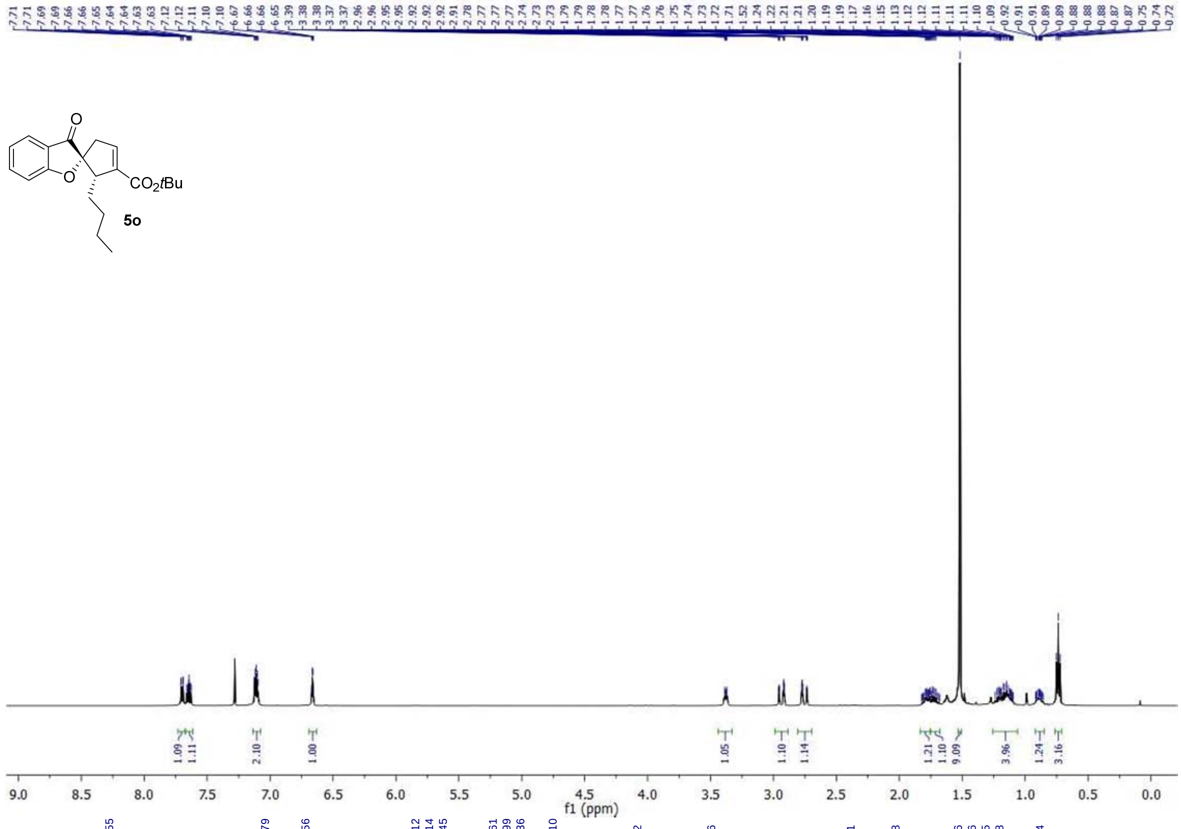
O
O
CO2tBu
5o
1.09
1.11
2.10
1.00
1.05
1.10
1.14
1.21
1.10
9.09
3.96
1.24
3.16
9.0 8.5 8.0 7.5 7.0 6.5 6.0 5.5 5.0 4.5 4.0 3.5 3.0 2.5 2.0 1.5 1.0 0.5 0.0
f1 (ppm)

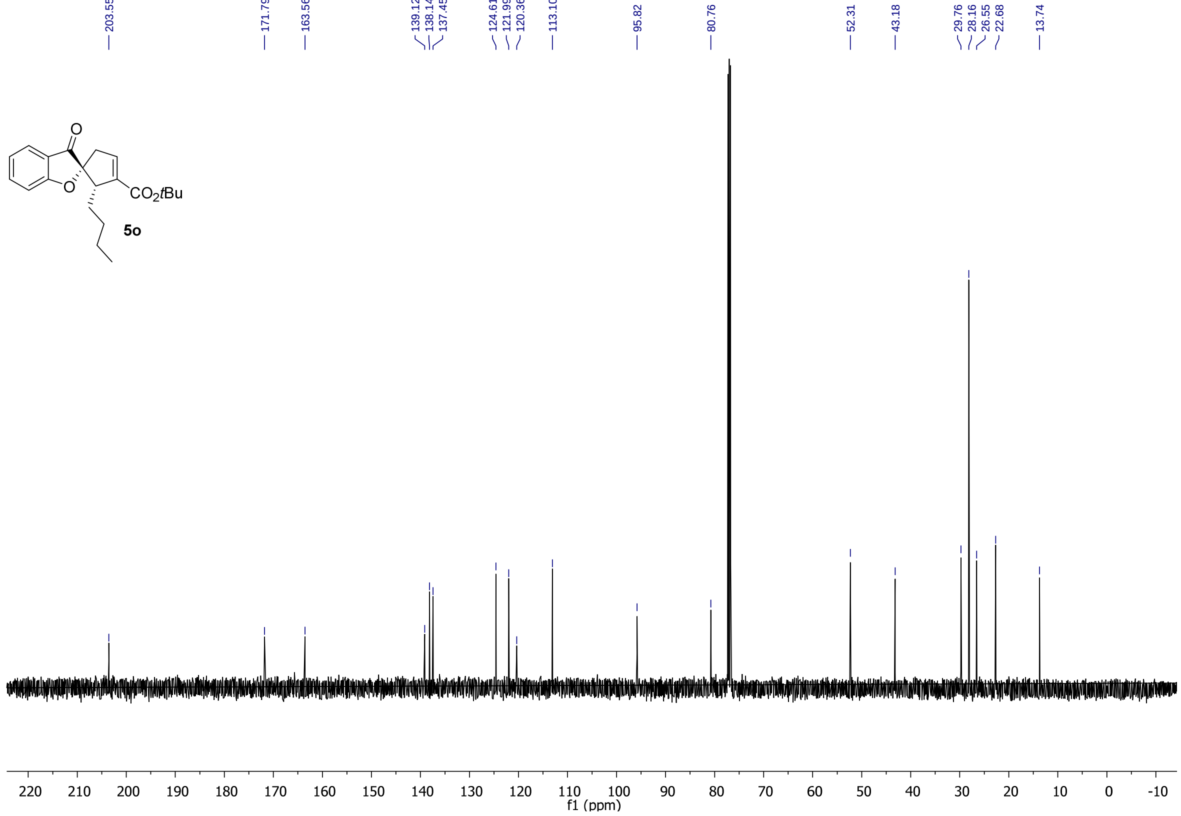
203.55
171.79
163.56
139.12
138.14
137.45
124.61
121.99
120.36
113.10
95.82
80.76
52.31
43.18
29.76
28.16
26.55
22.68
13.74
O
O
CO2tBu
5o
220 210 200 190 180 170 160 150 140 130 120 110 100 90 80 70 60 50 40 30 20 10 0 -10
f1 (ppm)

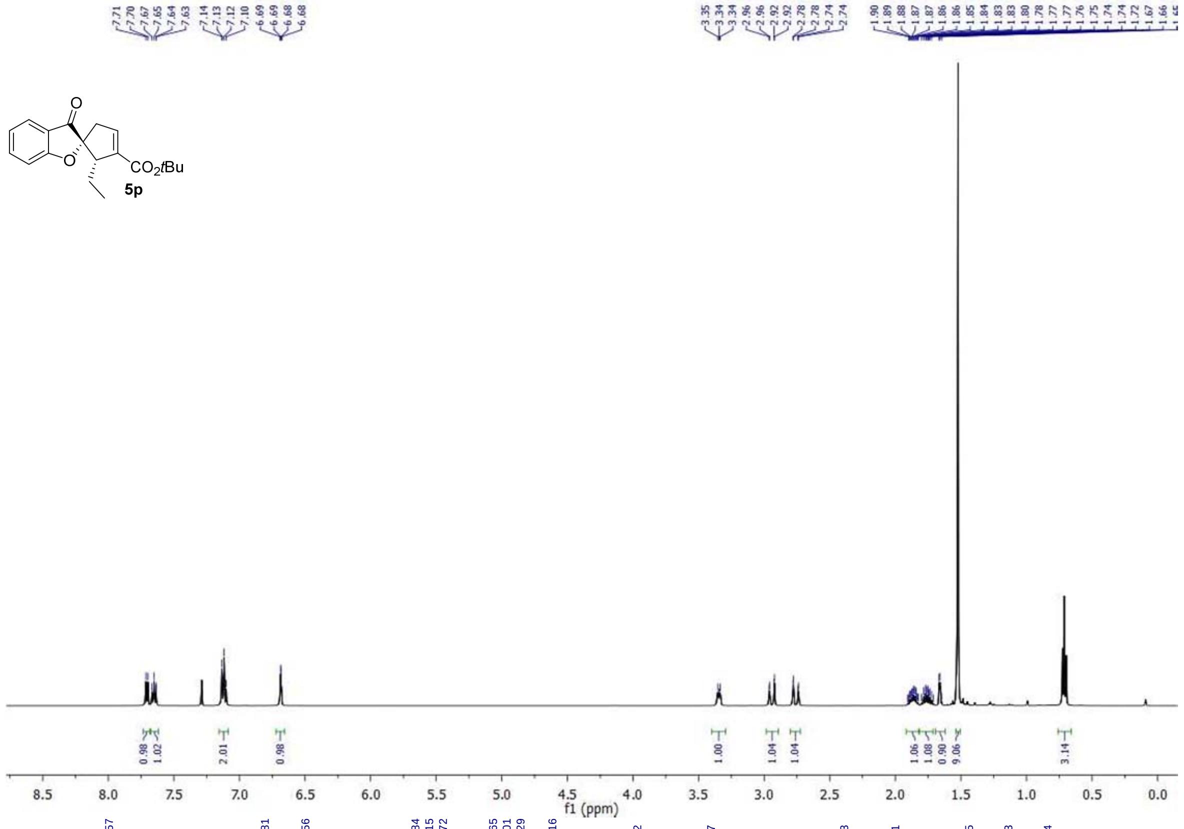

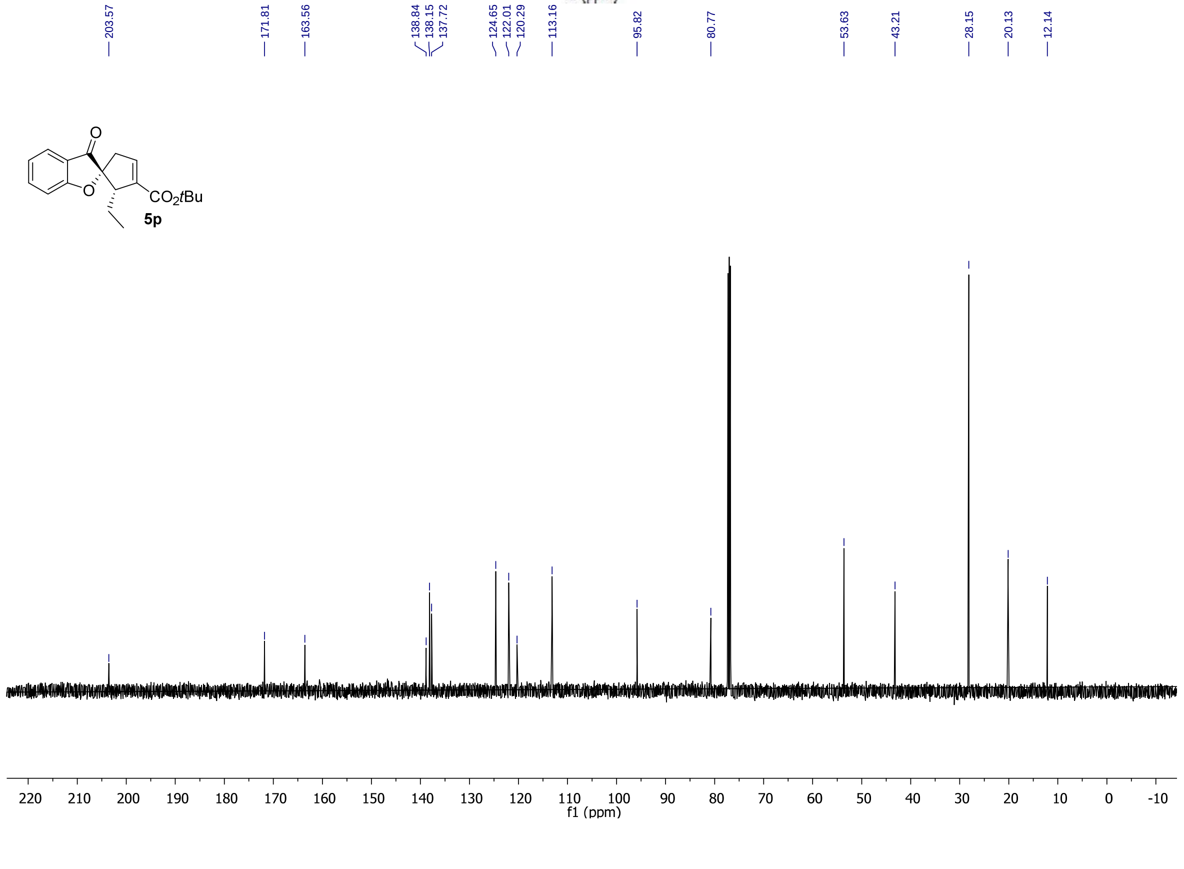

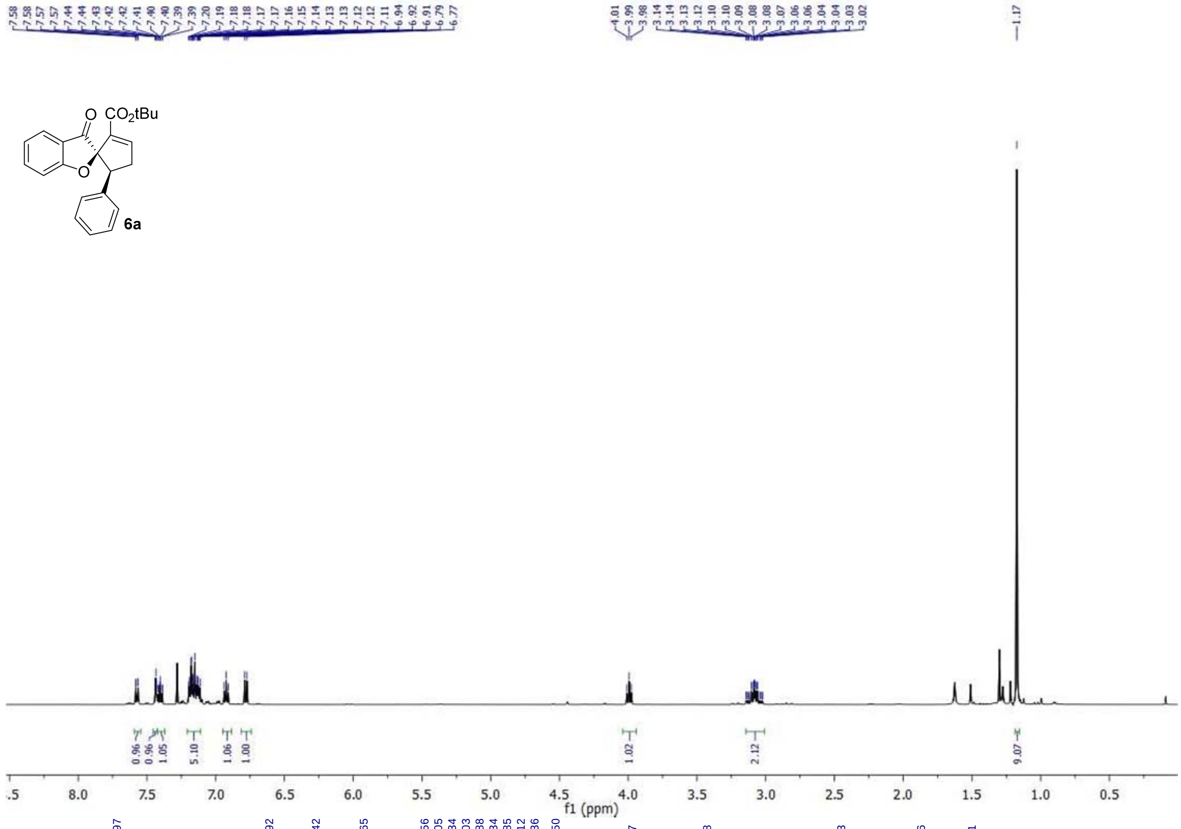

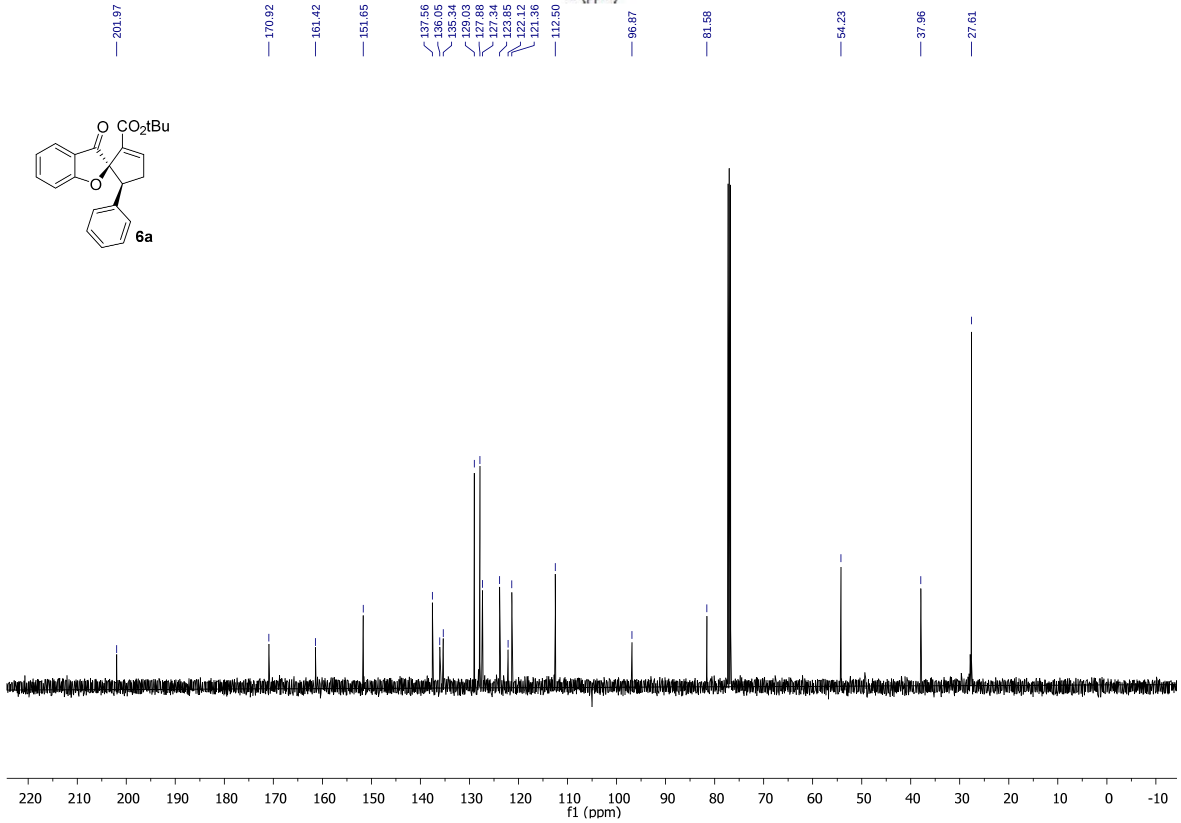

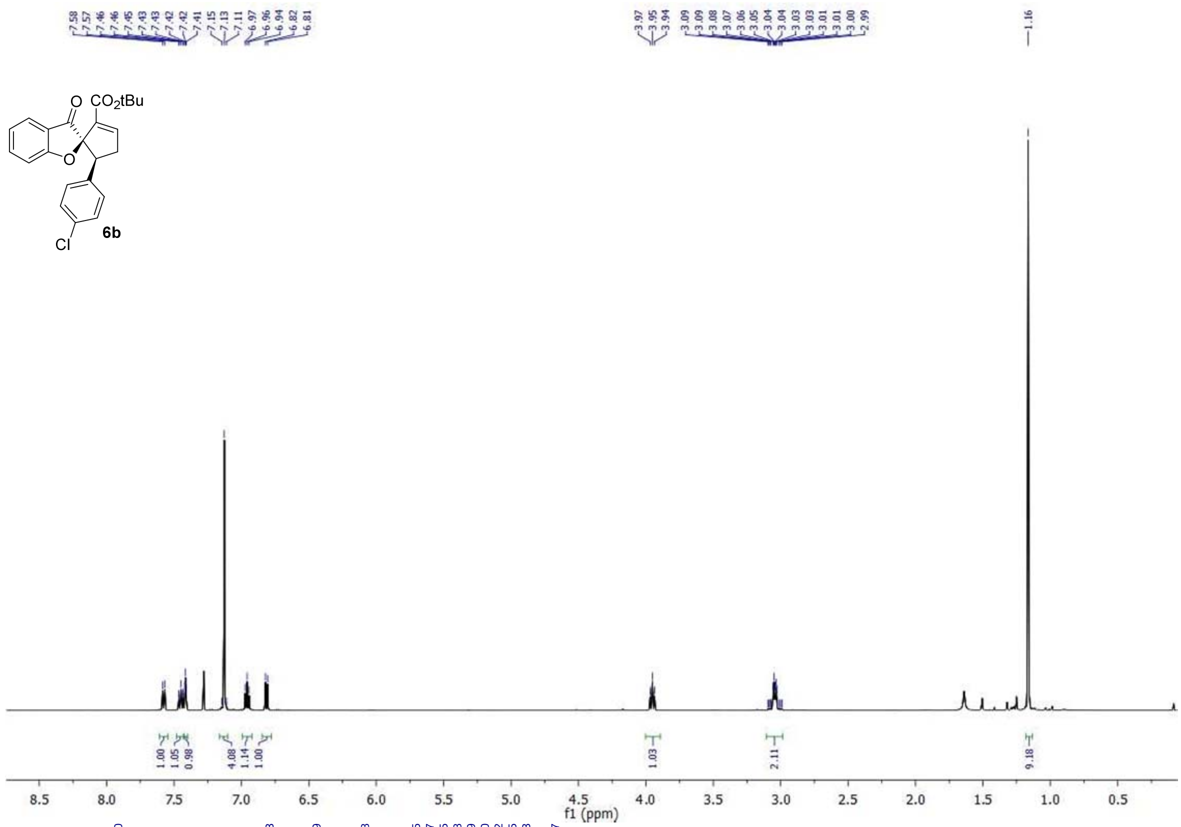
O CO2tBu
O
Cl
6b
7.58 7.57 7.46 7.46 7.45 7.43 7.43 7.42 7.42 7.41 7.15 7.13 7.11 6.97 6.96 6.94 6.82 6.81
3.97 3.95 3.94 3.09 3.09 3.08 3.07 3.06 3.05 3.04 3.04 3.03 3.03 3.01 3.01 3.00 2.99
1.16
1.00 1.05 0.98 4.08 1.14 1.00 1.03 2.11 9.18
f1 (ppm)

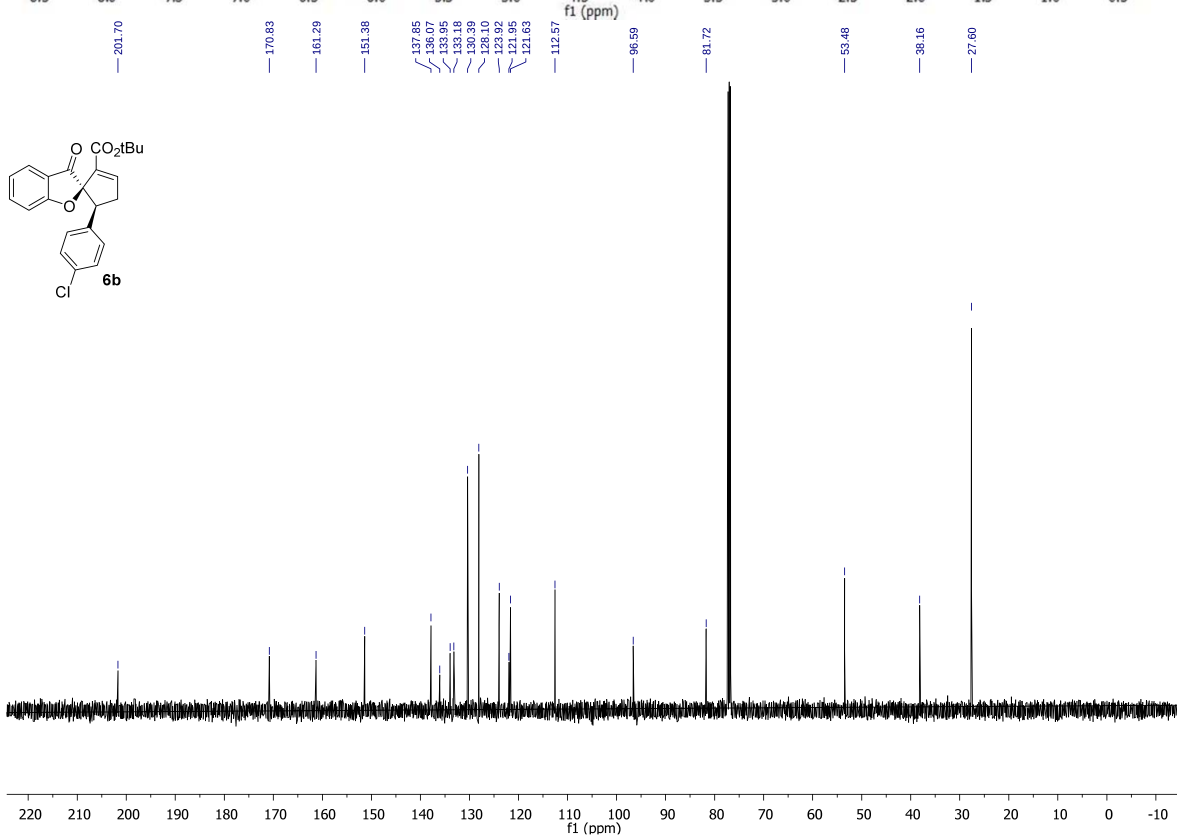
201.70 170.83 161.29 151.38 137.85 136.07 133.95 133.18 130.39 128.10 123.92 121.95 121.63 112.57 96.59 81.72 53.48 38.16 27.60
O CO2tBu
O
Cl
6b
f1 (ppm)

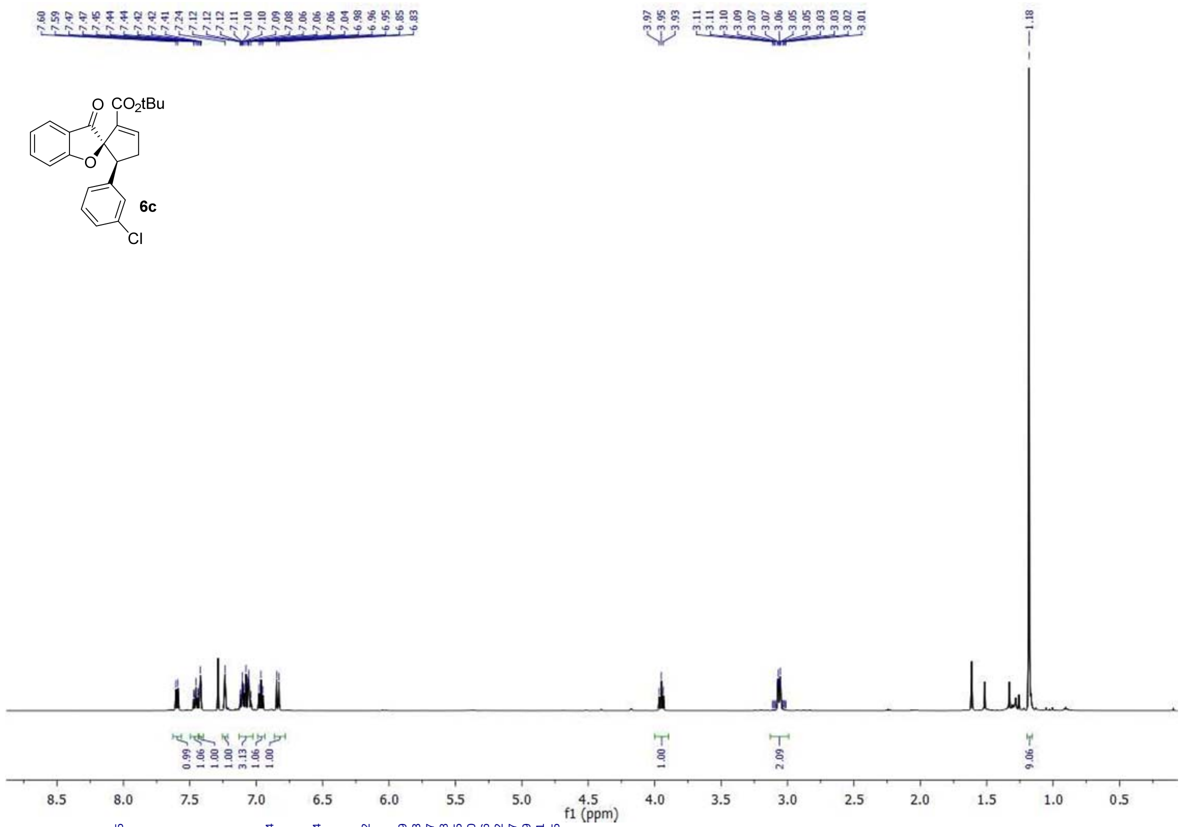
CO2tBu
6c
Cl
f1 (ppm)

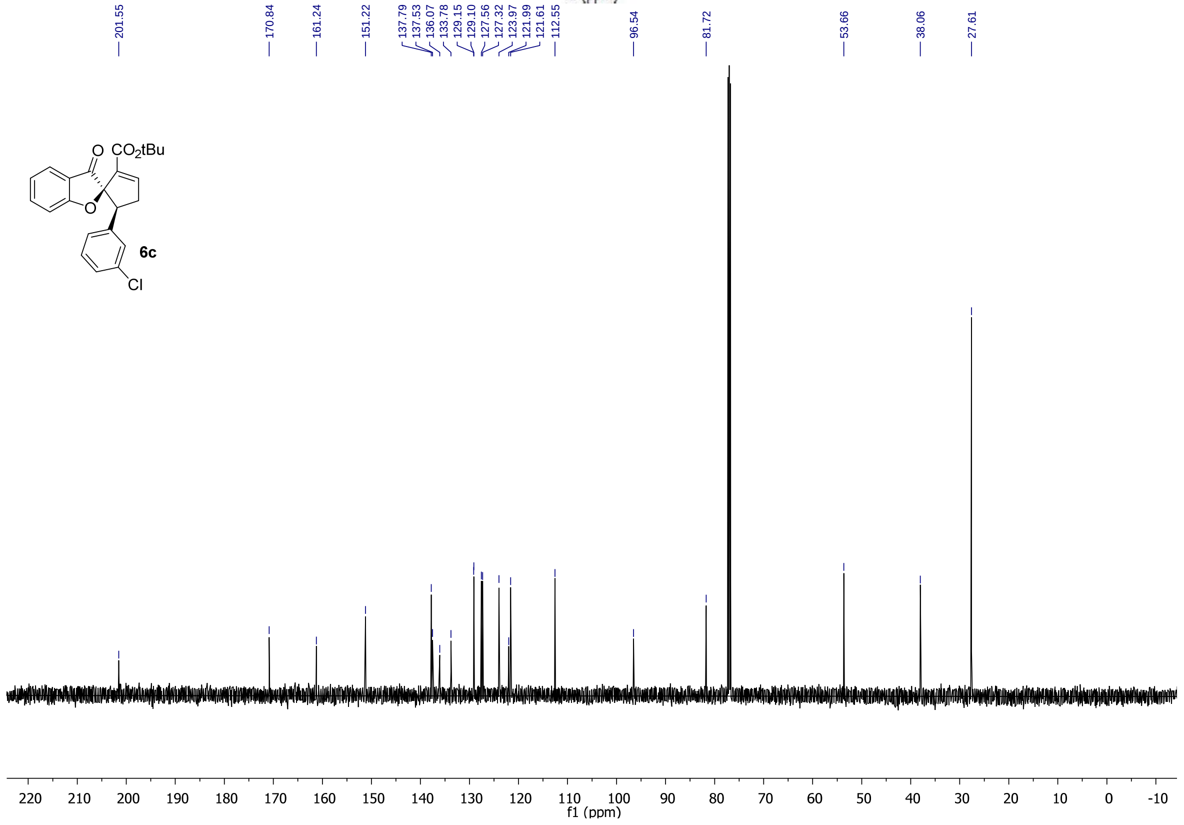
201.55
170.84
161.24
151.22
137.79
137.53
136.07
133.78
129.15
129.10
127.56
127.32
123.97
121.99
121.61
112.55
96.54
81.72
53.66
38.06
27.61
CO2tBu
6c
Cl
f1 (ppm)

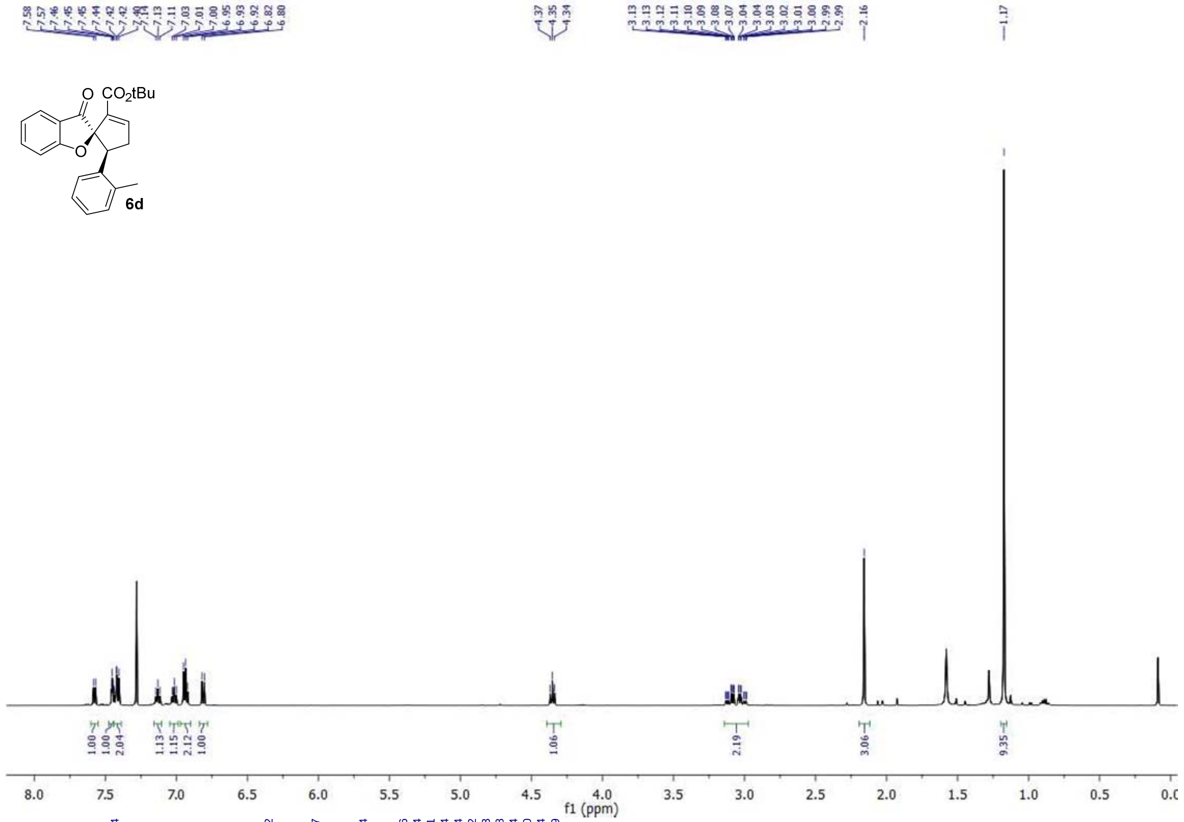
7.58
7.57
7.46
7.45
7.45
7.44
7.42
7.42
7.13
7.11
7.03
7.01
7.00
6.95
6.93
6.92
6.82
6.80
4.37
4.35
4.34
3.13
3.13
3.12
3.11
3.10
3.09
3.08
3.07
3.04
3.03
3.03
3.02
3.01
3.00
2.99
2.99
2.16
1.17
O
CO2tBu
O
6d
1.00
1.00
2.04
1.13
1.15
2.12
1.00
1.06
2.19
3.06
9.35
8.0
7.5
7.0
6.5
6.0
5.5
5.0
4.5
4.0
3.5
3.0
2.5
2.0
1.5
1.0
0.5
f1 (ppm)

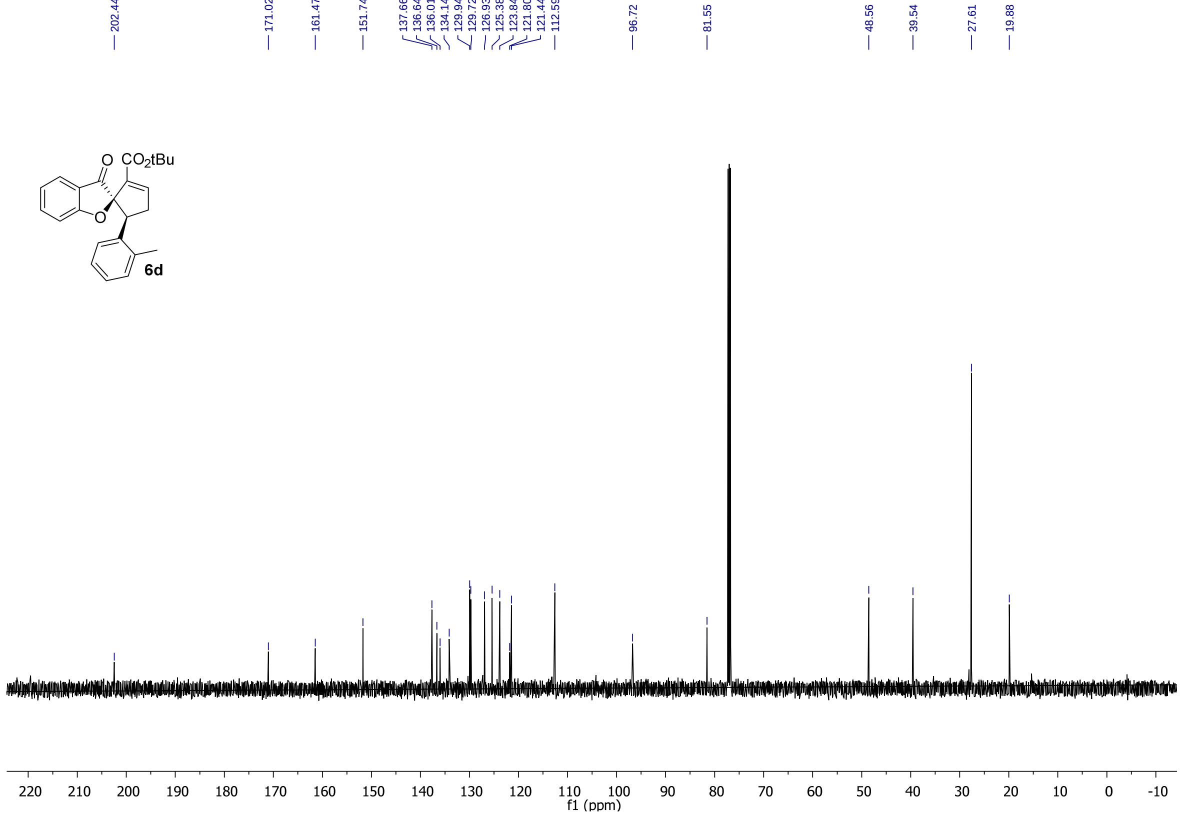
202.44
171.02
161.47
151.74
137.66
136.64
136.01
134.14
129.94
129.72
126.93
125.38
123.84
121.80
121.44
112.59
96.72
81.55
48.56
39.54
27.61
19.88
O
CO2tBu
O
6d
220
210
200
190
180
170
160
150
140
130
120
110
100
90
80
70
60
50
40
30
20
10
0
-10
f1 (ppm)

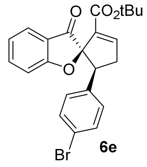

6e

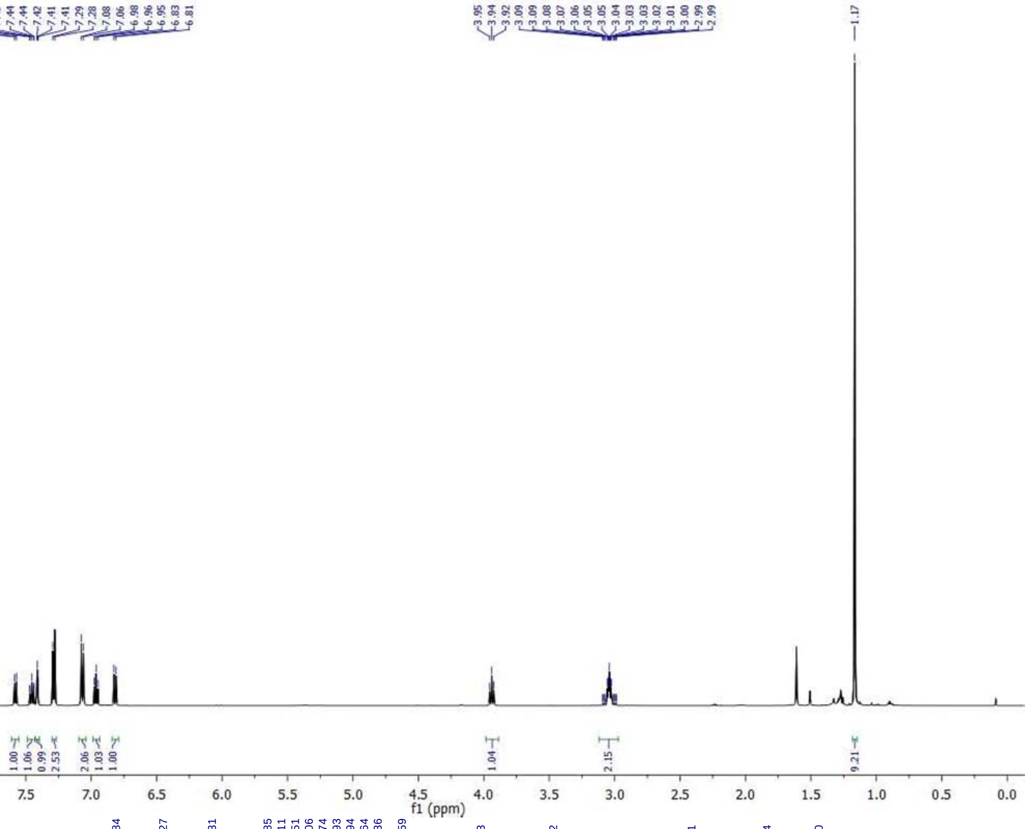

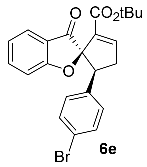

6e

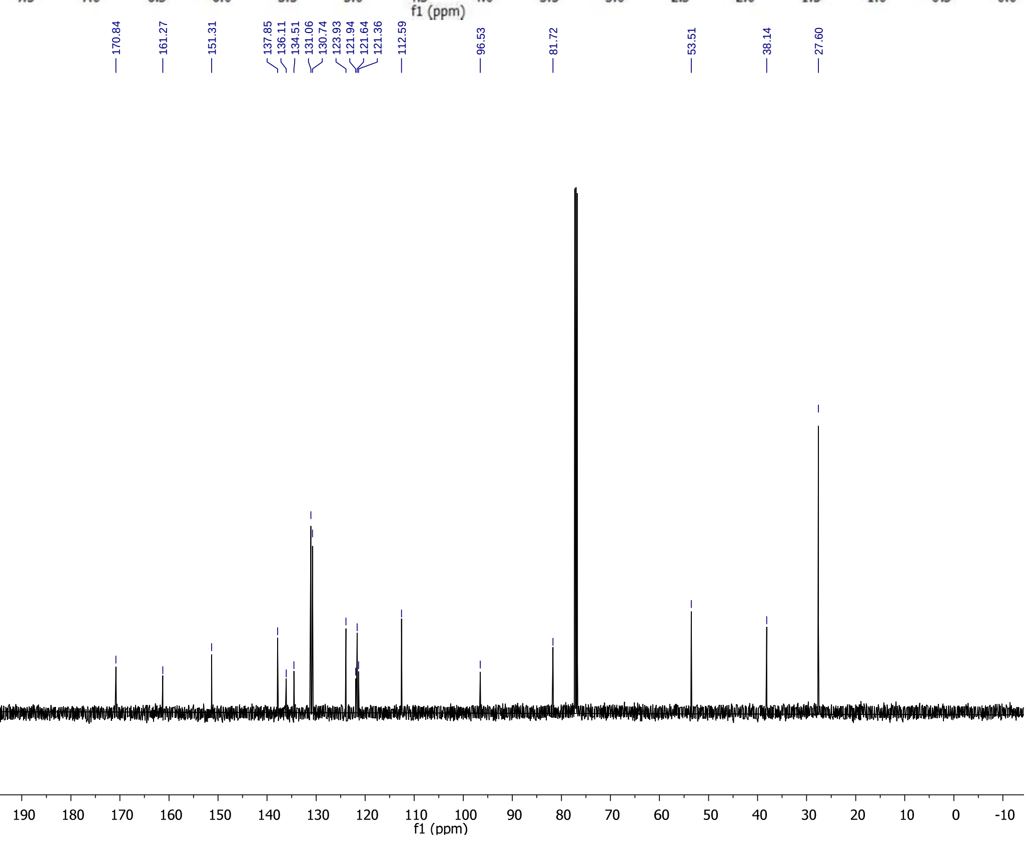

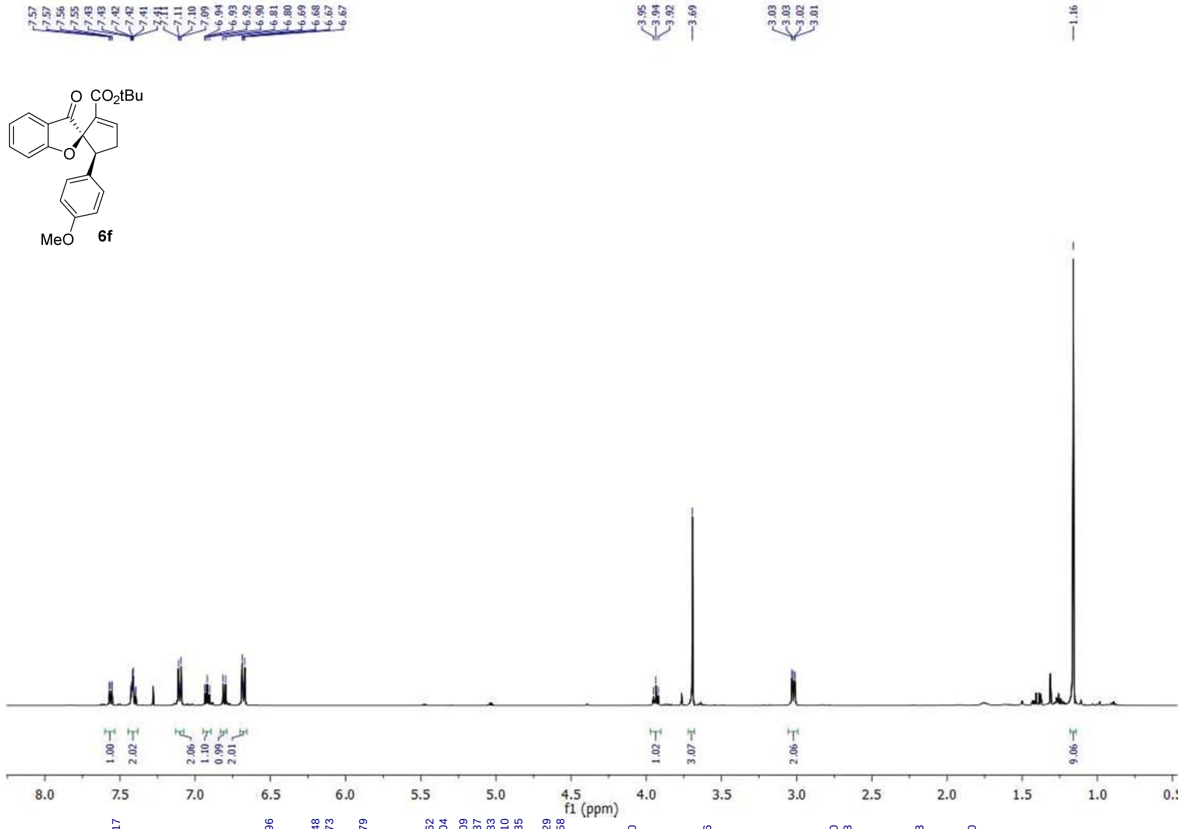

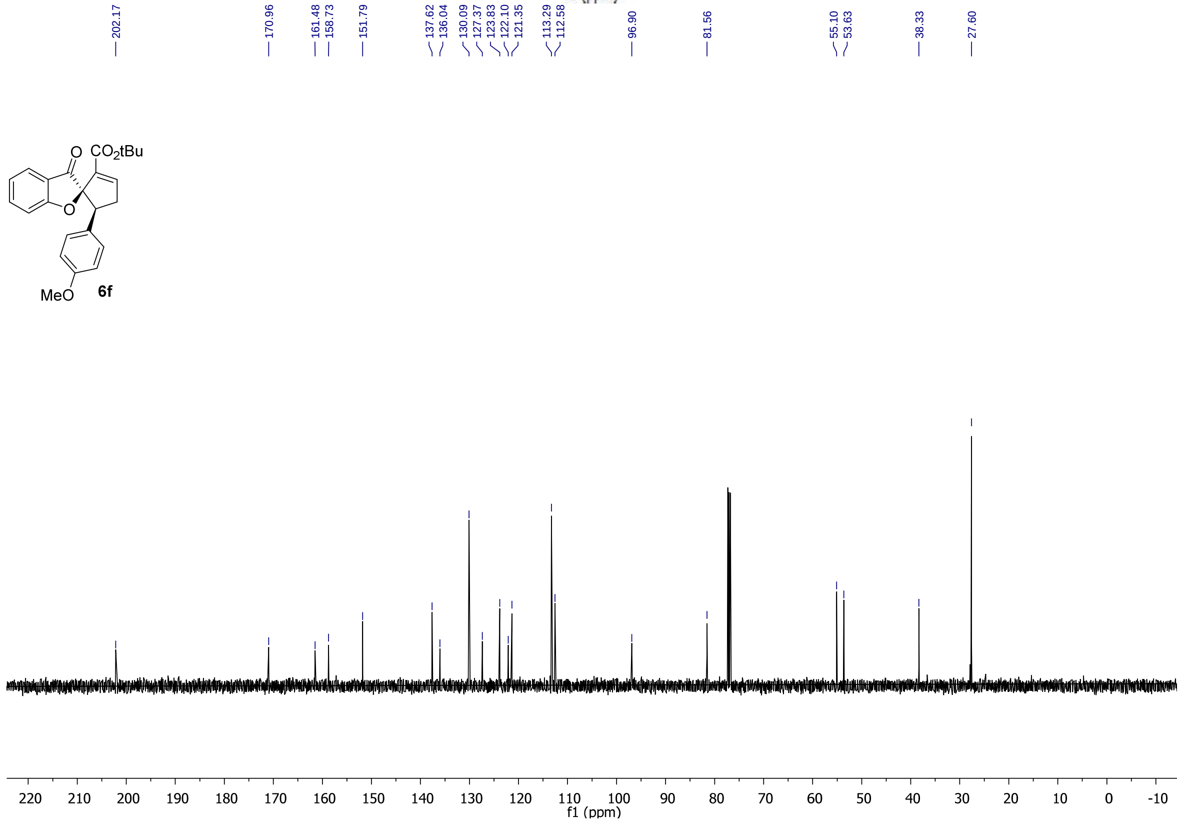

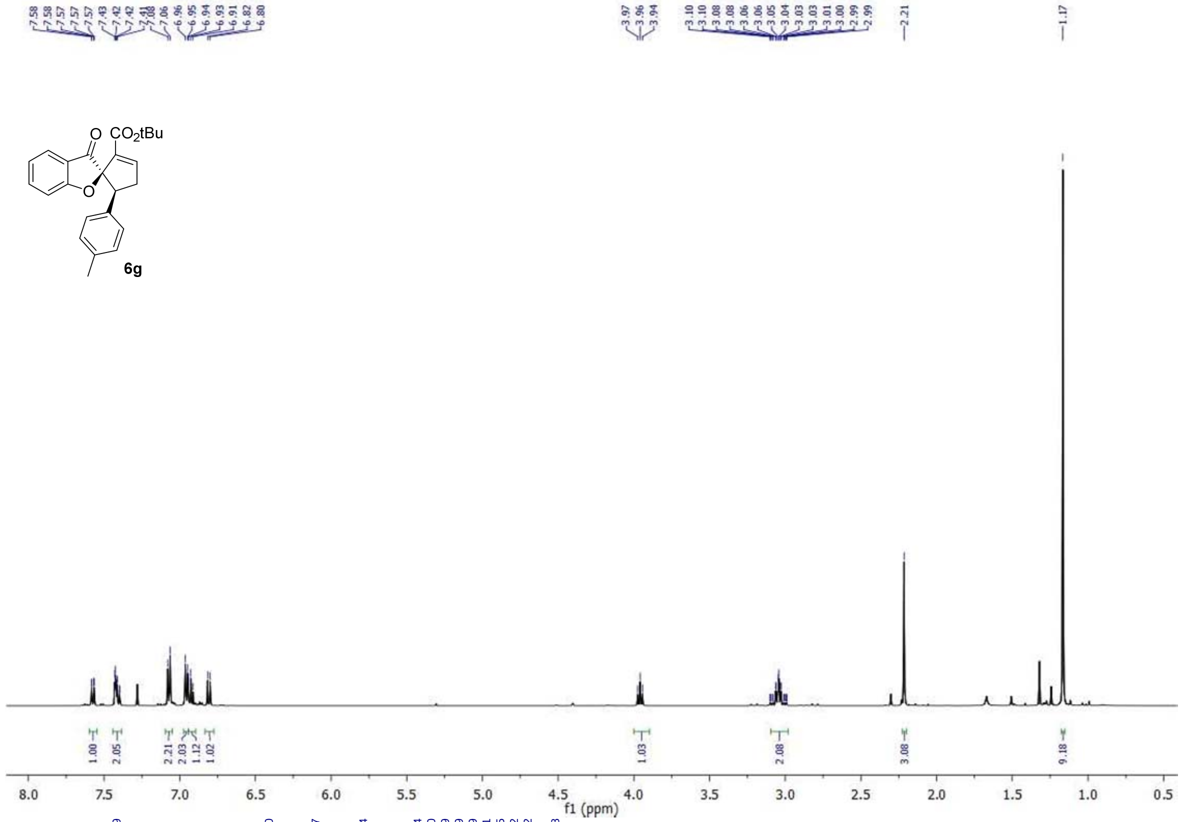
7.58 7.58 7.57 7.57 7.43 7.42 7.42 7.41 7.08 7.06 6.96 6.95 6.94 6.93 6.91 6.82 6.80
3.97 3.96 3.94
3.10 3.10 3.08 3.08 3.06 3.06 3.05 3.04 3.03 3.03 3.01 3.00 2.99 2.99
2.21
1.17
O
CO2tBu
O
6g
1.00 2.05 2.21 2.03 1.12 1.02 1.03 2.08 3.08 9.18
8.0 7.5 7.0 6.5 6.0 5.5 5.0 4.5 4.0 3.5 3.0 2.5 2.0 1.5 1.0 0.5
f1 (ppm)

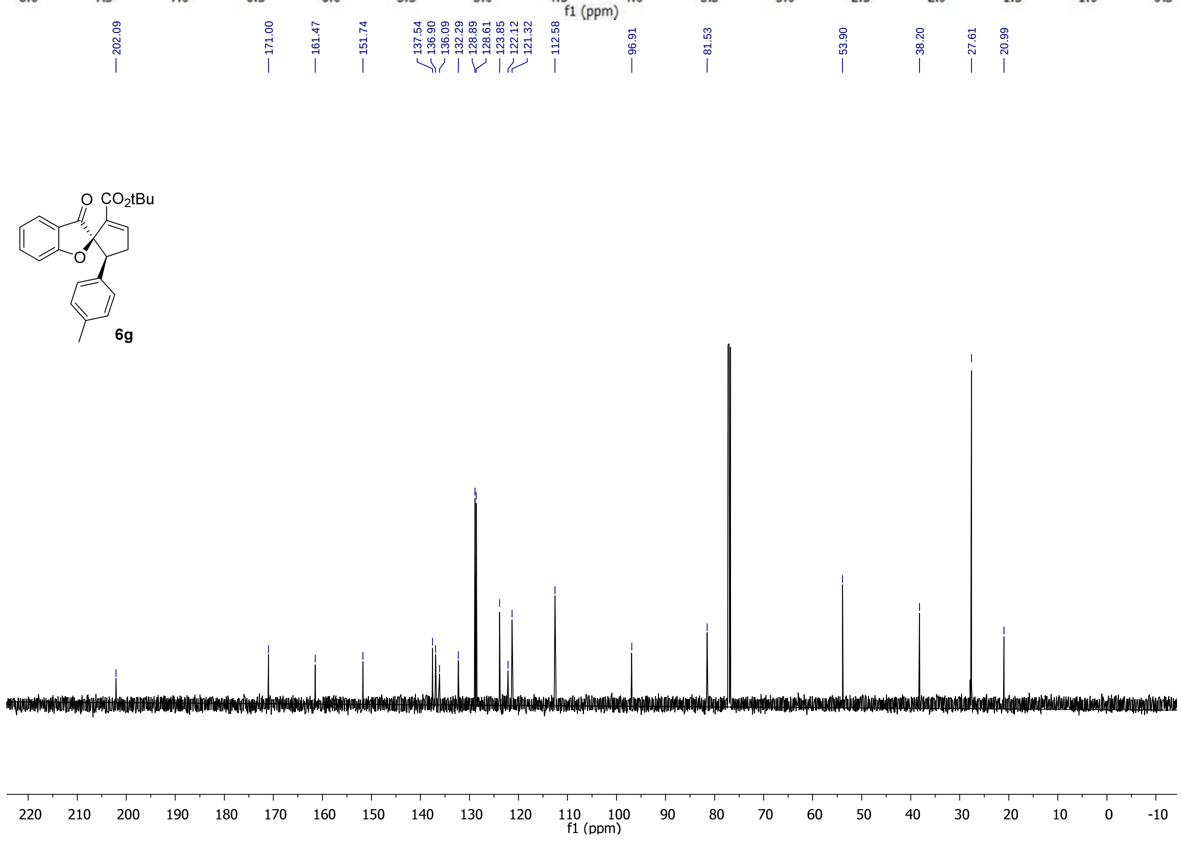
202.09
171.00
161.47
151.74
137.54 136.90 136.09 132.29 128.89 128.61 123.85 122.12 121.32
112.58
96.91
81.53
53.90
38.20
27.61
20.99
O
CO2tBu
O
6g
220 210 200 190 180 170 160 150 140 130 120 110 100 90 80 70 60 50 40 30 20 10 0 -10
f1 (ppm)

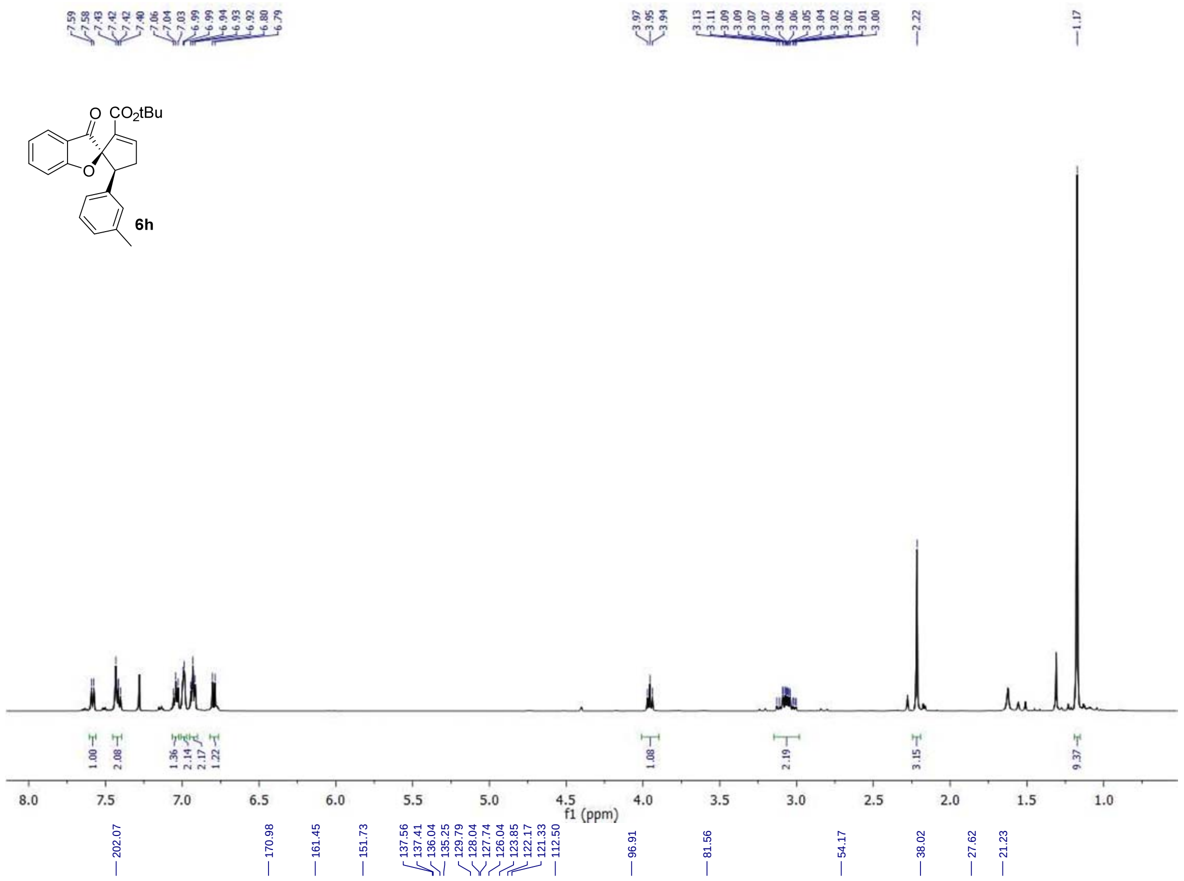

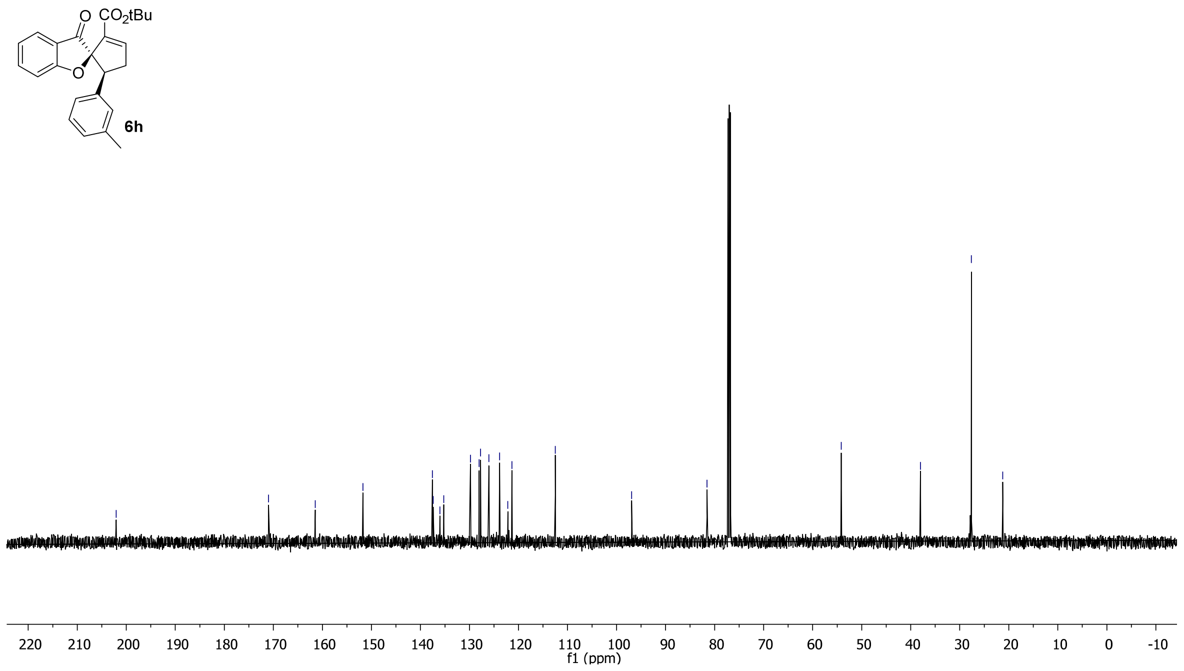

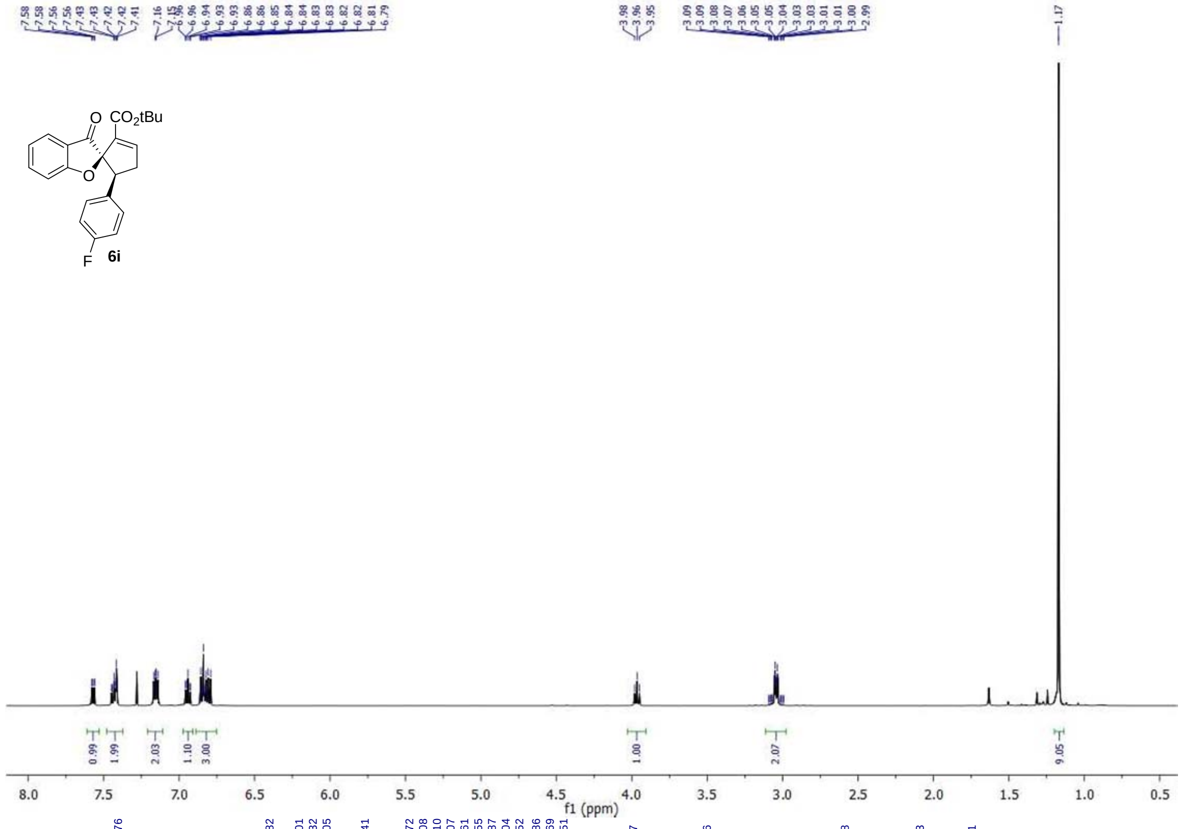

CO2tBu
O
O
F
6i
0.99
1.99
2.03
1.10
3.00
1.00
2.07
9.05
8.0 7.5 7.0 6.5 6.0 5.5 5.0 4.5 4.0 3.5 3.0 2.5 2.0 1.5 1.0 0.5
f1 (ppm)

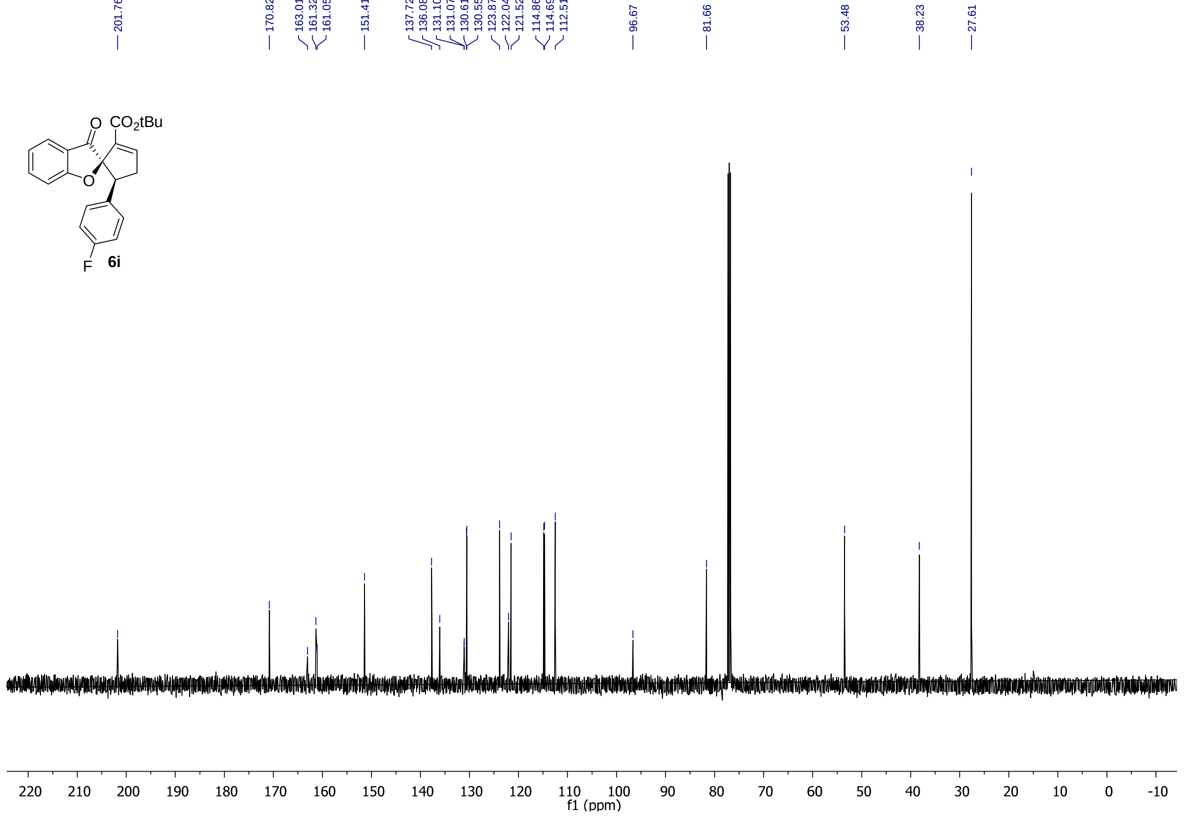

201.76
170.82
163.01
161.32
161.05
151.41
137.72
136.08
131.10
131.07
130.61
130.55
123.87
122.04
121.52
114.86
114.69
112.51
96.67
81.66
53.48
38.23
27.61
CO2tBu
O
O
F
6i
220 210 200 190 180 170 160 150 140 130 120 110 100 90 80 70 60 50 40 30 20 10 0 -10
f1 (ppm)

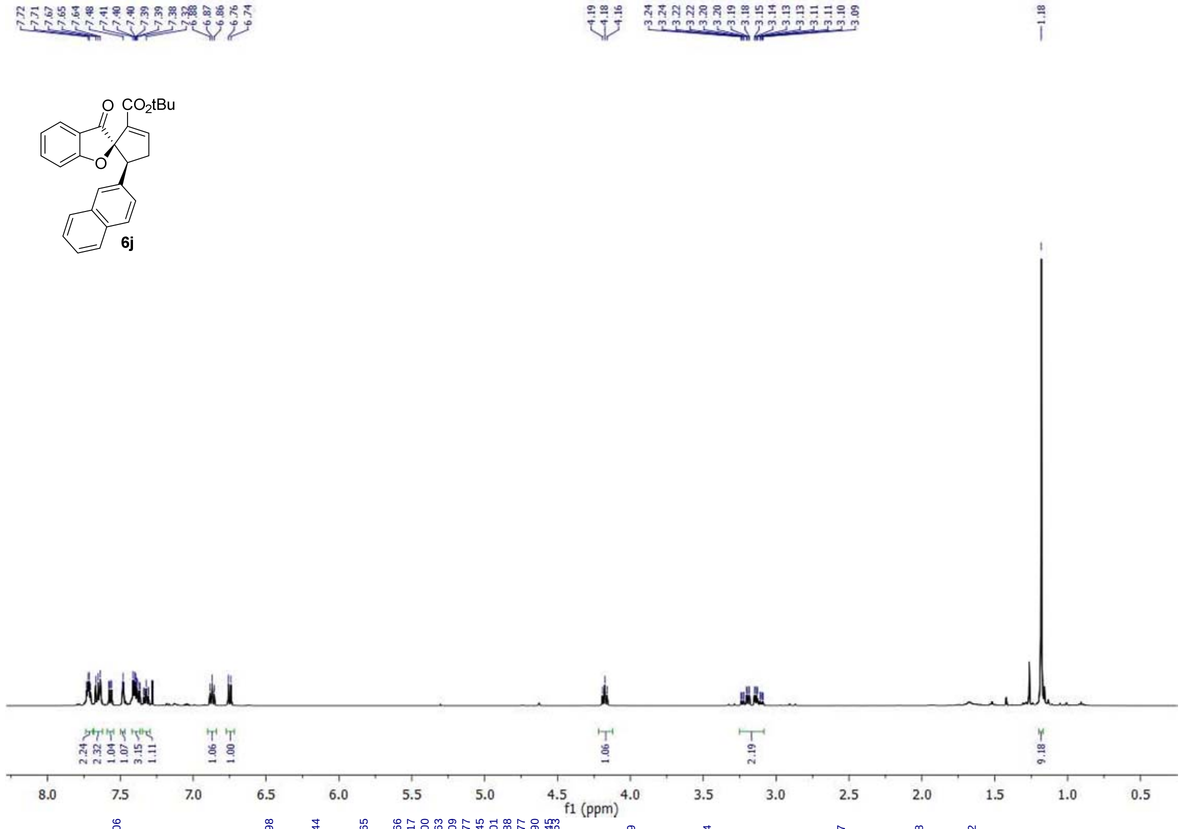

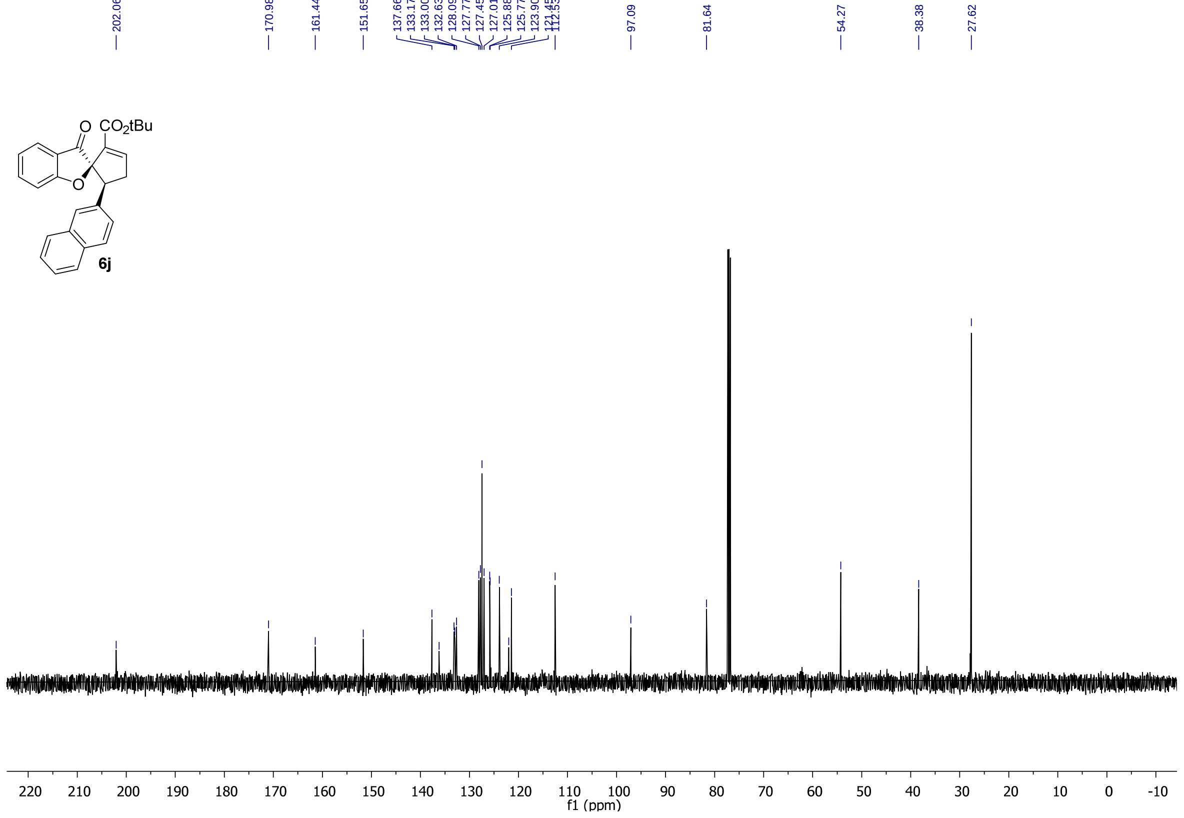

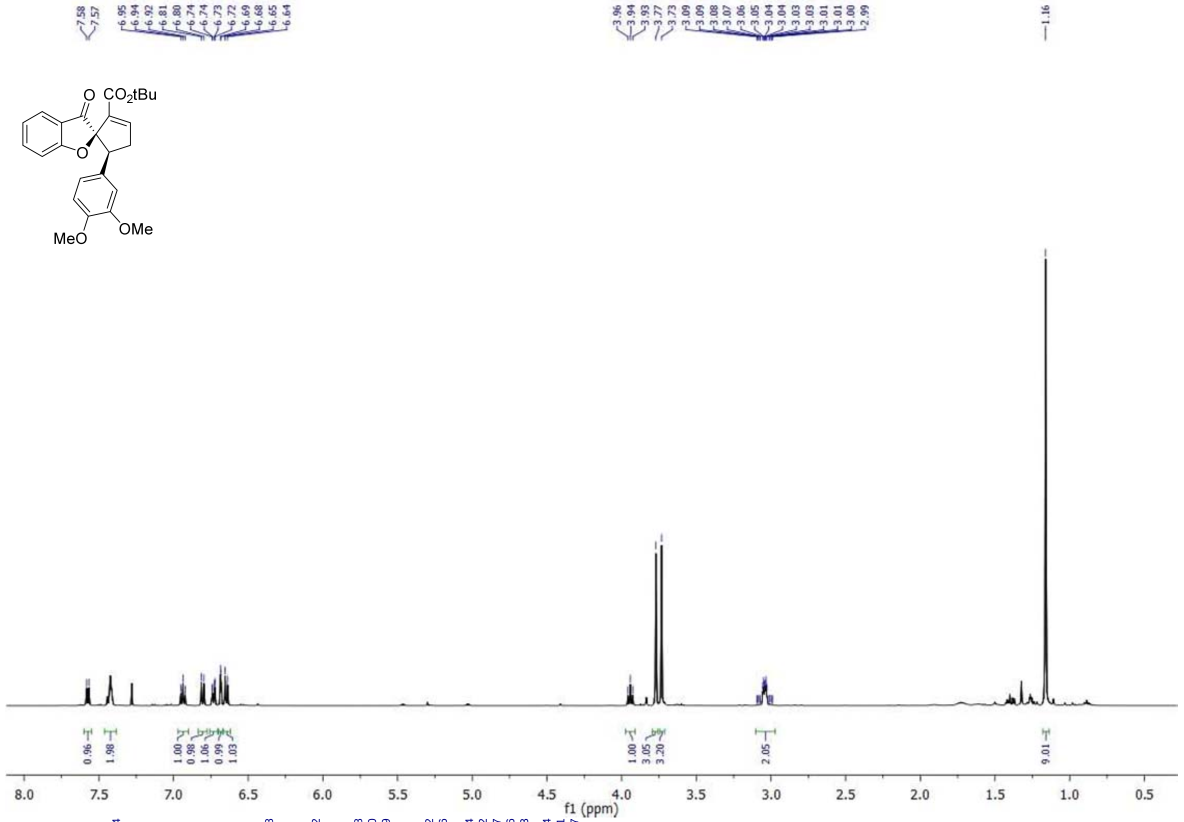

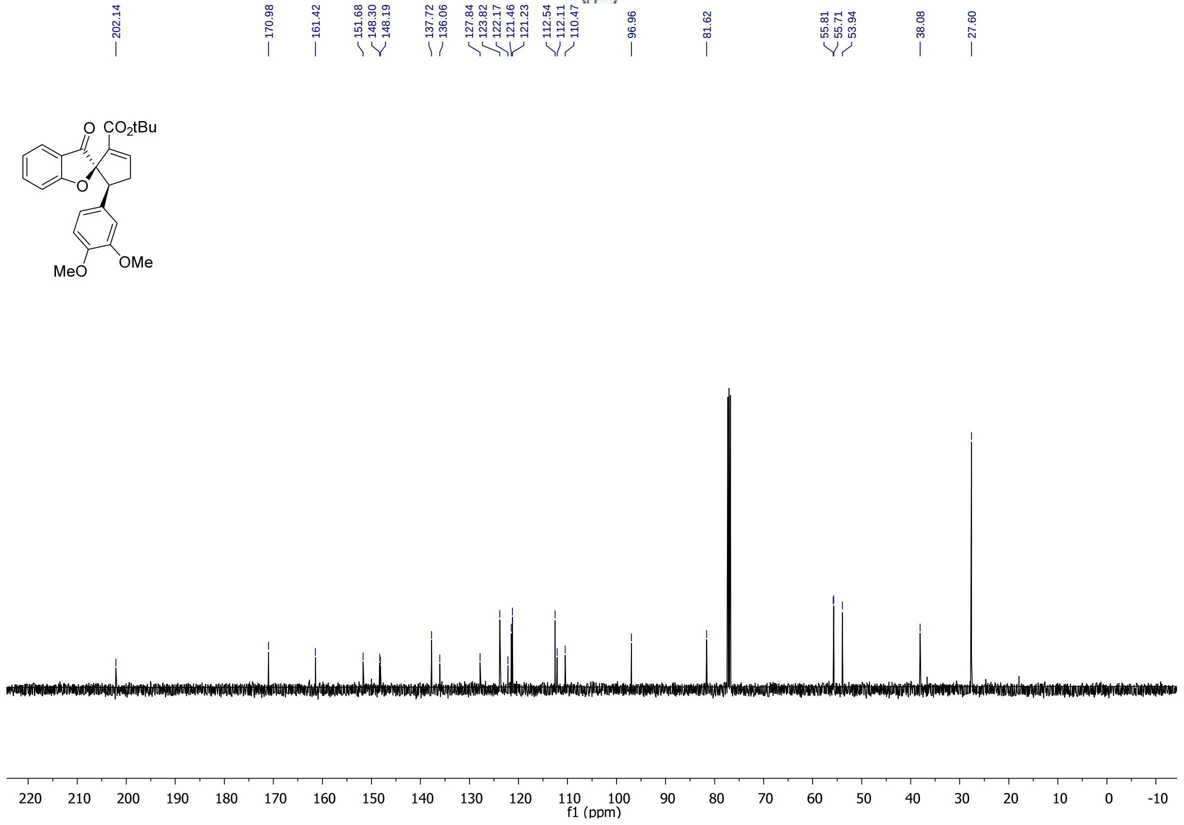

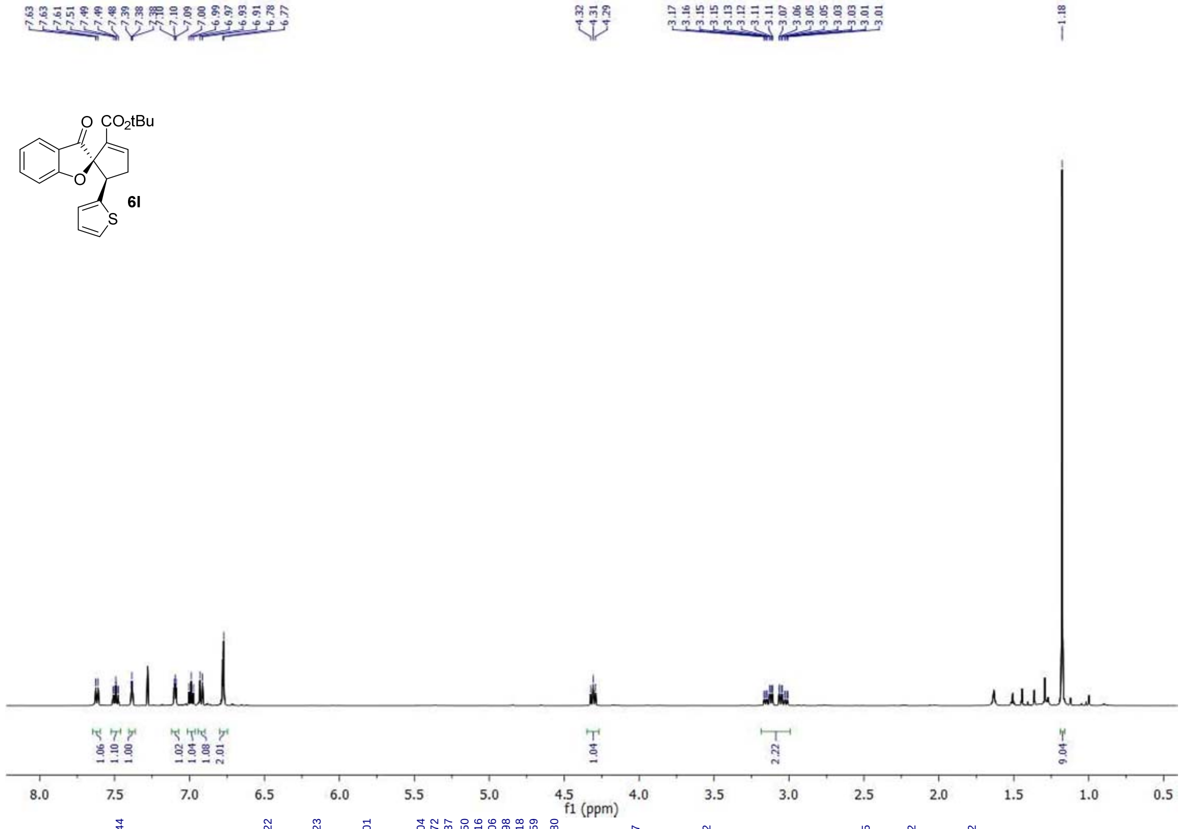

CO2tBu
O
O
S
6l
4.32
4.31
4.29
3.17
3.16
3.15
3.13
3.12
3.11
3.11
3.07
3.06
3.05
3.05
3.03
3.01
3.01
1.18
1.06
1.10
1.00
1.02
1.04
1.08
2.01
1.04
2.22
9.04
8.0 7.5 7.0 6.5 6.0 5.5 5.0 4.5 4.0 3.5 3.0 2.5 2.0 1.5 1.0 0.5
f1 (ppm)

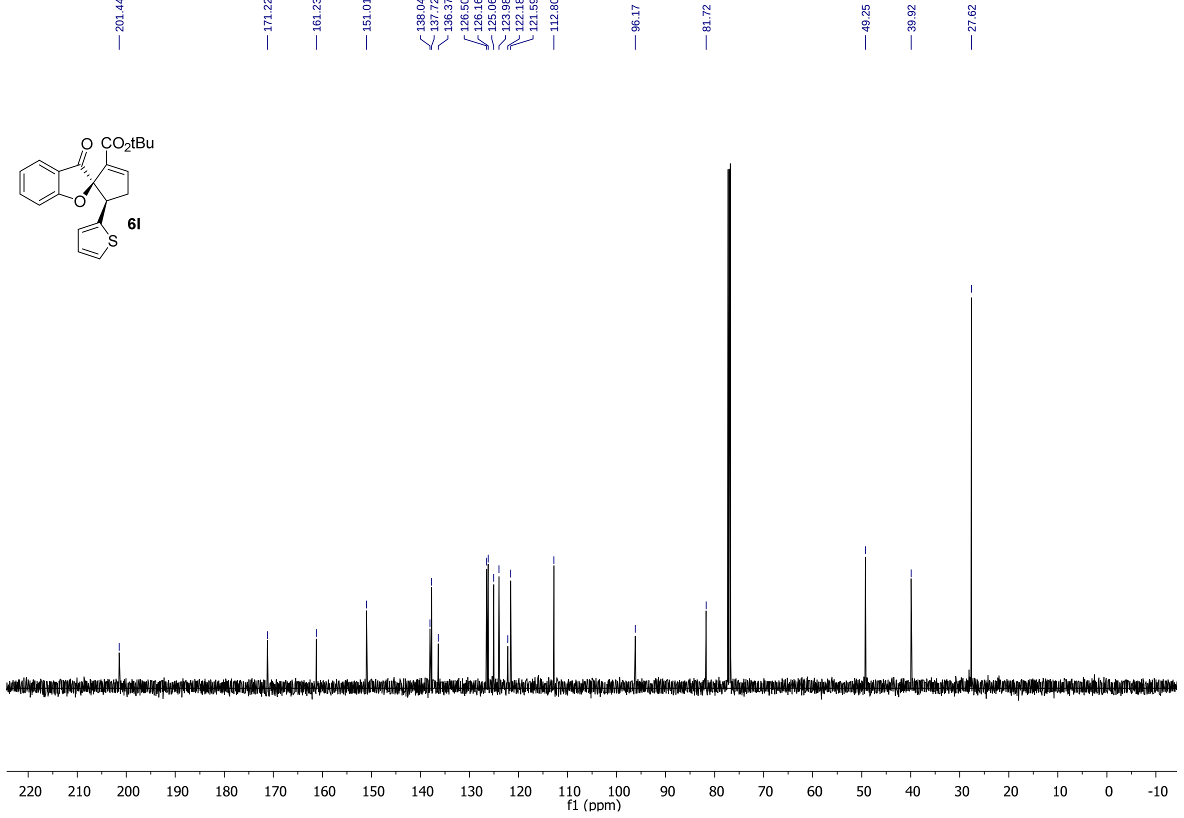

201.44
171.22
161.23
151.01
138.04
137.72
136.37
126.50
126.16
125.06
123.98
122.18
121.59
112.80
96.17
81.72
49.25
39.92
27.62
CO2tBu
O
O
S
6l
220 210 200 190 180 170 160 150 140 130 120 110 100 90 80 70 60 50 40 30 20 10 0 -10
f1 (ppm)

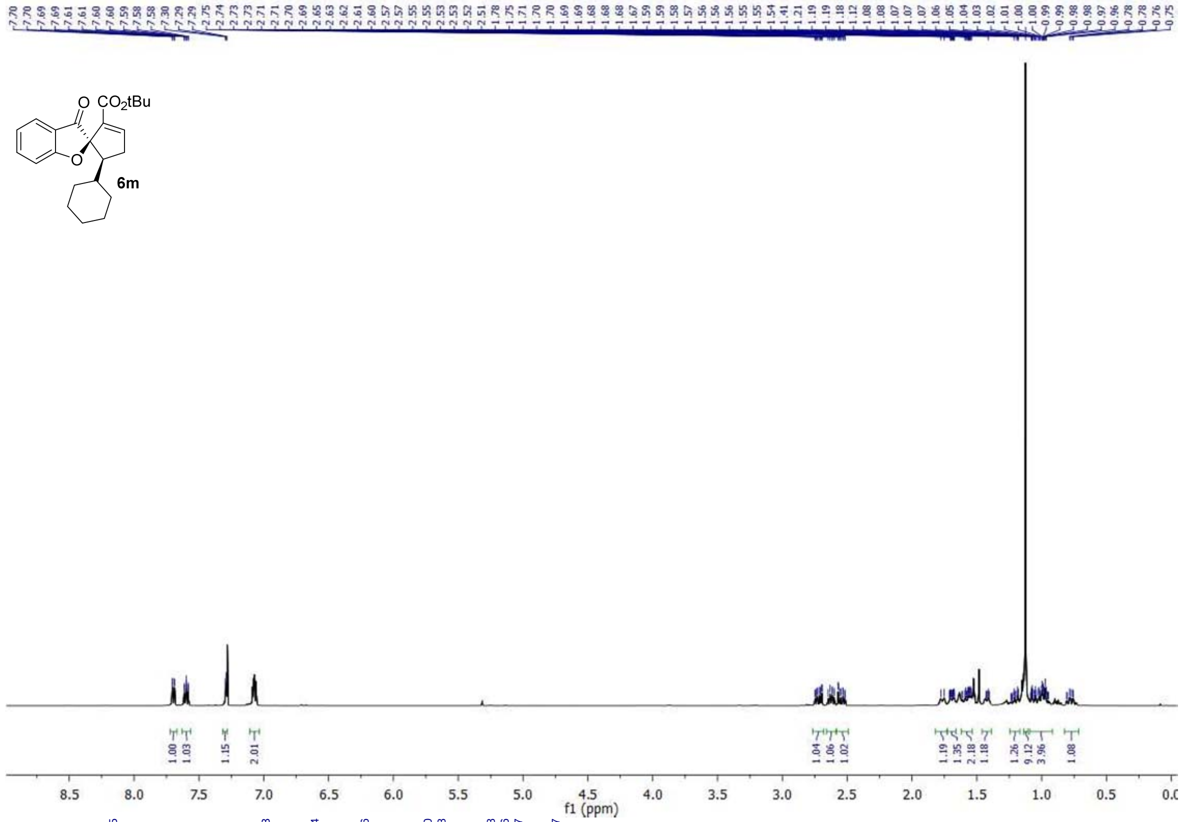
7.70 7.70 7.69 7.69 7.61 7.60 7.60 7.59 7.58 7.58 7.30 7.29 7.29 2.75 2.74 2.73 2.73 2.71 2.71 2.70 2.69 2.65 2.63 2.62 2.61 2.60 2.57 2.57 2.55 2.55 2.53 2.53 2.52 2.51 1.78 1.75 1.71 1.70 1.69 1.69 1.68 1.68 1.67 1.59 1.59 1.58 1.57 1.56 1.56 1.56 1.55 1.55 1.54 1.41 1.21 1.19 1.19 1.18 1.12 1.08 1.08 1.07 1.07 1.07 1.06 1.05 1.04 1.03 1.02 1.01 1.00 0.99 0.99 0.98 0.98 0.97 0.96 0.78 0.76 0.75
O
CO2tBu
O
6m
1.00
1.03
1.15
2.01
1.04
1.06
1.02
1.19
1.35
2.18
1.18
1.26
9.12
3.96
1.08
8.5 8.0 7.5 7.0 6.5 6.0 5.5 5.0 4.5 4.0 3.5 3.0 2.5 2.0 1.5 1.0 0.5 0.0
f1 (ppm)

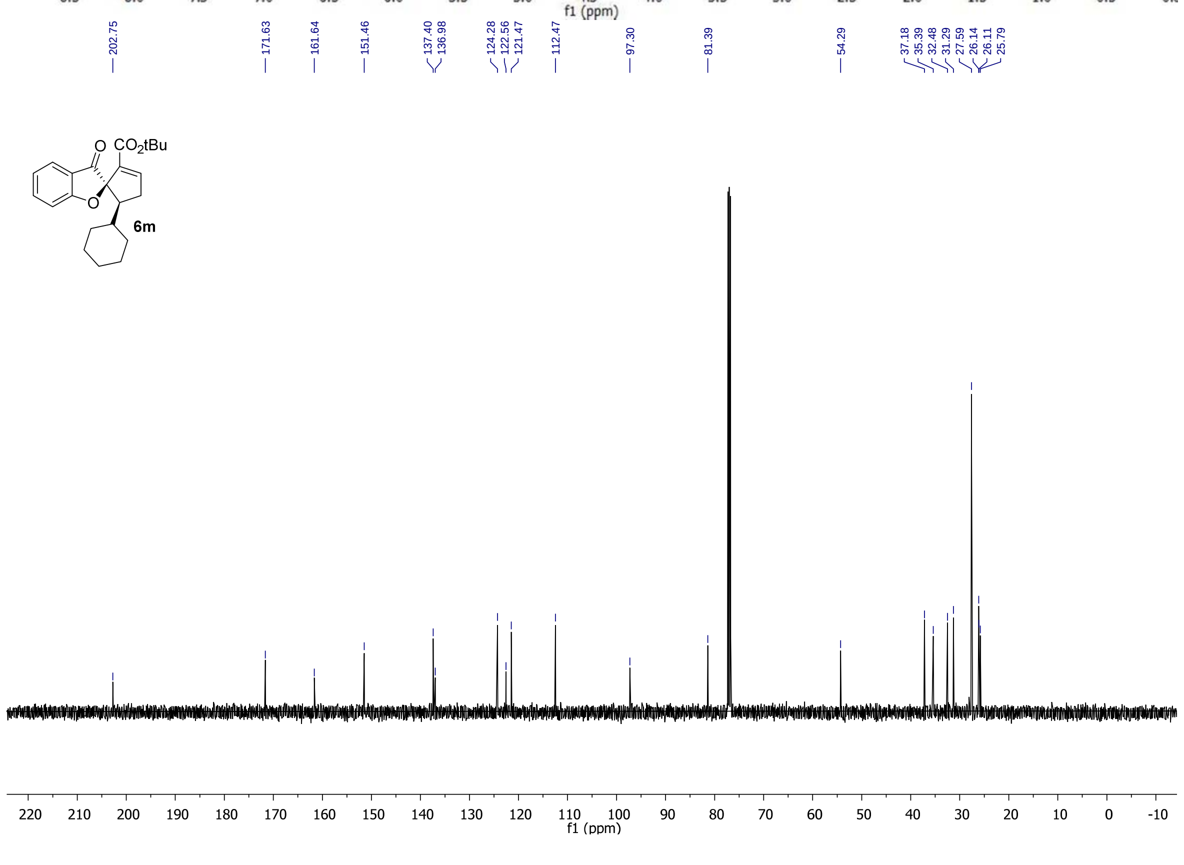
202.75
171.63
161.64
151.46
137.40
136.98
124.28
122.56
121.47
112.47
97.30
81.39
54.29
37.18
35.39
32.48
31.29
27.59
26.14
26.11
25.79
O
CO2tBu
O
6m
220 210 200 190 180 170 160 150 140 130 120 110 100 90 80 70 60 50 40 30 20 10 0 -10
f1 (ppm)

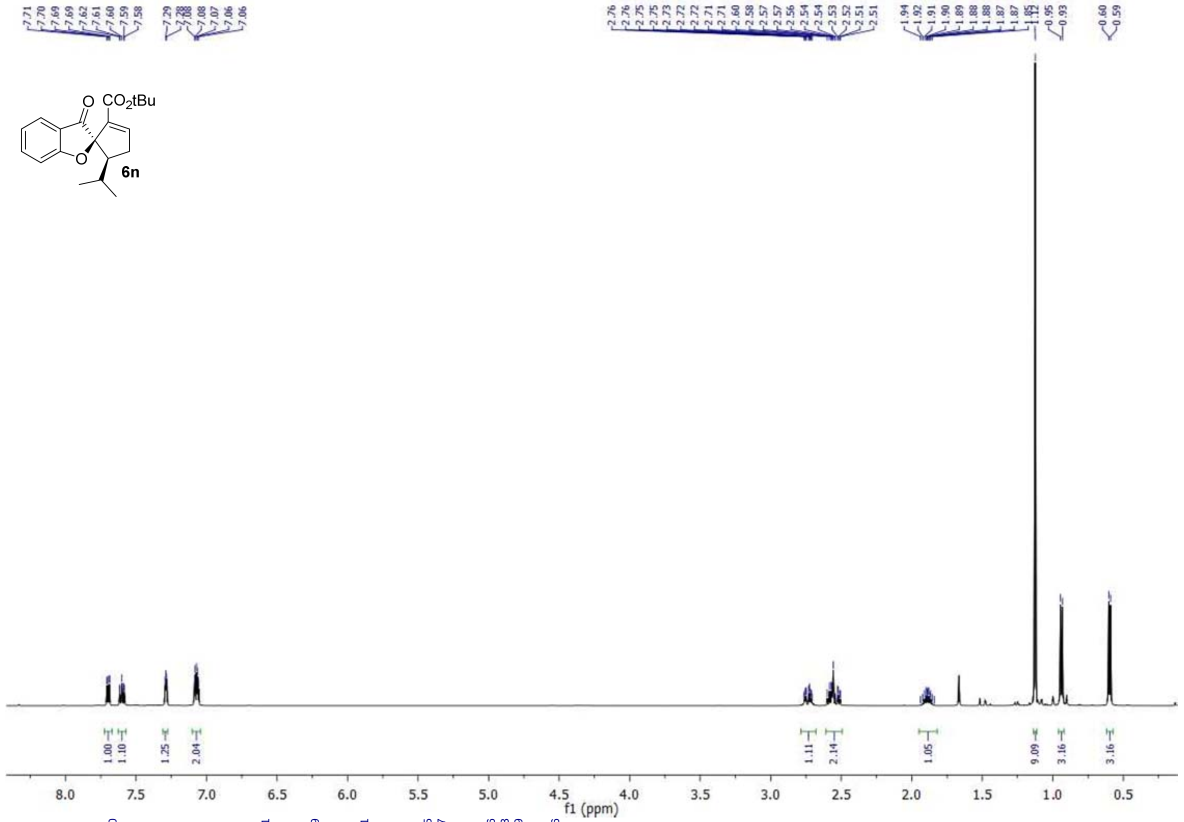
7.71 7.70 7.69 7.69 7.62 7.61 7.60 7.59 7.58 7.29 7.08 7.08 7.07 7.06 7.06
2.76 2.76 2.75 2.73 2.72 2.72 2.71 2.60 2.58 2.57 2.57 2.56 2.54 2.54 2.53 2.52 2.51 2.51
1.94 1.92 1.91 1.90 1.89 1.88 1.88 1.87 1.87 1.12 0.95 0.93 0.60 0.59
CO2tBu
6n
1.00 1.10 1.25 2.04 1.11 2.14 1.05 9.09 3.16 3.16
f1 (ppm)

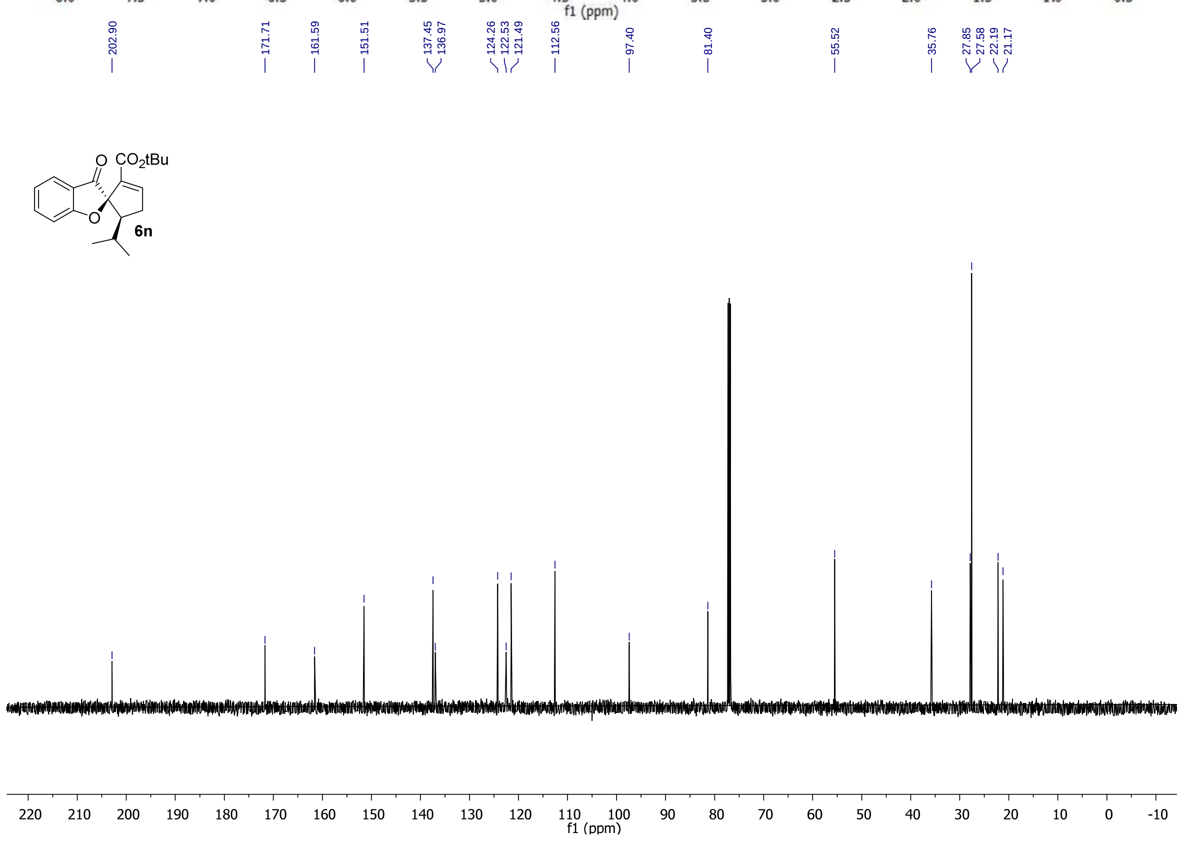
202.90 171.71 161.59 151.51 137.45 136.97 124.26 122.53 121.49 112.56 97.40 81.40 55.52 35.76 27.85 27.58 22.19 21.17
CO2tBu
6n
f1 (ppm)

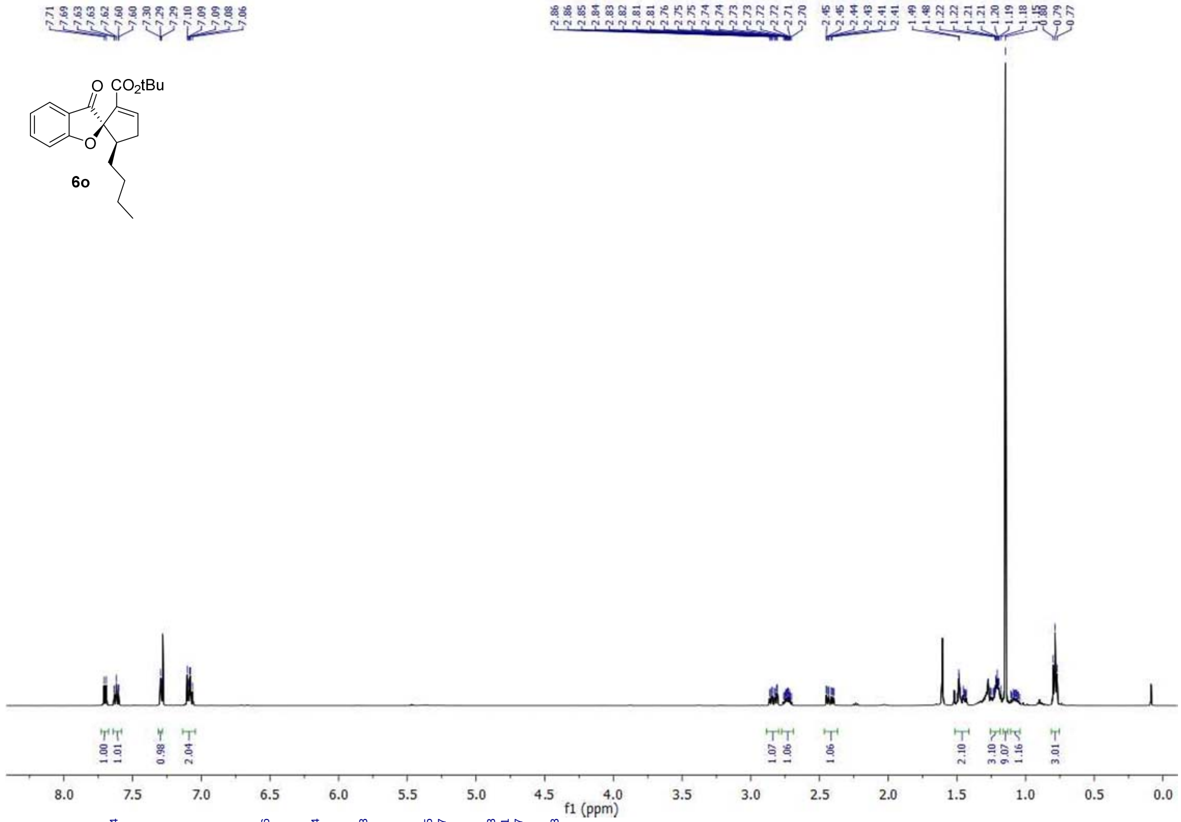

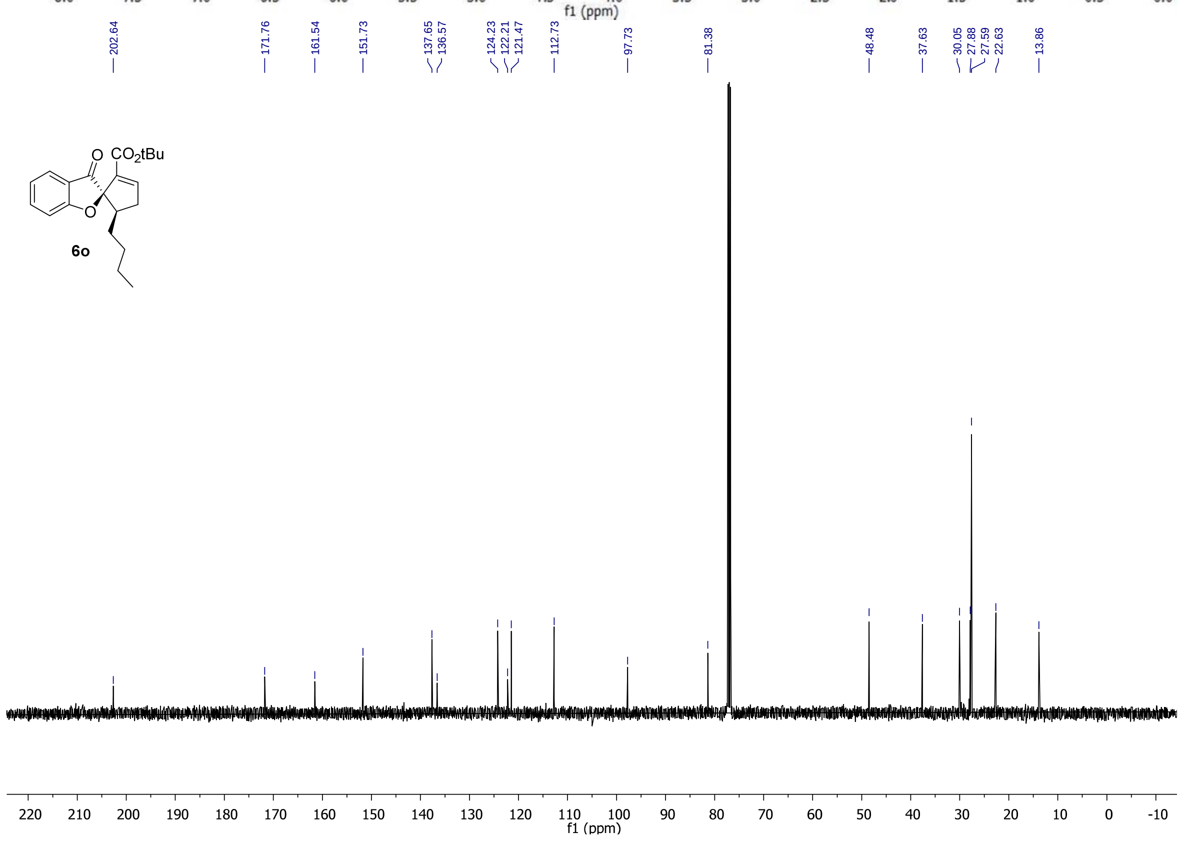

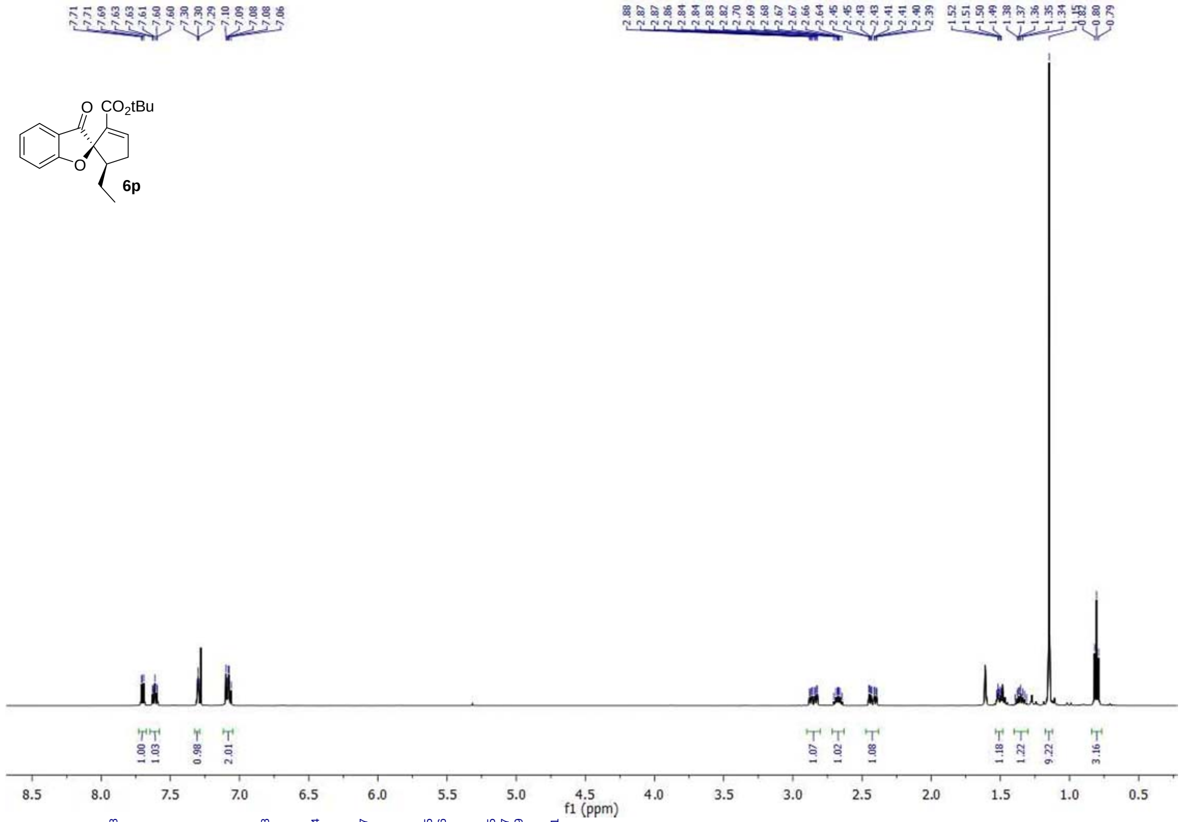

O CO2tBu
O
6p
7.71 7.71 7.69 7.63 7.63 7.61 7.60 7.30 7.30 7.29 7.10 7.09 7.08 7.08 7.06
2.88 2.87 2.86 2.84 2.84 2.83 2.82 2.70 2.69 2.68 2.67 2.66 2.64 2.45 2.43 2.43 2.41 2.40 2.39
1.52 1.51 1.50 1.49 1.38 1.37 1.36 1.35 1.34 1.15 0.82 0.80 0.79
1.00 1.03 0.98 2.01 1.07 1.02 1.08 1.18 1.22 9.22 3.16
8.5 8.0 7.5 7.0 6.5 6.0 5.5 5.0 4.5 4.0 3.5 3.0 2.5 2.0 1.5 1.0 0.5
f1 (ppm)

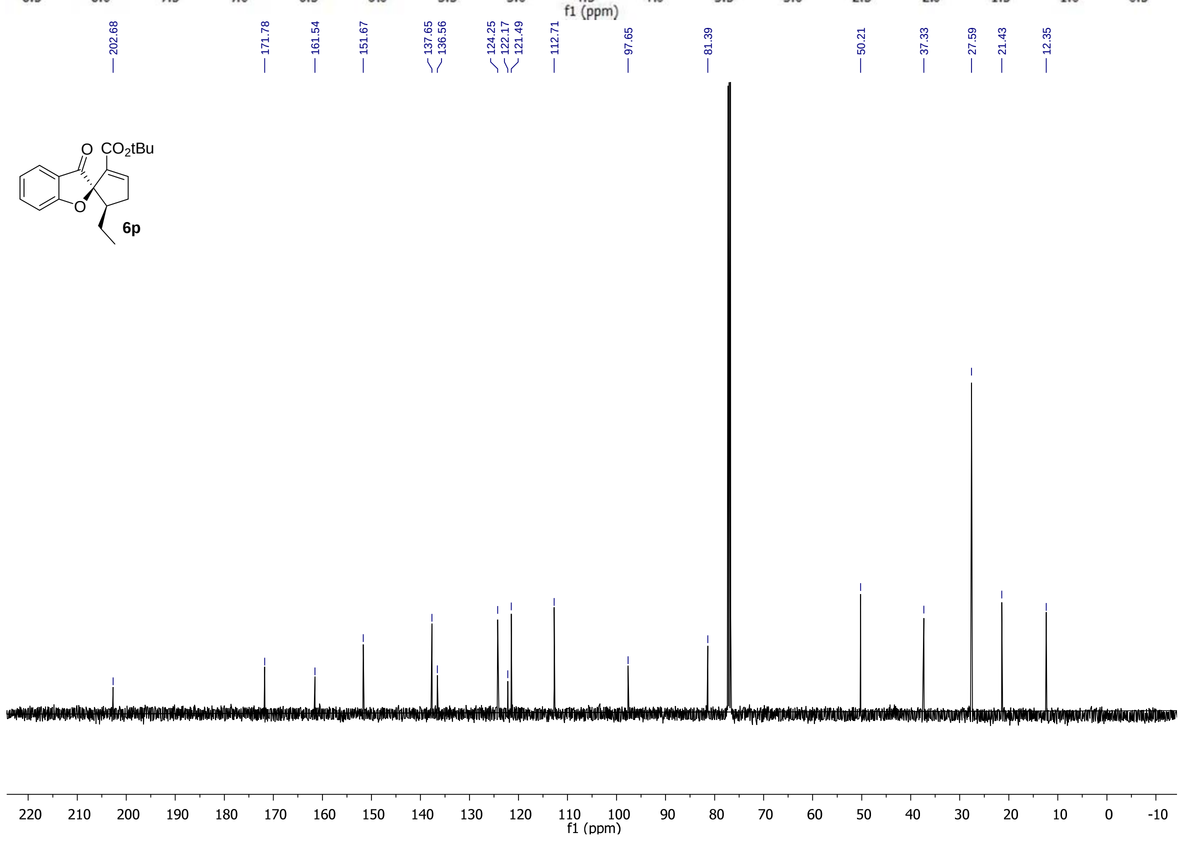

202.68 171.78 161.54 151.67 137.65 136.56 124.25 122.17 121.49 112.71 97.65 81.39 50.21 37.33 27.59 21.43 12.35
O CO2tBu
O
6p
220 210 200 190 180 170 160 150 140 130 120 110 100 90 80 70 60 50 40 30 20 10 0 -10
f1 (ppm)

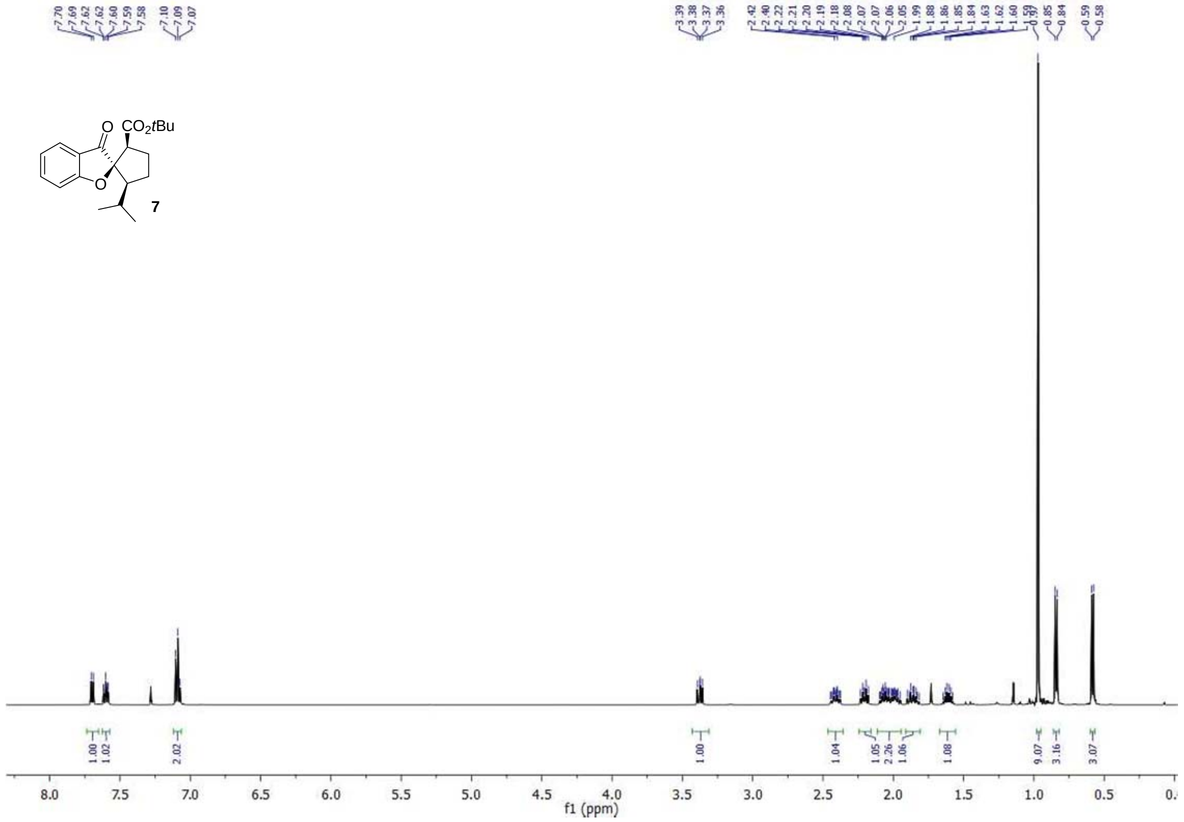
CO2tBu
O
O
7
7.70
7.69
7.62
7.60
7.59
7.58
7.10
7.09
7.07
3.39
3.38
3.37
3.36
2.42
2.40
2.22
2.21
2.20
2.19
2.18
2.08
2.07
2.07
2.06
2.05
1.99
1.88
1.86
1.85
1.84
1.63
1.62
1.60
0.97
0.85
0.84
0.59
0.58
1.00
1.02
2.02
1.00
1.04
1.05
2.26
1.06
1.08
9.07
3.16
3.07
8.0
7.5
7.0
6.5
6.0
5.5
5.0
4.5
4.0
3.5
3.0
2.5
2.0
1.5
1.0
0.5
f1 (ppm)

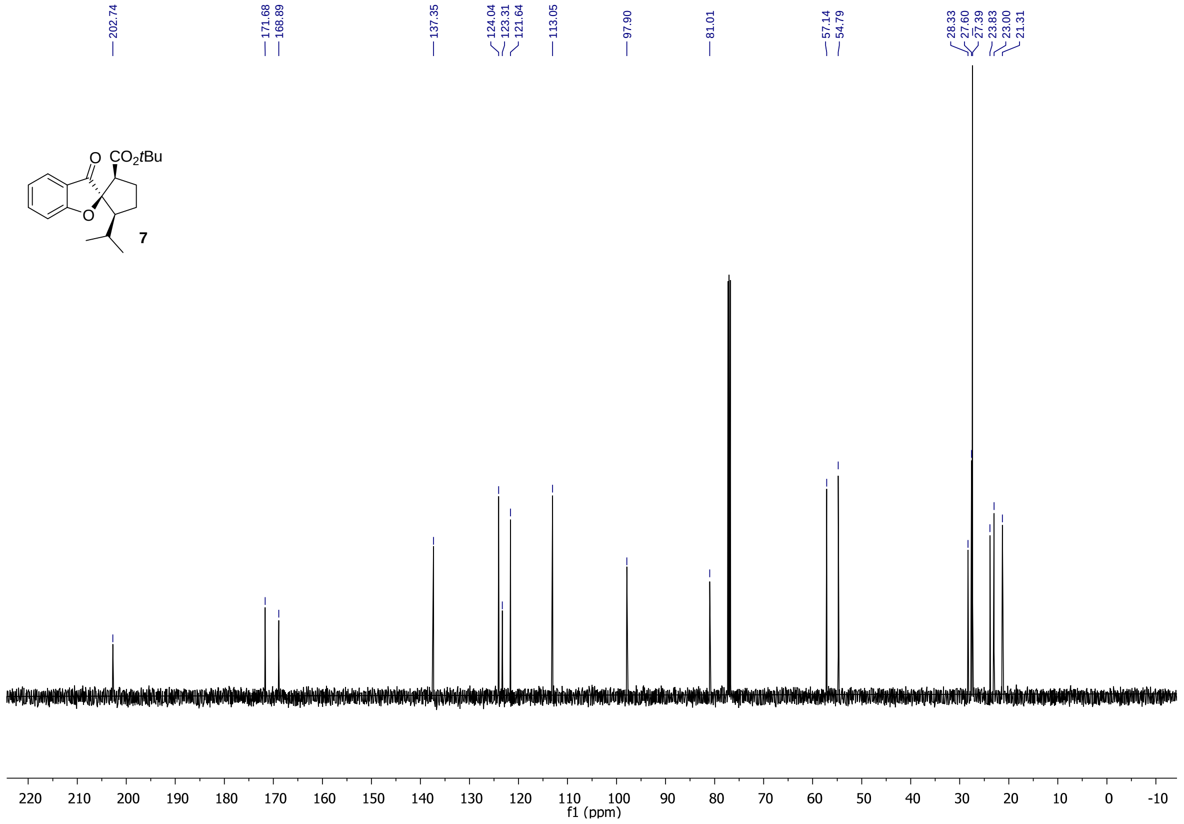
202.74
171.68
168.89
137.35
124.04
123.31
121.64
113.05
97.90
81.01
57.14
54.79
28.33
27.60
27.39
23.83
23.00
21.31
CO2tBu
O
O
7
220
210
200
190
180
170
160
150
140
130
120
110
100
90
80
70
60
50
40
30
20
10
0
-10
f1 (ppm)

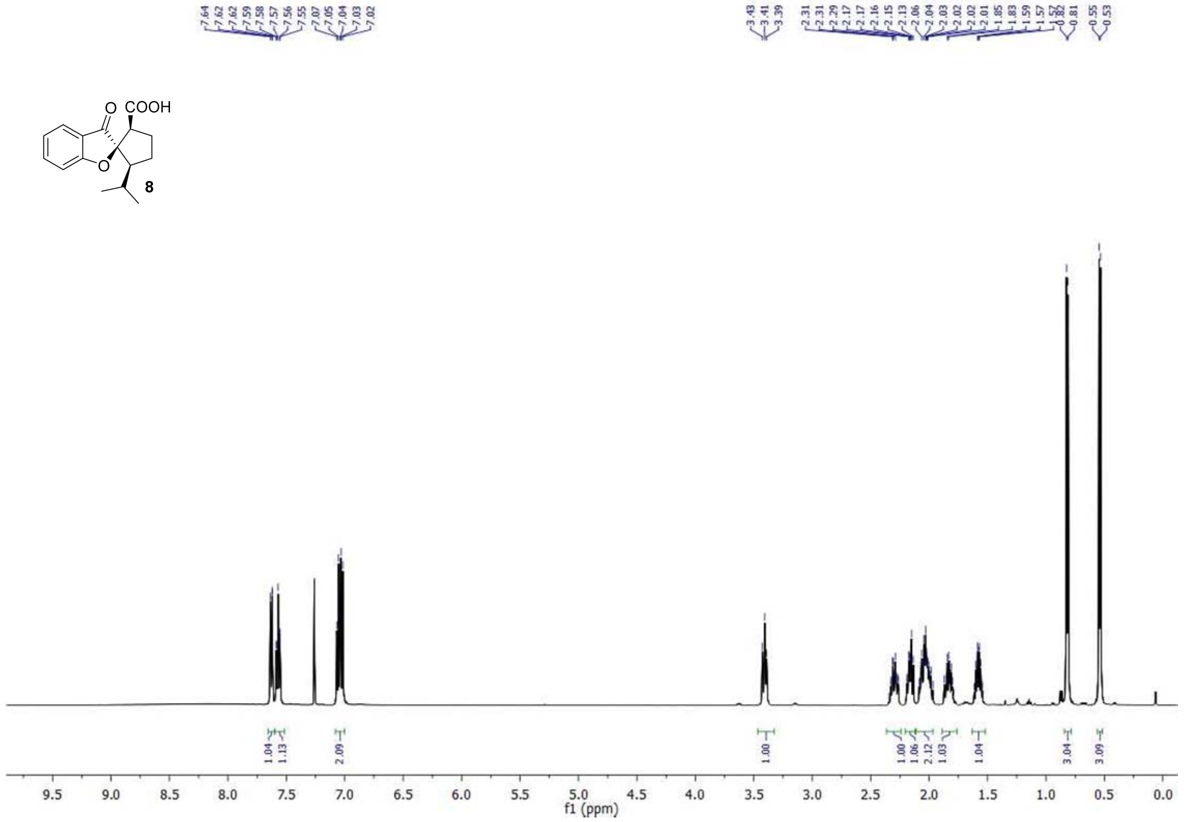

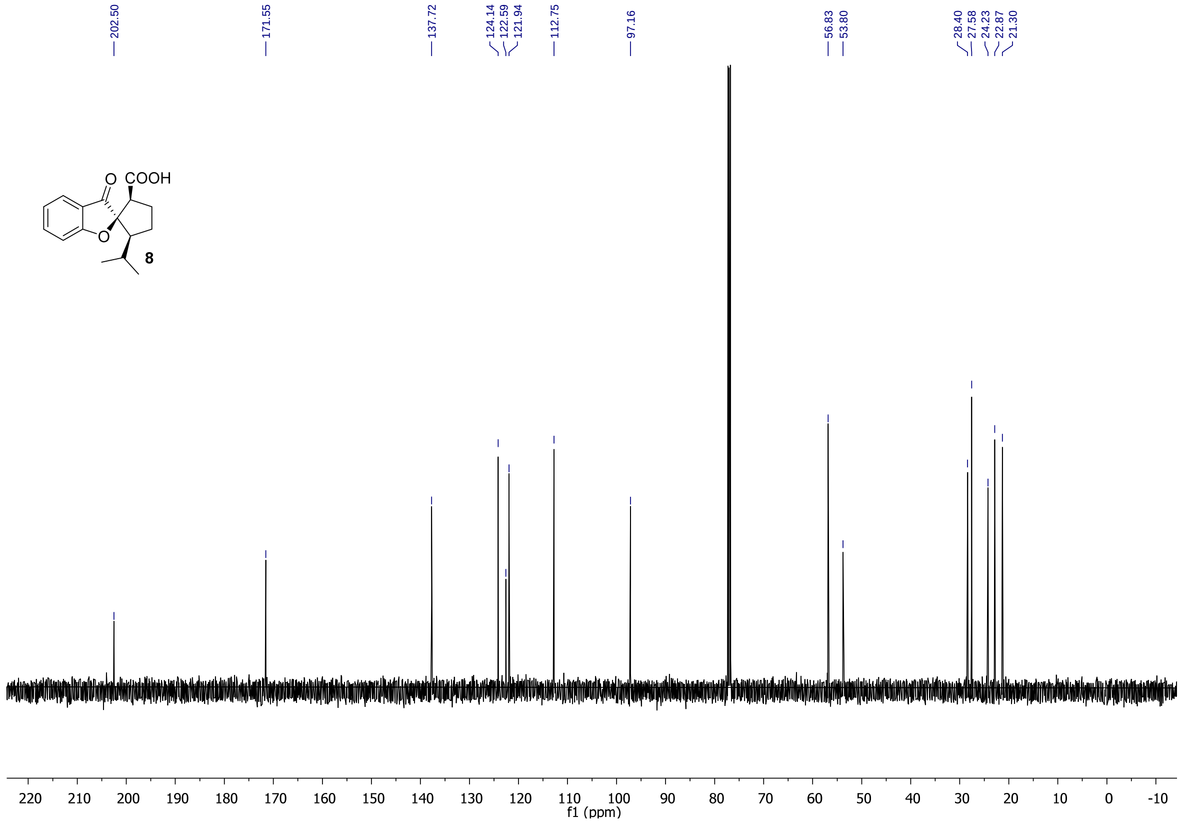

Supplement: Supplementary file 1 [file SC-008-C7SC02176C-s001.pdf]
